# The decline of child stunting in 122 countries: a systematic review of child growth studies since the 19th century

**DOI:** 10.1136/bmjgh-2024-018607

**Published:** 2026-02-18

**Authors:** Eric B Schneider, Juliana Jaramillo Echeverri, Matthew Purcell, Brian A’Hearn, Vellore Arthi, Matthias Blum, Elizabeth Brainerd, Joseph Capuno, Alexandra Lopez Cermeño, Amílcar Challú, Young-Jun Cho, Tim Cole, Jose Corpuz, Ewout Depauw, Federico Droller, Dieter von Fintel, Joël Floris, Gregori Galofré-Vilà, Bernard Harris, Tim Hatton, Laurent Heyberger, Tuuli Hurme, Kris Inwood, Hanaliis Jaadla, Jan Kok, Michał Kopczynśki, Samuel Lordemus, Brian Marein, Adolfo Meisel-Roca, Stephen Morgan, Stefan Öberg, Kota Ogasawara, José Antonio Ortega, Nuno Palma, Anastasios Papadimitriou, Renato Pistola, Björn Quanjer, Helena Rother, Sakari Saaritsa, Ricardo Salvatore, Kaspar Staub, Pierre van der Eng, Evan Roberts

**Affiliations:** 1 Economic History Department, The London School of Economics and Political Science School of Public Policy, London, UK; 2 Centre for Economic Policy Research, London, UK; 3 Bank of the Republic of Colombia, Bogotá, Colombia; 4 University of Oxford, Oxford, UK; 5 University of California Irvine, Irvine, California, USA; 6 German Medical Association, Berlin, Germany; 7 Brandeis University, Waltham, Massachusetts, USA; 8 University of the Philippines Diliman, Quezon City, Philippines; 9 Lund University, Lund, Sweden; 10 Bowling Green State University, Bowling Green, Ohio, USA; 11 Seoul National University, Gwanak-gu, Seoul, Korea (the Republic of); 12 Population, Policy and Practice Programme, UCL, London, UK; 13 University of Warwick, Coventry, UK; 14 Ghent University, Ghent, Belgium; 15 Universidad de Santiago de Chile, Santiago, Chile; 16 Economics, Stellenbosch University, Stellenbosch, South Africa; 17 University of Zurich, Zürich, Switzerland; 18 Universitat de Valencia, Valencia, Spain; 19 University of Strathclyde, Glasgow, UK; 20 University of Essex, Colchester, UK; 21 Université de Technologie de Belfort-Montbéliard, Belfort, France; 22 Helsingin yliopisto, Helsinki, Finland; 23 University of Guelph, Guelph, Ontario, Canada; 24 University of Cambridge, Cambridge, UK; 25 Radboud Universiteit, Nijmegen, The Netherlands; 26 Uniwersytet Warszawski, Warsaw, Poland; 27 Universitat Luzern, Lucerne, Switzerland; 28 Wake Forest University, Winston-Salem, North Carolina, USA; 29 Universidad del Norte, Barranquilla, Colombia; 30 University of Nottingham, Nottingham, UK; 31 Tokyo Institute of Technology, Meguro, Japan; 32 Universidad de Salamanca, Salamanca, Spain; 33 The University of Manchester, Manchester, UK; 34 Ethniko kai Kapodistriako Panepistemio Athenon, Athens, Greece; 35 Universidade de Lisboa, Lisbon, Portugal; 36 International Trademark Association, New York City, New York, USA; 37 Universidad Torcuato Di Tella, Buenos Aires, Argentina; 38 Australian National University, Canberra, Australian Capital Territory, Australia; 39 University of Minnesota Twin Cities, Minneapolis, Minnesota, USA

**Keywords:** Stunting, Child health, Medical demography, Public Health, Nutrition

## Abstract

**Introduction:**

Child stunting, a measure of malnutrition, is a major global health challenge affecting 148.1 million children in 2022. Global stunting rates have declined from 47.2% in 1985 to 22.3% in 2022; however, trends before the mid-1980s are unclear, including whether child stunting was previously prevalent in current high-income countries (HICs). We conducted a systematic review of child growth studies before 1990 to reconstruct historical rates of child stunting.

**Methods:**

We included reports of mean height by age and sex for children up to age 10.99 years. We excluded studies that were not representative of the targeted population and data for children under age 2. Stunting rates were computed by converting the means and SDs of height to height-for-age Z-scores (HAZ) using the WHO standard/reference, combining the HAZ distributions for all ages and measuring the share of the combined distribution below the stunting threshold.

**Results:**

We found 923 child growth studies at the community, regional and national level covering 122 countries from 1814 to 2016. We supplemented these historical studies with stunting estimates from the 1990s onward from the Joint Malnutrition Estimates database. Many current HICs had high levels of child stunting in the early 20th century, similar to low- and middle-income countries (LMICs) today. However, there was heterogeneity: stunting rates were low in Scandinavia, the European settler colonies and in the Caribbean, higher in Western Europe and exceptionally high in Japan and South Korea. Child stunting declined across the 20th century.

**Conclusion:**

The global child stunting rate was substantially higher in the early 20th century than in 1985, and the reduction of child stunting was a central feature of the health transition. The high stunting rates and subsequent reduction of stunting in HICs suggest that current HICs provide lessons for eradicating child stunting and that all LMICs can eliminate stunting.

**WHAT IS ALREADY KNOWN ON THIS TOPIC**

- Global health researchers have estimated child stunting rates from the 1990s onward and traced trends in mean adult stature from the late 19th century to the present.

**WHAT THIS STUDY ADDS**

- This study computes comparable child stunting rates for 122 countries across the 20th century, extending our understanding of child stunting much deeper into the past.

**HOW THIS STUDY MIGHT AFFECT RESEARCH, PRACTICE OR POLICY**

- This study highlights new countries that either have eradicated stunting from high levels (Japan) or have had low stunting rates in the early stages of development (the Caribbean) that could provide further evidence on how to eradicate child stunting today.
- These historical stunting rates may also affect how researchers interpret population ageing today, since the elderly today had different experiences of malnutrition in childhood.
- Our findings highlight the importance of changing hygiene behaviours for eradicating stunting.

## Introduction

 Poor child health remains an urgent global problem with an estimated 148.1 million children stunted in 2022.^1^ Stunted children grow too slowly and are shorter than healthy children of the same age. Stunted growth reflects poor nutrition, a virulent disease environment and chronic illness in childhood and leads to poor health and human capital outcomes later in life.^2 3^ Child stunting rates, the share of children under age 5 whose height-for-age Z-score (HAZ) is below −2, are a key indicator for Sustainable Development Goal 2.2. Global stunting rates have fallen from 47.2% in 1985 to 22.3% in 2022,^4^ with stunting currently most prevalent in sub-Saharan Africa and South and Southeast Asia. Although a wide number of nutritional surveys have been implemented to track child stunting since the mid-1980s, there is very limited evidence on stunting rates before then.

This study reconstructs the evolution of child stunting for 122 countries before 1985 and extends existing evidence in three important ways. First, evidence on the secular increase in adult stature suggests that reductions in child stunting may have been a part of the historical health transition in current high-income countries (HICs).^5^,^8^ However, without historical estimates of child stunting for these countries, it is not possible to compare the levels or rates of stunting decline over time or assess the causes of stunting in the past.^9^ Second, several studies have scrutinised the past 30 years for countries that have experienced a rapid decline in stunting.^10^ Extending estimates of child stunting into the past may highlight other exemplar countries that provide insights into reducing child stunting today. Finally, past cross-country change in stunting levels may indicate how quickly stunting rates can be reduced in the future.

## Methods

### Search strategy and selection criteria

We conduct a systematic review of child growth studies published since the late 19th century. Our search strategy builds on the existing stunting estimates from the Joint Malnutrition Estimates (JME) database compiled by the WHO, World Bank and UNICEF, which contains stunting estimates for low- and middle-income countries (LMICs) from the 1990s onward.^11^ We supplement the JME data in three ways: (1) for all countries, we included child growth studies published before the 1990s; (2) we included studies of historical child growth from unpublished, archival data; and (3) we included studies after 1990 for HICs missing from the JME database.

The search strategy sought to balance completeness of coverage with the time cost of accessing historical, non-digitised publications, which had to be physically consulted in libraries or requested via interlibrary loan. We started by searching Google Scholar and PubMed for recent growth references, studies on the secular change in stature and child growth studies published before 1990 for each country. We also used historical bibliographies of child growth studies to find earlier references. Because many historical studies were not indexed, we built from this initial search by tracing references that the discovered studies cited. Where forward citation was available because the article had been indexed, we also tracked references forward (full details of search strategy in online supplemental appendix A). To aid in this effort, a team of 43 data contributors was recruited, whose expertise covered 34 countries around the world and who were fluent in 17 languages. The corresponding author and his team added a further 88 countries.

Online supplemental appendix B provides the full inclusion and exclusion criteria and their rationale. The inclusion criteria were that the study (1) reported mean heights by age; (2) contained data for children up to age 10.99 years; (3) reported data numerically in tables; (4) reported height separately by sex; (5) had a sample size in each 1 year age group by sex cell greater than 20; and (6) reported the children’s birth year. The inclusion criteria were assessed independently by one reviewer and later systematically checked by the corresponding author, given their objective criteria. Growth measurements were compared against the WHO 2006 child growth standard and 2007 growth reference for school-aged children, which were constructed to fit seamlessly together.^12^ Note we excluded most studies that reported stunting rates relative to superseded growth references such as the old WHO or 1977 National Center for Health Statistics (NCHS) growth reference because of the difficulty in converting these stunting rates to rates relative to the 2006/2007 WHO child growth standard/reference (see online supplemental appendix B.1.2 for details).

Including children older than age 5.0 differs from typical stunting definitions and is necessary to increase the number of historical studies. Early studies of child growth were often conducted in primary schools because there was no sample frame and schools were a convenient place to measure large groups of children (see online supplemental appendix B.1.2). By including these children, we assume that stunting over age 5 is closely correlated with stunting under age 5. This assumption is justified because existing literature shows that at the population level, relative height loss (growth faltering) compared with the WHO standard occurs before age 2 and remains stable afterwards.^13^ Thus, we would not expect children over age 5 to experience further faltering. Whether children over age 5 can experience catch-up growth is more controversial.^14^ However, while recent research suggests that catch-up growth is possible,^15 16^ it appears to be rare and is unlikely to bias stunting rates over age 5.^17^ We exclude children over age 11 since differences in the timing of the pubertal growth spurt may distort children’s position relative to the growth reference.^18 19^

Data contributors provided data for each study in standardised forms. Typical studies provided, by age and sex, the sample size, the mean height and a measure of the dispersion of height, for example, SD, frequency distribution or percentiles. Very few studies provided individual-level data, and those that did were often excluded because they were non-representative. The data contributor also completed a questionnaire on the quality and representativeness of each study, providing systematic information about the temporal, spatial and urban-rural coverage of the study, along with study characteristics relating to potential measurement error: whether information was available for both sexes, whether heights were measured with shoes and whether ages were rounded down to the nearest whole year or not. The data contributor also answered detailed questions about the representativeness of the study with particular attention paid to selection based on socioeconomic or health status (see online supplemental appendix C for full details).

There were two exclusion criteria. First, we excluded studies where there was evidence of selection into the sample on socioeconomic status (SES) or health, the greatest source of potential bias in our analysis. Examples of direct selection were studies that targeted low or high SES groups within a population, for example, when high SES children were targeted to produce a standard of healthy child growth, or when schools were used as a sample frame, but only high SES children attended schools in a particular area.^20^ Judgements on representativeness were based on the study text and expert knowledge of the historical context (see online supplemental appendix B.2.1 for more detail and online supplemental appendix L for examples). We do include community (town and city) and regional (subnational) studies that are not nationally representative, which is necessary to increase historical coverage for most countries: nationally representative studies were only available for 27 and 11 countries before 1950 and 1930, respectively.

Second, we excluded children under age 2. Children under age 2 were often measured in a recumbent position with varying methodologies across studies and over time, adding potential measurement error. In addition, children become stunted, in part, through growth faltering, slower than normal growth between ages 0 and 2, which leads them to fall behind the growth standard. Thus, children aged 0–2 mechanically have a lower stunting rate than children aged 2–5 because they have not yet experienced full growth faltering.^21^ This means that the age composition of the sample used to estimate the stunting rate matters. Given that historical studies cover a wide variety of ages and many do not include children under 2, excluding the younger age groups improved comparability across studies. To match the historical studies, we also use stunting above age 2 for the JME dataset (see online supplemental appendix H for further discussion), meaning stunting rates will be higher in our dataset than what appears in the JME for ages 0–5.

### Data analysis

To estimate stunting rates for each study, we begin by computing means and SDs of height by age and sex for all studies following standard methods (online supplemental appendix C.1). We next conducted validation cross-checks, verifying outlier values in the original studies. We also checked that data contributors had coded the representativeness of datasets consistently (online supplemental appendix D). We adjust the variance of the height distributions by age and sex so that the SD of height reflects an exact midpoint age (4.5) that can be compared with the WHO standard/reference rather than a range of ages (4.00–4.99)^22^ (online supplemental appendix E). Some studies reported SDs of height that were implausible, so we winsorised coefficients of variation of height by age and sex at the 5th and 95th percentiles of all observed coefficients of variation (online supplemental appendix F). 233 studies did not report measures of the dispersion of height, so we imputed SDs for each sex and age using the median coefficient of variation observed in studies that did report measures of dispersion. The error introduced by this procedure is relatively small (online supplemental appendix G.2).

**Figure 1:**
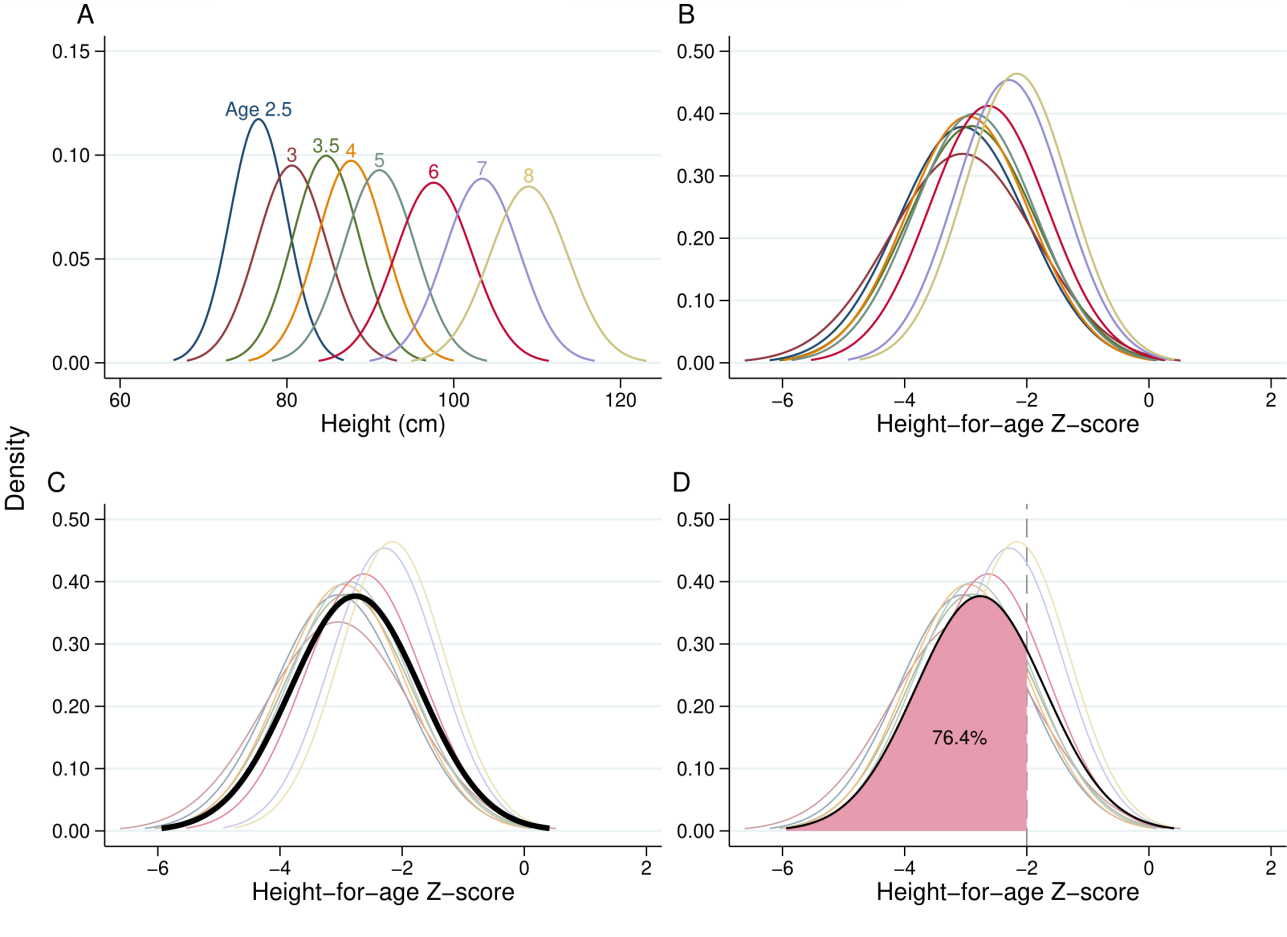
Process of aggregating height distributions at various ages into a single stunting rate. The height distributions are for Guatemalan girls measured in 1977 as part of the Institute of Nutrition of Central America and Panama (INCAP) study. See text and online supplemental appendix G for a complete discussion of methods. Sources: data from Martorell *et al* (1995).^62^

To compute a comparable stunting rate for all studies (figure 1), we first convert the reported mean and SDs of height at each age into HAZ using the 2006/2007 WHO child growth standard/reference.^19 23^ We then compute the mean and SD of the combined distribution, that is, as if all children were drawn from the same distribution, using standard methods and weighting by the sample size at each age where possible. Finally, assuming that the combined HAZ distribution is normal, a reasonable assumption for children aged 2–10, we compute the stunting rate as the area under the combined HAZ distribution below the stunting threshold of −2 (online supplemental appendix G.1). We do these computations for each sex separately and then take the average stunting rate of boys and girls as the final stunting rate. We follow the same procedure when aggregating regional or ethnic-group data, using population weights.

We assess the certainty of evidence for each study by adapting the Grades of Recommendation, Assessment, Development and Evaluation approach for a descriptive study such as our own.^24^ We consider the five domains for downgrading or upgrading the certainty of evidence: risk of bias (excluding non-representative studies); unexplained heterogeneity (where HAZ scores by age differ significantly from one another within a study); indirectness of evidence (data for only one sex, inclusion of children over 5 and spatial coverage of the study); imprecision of the results (small sample size and stunting estimates based on mean height only); and publication bias (not relevant when compiling descriptive data). We use these domains to assign each study a certainty-of-evidence score. Our full approach is described in online supplemental appendix I.

To make country-level comparisons across time, we aggregate studies by birth decade at the country level. Rather than averaging all studies within a country-decade bin, studies with very high certainty-of-evidence scores are weighted more highly than those with lower scores. This ensures that high-quality, nationally representative studies are weighted more prominently than small community studies (online supplemental appendix K). Online supplemental appendix O includes graphs for each country of the study-level certainty-of-evidence scores so that readers can assess the quality of the data underpinning each country-by-decade stunting estimate. Note that data are missing for many birth decades, and we do not impute stunting rates where the data are missing.

## Results

**Figure 2:**
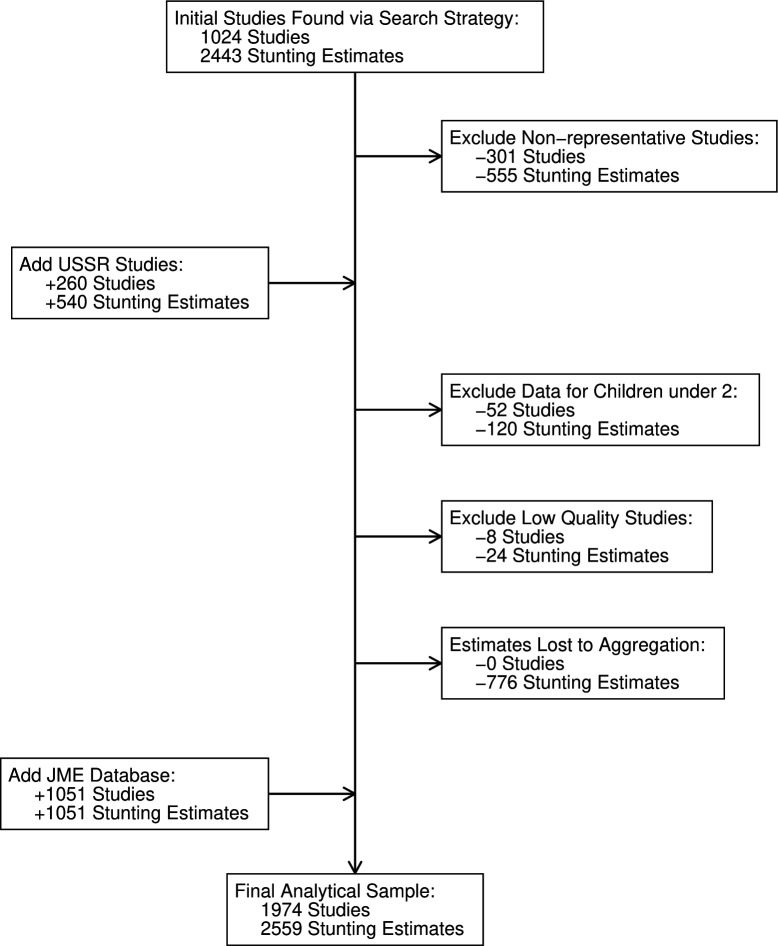
Systematic review results. JME is the Joint Malnutrition Estimates produced by UNICEF, the WHO and the World Bank. Sources: Worldwide Historical Stunting Database and UNICEF/WHO/World Bank (2023). USSR, Union of Soviet Socialist Republics.

**Figure 3:**
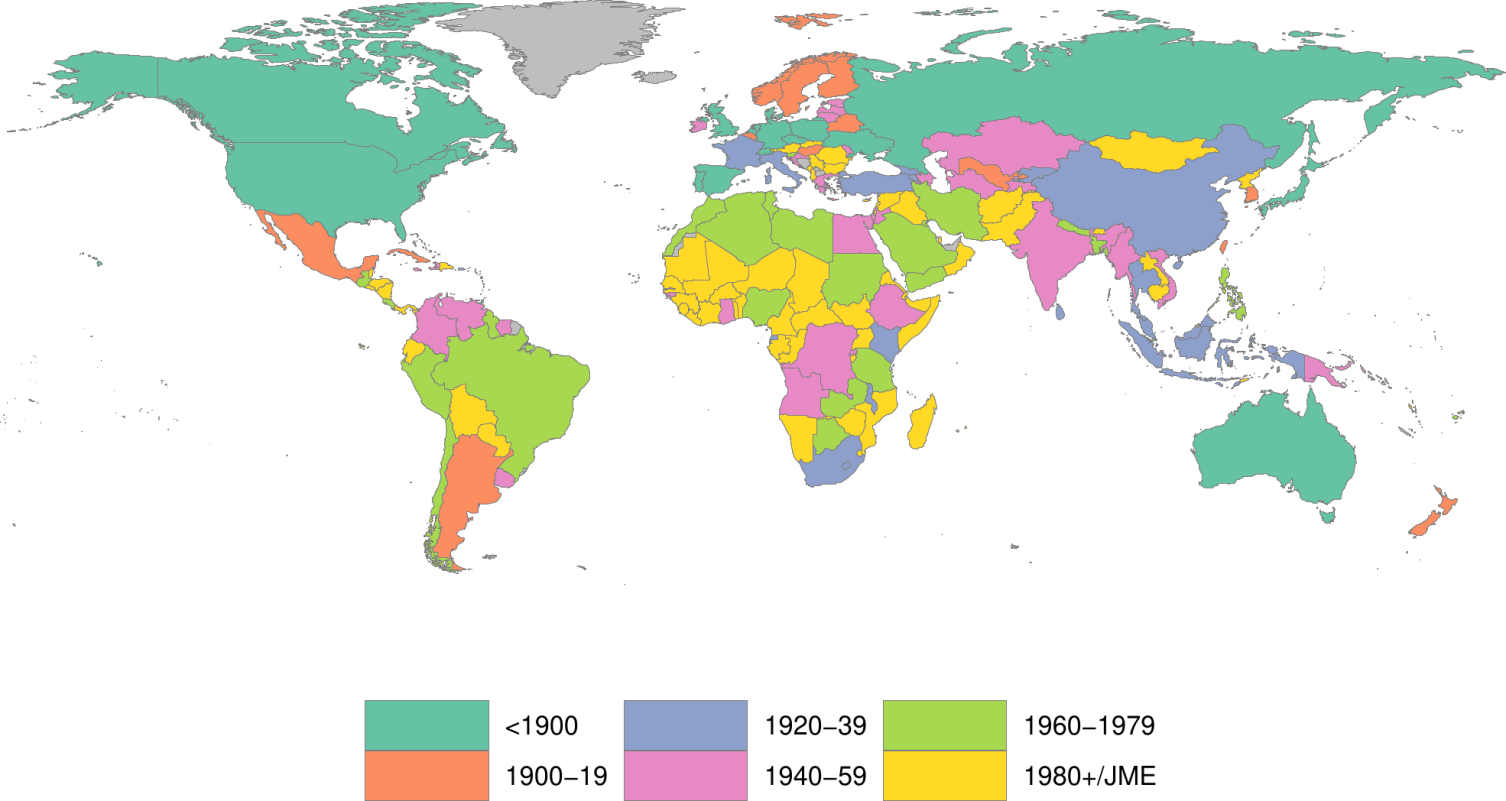
Period of earliest birth cohort under observation in the final analytical sample. JME is the Joint Malnutrition Estimates produced by UNICEF, the WHO and the World Bank. Grey countries have no data. Sources: Worldwide Historical Stunting Database and UNICEF/WHO/World Bank (2023).

In total, we found 1024 studies that met the inclusion criteria, providing 2443 stunting estimates for 122 countries (figure 2). There are more stunting estimates than studies because some studies reported child height for different regions, sub-groups or time periods. Common reasons for seemingly pertinent studies not meeting the inclusion criteria were failure to report tabular data,^25^ only reporting combined data for both sexes^26^ and sample sizes below the minimum requirements.^27^ We excluded 301 studies that were not representative of the study’s target population. We then included 260 studies from the former Union of Soviet Socialist Republics where the representativeness could not be assessed systematically (see online supplemental appendix B.3). We exclude some studies that only reported on children under age 2; others that had low sample sizes and other data quality issues; and we lose some regional stunting estimates where we applied population weights to aggregate to a larger geographic area. The studies in our review reflect the measurement of more than 129 million children. An Excel file with complete details for the 923 studies we contribute is provided in online supplemental materials, including each study’s bibliographic details, sample size and score on the domains of certainty of evidence (online supplemental appendix J). The full data is published online.^28^ Finally, we added 1051 studies from the JME database, providing coverage for LMICs since 1990.^11^ This left us with a total of 1974 studies and 2559 stunting estimates in our final analytical sample. The certainty of evidence improves over time as more nationally representative samples become available, but certainty is rather low for earlier periods. Figure 3 shows the birth cohort range in which we first observe stunting for each country.

**Figure 4:**
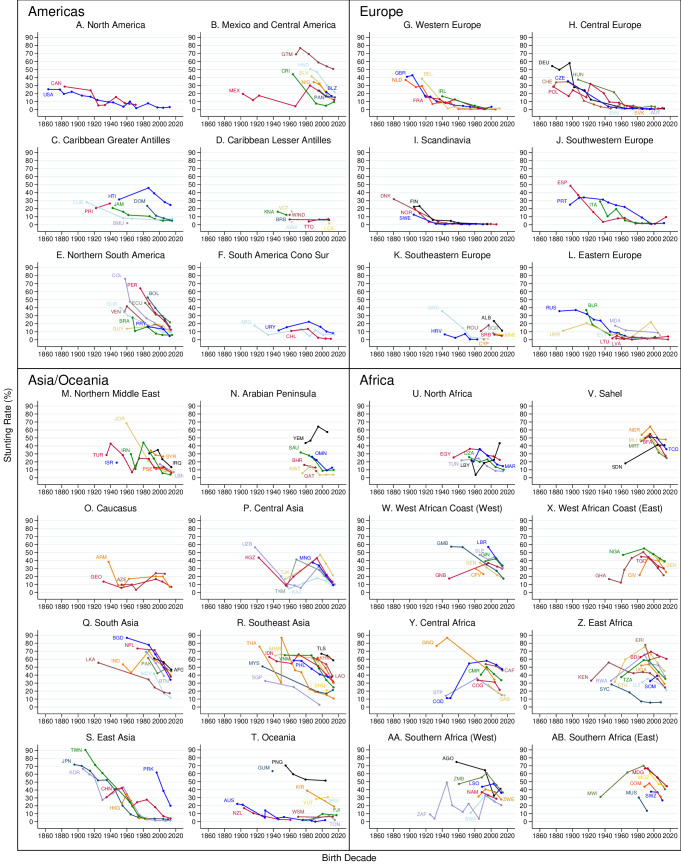
Country-level time series: Americas. The figures plot decadal mean stunting rates for all countries organised by geographic areas. These include countries where historical data were not found; that is, the only stunting data come from the Joint Malnutrition Estimates dataset. Three-letter codes are standard country codes. Sources: Worldwide Historical Stunting Database and UNICEF/WHO/World Bank (2023).

Figure 4 presents stunting rates for countries by birth decade and region from the late 19th century onwards. For reference, when excluding children under age 2, the country with the highest stunting rate in the 2010s birth decade was Burundi (60.4%). The 75th and 90th percentiles of country-level stunting rates in our dataset for the 2010s were Liberia (33.6%) and Angola (41.2%), respectively. India had a stunting rate of 40.6%.

**Figure 5:**
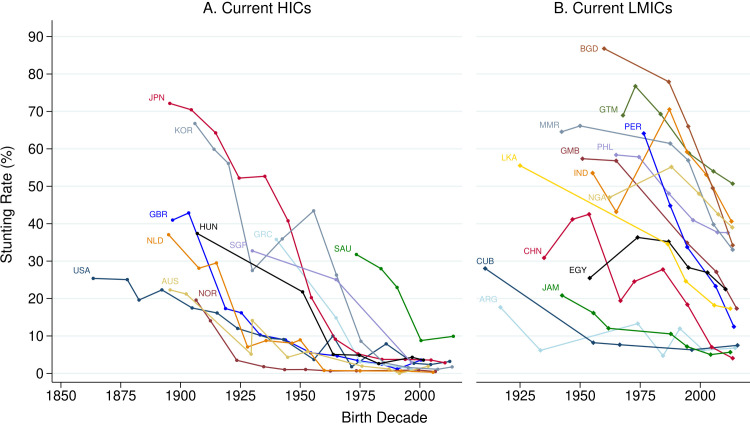
Comparison of historical stunting trends in high-income countries (HICs) and low- and middle-income countries (LMICs). Three-letter codes are standard country codes. The scale of both the x- and y-axes in both panels is the same so that the rate of stunting decline can be compared across the two panels. Sources: Worldwide Historical Stunting Database and UNICEF/WHO/World Bank (2023).

We present data for all countries in figure 4 for completeness (online supplemental appendix N presents world maps), but our analysis focuses on the 25 countries presented in figure 5, 11 HICs (5A) and 14 LMICs (5B). Although reliable historical data are available for a larger number of countries, these countries capture the key findings from the global dataset. Figure 5A shows that HICs had higher stunting rates in the past. While early 20th-century stunting rates were relatively low in Scandinavia and the European settler colonies such as the USA and Australia, stunting rates in Western and Southern Europe were higher, placing them close to the top decile of stunting rates today. Stunting rates were much higher (70% or above) in Japan and South Korea in the early 20th century. Both had fully eradicated stunting by the 1980s, with most of their stunting decline occurring after WWII.

Turning to current LMICs, figure 5B highlights that most countries have experienced a decline in stunting over time, and yet there was considerable heterogeneity in historical stunting rates. Stunting rates were high in mid-20th-century sub-Saharan Africa and South and Southeast Asia, but were much lower in North Africa, the Middle East and the Caribbean. Peru, the Gambia, Thailand and Bangladesh have seen large decreases in the stunting rate since the 1950s, whereas progress in Nigeria and the Philippines has been less dramatic.

With similar stunting rates in the 1950s and 1960s to those observed in South and Southeast Asia today, East Asian countries provide a benchmark for stunting decline. Focussing on Japan, which has reliable, nationally representative data across the 20th century, stunting rates declined at a relatively slow rate of 0.79 percentage points per year before WWII, accelerating to 2.18 percentage points per year post-WWII. Comparing this rate to the stunting decline between the earliest and latest observation in the JME dataset, it is one of the fastest observed. Countries often praised as success stories, such as Nepal, Ethiopia and Peru,^29^ experienced rates of decline of 1.85, 1.2 and 1.14 percentage points per year, respectively.

The results do not support a simple, monotonic view of stunting decline. There were periods of increasing stunting rates, including in Japan and South Korea during WWII: the decadal data in figure 4S hides a 12-percentage-point increase in the stunting rate in Japan (see online supplemental appendix figure O.57). However, other increases in stunting must be viewed with scepticism given the low certainty of evidence of early studies.

## Discussion

Our historical data suggest that eradicating child stunting was a central feature of the health transition. Although we have not estimated a global stunting rate, the global stunting rate was far higher in 1900 than the 47.2% estimated for 1985.^4^ Thus, the global burden of child stunting has reduced dramatically in the past 120 years.

Reductions in child stunting reflect the secular increase in adult stature^5 8^: stunting rates have declined as mean adult stature has increased. However, as a measure of absolute deprivation, stunting rates allow for simpler comparisons of child malnutrition across countries and over time than adult stature. The elimination of child stunting marks the end of child malnutrition, even if mean adult stature continued to increase thereafter.

The striking regional patterns of child stunting in history change the way clinicians and epidemiologists should consider population health and the process of ageing across countries. While there is strong evidence that stunting affects health in childhood and adolescence,^2^ the effect of stunting on adult health via metabolic and cardiovascular disease is more mixed and context-dependent.^30^ Our study provides a new context that has not been considered before. For instance, higher rates of stunting in Japan and Korea during the 1930s and 1940s may mean the ageing process experienced by these cohorts differed from that experienced by members of the same cohorts in other HICs. Likewise, the patterns of ageing in the Caribbean may differ from other LMICs because the region had much lower levels of stunting in the past. Future research should reinterpret existing evidence, considering our findings.

Focusing on case countries with reliable historical data, our study provides new evidence about the causes of child stunting and interventions that have reduced stunting. Comparison across countries suggests there are a range of necessary conditions for reducing stunting, including the wide availability of adequate nutrition and better sanitation. However, these preconditions appear to be insufficient on their own. A historical perspective emphasises that changes in health behaviours are required to eliminate stunting.

The variation in stunting rates among current HICs in the early twentieth century was likely related to urbanisation. Highly urbanised and industrialising countries like the UK, Germany and Belgium had higher stunting rates than more rural countries such as those in Scandinavia and North America, in part because overcrowding and poor sanitary conditions produced a virulent disease environment. Western European countries experienced their stunting decline during the interwar period after achieving adequate levels of nutrition and implementing clean water and sanitation systems, but before widespread adoption of antibiotics and the establishment of public child health services.^7^

The remarkable decline of child stunting in East Asia and parts of Southeast Asia shows that eliminating high stunting rates is possible and that genetic differences in height potential between populations cannot explain stunting rates in LMICs today. Historical high stunting rates in these regions likely stem from small size at birth, followed by growth faltering.^2^ Consequently, increasing size at birth may be an important source of stunting decline^31^: Japan experienced a 250 g increase in mean birth weights between 1900 and 1970^32 33^ and the share of low-birth-weight babies has fallen as stunting declined in many Asian countries since 2000.^34^ Interestingly, birth weight means and distributions in Europe and North America have not changed as dramatically over the past 150 years, potentially explaining why stunting rates above 50% were uncommon in these regions.^35^ Small birth size may be related to gender inequalities in health, especially if maternal nutrition is poor.^36^

While there does seem to be a negative relationship between economic development (gross domestic product (GDP) per capita) and child stunting, the relationship is not straightforward. Stunting rates varied widely at the same level of income. For instance, Taiwan in 1921 had roughly the same GDP per capita (c. 1500 2011$) as Jamaica in 1942,^37^,^39^ but Taiwan’s stunting rate was 74% and Jamaica’s was only 21%. Tracking change in economic growth and child stunting over time also shows that the relationship is complex. Britain, Japan and the Gambia experienced sharp reductions in stunting rates during periods where there was little economic growth, whereas Nigeria and Egypt have experienced relatively little stunting decline despite impressive economic growth in the second half of the 20th century. These mixed findings are consistent with studies that show no effect of GDP growth on stunting since the 1990s.^40^

Japan’s rapid stunting decline after WWII reflected improvements in many broad indicators of development: GDP per capita grew by 8.2% annually in the 1950s, life expectancy increased by 8 years and the diet diversified to include more animal protein.^41^ These factors interacted with and complemented each other, facilitating large improvements in child health. Thus, broad-based development is needed rather than specific interventions targeting just one determinant of stunting, like malnutrition or sanitation.^942^,^44^

Our study also provides suggestive evidence about the successes and failures of public health interventions in reducing stunting rates in the past. A key explanation for stunting is poor water, sanitation and hygiene (WASH) since children without access to clean water and hygienic conditions are far more likely to contract diarrhoeal pathogens, which when chronic, prevent children from absorbing nutrients and slow their growth. Studies of current LMICs, and especially India, show that open defecation is a key cause of stunting,^45^ yet trials of WASH interventions have yielded mixed results.^43^ This mixed picture is present in our historical evidence as well.

In England, major sanitary and water improvement works took place in the second half of the 19th century. These interventions were effective at eliminating mortality from major faecal-oral pathogens: cholera epidemics were eliminated despite their continued presence in other European countries and under-five typhoid mortality rates fell by 88.5%.^46^ However, despite these vast improvements in clean water and sanitation provision, child stunting in the 1890s was above 40%, suggesting that providing clean water and sewerage was not enough to eliminate child stunting. In fact, morbidity and mortality from other faecal-oral diseases, particularly diarrhoea, remained stubbornly high across the 19th century. Diarrhoeal mortality only began to fall after c. 1905^46^ and partly explains the reductions in child stunting in England in the early 20th century.^47^ The causes of declining diarrhoeal morbidity are not settled, but studies point to reductions in urban animal dung and hence fly populations as cars replaced horses for transport,^48^ the diffusion of hygiene knowledge to the working class^49^ as well as safer artificial infant feeding practices.^50^ These factors are mostly related to individual health behaviours, not the provision of public goods.

Although the provision of clean water and sewerage did not eliminate stunting in England, WASH interventions were far more effective in Jamaica. In the early 20th century, open defecation was common in Jamaica. This led to the high prevalence of hookworm (c. 67%), a parasitic worm spread via skin contact with human faeces.^51^ Beginning in 1919, the Rockefeller Foundation launched a decades-long programme to eliminate hookworm from Jamaica. The intervention was multifaceted, including screening for hookworm, drug treatment for those infected, education campaigns to teach people about hookworm and good hygiene behaviour, and the construction of improved, fly-proof latrines for most households. These public health efforts galvanised the Jamaican people into action. The education campaigns took microscopes into the field so that people could see hookworm eggs and larvae for themselves. Rockefeller sources noted that after treatment, children began to grow more quickly and adults felt more energetic and vigorous in their work. The campaign led to widespread changes in sanitary behaviour. Jamaicans built and paid for most of the improved latrines themselves at considerable cost and began to demand better health services from the British colonial government.^51 52^ The campaign led to a 41% decline in infant mortality across Jamaica in the 1920s and 1930s.^52^ Taken together, these factors may explain why the stunting rate was only 21% in the 1940s despite Jamaica’s relative poverty.

These two historical WASH interventions highlight the central role of behaviour change in determining whether improvements in water and sanitation translate into gains in child growth. We cannot directly test the many factors that drive behaviour change in this paper, but we hypothesise that a key contextual difference between these historical cases and LMICs today is the higher level of child mortality in the past. Infant mortality rates (IMR) were 154 deaths per 1000 births in England and Wales in 1900^53^ and 176 in Jamaica in 1920–1922,^51^ far exceeding current levels: the highest IMR in 2023 is 73 in South Sudan, and India’s IMR is 24.^54^ Mortality is a highly salient health outcome that parents readily observe and understand. By contrast, child growth and stunting are more difficult for parents to assess. Children may appear of average height relative to their peers while remaining short relative to a healthy reference population, which parents do not observe directly.^55^

This difference in salience may have influenced health behaviours. In the absence of effective medical treatments, high mortality in the past created strong incentives for households to adopt hygiene practices that reduced exposure to faecal-oral pathogens. In contemporary LMICs, modern medical interventions such as oral rehydration therapy, antibiotics and vaccination programmes have dramatically reduced infant and child mortality,^56^ weakening the mortality incentive for behaviour change without changing many of the determinants of child stunting such as chronic diarrhoeal infections. As a result, WASH interventions that focus primarily on infrastructure provision, such as latrine construction, may have limited effects on stunting unless they also attempt to increase the salience of child growth outcomes and successfully shift hygiene behaviours. This perspective may help explain the persistence of open defecation in settings such as India despite large-scale investments in sanitation infrastructure.^57^

While behaviour change appears critical in relation to WASH interventions, we should not underestimate structural factors such as poverty, low educational attainment, gender-based violence and deep-seated cultural practices that may constrain people’s health behaviours.^57^,^60^ These impediments to behaviour change may vary substantially across different countries and emphasise the importance of context in understanding the causes of child stunting.

The strengths of our study are the rich historical data collected and analysed; the robust methods for estimating stunting rates and assessing the certainty of evidence of each study; and the presentation of historical stunting trends across numerous countries showing different regional patterns.

The main limitation of our study is the varying certainty of evidence among the studies in our systematic review. Although we followed objective inclusion and exclusion criteria strictly, some historical estimates are more reliable than others. Certain trends, especially large increases in stunting in African countries, seem implausible. Early African studies often involved community samples conducted by European researchers in easily accessible locations, possibly leading to inadvertent over-sampling of healthier subgroups (see online supplemental appendix L for examples from the Democratic Republic of Congo and Kenya). Other researchers have shown inverse-U-shaped patterns in female adult stature from 1950 onward in Africa,^61^ but the changes in adult stature were small compared with the swings in child stunting presented here. We include non-nationally representative data to extend the country-level series backwards in time and increase the number of countries covered. While these data may require more careful interpretation than a typical global health dataset (ie, the JME), by carefully considering the certainty of evidence and context, researchers can still make use of these community and regional data. Online supplemental appendix M also replicates figures4  5, excluding community studies and other studies with lower certainty of evidence. Our main results are robust to excluding these studies. In addition, we were not able to assess the representativeness of some studies covering former Soviet countries because the COVID-19 pandemic and the Ukraine war limited access to archival material in Russia (see online supplemental appendix B.3). These limitations highlight the need for more local studies, archival research and historical analysis sensitive to specific contexts to clarify stunting trends.

Another limitation is that our method for computing decade-by-country stunting rates does not account for differences in study characteristics, that is, one decade may be based on urban studies and the next on rural ones, increasing the variability of stunting estimates. Previous studies have used Bayesian Hierarchical Models to produce global trends and CIs in child stunting rates.^4^ We do not follow their procedure or attempt other statistical modelling for several reasons (see online supplemental appendix K.1 for further discussion). First, the data coverage is sparse before the mid-20th century, requiring substantial out-of-sample prediction to estimate global trends. Second, most of our studies are not nationally representative, complicating extrapolation. Third, any modelling exercise would require stable hierarchies or relationships between study characteristics and stunting over time. However, these relationships were not stable: for instance, stunting rate trends vary significantly across Indian states and Kenyan ethnic groups (see online supplemental appendix L). Finally, our study aims to analyse the variation in stunting rates across countries and identify the early levels and speed of stunting decline in under-researched regions, rather than estimating a global stunting rate.

A final limitation is that our focus on historical publications in the systematic review made typical exhaustive search strategies impractical. Search costs were high since most articles had to be physically consulted in libraries and abstracts for many articles were not indexed in Google Scholar or PubMed. Thus, we focused on forward and backward reference tracing from existing studies. While our procedure may mean that we missed some studies that were not cited in later work, these studies are likely to be small, community studies rather than large, nationally-representative studies that could substantially improve our estimates.

Our novel historical data on child stunting reveal that the factors behind stunting decline vary across countries, encompassing urbanisation, disease environments, public health interventions, socioeconomic development and change in health behaviours. This study underscores the importance of context-sensitive approaches to addressing child stunting today.

## Supplementary material

10.1136/bmjgh-2024-018607 online supplemental file 1

10.1136/bmjgh-2024-018607 online supplemental file 2

10.1136/bmjgh-2024-018607 online supplemental file 3

## Data Availability

All data relevant to the study are included in the article or uploaded as supplementary information.
