# Supplementary material for: The decline of child stunting in 122 countries: a systematic review of child growth studies since the 19th century
Source: BMJ Glob Health. 2026 Feb 18;11(2):e018607. doi: 10.1136/bmjgh-2024-018607 (PMC12918666; doi:10.1136/bmjgh-2024-018607)
Supplement: online supplemental file 1 [file bmjgh-11-2-s001.docx]

### BMJ Global Health Author Reflexivity Statement

Adapted from Morton, B., Vercueil, A., Masekela, R., Heinz, E., Reimer, L., Saleh, S., Kalinga, C., Seekles, M., Biccard, B., Chakaya, J., Abimbola, S., Obasi, A. and Oriyo, N. (2022), Consensus statement on measures to promote equitable authorship in the publication of research from international partnerships. Anaesthesia, 77: 264-276. <https://doi.org/10.1111/anae.15597>

*Only 5 of the 43 authors on this paper are based in LMICs (two from Colombia, and one each from South Africa, the Philippines, and Argentina). We recognise that this reflects an imbalance in representation typical of large global historical datasets and systematic reviews led and funded from HIC institutions. The LMIC authors involved are established academics who contributed substantive country-specific expertise to the project. However, we acknowledge that their limited number and the absence of dedicated funding for LMIC-based collaboration constrained opportunities for more equitable leadership and capacity-building. The project was conceived and the collaboration assembled before publication of the 2022 consensus statement that informed this reflexivity framework, which limited the extent to which those recommendations could be prospectively implemented.

| **Study conceptualisation** | |
| --- | --- |
| 1. How does this study address local research and policy priorities? | The research aimed to identify global historical patterns rather than address specific local policy priorities. While this limits immediate local applicability, we anticipate that the long-term country-level perspective on child stunting may still be of value to researchers and policymakers in LMICs seeking historical context for contemporary nutrition challenges. |
| 1. How were local researchers involved in study design? | Juliana Jaramillo-Echeverri (based in Colombia) was involved in study design from an early stage, contributing to framing of the research questions and analytical approach. |
| **Research management** | |
| 1. How has funding been used to support the local research team(s)? | The project was completed with limited funding (approximately £10,000), most of which supported research assistance based at the corresponding author’s institution. No dedicated funds were available to support LMIC-based researchers, which constrained opportunities for compensated time, travel, or additional collaboration. |
| **Data acquisition and analysis** | |
| 1. How are research staff who conducted data collection acknowledged? | Everyone who collected data for the project is a co-author on the paper. |
| 1. How have members of the research partnership been provided with access to study data? | All authors have had access to study data and to the studies collected as part of our systematic review. |
| 1. How were data used to develop analytical skills within the partnership? | The data produced was shared with all authors so that they could explore the data and discuss results. |
| **Data interpretation** | |
| 1. How have research partners collaborated in interpreting study data? | Research partners shared their interpretations of country-specific data with the corresponding author, primarily through written correspondence. We acknowledge that this mode of engagement reflects a centralised analytic process and may have limited opportunities for collective, real-time interpretation. |
| **Drafting and revising for intellectual content** | |
| 1. How were research partners supported to develop writing skills? | All authors were invited to comment on multiple drafts of the manuscript. Juliana Jaramillo-Echeverri played a particularly important role in revising and strengthening the paper. |
| 1. How will research products be shared to address local needs? | The study-level stunting estimates generated as part of our systematic review will be published in an open-access repository following publication so that the data will be openly available to all researchers. The article will also be published open access so that LMIC researchers can access it. |
| **Authorship** | |
| 1. How is the leadership, contribution and ownership of this work by LMIC researchers recognised within the authorship? | Leadership and contributions are described transparently in the authorship statement. We recognise, however, that leadership roles were predominantly held by HIC-based researchers, reflecting where funding, coordination, and primary analytic responsibility were located. |
| 1. How have early career researchers across the partnership been included within the authorship team? | Early career researchers are also included as authors including the research assistants who worked for the corresponding author in London. |
| 1. How has gender balance been addressed within the authorship? | We aimed for gender balance where possible, though this was secondary to identifying country-specific expertise, which may have perpetuated existing gender imbalances in the field. |
| **Training** | |
| 1. How has the project contributed to training of LMIC researchers? | Participation in the project provided LMIC researchers with experience contributing to a large global systematic review, including study identification and data collation. However, we acknowledge that structured training or mentorship activities were limited. |
| **Infrastructure** | |
| 1. How has the project contributed to improvements in local infrastructure? | The project has not contributed to local infrastructure. Again, there were very limited funds, so it was not possible to contribute in this way. We recognise that the absence of infrastructure investment reflects broader inequities in how global health research is funded and organised. |
| **Governance** | |
| 1. What safeguarding procedures were used to protect local study participants and researchers? | Although no primary data collection or human participants were involved, we sought to foster an inclusive and respectful collaboration by ensuring equal access to communication and decision-making processes. |
