# Supplementary material for: The decline of child stunting in 122 countries: a systematic review of child growth studies since the 19th century
Source: BMJ Glob Health. 2026 Feb 18;11(2):e018607. doi: 10.1136/bmjgh-2024-018607 (PMC12918666; doi:10.1136/bmjgh-2024-018607)
Supplement: online supplemental file 3 [file bmjgh-11-2-s003.pdf]

Web Appendix

The Decline of Child Stunting in 122 Countries: A Systematic Review of Child Growth Studies Since the Nineteenth Century

Eric B. Schneider,[*] Juliana Jaramillo-Echeverri,[†] et al. (see below)

22 December 2025

Data Contributors (co-authors): Matthew Purcell, Brian A'Hearn, Vellore Arthi, Matthias Blum, Elizabeth Brainerd, Joseph Capuno, Alexandra L. Cermeño, Amílcar Challú, Young-Jun Cho, Tim Cole, Jose Corpuz, Ewout Depauw, Federico Droller, Dieter von Fintel, Joël Floris, Gregori Galofré-Vilà, Bernard Harris, Tim Hatton, Laurent Heyberger, Tuuli Hurme, Kris Inwood, Hannaliis Jaadla, Jan Kok, Michał Kopczyński, Samuel Lordemus, Brian Marein, Adolfo Meisel-Roca, Stephen Morgan, Stefan Öberg, Kota Ogasawara, José Antonio Ortega, Nuno Palma, Anastasios Papadimitriou, Renato Pistola, Björn Quanjer, Helena Rother, Sakari Saaritsa, Ricardo Salvatore, Kaspar Staub, Pierre van der Eng and Evan Roberts

[*]London School of Economics, e.b.schneider@lse.ac.uk, https://www.ericbschneider.com/
[†]Banco de la República, Colombia, juliana.jaramilloe@gmail.com, https://j-jaramillo-echeverri.com/

Contents

A Search Strategy 5

A.1 Search Google Scholar and PubMed: 5

A.2 Trace Citations from Known Studies 6

A.3 Complete Search through Forward and Backward Citation Linkage 7

A.4 Ensuring Complete Search Coverage 7

A.5 Limitations to Search Coverage 9

B Inclusion and Exclusion Criteria 11

B.1 Inclusion Criteria and Justification 11

B.1.1 Inclusion criterion 1: Mean heights available 11

B.1.2 Inclusion criterion 2: Children under age 10.99 12

B.1.3 Inclusion criterion 3: Data reported in tables 16

B.1.4 Inclusion criterion 4: Sexes reported separately 17

B.1.5 Inclusion criterion 5: Large sample size at each age 17

B.1.6 Inclusion criterion 6: Study reports the children's birth year . . . 18

B.2 Exclusion Criteria and Justification 18

B.2.1 Exclusion Criteria 1: Exclude non-representative studies 18

B.2.2 Exclusion Criteria 2: Exclude children under age two 20

B.3 USSR Data 21

C Transcription Protocols and Example Studies 25

C.1 Forms 26

C.1.1 Form 1: Frequency Distribution Form 26

C.1.2 Form 2: Percentile Form 27

C.1.3 Form 3: Mean and Standard Deviation Form 29

C.1.4 Form 4: Individual-Level Data Form 29

C.2 Study-level Survey on Data Quality and the Inclusion and Exclusion Criteria 29

D Data Cleaning and Verification 34

D.1 Cross-validation Checks of the Data 34
D.2 Verification of Representativeness 35

E Healy Variance Adjustment 36

F Dealing with Outliers 38

G Computing Study-Level Stunting Rates 40
G.1 Computing Study-Level Stunting Rates from Means and Standard Deviations 40
G.2 Computing Study-Level Stunting Rates from Mean-only Data 41

H The Unicef/WHO/World Bank Joint Malnutrition Estimates 46
H.1 Background of the JME Dataset 46
H.2 Further Adjustments to the JME Dataset 47

I Assessing Certainty of Evidence 51
I.1 Adapting the GRADE System to Assess the Certainty of Evidence 51
I.2 Certainty of Evidence in our Meta-analysis 55

J Data Sources and Stunting Estimates 59
J.1 Variable Definitions 59

K Computing Country by Birth Decade Stunting Rates 61
K.1 Problems with Aggregating Studies with Disparate Methodologies and Sampling Procedures 61
K.2 Our Method for Aggregating Studies 63

L Assessing Sources of Error in the Studies 67
L.1 Democratic Republic of Congo 68
L.2 Kenya 73
L.3 India 77
L.3.1 Indian Council of Medical Research Study 79
L.3.2 Stunting Trends Across States 87

L.4 South Africa 90

M Replication of Main Results Excluding Studies with Low Certainty of Evidence 96

N World Maps of Stunting Rates over Time 102

O Country Study-Level Stunting Rate Graphs 106

P References 229

A Search Strategy

Our search strategy deviated from standard meta-analysis procedures because the target studies of our search often appeared in journals that had not been digitised and some were not indexed in Google Scholar or PubMed. Search costs for these studies were very high since they had to be physically consulted in libraries or requested via interlibrary loan. Thus, while we often began our searches with these modern databases, most of our studies were found by tracing references forward and backward from known studies. This increased the efficiency of our searches since we could be more confident that a study contained useable data from a historical period than from simply seeing a title on a bibliographic index. The following sections explain our search strategy and show that it is unlikely to have produced biased results.

A.1 Search Google Scholar and PubMed:

We began our search for studies about a particular country by searching Google Scholar and PubMed for studies using the country name, child and the following keywords: growth, height and stunting. The focus of our meta-analysis was to expand the existing Joint Malnutrition Estimate (JME) database (Unicef et al., 2023) into the past wherever possible rather than to collect all studies of child growth conducted for each country.[1] Thus, in this initial search, we looked for three types of articles that would help us find historical studies, i.e. studies mainly before 1990.

1. *Recent Growth References*: Recent growth references were often helpful because they would cite earlier growth references created for each country and potentially other neighbouring countries, allowing us to find high quality studies conducted in the past.

2. *Literature on the Secular Increase in Stature*: There were a number of studies by physical anthropologists, human biologists and economic and anthropometric historians that collated available information on child growth from earlier studies in

[1] The JME dataset is discussed at length in Appendix H.

order to illustrate changes in child growth over time. Some examples of these types of studies would be Tanner (1981), Cameron (1979), Rosenbaum (1988), Meredith (1964, 1976), Steckel (1987), and Papadimitriou et al. (2002). We then traced the references in these studies to find the original report of the data wherever possible.

3. *Studies conducted before the JME stunting estimates*: Where we found studies for earlier periods before the JME, we searched for these studies and traced their citations forward and backward.

A.2 Trace Citations from Known Studies

In parallel to the keyword search strategy discussed above, we also systematically traced references forward and backward from a number of earlier international meta-analyses either conducted historically or of historical child growth studies. These provided a large number of studies across the nineteenth and twentieth centuries that could be found and then traced forward or backward. We used the following studies as a starting point in the search, and in each case systematically searched for all studies that appeared to contain quantitative information on child growth:

1. Burk (1898) provides a review of research conducted before 1898 in a large number of North American and European countries.

2. Baldwin (1921) provides a meta-analysis of anthropometric research before 1920, including an annotated bibliography of 911 studies covering countries all over the world.

3. Eveleth and Tanner (1976, 1991) found a very wide range of published studies of growth curves from around the world from the 1960s and early 1970s in the first edition and the 1980s in the second edition. We systematically searched for all of the studies that they cite and provide data for in their books.

4. Hermanussen et al. (2018, 2016) provide lists of studies of historical child growth, and we systematically traced their citations as well.

A.3 Complete Search through Forward and Backward Citation Linkage

Using the initial studies found following the procedure above, we searched for all studies on child growth cited in these studies and also searched for articles citing these studies in Google Scholar and PubMed (forward citation tracing) where possible. This yielded a large body of articles for which to search. These studies were systematically found and assessed in relation to the inclusion criteria (discussed below). For instance, using the search methods discussed above, we found 18 pre-JME studies discussing child growth in Nigeria. We found PDFs online for seven of the articles and the other eleven were consulted in the British Library and Wellcome Library. Only three of the studies met our inclusion criteria.

A.4 Ensuring Complete Search Coverage

To ensure completeness in our search, we recruited a team of 43 data contributors. These data contributors were experts on child growth or anthropometric history for their particular country or set of countries and in total covered 34 countries around the world. Wherever possible we chose experts who were also physically resident in the country they were studying so that they could search for additional studies in their national libraries, which were more likely to contain older historical studies than libraries elsewhere. These experts also provided language expertise in 17 languages: English, French, Spanish, Portuguese, Italian, Greek, Dutch, German, Swedish, Finnish, Polish, Estonian, Russian, Indonesian, Mandarin, Korean and Japanese. While these languages are not exhaustive, they include the languages of all the major colonial powers in the nineteenth and twentieth centuries, giving us access to research produced in these languages on colonies in historical periods. This research was particularly important in South Asia, Southeast Asia and Africa.

The data contributors focused on their own countries but also worked collaboratively using Slack so that if they found reference to a study on a country outside of their remit,

Table A.1: List of Data Contributors

Country	Data Contributors
Europe	
Belgium	Ewout Depauw
Estonia	Hannaliis Jaadla
Finland	Tuuli Hurme, Sakari Saaritsa
France	Laurent Heyberger
Germany	Matthias Blum, Helena Rother
Greece	Anastasios Papadimitriou
Italy	Brian A'Hearn, Juliana Jaramillo-Echeverri
Netherlands	Jan Kok, Björn Quanjer
Poland	Michał Kopczyński
Portugal	Alexandra Cermeño, Nuno Palma, Renato Pistola
Russia/USSR	Elizabeth Brainerd
Spain	Gregori Galofré-Vilà
Sweden	Stefan Öberg, Eric Schneider
Switzerland	Joël Floris, Kaspar Staub
UK	Vellore Arthi, Bernard Harris, Tim Hatton, Eric Schneider
North America	
Canada	Kris Inwood, Matthew Purcell
USA	Evan Roberts
Latin America	
Argentina	Ricardo Salvatore
Chile	Federico Droller
Cuba	Brian Marein
Colombia	Juliana Jaramillo Echeverri, Adolfo Meisel-Roca
Mexico	Amilcar Challu
Puerto Rico	Brian Marein
Africa	
Equatorial Guinea	Jose Antonio Ortega
South Africa	Dieter von Fintel
Asia	
China, Hong Kong	Stephen Morgan
Indonesia	Pierre van der Eng
Japan	Kota Ogasawara
Philippines	Joseph Capuno, Jose Corpuz, Samuel Lordemus
South Korea	Young-Jun Cho
Taiwan	Stephen Morgan
Australia/Pacific Islands	
Australia	Kris Inwood, Matthew Purcell
New Zealand	Kris Inwood, Matthew Purcell, Evan Roberts

they could recommend it to the data collection team for that particular country. This cross-referencing was helpful in many cases.

In order to ensure coverage beyond the 34 countries with expert data contributors, the PI (Eric Schneider) and his team of research assistants (Juliana Jaramillo-Echeverri, Mathew Purcell and Helena Rother) searched for studies for all other countries in the world, finding data for another 88 countries. Conducting this work from London was crucial for the team, as they had easy access to the British Library, the largest library in the world, the library of the School of African and Oriental Studies (SOAS), and the Wellcome Library, which specialises in the history of medicine and contains many old print periodicals. This allowed the team to find articles in country and region-specific journals that would not be a part of even an excellent university library. All data contributors also made extensive use of interlibary loans and online repositories such as JSTOR, Hathi Trust and archive.org to access studies. These varied resources meant that there were very few studies that we tried to find and could not: an estimate based on the search for African studies was less than 5%. The PI and his team also cross-checked the studies provided by data contributors with the studies listed in Section A.2 above to ensure good coverage.

A.5 Limitations to Search Coverage

Despite our best efforts, there are still some limitations in the coverage of studies. Although our team included experts in 17 languages, there were still a handful of articles in languages that we could not cover. In some cases, we used Google Translate to translate parts of the articles, but this was not possible for languages that did not use Latin script since we were not able to type the (non-digitised) text into Google Translate. Not only could we not incorporate these studies, but it was also very difficult to trace their references. However, given the importance colonialism and its influence on scholarly work on Africa, South Asia, and Southeast Asia, there were relatively few articles in languages that we could not incorporate. We also recruited experts to cover countries in East Asia whose languages would be most difficult for us to grapple with. We were also limited by the exclusion from many older, country- or region-specific medical journals from PubMed

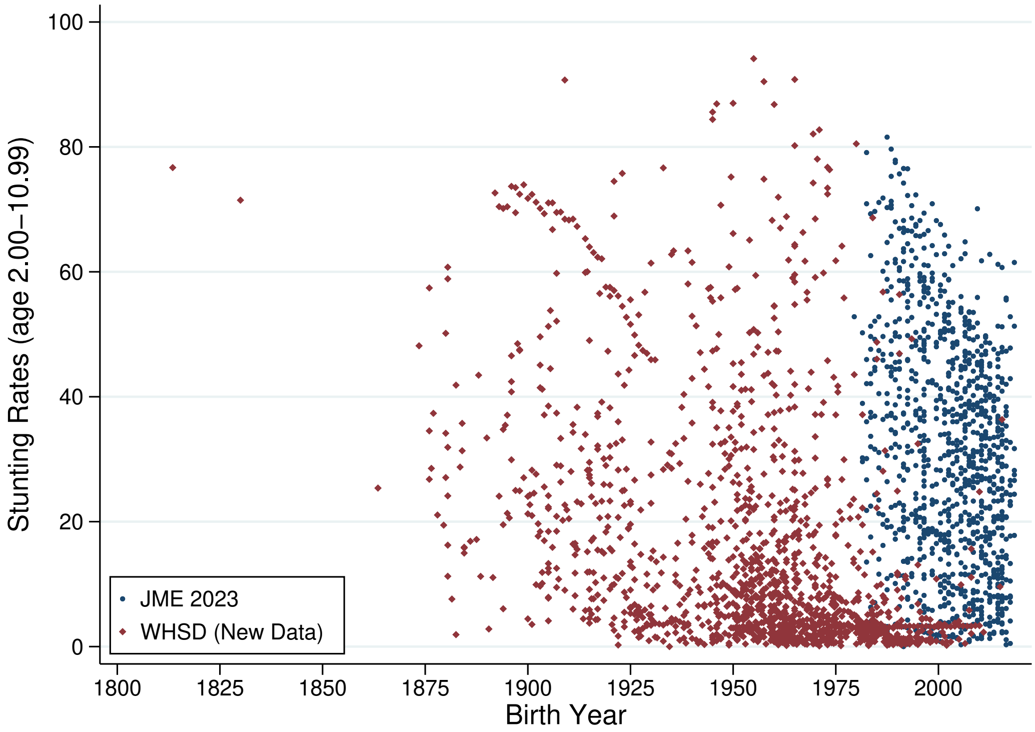

Figure A.1: Stunting Rates in the Final Analytical Sample by Provenance

Notes: JME is the Joint Malnutrition Estimates produced by Unicef, the WHO and the World Bank. *Sources*: Worldwide Historical Stunting Database and UNICEF/WHO/World Bank (2023).

and Google Scholar. This limited our ability to trace references forward and to discover articles in the first place.

Overall, however, we believe that the search coverage is fairly exhaustive and unlikely to contain systematic biases. With such high search costs, we had to balance a truly exhaustive search with researchers' time in consulting physical copies of old journals (mostly) in libraries. While our procedure may mean that we are missing some studies that were not cited in later work, these studies are likely to be small, community studies rather than large, nationally representative studies that could substantially improve our estimates.

Figure A.1 presents the new stunting estimates that we found (maroon) in relation to the JME data that came before showing just how much our systematic review has contributed to understandings of child stunting before the 1990s.

B Inclusion and Exclusion Criteria

B.1 Inclusion Criteria and Justification

When searching for studies, we required the following six inclusion criteria to be met for the study to be officially recorded and have its data transcribed:

1. Study contains mean heights or information from which mean height can be computed
2. Study contains data for children under age 10.99
3. Study reports data numerically in tables
4. Study reports height separately by sex
5. Sample size for each one-year age group by sex cell is greater than 20
6. Study reports the children's birth year

While conducting the search, data contributors were also careful to exclude multiple reports of the same underlying study.

In the sections below, we carefully explain and justify these inclusion criteria. In addition, we consider whether adhering to these inclusion criteria produce bias to the meta-analysis or estimated stunting rates for each study.

B.1.1 Inclusion criterion 1: Mean heights available

The most basic information necessary to compute a stunting rate is mean height by age, and therefore mean height or information from which mean height by age could be computed was a key inclusion criteria. In the vast majority of studies, mean height was reported. In others, we computed the mean height from a frequency distribution or from height percentiles (see Section C.1.2 for more detail). However, we were unable to include a number of studies that did not report descriptive statistics of height. After the introduction of the first WHO growth reference (Jelliffe, 1966), it became more common for

studies to report stunting rates relative to this reference and not report basic descriptive statistics of height by age. Unfortunately, the vast majority of these studies could not be included because it was not possible to translate these shares into estimates of mean and standard deviation of height at each age necessary to compute stunting rates relative to the 2006/7 WHO standard/reference.[2]

B.1.2 Inclusion criterion 2: Children under age 10.99

Child stunting is normally only computed for children under the age of five, making comparisons with the 2006 WHO growth standard, which covers children age 0 to 5, relatively simple. However, studies of the heights of young children under five are much less common in the past, especially in the late nineteenth and early twentieth centuries. The relatively paucity of early childhood studies likely reflects the difficulties in measuring children's heights before they can stand, and the ease with which children can be measured in schools. Figure B.2 presents the number of mean height observations for both sexes by one-year age-group and birth decade. Clearly, only conducting the meta-analysis on children age five and younger would dramatically reduce the potential stunting observations. This also would affect the number of studies and countries covered and how far back in time we can estimate a stunting rate. If we restricted our data to observations of children under age five, we would lose 564 studies from a total of 923 studies and 35 countries from a total of 122 countries, but these figures are even more striking for the period before 1950. Restricting our analysis to studies including children under age five before 1950 would result in the loss of 265 studies from a total of 340 studies and the loss of 33 countries from a total of 64 countries. Given the potential to drastically expand the coverage of our meta-analysis, it seemed prudent to look at children above the age of five as well.

[2] In order to compute the standard deviation of the distribution, one needs two percentiles of the distribution (see Equation 1 below). This kind of information was sometimes available, i.e. the authors reported the share of children below -2 and below -1 Z-scores relative to the old WHO standard, but in most of these cases, age groups or sexes were combined meaning that we still could not convert the measures into the 2006/7 WHO standard/reference. Yang and de Onis (2008) developed an algorithm for converting stunting estimates from the 1977 NCHS/WHO reference to the 2006 WHO growth standard, but this algorithm requires data for children ages 0 to 5 to work effectively and does not allow for children under age two to be excluded as is necessary for reasons of comparability (see Appendix B.2 below).

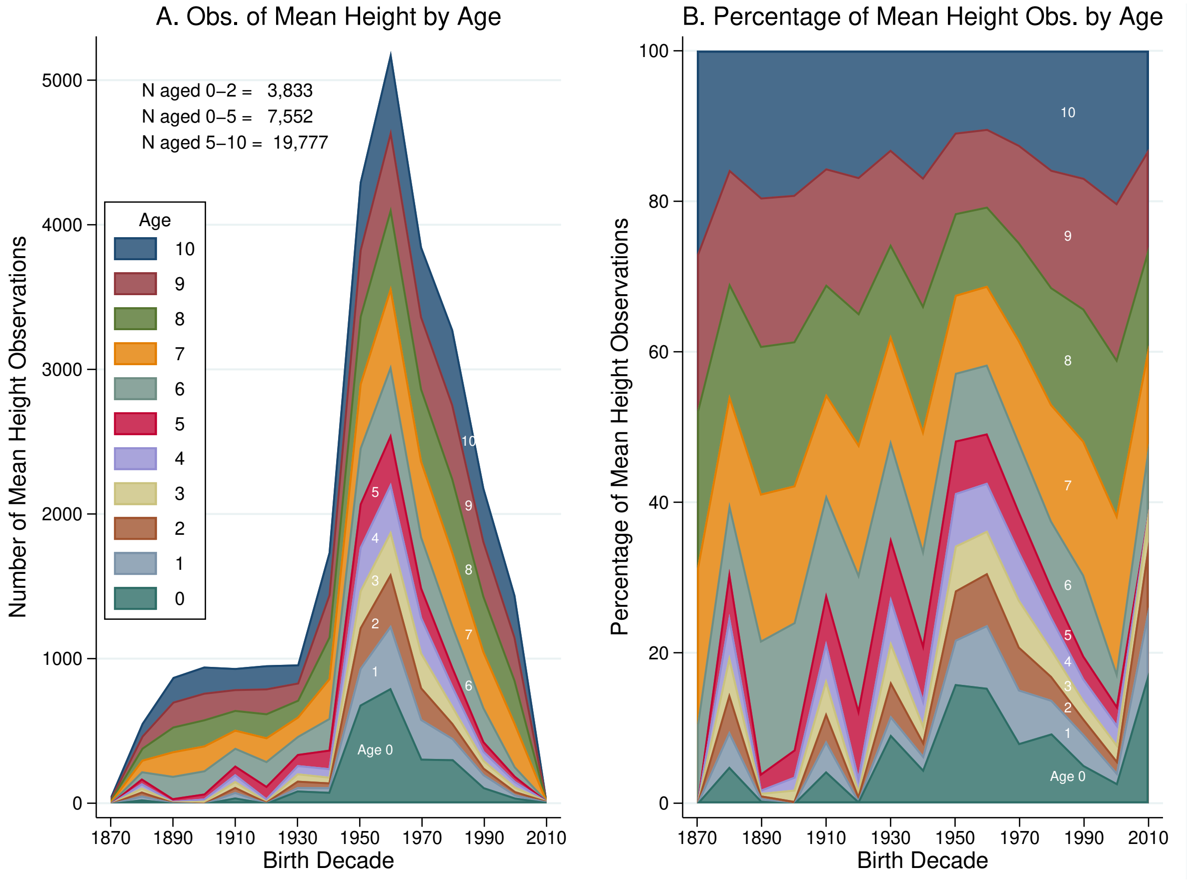

Figure B.2: Mean Height Observations of Both Sexes by Birth Decade and Age

Notes: Age groups are rounded down ages, i.e. age 1 includes children aged 1.00 to 1.99.

Sources: Worldwide Historical Stunting Database.

The question then is to what extent does including children above the age of five bias our estimates of the stunting rate. There are several issues at play here. First, by accepting children above the age of five, we are forced to use both the 2006 WHO growth standard for children 5 and younger and the 2007 growth reference, which covers school-aged children. The 2006 standard was the product of a multi-year study of child growth in six countries around the world, and therefore is taken to be reflective of how healthy children should grow irrespective of genetic differences in height (de Onis et al., 2004, 2012; WHO, 2006). However, conducting a similar study for school-aged children was deemed infeasible, so the 2007 WHO growth reference was based on the NCHS study of school-age child growth in the United States that was conducted in the mid-1970s with some adjustments to make it compatible with the 2006 standard and to adjust for the beginnings of the child obesity crisis, which was beginning to emerge at that time (de Onis et al., 2007). This means that the reference cannot be taken as the normative standard of growth in the same way that the 2006 standard can.

Using the 2007 reference is particularly difficult once children begin experiencing their pubertal growth spurts. There is considerable variation in the timing of the pubertal growth spurt across historical and modern populations (Eveleth and Tanner, 1991; Gao and Schneider, 2021; Schneider, 2024; Steckel, 1987), so if a population experiences the pubertal growth spurt earlier or later than the reference population, this will result in systematic distortions in the Z-scores that do not reflect the population's nutritional status. For this reason, we only use studies for children under age ten, who are very unlikely to have started their pubertal growth spurts, especially in history when the pubertal growth spurt occurred at later ages than it does today (Schneider et al., 2021).

The question then is whether the reference creates any systematic biases from exact ages 5.00 to 10.99 when we are employing it in our study. Fortunately, when creating the new 2007 growth reference, the authors input the data for earlier ages used to create the 2006 standard, which means that 2006 standard flows smoothly into the 2007 reference across age five, preventing sharp jumps in the Z-scores that had been present in the earlier WHO reference (de Onis et al., 2007; Jelliffe, 1966). While the 2007 reference is not as

robustly constructed at the 2006 standard, there is no reason to believe that it cannot serve as a useful and comparable benchmark for stunting above age five. We also note that the 2007 reference is commonly used to compare school-aged children's heights in the literature (Lundeen et al., 2014; Prentice et al., 2013).

In addition to the reference or standard with which to compare children's growth, another issue with using children above the age of five is that they could have suffered further growth faltering (falling behind the standard/reference) or experienced catch-up growth (improving their position relative to the standard/reference) from their condition in early childhood, which would then complicate comparisons between older and younger children. We will discuss growth faltering and then catch-up growth. There is a clear pattern of growth faltering by age in modern LMICs (Victora et al., 2010). Children in nearly all contexts start their lives in a relatively healthy position compared to the standard and then fall behind the standard in the first two years of life. From ages two to five, the mean height-for-age Z-score and stunting rate are reasonably stable. Growth faltering has not been studied as extensively after age five, but most scholars seem to agree that the children's position remains more or less stable (Benjamin-Chung et al., 2023).

There is greater controversy surrounding the possibility of catch-up growth at a population level: children experiencing faster growth than the standard/reference because of improved health conditions. There has long been a consensus among nutritionists and development economists that once children experience child stunting, it is very difficult for them to recover (Alderman et al., 2006; Golden, 1994; Victora et al., 2010; Wells, 2017). However, the evidence for this position has weakened considerably in recent years. Prentice et al. (2013) showed that some African populations did experience catch-up growth, suggesting that catch-up was possible. Prentice et al. (2013)'s findings have been challenged by showing that the height gap between LMIC children and the reference may be increasing even as Z-scores are improving since the variance of height increases with age (Leroy et al., 2015; Lundeen et al., 2014). Thus, it is not clear whether this is 'real' catch-up growth or a product of the reference. However, there are a number of

examples from history that show catch-up growth is possible at the population level. Steckel (1986) showed that enslaved people in the antebellum US South experienced very dramatic catch-up growth (see also Schneider 2017). Schneider et al. (2021) also showed that Japanese children who were born and experienced early childhood during the food shortages of the Second World War experienced remarkable catch-up growth once health conditions improved after the war.

However, although catch-up growth does appear to be possible, it likely to be somewhat rare rather than a typical part of the growth pattern. It occurs as a recovery from significant health shocks or a response to clear improvements in health conditions. Likewise, further growth faltering between the ages of two and ten is possible in response to a health shock, but most commonly this is stable at the population level. This means that there shouldn't be systematic differences between stunting rates computed from a sample of 4-5 year-olds and stunting rates computed from a sample of 7-8 year-olds.

Growth faltering in the first two years of life, on the other hand, appears to be a typical part of the growth pattern for most populations with poor health conditions whether in the past or the present (Roberts and Warren, 2017; Schneider, 2024). Thus, the age composition of children in a sample matters for the stunting rate: i.e. it is not straightforward to compare a stunting rate computed from a sample of 0-2 year-olds to a stunting rate computed from a sample of 4-5 or 7-8 year-olds. Alderman and Headey (2018) even show that the determinants of undernutrition vary when looking at children 0-2 and 2-5 years old. We discuss how we deal with these age related issues in Appendix B.2.

B.1.3 Inclusion criterion 3: Data reported in tables

We also required that the actual values of height be recorded in tables. Many studies presented growth curves of children in graphical form but failed to report the numerical values. We experimented with using standard software to convert figures into tables, but the results were highly erratic often giving implausible values for the stunting rate. The measurement error in the graphical data likely occurs because most of the graphs were hand-drawn as was common before graphical software and printers made highly precise

graphs commonplace. Rather than cherry-picking the graphical conversions that seemed plausible, we decided to exclude this type of data from our analysis. The only exception was where height by age was plotted against both vertical and horizontal grid lines, which enabled us to estimate quite carefully what the height value was at each age. This exception only applied in a handful of studies.

B.1.4 Inclusion criterion 4: Sexes reported separately

It was also necessary for the study to report the height information for the sexes separately. The WHO 2006 standard and 2007 reference are computed separately for boys and girls. While there are only relatively small differences between the standard/reference between boys and girls for children aged 10 and under, creating a combined reference for both sexes is not straightforward, nor would it allow to us make straightforward comparisons with datasets that reported heights separately for the sexes. In most cases, studies that reported boys and girls together also tended to be small community studies, so not including these studies did not result in the loss of studies with a high certainty of evidence.

B.1.5 Inclusion criterion 5: Large sample size at each age

We also required that there be at least 20 individuals measured in each one-year by sex age bin. This ensured that the sample mean for each age was an accurate reflection of the population and that the sample was large enough to get a fairly reasonable estimate of the standard deviation. This 20 individual minimum is still on the low side, but the median sample size in each cell (where reported) was 147, clearly a large enough sample to robustly estimate the mean and standard deviation. We also included studies where the number of individuals was not reported at each age, but the total sample size was reported and was large enough for the sample size at each age to meet this inclusion criterion. There were perhaps a few age groupings that may have had a smaller sample size, but we felt that on balance including these studies was worth the potentially increased measurement error.

B.1.6 Inclusion criterion 6: Study reports the children's birth year

In order to compare studies over time, we also needed some degree of certainty about the average birth year for children in the study. For most studies, this was relatively easy to determine: the study was conducted over a few months within one year and so the average birth year was simply the study year minus the average age. However, other studies were less clear about when the measurements were taken and a few were excluded because we could not place the average birth year within a decade.

B.2 Exclusion Criteria and Justification

We used the search strategy and inclusion criteria to find a full set of studies that could help us reconstruct child stunting rates around the world into the past. Beyond these, however, we did enforce two exclusion criteria in relation to this particular meta-analysis. We excluded studies and data based on the following criteria:

1. We excluded studies where there was evidence of direct selection on socioeconomic status or health into the sample of children measured.
2. We excluded at times studies and at other times some data within a study where the measurements were for children below the age of two.

We discuss the justification for and implementation of these two exclusion criteria in the subsections below. These two criteria were exclusion rather than inclusion criteria because we wanted to have access to data for specific groups (such as working-class populations) for more fine-grained historical analysis, but these are difficult to incorporate into a meta-analysis of this type.

B.2.1 Exclusion Criteria 1: Exclude non-representative studies

First, we excluded studies where there was evidence of direct selection on socioeconomic status or health. While many studies were designed to be representative of the population being examined, others had an explicit focus on sub-sections of the population such as the

working classes or upper classes. For instance, some studies (including some by economic historians today) focussed on the working classes in order to understand the health of working class populations changed over time (c.f. Schneider 2016). Other studies explicitly targeted upper class populations in order to determine how healthy children should grow, not dissimilar to what the WHO MGRS study did when constructing the child growth standard (de Onis et al., 2004). In other studies, the selection was not as clear cut. For instance, the sample may be drawn from children in school, and if poorer and less healthy individuals were less likely to be at school, this would lead to an underestimate of the stunting rate. Schneider (2020) shows that there is selection of healthy, tall individuals into secondary schools in Japan and likely also in Boston in the 1860s. Fortunately, children age 10 and under tended to be in primary school, and primary school enrolment was universal at an earlier date than secondary school enrolment. Where we only have information on one class, it is very difficult to extrapolate from the stunting rate of that class to the stunting rate of the population. This means that we must exclude these studies, even if they would be very relevant and useful in a more historical study of a particular country or region.

We also excluded studies where there was evidence that malnourished or unhealthy children were excluded from the sample. Some studies had selection criteria for individuals to be included in the sample such as satisfactory weight gain up to that point or birth weights above a certain threshold. This again results in selection bias that is very difficult to counter, so there is no way to include these studies in the wider meta-analysis.

Note that our definition of representativeness here is very specific. We hold studies to be representative if the sample of children studied is representative of the targeted population. For instance, Bowditch (1877) is taken to be representative because Bowditch's intention was to measure child growth in Boston school children and based on the discussion of the sampling procedure in his report, it seems reasonable that his sample was representative of children in Boston. There is no evidence of selection on socioeconomic characteristics or on children's health status, at least for children aged 10 and under (Schneider, 2020). However, these children may not have been representative of children

in the United States more broadly since Boston was an urban industrial centre and did not include significant shares of black people as existed in the South.

B.2.2 Exclusion Criteria 2: Exclude children under age two

Secondly, we exclude data (and hence some studies) for children aged under two. As mentioned above, there is a well-known pattern of growth faltering, slower than normal growth, during the first two years of life among stunted children (Victora et al., 2010). This means that a cohort of children will be mechanically less stunted at ages 0-1 than that same cohort would be at ages 4-5. Alderman and Headey (2018) show that this process of growth faltering affects the socioeconomic factors associated with child stunting. It also means that the age composition of samples will affect the stunting rate (Aiyar and Cummins, 2021). This is particularly important in the context of our meta-analysis because many of our studies only include children above the age of two.

There are two ways of overcoming this issue. One is to model the effect of age on the stunting rate to adjust for the age composition of each study. However, this is very difficult to implement in practice because we would not expect the relationship between age and child stunting to be the same around the world or over time. Not only do different populations experience different degrees of growth faltering, but the growth faltering is also affected by birth size, which varies considerably across countries (Schneider, 2024).

Another option is simply to exclude children under aged two who are subject to this bias. We have opted for this option because it is simpler to implement, requires fewer assumptions about the relationship between age and stunting and also limits out of sample prediction that would be required for countries without sufficient data to systematically estimate the relationship between age and stunting over time. However, it does mean that our stunting estimates are not as closely comparable to the Joint Malnutrition Estimates (JME), which include children under two, as would be ideal. Thus, we adjust the JME data to make it comparable with our data that excludes children under age two. This is discussed in detail in Appendix H.

B.3 USSR Data

A subset of our data for countries that were formerly part of the USSR were kindly provided by Elizabeth Brainerd, who originally collected the data for her article on living standards in the Soviet Union (Brainerd, 2010). The data were largely drawn from archival collections of height studies that were compiled by the Semashko Institute of Public Hygiene in Moscow. With COVID travel restrictions and archive closures and later, the war in Ukraine, it was not possible to gain access to the original archival material in order to assess these studies using the same methodology that we used for all other studies in our meta-analysis. Thus, the data contributed in this way for countries that were formerly part of the USSR should be taken with a degree of scepticism.[3]

However, despite being unable to scrutinise study-level criteria for these data, we have performed a number of checks to ensure that the data we are including is of high quality. First, we excluded studies that had more than one mean height recorded for the same age group, sex and geographic area, i.e. two height measurements for 8-year-old boys in the city of Moscow in 1934. In these cases, it seemed likely that the studies were comparing two sub-groups within the population, for instance rich and poor or unhealthy and healthy, and given that we did not have information about these sub-groups, it seemed prudent to drop the observations all together rather than introduce potentially unrepresentative studies into the sample. This led to a loss of 666 mean height by age and sex observations.

Second, we sought to ensure that the remaining mean height by age observations had sample sizes above the threshold of 20 in our inclusion criteria. Table B.2 presents the distribution of sample sizes for mean height observations by birth decade for the USSR data. Overall, only 0.1% of mean height observations had sample sizes below 20 and the median sample size was 121. Observations with low sample sizes less than 20 were excluded from our analysis, but the more tricky issue was that the sample size for each mean height observation was missing from a subset of studies. The observations with missing sample sizes made up 11.4% of the total observations, but the share was much

[3]Note that we also corroborated this data for the USSR countries with other studies where we were able to scrutinise study quality carefully.

higher for the 1910 to 1930 birth decades. As mentioned above, we did include studies that did not report the sample size for each mean height observation if the total sample size of the study was reported and was large enough that the mean height observations would have had more than 20 observations. Unfortunately, for these studies, the total study sample size was not reported, so we coud not follow that rule strictly. However, given the sample size distributions that we see for the studies that do report sample size, it seems likely that the remaining studies would meet this requirement. Excluding studies without reported sample sizes leads to large-scale reductions in the number of studies available before the 1950s, which make it more difficult to trace trends in child stunting before the Second World War. Thus, on balance, we think it prudent to include studies without reported sample sizes for the USSR data assuming that they would very likely meet our inclusion criteria.

Table B.2: Distribution of sample sizes (N) for mean height observations by sex and age and by birth decade along with the total number of observations and observations with sample sizes reported for the studies covering the USSR provided by Brainerd (2010)

	Dist. of N for Mean Height Obs.				% of Obs.		Number of Obs.	
Birth Decade	Min	p5	p25	p50	N < 20	N Missing	N Present	Total
1910	13	13	23	77.5	21.4	79.4	14	68
1920	100	100	100	100	0	90.8	8	87
1930	288	288	444	557	0	87.3	10	79
1940	100	104	123	169	0	28.4	116	162
1950	41	91	104	120	0	6.7	1551	1662
1960	15	94	108	125	.1	9	1565	1719
1970	24	54	100	112	0	1.4	821	833
1980	15	39	103	123	.2	11.4	437	493
1990	36	45	100.5	107.5	0	7.7	24	26
Total	13	75	104	121	.1	11.4	4546	5129

Sources: Worldwide Historical Child Stunting Database; Brainerd (2010).

Table B.3 presents the characteristics of the USSR studies from Brainerd (2010). The studies largely covered urban areas either at the community or subnational level. Community studies would be for a specific city like Moscow or St Petersburg whereas subnational studies tended to cover an oblast (equivalent to a province). Brainerd (2010) focussed her analysis on urban areas given that many of the samples were taken in schools and urban areas had higher enrolment rates. However, primary education became

compulsory in the USSR in 1958 and the vast majority of studies were conducted after that date suggesting that children measured in schools should be representative of the population of the spatial unit covered in the study.

Generally the output from the USSR data looks fairly reasonable, which is reassuring. The data definitely skew toward urban children which may mean that the national stunting rates are an underestimate of the stunting rates for each country, but that issue is no different from that of many other countries in our dataset. The only set of results that are difficult to understand are the results for the Central Asian countries. The USSR data shows stunting rates for the birth cohorts in the 1950s and 1960s that are far lower than the first JME data in the late 1990s and lower than other studies from earlier periods in the cases of Uzbekistan and Kyrgyzstan. The urban focus of the vast majority of these studies may explain some of this effect, but these low stunting rates do seem puzzling given the economic development of Central Asia at the time. Thus, our results for Central Asia should be viewed with a good deal of scepticism.

In total and after applying the various exclusion criteria and data cleaning procedures described above and below, we include 220 studies from former USSR countries from this archival data that could not be checked.

Table B.3: Number of stunting estimates from the USSR data by geographic coverage and the share of estimates conducted before and after 1958

	Community		Subnational		National		Share (%)	
Country	Rural	Urban	Rural	Urban	Rural	Urban	<1958	1958+
Russia		28	42	210			11.1	88.9
Belarus	1	7	3	1		1	30.8	69.2
Moldova		4			4		0	100
Ukraine	8	31	8	7	1	1	8.9	91.1
Estonia		5					20	80
Latvia		7			2		0	100
Lithuania	1	5	1				0	100
Armenia		5					20	80
Azerbaijan		8			1		0	100
Georgia		2					50	50
Kazakhstan		1		10		6	0	100
Kyrgyzstan		7				1	12.5	87.5
Tajikistan		2	1			1	0	100
Turkmenistan		1			1		0	100
Uzbekistan		11	3	1		2	5.9	94.1

Sources: Worldwide Historical Child Stunting Database; Brainerd (2010).

C Transcription Protocols and Example Studies

Once studies that met the inclusion criteria were identified, contributors collected systematic information about each study in two ways: by completing Excel forms with the quantitative data and by completing a systematic survey for each study about the inclusion and exclusion criteria and representativeness of the study.

First, depending on the way that the data were reported, contributors completed one of four Excel sheet forms. These Excel forms required contributors to complete two sheets. The first, common across all forms, asked contributors to input bibliographic information for each study, including listing the page numbers from which data was collected. The second sheet provided a structure for the contributor to provide information on children's heights, ages and the way they were measured. The following information was collected in all forms if in slightly different ways.

- Mean Age - One difficulty of the studies is that they often only listed mean heights for whole-year ages. However, this can create confusion as 6 years old can mean 5.5 to 6.49 year olds or 6.0 to 6.99 year olds. Different studies reported this differently, so contributors were clearly instructed to transcribe the mean age for children whose heights were reflected in the measurements.

- Age Units - Sometimes ages were reported in months and other times in years. In addition, this variable captured the width of the age interval for the children measured. This was most frequently one year, but in some studies, means and standard deviations of height were reported for more frequent intervals such as a half or quarter of a year. These widths matter because wider intervals overstate the variance of height at the mean age since they include children say from 4.0 to 4.99, and these need to be adjusted (see Appendix E).

- Sex - Reporting children's heights separately by sex was required for inclusion of the study, and so we tracked height by sex.

- Number of Observations - Where the number of height measurements at each age

was reported, this was recorded. However, this was not always recorded for each mean height observation, and we included studies without sample sizes for each measurement if it was clear from the study overall that the sample size met the inclusion criteria.

- Height Units - Some studies reported height in inches and others in cm. We converted all measurements to cm for clarity.

- Supine Measurements - Young children were often measured lying down rather than standing up. The 2006 WHO growth standard accounts for supine measurement in computing height-for-age Z-scores, so we collected this information in accordance with the standard. Physical anthropologists have developed more and more advanced protocols for measuring children over time, but we have not attempted to record the method used for taking measurements (beyond supine or standing height) nor have we tried to standardise this in any way. This introduces measurement error in our studies, but in practice it would not be possible to adjust for these factors anyway since many studies to not report their precise measurement methodology. This almost exclusively affected children under age two, who were excluded from our analysis anyway.

- Measures of the Mean and Variance of Height - These are reported differently depending on the form in which the data were reported and will be discussed in detail below.

C.1 Forms

C.1.1 Form 1: Frequency Distribution Form

Because percentiles and standard deviations were not invented until the late nineteenth and early twentieth centuries respectively, the earliest form of distributional measures that appear in scientific studies were frequency distributions. Figure C.1 provides a typical example. The number of individuals at different ages are reported in one-inch increments of height. In this case, it is straightforward to compute means and standard deviations

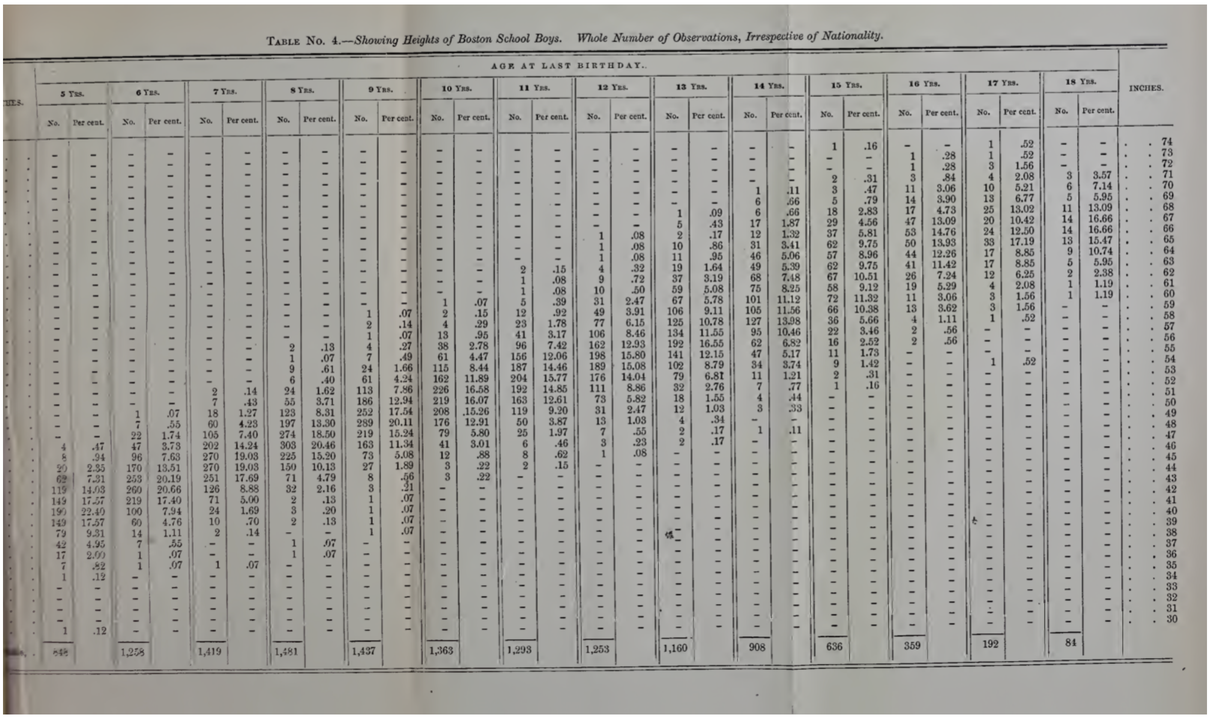

Figure C.1: Bowditch Distributions

Sources: Bowditch (1877, Table 4 - foldout at back of volume)

from the frequency distribution by simply computing the mean and standard deviation of the height levels and applying the sample size at each increment as a weight. In total 10 studies presented frequency distributions in our total dataset.

C.1.2 Form 2: Percentile Form

After percentiles were invented, some studies beginning in the 1890s reported the percentiles of height by age. By assuming height is normally distributed, one can compute the mean and standard deviation of height from two (or more) percentiles. First, we computed the standard deviation by converting the percentiles to their respective Z-scores in the normal distribution.[4] Equation 1 shows that the standard deviation of the distribution is simply the change in the height values of two percentiles (H_{p1} and H_{p2}) over the change in the Z-scores associated with the two percentiles (Z_{p1} and Z_{p2}), or the slope of the line connecting the two points. This means that the best estimate of the standard deviation can be computed as the regression coefficient of a regression of the height values

[4]For instance, the 10th and 90th percentiles relate to Z-scores of -1.28 and 1.28 respectively.

TABLE No. 18.
THE HEIGHT STANDING.

Age at Nearest Birthday.	Sex.	Number of Observations.	Value in Centimetres at the following Percentile Grades.											Average.
			5	10	20	30	40	50	60	70	80	90	95	Centimetr's
Six	Boys.	709	101.77	103.50	105.25	106.73	107.90	109.23	110.40	111.86	113.51	115.81	118.32	108.94
	Girls.	780	100.20	101.41	103.97	105.51	106.78	108.10	109.40	110.61	111.98	115.00	116.90	107.67
Seven	Boys.	1850	105.67	107.56	110.23	111.81	112.97	114.48	115.81	117.13	118.90	121.24	123.39	114.03
	Girls.	1791	104.93	106.24	108.93	110.51	111.89	113.44	115.03	116.32	117.82	120.38	121.94	112.95
Eight	Boys.	2223	109.68	112.28	115.15	117.01	118.44	119.78	121.23	122.72	124.44	126.73	129.05	119.13
	Girls.	2193	110.18	111.86	114.32	115.82	117.40	118.75	120.16	121.42	123.27	125.79	127.79	118.36
Nine	Boys.	2205	115.89	118.11	120.29	121.99	123.38	124.87	126.25	127.87	129.64	131.90	134.06	124.35
	Girls.	2122	115.17	117.12	119.59	121.21	122.76	124.11	125.53	126.54	128.62	130.97	133.42	123.67
Ten	Boys.	2087	120.04	121.92	124.58	126.40	127.98	129.45	130.98	132.54	134.53	137.14	139.45	128.87
	Girls.	2053	119.43	121.34	124.14	125.77	127.32	128.85	130.33	131.82	133.33	136.86	138.84	128.43
Eleven	Boys.	1819	124.53	126.59	129.11	130.95	132.75	134.44	135.85	137.24	139.33	142.18	144.75	133.84
	Girls.	1772	122.95	125.41	128.16	130.29	132.08	133.60	135.25	136.87	139.06	142.16	144.69	133.19
Twelve	Boys.	1653	128.65	130.59	133.26	135.23	136.87	138.57	140.41	141.94	144.05	147.02	149.83	138.21
	Girls.	1732	128.06	130.66	133.53	135.68	137 55	139.54	141.38	143.29	145.58	149.16	152.19	139.11
Thirteen	Boys.	1268	131.36	134.14	137.39	139.51	141.39	143.29	145.12	147.01	149.54	153.55	155.91	142.91
	Girls.	1322	134.16	137.09	139.84	142.12	144.23	146.19	148.26	150.68	152.90	156.03	158.63	146.53
Fourteen	Boys.	925	137.10	139.63	142.30	144.56	146.50	148.86	150.67	153.26	155.77	159.19	162.88	148.58
	Girls.	1085	139.02	141.66	145 68	148.01	150.25	151.94	153.70	155.37	157.26	160.31	161.99	150.84
Fifteen	Boys.	490	140.94	143.69	147.50	150.26	152.65	155.25	157.47	160.19	163.53	168.00	170.44	154.90
	Girls.	680	145.11	148.20	150.82	152.68	154.28	155.82	156.89	158.60	160.55	162.77	164.83	155.04
Sixteen	Boys.	189	146.42	149.38	153.93	156.62	159.45	161.27	163.08	165.47	168.60	171.02	173.55	160.27
	Girls.	420	149.00	150.45	153.05	154.82	156.52	158.03	159.54	160.97	162.65	165.93	167.64	157.52
Seventeen	Boys.	78	154.90	156.90	159.72	161.85	164.20	166.00	168.13	170.20	172.48	175.07	177.05	165.13
	Girls.	206	150.43	152.46	154.73	156.57	157.77	159.40	160.54	162.02	163.83	166.23	168.46	159.33
Eighteen	Girls.	164	151.10	153.47	155.73	157.13	158.54	170.50 159.50	160.57	162.26	164.31	166.45	169.60	159.42
Nineteen	Girls.	85	150.13	151.36	153.00	155.64	158.43	159.56	160.83	162.42	164.11	165.17	167.76	158.46

Porter — The Growth of St. Louis Children. 313

Figure C.2: Porter Distributions

Sources: Porter (1894, p. 313).

on the Z-scores. The coefficient of the regression will show the change in height given by a one-unit change in the Z-score, which is one standard deviation by definition.

$$SD = \frac{H_{p2} - H_{p1}}{Z_{p2} - Z_{p1}} \tag{1}$$

We can then use the computed standard deviation and Z-scores to determine the mean of the distribution when the mean is not reported. As Equation 2 shows, the mean ($\overline{X}$) will be equal to the height value at a given percentile (H_p) minus its Z-score (Z_p) times the standard deviation (sd). This produces estimates of the mean for the value of each percentile, and we simply take the average of these as the best estimate of the mean of the distribution. Figure C.2 presents an example of percentile data from Porter's study of children in St. Louis in 1894. In total, 49 studies included data as percentiles.

$$\overline{X} = H_p - Z_p \times SD \tag{2}$$

C.1.3 Form 3: Mean and Standard Deviation Form

Most commonly the means and standard deviations of height were reported directly. Our first measures of the standard deviation also come from the 1880s but become much more common from the early twentieth century onward. They can be used straightforwardly in our analysis. In total 632 studies reported the mean height directly. Of these, 399 also reported the standard deviation. Standard deviations are imputed for the 233 studies that do not report them (see Appendix G.2 for more information). Figure C.3 presents an example of a study in this form: Habbakuk's study of children in Barry Wales from 1926. Note that this data could also include studies conducted by current historians on historical data where the mean and standard deviation of height by age were reported: for instance, the studies of enslaved people in Trinidad and the US South in the early nineteenth centuries (Higman, 1979; Steckel, 1987).

C.1.4 Form 4: Individual-Level Data Form

Finally, there were 11 studies where the individual-level data on children's heights and precise ages were available. These were relatively recent studies for countries that were not covered in the World Bank dataset, mostly the UK cohort studies.

C.2 Study-level Survey on Data Quality and the Inclusion and Exclusion Criteria

In addition to these Excel forms, the contributors completed a systematic survey for each study, collected using Google Forms. These structured questions ensured that we collected consistent information that allowed us to systematically assess the inclusion and exclusion criteria for each study. The following were the fields/questions that had to be completed for each study. For many questions, there were fixed responses to ensure

Table II. ***Mean weights and heights of sample of Barry children, at each age, compared for boys and girls, and comparison of these means, showing the excess in favour of either sex at different ages.***

Average age	Boys			Girls			Excess in the mean			
	No.	Mean	P.E.	No.	Mean	P.E.	Boys over girls	P.E.	Girls over boys	P.E.
					Weights (kilogrammes)					
3½	145	15·18	·089	131	14·89	·105	·29	·138	—	—
4½	664	16·09	·042	600	15·89	·044	·20	·061	—	—
5½	282	17·47	·081	346	17·06	·078	·41	·112	—	—
6½	363	19·89	·078	324	19·20	·087	·79	·117	—	—
7½	467	20·57	·071	388	20·10	·074	·47	·103	—	—
8½	861	23·24	·063	820	22·62	·063	·62	·090	—	—
9½	692	25·44	·078	669	24·82	·081	·62	·112	—	—
10½	324	27·19	·126	333	26·13	·124	1·06	·177	—	—
11½	—	—	—	—	—	—	—	—	—	—
12½	2021	32·72	·062	1893	33·30	·083	—	—	·58	·103
					Heights (centimetres)					
3½	145	93·24	·260	131	92·28	·266	·96	·372	—	—
4½	664	96·90	·124	600	96·31	·130	·69	·180	—	—
5½	282	102·22	·240	346	101·71	·197	·51	·309	—	—
6½	363	110·36	·197	324	109·43	·206	·93	·285	—	—
7½	467	113·25	·152	388	112·76	·173	·49	·230	—	—
8½	861	120·14	·132	820	119·68	·130	·46	·185	—	—
9½	692	125·00	·148	669	124·35	·159	·65	·217	—	—
10½	324	128·46	·237	333	127·37	·232	1·09	·332	—	—
11½	—	—	—	—	—	—	—	—	—	—
12½	2021	138·71	·099	1893	140·57	·114	—	—	1·86	·151

Table VIII. ***Variation in weights and heights of boys and girls of the same age as expressed in standard deviations, and the corresponding coefficients of variation.***

Average age	Standard deviations				Coefficients of variation			
	Boys		Girls		Boys		Girls	
	S.D.	P.E.	S.D.	P.E.	C.V.	P.E.	C.V.	P.E.
				Weights (kilogrammes)				
3½	1·587	±·063	1·783	±·075	10·46	±·419	11·90	±·503
4½	1·605	±·029	1·582	±·037	9·974	±·186	9·957	±·196
5½	2·036	±·058	2·059	±·055	11·66	±·335	12·07	±·313
6½	2·212	±·055	2·307	±·061	11·12	±·283	12·01	±·406
7½	2·287	±·050	2·174	±·053	11·12	±·250	10·81	±·265
8½	2·716	±·044	2·703	±·045	11·69	±·192	11·95	±·202
9½	2·865	±·052	3·125	±·057	11·27	±·207	12·59	±·236
10½	3·362	±·089	3·350	±·088	12·37	±·332	10·06	±·266
11½	—	—	—	—	—	—	—	—
12½	4·129	±·044	5·344	±·059	12·59	±·136	16·04	±·180
				Heights (centimetres)				
3½	4·704	±·186	4·518	±·189	5·046	±·200	4·897	±·205
4½	4·738	±·088	4·658	±·091	4·890	±·091	4·838	±·094
5½	5·610	±·159	5·432	±·140	5·489	±·156	5·343	±·137
6½	5·584	±·140	5·474	±·145	5·060	±·127	5·001	±·133
7½	4·872	±·107	5·028	±·121	4·302	±·095	4·458	±·108
8½	5·722	±·094	5·504	±·092	4·763	±·078	4·599	±·077
9½	5·772	±·120	6·090	±·112	4·618	±·085	4·898	±·090
10½	6·328	±·168	6·312	±·165	4·926	±·131	4·956	±·129
11½	—	—	—	—	—	—	—	—
12½	6·590	±·070	7·358	±·081	4·750	±·050	5·234	±·057

Figure C.3: Anthropometric Measures of Boys and Girls Living in Barry, Wales

Notes: The children were born between 1902 and 1909 even though the publication came much later.

Sources: Habakkuk (1926, pp. 300, 309).

comparability in responses.

Information about the dataset

- Contributor surname
- Country covered by study
- Dataset name (a unique id for each dataset that took the form country_authorsurname_yearpublished without spaces)
- Have you uploaded a PDF for the original datasource?
- Have you uploaded the datafile?

Characteristics of the children studied

- Birth years of the children in the data
- Birth decades covered
- Ages of the children in the data
- How were ages reported in the data source? - 3 options
  - Ages rounded down (age 1 contains children aged 1.00 to 1.99)
  - Ages rounded to the nearest integer age (age 1 contains children aged 0.50 to 1.49)
  - Measurements reported for more frequent age intervals (e.g. half-yearly or monthly)
- Sex of children measured
- Sample size - select a range for the approximate average sample size available for each whole age by sex
- Heights measured with shoes?

- Describe any discussion in the source about adjustments for shoe height
- Are any height measurements taken as supine length rather than standing height?
- Is there information about the distribution of height?

Characteristics of the study

- Spatial coverage of the study (national, subnational or community)
- Write the spatial coverage of the study (free text)
- Rural/urban coverage of the study
- Is the sample representative?
  - Again, representativeness refers to a targeted population, not the whole population of a country.
  - Here are the instructions listed on the form: 'Is the sample representative of the targeted population of children under age 10? For instance, if the study focused on urban children in Boston, is the sample representative of urban children in Boston? The sample would not be representative, for instance, if the data were drawn from a reformatory school that targeted the working classes.'
- Where the study was not representative, there were additional questions about why the study was not representative, giving the data contributor the opportunity to suggest that there was selection by socioeconomic status or on health. These questions included fixed options and free text so that the data contributor could list in detail what the study design was.
- Finally, there was a free text field asking the data contributor for any additional information that was relevant.

The responses to the form were then linked to the study data provided as an Excel file and cross-checked with the data in the form, particularly the information about the mean age of each age interval. We also validated the data contributors' judgements on representativeness as described in section D.2.

D Data Cleaning and Verification

With 43 people contributing data to this study, it was important to verify and check the data submitted even though there were clear transcription protocols. This section describes (in brief) the checks that were conducted.

D.1 Cross-validation Checks of the Data

When a contributor submitted a study, it was first checked by a team member to ensure that the transcription protocols had been followed both in the excel file with data submitted and in the Google form used to collect study-level information.

We then flagged cases (mean height by age observations) to be independently verified based on the following criteria. We verified cases:

1. where the HAZ score was more than 1 unit from the mean HAZ score for the dataset, sub-category, and sex;
2. where the mean HAZ score was less than -3;[5]
3. where the mean HAZ score was greater than 2;
4. where the coefficient of variation was below the 1st percentile for each age;
5. where the coefficient of variation was above the 99th percentile for each age.

Errors in the transcription could have occurred because of typing errors in the mean height or age of a particular group, and therefore we verified all flagged cases. More worrying would be systematic errors affecting all cases from a study. Thus, we also verified the entirety of the data from studies where more than 20% of height-by-age observations were flagged using the criteria above. The data were largely reliable, but the following were among the more common systematic mistakes:

- contributors recorded the wrong mean age of the age interval reported in the data, i.e. recording the mean age as 4.0 instead of 4.5;

[5] Note that our HAZ score plausibility ranges differ from those of the WHO that use -6 to 6 because our cases are mean HAZ scores rather than individual-level HAZ scores.

- contributors recorded the incorrect heights for ages, i.e. they systematically recorded the heights for a lower age group;
- contributors recorded the wrong units for the height measurements, i.e. inches instead of centimetres;
- contributors recorded the standard error as the standard deviation.

Where errors were found, these were fixed in the original transcription files, and any cases that were verified as correct in the original records were flagged as such in the dataset. Some of these outlier values were later excluded or winsorized: see Appendix F for details.

D.2 Verification of Representativeness

Aside from the actual data being transcribed, it was also important to verify that all participants had followed the inclusion and exclusion criteria in a similar manner. This was straightforward for the more concrete criteria based on sample size or children's ages, but criteria related to representativeness and selection are more likely to vary based on subjective judgements. To ensure that the criteria were followed uniformly, a single member of the team (Juliana Jaramillo-Echeverri) who had access to the original studies and the survey that contributors had completed for each study reviewed all of the studies, systematically checking to ensure that contributors had judged the representativeness similarly. This led to some revision in the classification of representativeness, but overall it confirmed the quality of the contributions made to the meta-analysis.

E Healy Variance Adjustment

With recent data, stunting rates are computed from individual-level data where children's exact height and age are reported. This means that an individual's HAZ score can be computed against the standard/reference distribution at their exact age. However, in historical studies, we often only have mean and distributional measures for height for pooled distributions of children with different exact ages, i.e. the mean and standard deviation of boys aged 4.00 to 4.99. If these pooled individuals are uniformly distributed across the age interval, then the mean height for the pooled group will be equal to the mean height of children at the midpoint age. However, this would not hold for the standard deviation of height, which will be overestimated relative to the standard deviation at the midpoint age.

This problem is well known in the anthropometric literature, and Healy (1962) developed a method for correcting the variance of the pooled distribution in order to get an approximation of the variance of the midpoint distribution. He postulated the following equation:

$$\sigma_p^2 = \sigma_m^2 + \frac{i^2}{12} \tag{3}$$

where σ_p^2 is the variance of the pooled distribution of heights, σ_m^2 is the variance of the midpoint distribution of heights, and i 'is the height increment between the exact ages that form the endpoints of the age group' (Healy, 1962; Steckel, 1996). Since we often lack information in historical sources on the height measurements of the increment's endpoints, we also replace i with the height increment between the midpoint ages above and below the midpoint age, i.e. if the midpoint age is 3.5, the increment would be between the heights at 4.5 and 2.5 divided by two. When solving for the variance of the midpoint distribution, this yields:

$$\sigma_m^2 = \sigma_p^2 - \frac{\left(\dfrac{h_{m+t} - h_{m-t}}{2}\right)^2}{12} \tag{4}$$

Finally, in order to adjust the variance when we only have two consecutive ages (or at the endpoints of the age distribution), we replace i with the height increment between the midpoint and the increment above or below midpoint.

$$\sigma_m^2 = \sigma_p^2 - \frac{(h_{m+t} - h_m)^2}{12} \qquad \text{or} \qquad \sigma_m^2 = \sigma_p^2 - \frac{(h_m - h_{m-t})^2}{12} \tag{5}$$

Healy's equation assumes that both the mean and variance of height increase linearly across the age increment, that the midpoint age height distribution is normal and that the children in the pooled distribution are uniformly distributed across the whole pooled age range (Healy, 1962). Note that Equation 4 will accurately adjust the pooled variance no matter how wide or narrow the pooled age interval, though the necessary assumptions are more likely to hold over shorter age intervals.

We verified these methods using individual-level DHS data and historical data that reported distributions of height by monthly age groups and found that the method significantly reduced the overestimation of the midpoint variance when using the pooled distribution. The differences between the pooled and midpoint variances were greatest for children under the age of two when children are growing rapidly and their growth is decelerating rapidly.

These adjustments were applied to all standard deviations derived from pooled distributions before the final stunting rates were computed: see Appendix G.

F Dealing with Outliers

Once the data had been fully verified, we were left with a number of extreme values of mean and standard deviation of height that had been verified as correct in the original study. These outliers could have been typos in the original source (either in height or age), or could have reflected methodological inconsistencies in the original study.

We treat errors in the mean and standard deviation differently. We flag mean errors by comparing each height-for-age measurement, i.e. the HAZ score of four-year-old boys, to the mean HAZ score of all boys in that study. We only compare children within the same sub-group of the study if different sub-groups are delineated, i.e. region, city, etc. We exclude measurements where the HAZ score is more than one HAZ point from the mean HAZ score for the study. For instance, if the four-year-olds mentioned above had a HAZ score of -3, but the mean HAZ for boys in their study was -1.5, the HAZ observation for the four-year-olds would be excluded when computing the stunting rate for the study. In all, we drop 107 HAZ observations, which equates to 0.38% of all height by age observations. Thus, the share of data dropped is very small and is unlikely to alter the results of our analysis dramatically. However, we do not simply disregard these values. Studies where we drop more than 10% of height by age observations are also downgraded in the certainty of evidence exercise described in Appendix I because these outliers do reduce the certainty we should ascribe to a study.

We take a different approach with outliers in the standard deviation. Given our very large collection of means and standard deviations, we can compute distributions of coefficients of variation of height by whole-year age and sex to get a sense of normal variability in height at various ages (see Table F.1). These computations are limited (as stated above) to observations where the mean and standard deviation are computed from a sample of 20 or more children. We then use the 5th and 95th percentiles for each age and sex to winsorize outlier coefficients of variation, i.e. coefficients of variation below the 5th percentile and above the 95th percentile are reassigned the value of the 5th and 95th percentile respectively. We then compute new standard deviations using the winsorized coefficients of variation for observations with extreme values of coefficient of variation. By

definition this leads to the recalculation of 10 per cent of standard deviations of height.

We believe that this method is appropriate because there do seem to be plausible ranges of coefficients of variation of heights. High coefficients of variation likely reflect measurement error in the reporting of ages so that children outside the age range are included in the computation of the variance. This form of error is especially important in historical datasets where it was more difficult to verify children's ages. Measurement error in age would not affect the mean height as long as the measurement error is uniform, but winsorizing high coefficients of variation helps to produce a more plausible stunting estimate for these datasets. Low coefficients of variation are more difficult to explain: we have checked thoroughly to ensure that we are not mistaking standard errors for standard deviations. However, again, we know that there are plausible ranges of coefficient of variation of height, so it makes sense to winsorize these implausible values.

Table F.1: Distribution of Height Coefficients of Variation by Age and Sex

				Percentiles									
Age	Sex	N	Min	1	5	10	25	50	75	90	95	99	Max
2	0	537	1.07	1.66	2.49	2.98	3.62	4.10	4.93	6.25	7.12	8.70	10.93
3	0	594	0.26	2.02	2.89	3.30	3.74	4.19	4.93	6.23	7.09	8.98	11.34
4	0	664	0.17	2.27	3.06	3.46	3.85	4.24	4.82	6.15	6.77	9.16	22.98
5	0	659	1.89	2.44	3.26	3.49	3.87	4.22	4.68	5.64	6.37	8.74	30.50
6	0	952	1.99	2.57	3.43	3.63	3.92	4.21	4.66	5.33	5.99	8.22	14.28
7	0	1441	0.37	2.69	3.40	3.58	3.88	4.12	4.52	5.10	5.55	7.08	12.60
8	0	1434	0.26	2.93	3.51	3.70	3.98	4.26	4.63	5.04	5.48	6.92	11.80
9	0	1425	0.28	3.12	3.66	3.87	4.13	4.38	4.71	5.19	5.63	6.77	18.73
10	0	1412	0.27	3.04	3.82	4.07	4.34	4.61	4.92	5.39	5.78	6.76	12.91
2	1	548	0.43	1.75	2.79	3.05	3.56	4.08	4.92	6.12	6.92	8.85	17.35
3	1	607	0.21	2.07	2.83	3.19	3.69	4.16	4.94	6.07	7.05	9.01	12.47
4	1	667	0.99	2.48	3.11	3.38	3.80	4.20	4.77	5.96	6.84	9.51	15.23
5	1	671	1.13	2.42	3.24	3.47	3.87	4.21	4.70	5.66	6.39	8.41	13.67
6	1	996	0.82	2.49	3.25	3.51	3.88	4.16	4.60	5.26	5.89	7.78	12.94
7	1	1490	0.34	2.64	3.36	3.58	3.85	4.12	4.52	4.99	5.49	7.11	11.46
8	1	1478	0.12	2.82	3.43	3.65	3.92	4.18	4.54	5.05	5.53	6.76	11.18
9	1	1478	0.24	2.83	3.50	3.74	3.96	4.23	4.57	5.03	5.51	6.71	10.67
10	1	1459	0.18	2.59	3.53	3.73	4.03	4.30	4.62	5.04	5.41	6.57	11.71

Notes: Age is rounded down age. Sex is equal to 0 for females and 1 for males. Coefficients of variation are expressed as percentages. The coefficients of variation are reported after the Healy adjustment to the variance (see Appendix E for details).

Sources: Worldwide Historical Child Stunting Database.

G Computing Study-Level Stunting Rates

To summarise the process to this point, we have collected or transformed the data reported in the studies into estimates of mean height and the standard deviation of height for each sex and age. In addition, as described in Appendix E, we have adjusted the variance of the height distributions to account for the fact that the height distributions often covered children for a whole age interval. This appendix explains how we take these data and compute a single stunting rate for a study.

G.1 Computing Study-Level Stunting Rates from Means and Standard Deviations

Where we have means and standard deviations for all ages, we first convert the mean and standard deviations from cm into height-for-age Z-score units relative to the WHO standard/reference. Because the height-for-age Z-scores are comparable across ages, we can then combine these Z-scores into a common distribution. The mean of this common distribution is the weighted average of the Z-scores for each age, weighted by the sample size at each age if this is available or equally if the sample size by age is missing. Computing the standard deviation of the common distribution is more complicated. We cannot use the simple pooled standard deviation used to compute a confidence interval because this provides a within sub-group standard deviation. Following Higgins et al. (2019, p. 168), we instead estimate the standard deviation of the combined group, using the following equation:

$$\sqrt{\frac{(N_1-1)SD_1^2+(N_2-1)SD_2^2+\frac{N_1N_2}{N_1+N_2}\left(M_1^2+M_2^2-2M_1M_2\right)}{N_1+N_2-1}} \tag{6}$$

where 1 and 2 index two age groups and M is the mean, N is the sample size, and SD is the standard deviation of each group respectively. Because in many cases we must combine more than one group, we follow Higgins et al. (2019) and iterate through this process combining the new joint standard deviation for groups 1 and 2 with group 3 and

so forth. Where we do not precisely observe the sample size at each age, we again assume equal weighting of the age groups using the estimated sample size for each age group as the weight.

This leaves us with a combined mean and standard deviation for each study (or sub-study) by sex. Because the mean and standard deviation are expressed in terms of the height-for-age Z-score, the stunting rate is simply the area under the normal distribution (defined by the mean and standard deviation) below the stunting threshold of -2. To get a final, study-level stunting rate, we average the stunting rate of boys and girls with equal weight.

This process is illustrated graphically in Figure G.1. Figure G.1A presents the height distributions of Guatemalan girls at various ages measured during the INCAP study (Martorell et al., 1995). These height distributions are converted to height-for-age Z-scores in Figure G.1B. Figure G.1C reproduces the combine distribution for the children at all ages from the weighted average and combined standard deviation. Finally, Figure G.1D shows the stunting rate as the area under the combined distribution below the stunting threshold of -2.

G.2 Computing Study-Level Stunting Rates from Mean-only Data

As mentioned in Appendix C.1.3, there were 233 studies that only reported the mean height of children without the standard deviation. These studies reflect a wide range of countries and were especially prevalent in the late nineteenth and early twentieth centuries, which makes them particularly valuable. Thus, we sought a method for imputing standard deviations for these studies so that we could compute a stunting rate.

However, before explaining this imputation process, it is first important to understand the extent to which imputing the variance of height will impact the stunting rate. When we assume that height and HAZ are normally distributed, the stunting rate is mechanically determined by the mean and sd of height or HAZ. To get a sense of this, in Figure G.2A we plot the mean height-for-age Z-score (HAZ) and its corresponding stunting rate for

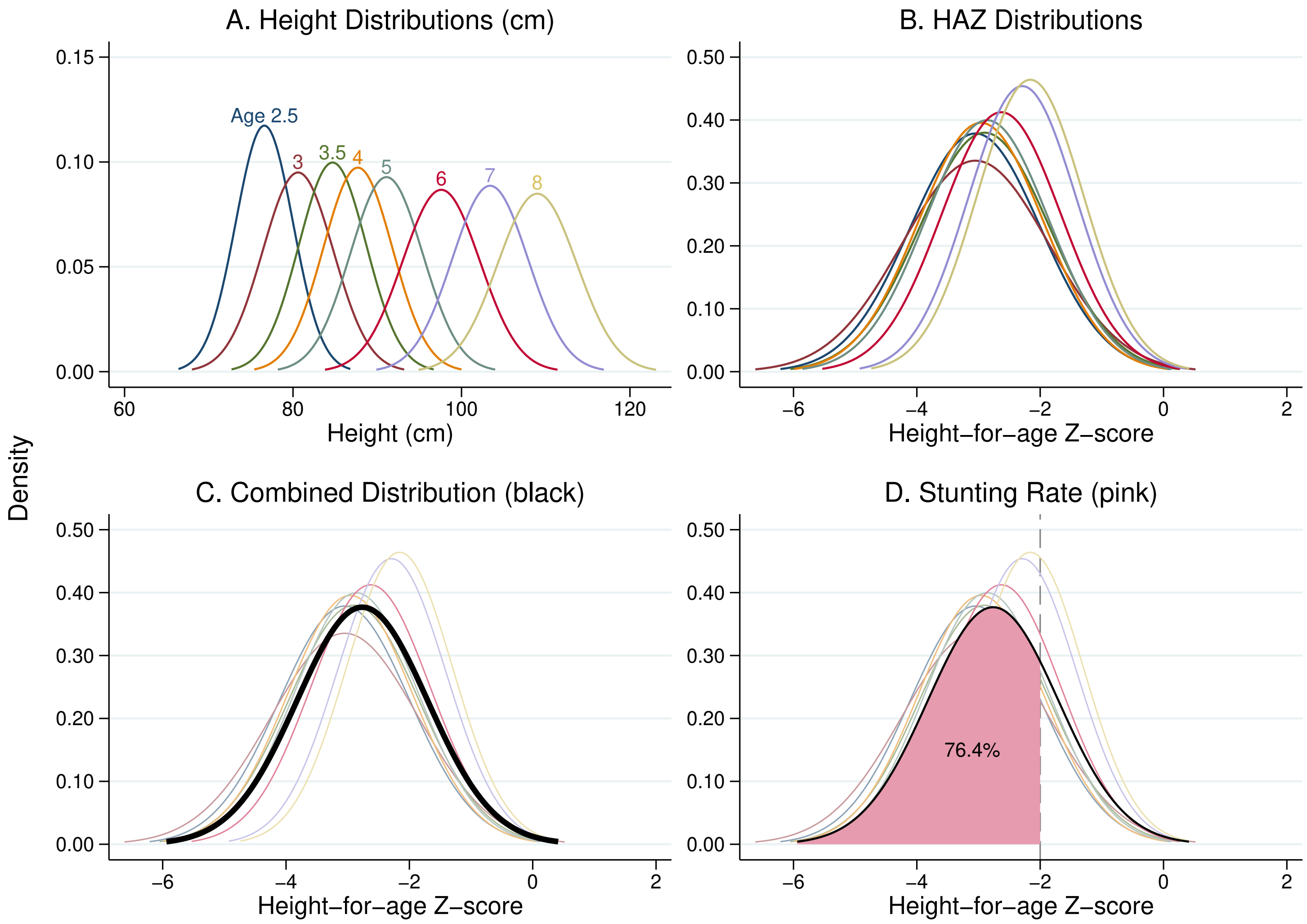

Figure G.1: Process of aggregating height distributions at various ages into a single stunting rate

Notes: The height distributions are for Guatemalan girls measured in 1977 as part of the INCAP study. See text for how the computations work.

Sources: Data from Martorell et al. (1995).

all studies (and sub-studies) which report both means and standard deviations of height, i.e. the studies discussed in the previous section (Appendix G.1). It is clear from this figure that the stunting rate at each level of mean HAZ is limited. This is not because population mean HAZ is limited: we observe values of mean HAZ from -3.66 to 0.99 across the studies in our meta-analysis. Instead, the standard deviation of HAZ is the limiting factor: note that because HAZ is age and mean standardised, we can compare HAZ SDs across studies much like a coefficient of variation. The 5th and 95th percentile of the HAZ SD are 0.82 and 1.27 respectively with a median of 0.96: the SD of the standard/reference is equal to one. We use these percentiles of the SD to create bounds for the stunting rate at each level of mean HAZ: i.e. we assume that the standard deviation of HAZ is constant at the 5th or 95th percentile of standard deviations and then compute the stunting rate as the mean HAZ moves from -5 to 1. These are the blue and lavender dashed lines in Figure G.2A. If our existing studies are representative, this implies that 90% of population stunting rates should fall between the two bounds.

Figure G.2B shows how the absolute difference between the two bounds, i.e. the gap between them, varies with respect to mean HAZ. The bounds are very narrow with the maximum gap being 10.23 percentage points of the stunting rate. Thus, although imputing the standard deviation of height or HAZ may lead to some error, it will not be especially large.

Note also that the symmetrical shape of the normal distribution mechanically leads the variance to be less important when the stunting threshold is close to the mean HAZ. If the mean HAZ is equal to the stunting threshold, then it does not matter what the variance of the distribution is: the stunting rate will always be 50%.

The exercises above rely heavily on the assumption that height (and HAZ) is normally distributed. Given that we only have frequency distributions or individual-level data for a small subset of studies, it is difficult to systematically test these assumptions using our own data. However, many other studies have found that assuming normality in the case of height is acceptable. In addition, Roth et al. (2017) use recent individual-level DHS surveys to show that growth faltering is a population-level outcome driven mostly

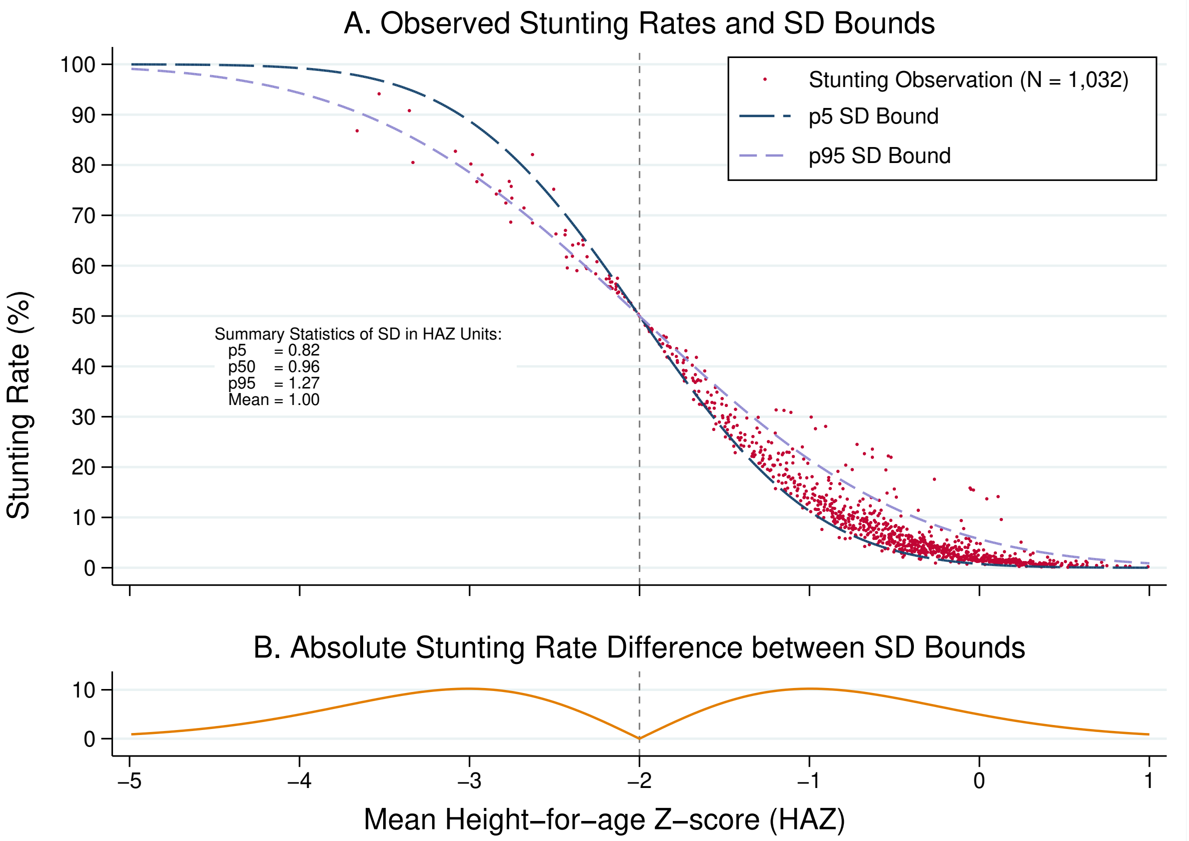

Figure G.2: Relationship between Mean Height-for-age Z-score (HAZ), SD of HAZ and the Stunting Rate

Sources: Worldwide Historical Stunting Dataset.

by shifts in the entire HAZ distribution (changes in mean HAZ) rather than changes in the dispersion of height or the presence of growth faltering in a sub-population. These findings are consistent with mean HAZ being the most important determinant of stunting as we find in Figure G.2.

Given our findings above, we believe it is justified to impute stunting rates for studies that only report the mean height of populations. We impute the standard deviations for height at each age and sex by using the median coefficient of variations of height for the studies in our analysis that reported mean and standard deviation (see Table F.1). These coefficients of variation were computed after the Healy adjustment, and therefore reflect the coefficient of variation for children at exact ages. We then combine the height distributions at different ages in the manner described in Appendix G.1 and using Equation 6.

H The Unicef/WHO/World Bank Joint Malnutrition Estimates

As mentioned above, the goal of this project was to extend the Joint Malnutrition Estimates (JME) produced by Unicef, the WHO and the World Bank Group as far back in time as possible. This meant that we focussed on periods before the 1990s unless to produce stunting estimates for countries not covered in the JME dataset. Unicef et al. (2021) provides technical details for the construction of the JME dataset, but there are a few details that matter for connecting our data to the JME dataset that are addressed in this appendix.

H.1 Background of the JME Dataset

The JME dataset was first published in 2012 in order to track progress related to the child malnutrition goals within the Millennium Development Goals. The dataset is constructed from household surveys in a large number of countries. The most common surveys included in the JME dataset are Demographic and Health Surveys (DHS), Multiple Indicator Cluster Surveys (MICS), SMART Surveys and Living Standards Measurement Surveys (LSMS). Where microdata were available, the studies were reanalysed to ensure that child malnutrition was analysed consistently using the new 2006 WHO child growth standards. For studies where microdata were not available, the reported stunting rates were used in the analysis. The JME group made three further adjustments to the stunting estimates from household surveys (Unicef et al., 2021):

1. **Age Adjustment:** where the original survey did not include children from the complete 0 to 5 age range (i.e. the survey only included children aged 3 to 5), the group used the relative proportions of stunting at each age in the nearest study for the same country to predict a stunting rate for the complete age range. This affected 63 out of 957 studies in the 2021 edition of the JME dataset (Unicef et al., 2021).

2. **Rural to National Adjustment:** where the original survey was only representative nationally of the rural population, the JME group used the relative proportion of stunting rates in rural and urban areas in another nationally representative study from the same country to impute the nationally representative stunting rate for the rural-only study. This adjustment was made for 26 out of 957 studies in the 2021 edition of the JME dataset (Unicef et al., 2021).

3. **Growth Reference Adjustment:** where original microdata were no longer available and studies were conducted before the adoption of the new 2006 WHO child growth standards, the stunting estimates based on the 1977 NCHS/WHO growth reference were adjusted to make them comparable with the new growth standard. These adjustments were made following an algorithm described in Yang and de Onis (2008). This adjustment was made for 178 out of 957 studies in the 2021 edition of the JME dataset (Unicef et al., 2021).

Precise stunting estimates are reported from the original surveys, but since 2021, the JME group has also produced model-based annual estimates of child stunting at the country-level covering the period 2000 to the present. These model-based estimations are discussed at length in Unicef et al. (2021), but because we are linking our historical study-level stunting rates to the JME dataset, we do not use the model predictions from the JME, instead relying on the survey estimates which are comparable with our earlier data (UNICEF/WHO/World Bank, 2023).

H.2 Further Adjustments to the JME Dataset

As mentioned above in Appendix B.2, we exclude stunting observations for children under the age of two from our meta-analysis. This is because the vast majority of our data comes from children over the age of two and adjusting for the differences in age composition across such a large set of heterogeneous studies is difficult. There are few historical studies where we have data for both older children (age 8-10) and also children under two, and even for those studies where this data is present, it is difficult to assume that the

relationship would be the same across countries or over time.

While excluding children under two reduces heterogeneity across historical studies, making the historical stunting estimates easier to interpret and more comparable, it also has the perverse effect of making our estimates incompatible with the typical definition of child stunting in the JME dataset, which includes children aged 0 to 2. Because of the well-known pattern of growth faltering whereby children start their lives relatively close to the mean of the modern standard and fall behind in the first two years, stunting rates are mechanically lower for children aged 0 to 2. Thus, the JME stunting estimates for children aged 0 to 5 will be systematically lower than our stunting estimates because they include younger children.

To overcome this issue, we use the expanded JME dataset which reports stunting rates for children aged 2 to 5 for 762 (72.5%) studies (UNICEF/WHO/World Bank, 2023). Thus, when a stunting rate for children age 2 to 5 is available, we use this in our comparisons with the historical data. In order to avoid losing information from the remaining 289 studies, we impute values of stunting for ages 2 to 5 from the stunting rate for ages 0 to 5 among studies where both are present (see Figure H.1). Following the method described in Yang and de Onis (2008), we regressed the logit transformed stunting rate aged 2 to 5 on the logit transformed stunting rate aged 0 to 5 (Equations 7 and 8) and used the coefficients to predict stunting rates aged 2 to 5 for studies where age-specific stunting rates were not available for children aged 2 to 5. Yang and de Onis (2008) use the logit transformation because the stunting rate is bounded between 0 and and 1. The regression fit is very strong with an R-square of 0.964. Thus, we are able to adjust all JME studies to be comparable with our own studies, which exclude children under age two.

$$logit(S_{2-5}) = a + b \times logit(S_{0-5}) \tag{7}$$

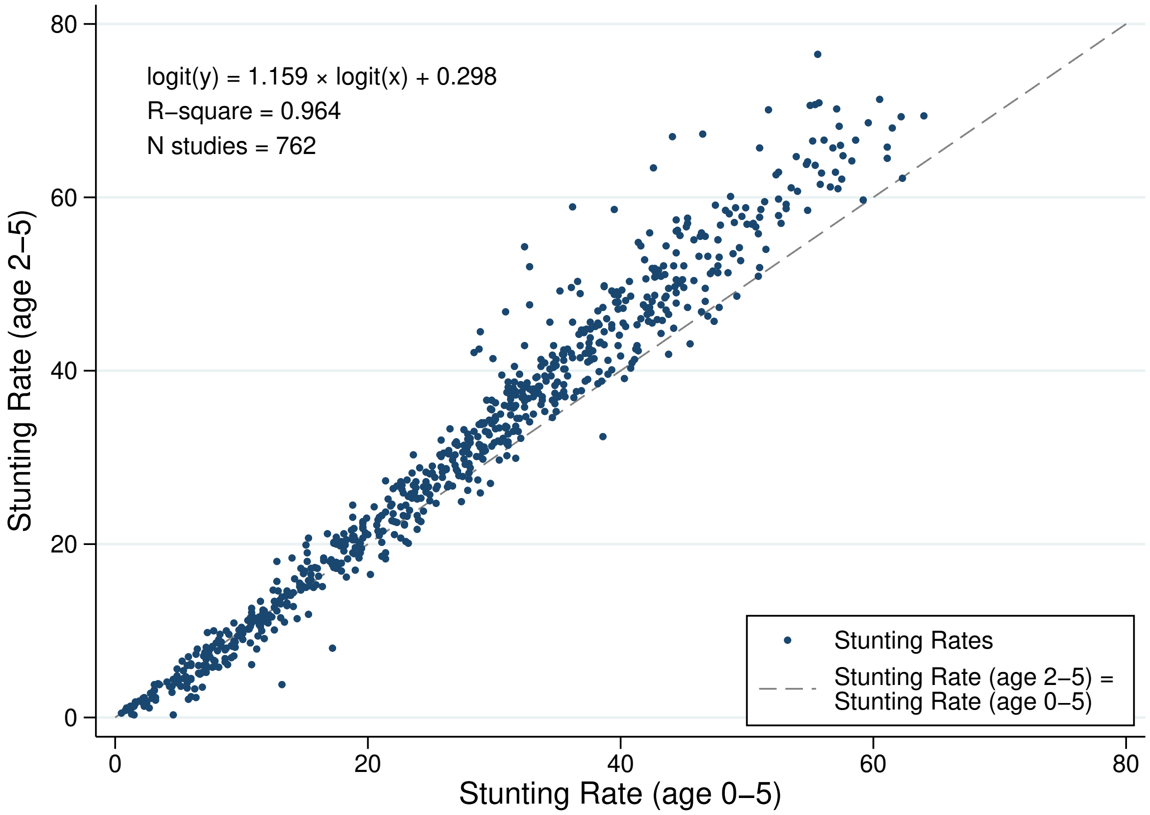

Figure H.1: Relationship between Stunting Rate Age 2-5 and Stunting Rate Age 0-5

Notes: The relationship is restricted to studies in the expanded JME 2023 dataset that report both stunting at ages 0 to 5 and stunting at ages 2 to 5. Both stunting rates are logit transformed and then regressed (see Equations 7 and 8). The regression coefficient is highly statistically significant with a standard error of 0.008 and a t-statistic of 141.8.

Sources: UNICEF/WHO/World Bank (2023).

$$logit(S) = \ln\left(\frac{S}{1-S}\right) \tag{8}$$

Figure H.2 presents the stunting estimates for ages 2 to 5 included in the JME dataset. As noted previously, there are relatively few studies from the 1980s birth cohorts and the density of studies only becomes consistent from the mid 1990s cohorts onward. There are 1,051 studies overall representing 159 countries with a median of 6 studies per country.

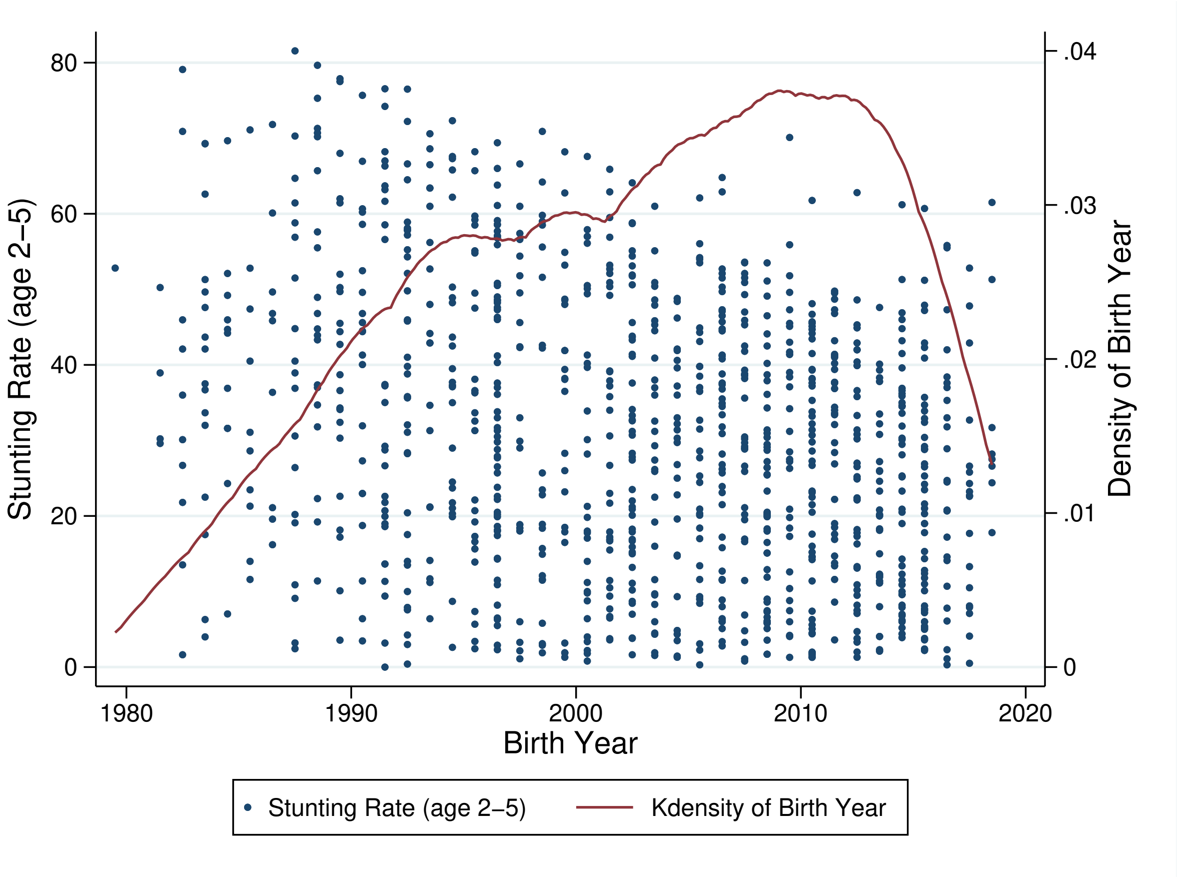

Figure H.2: Country-level stunting estimates (age 2-5) from the JME

Notes: Procedure for imputing stunting estimates aged 2-5 described in the text. Overall, there are 1,051 stunting estimates in the JME reflecting 159 countries with a median of 6 studies per country.

Sources: UNICEF/WHO/World Bank (2023).

I Assessing Certainty of Evidence

As mentioned in the main text, one of the main limitations of meta-analysis is the heterogeneity of the individual studies that we have found. Because these studies span 150 years, they have different methodologies and include both nationally representative and small community studies. This meant that it was important for us to systematically assess the study quality in a uniform way. This appendix presents out approach.

I.1 Adapting the GRADE System to Assess the Certainty of Evidence

To assess the certainty of evidence in our study, we have adapted the GRADE System of evaluating the quality of studies (Schünemann et al., 2019). Since the GRADE System was built for assessing the certainty of evidence of treatment effects, it is not entirely appropriate to a study that is collecting simple descriptive data. However, we have considered the domains within the GRADE System for downgrading or upgrading the certainty of evidence and adapted them for a descriptive meta-analysis. To summarise the certainty of evidence for each study, we create a certainty of evidence score. Each study starts with a score of 10 and receives score deductions within each domain of the GRADE System where the certainty of evidence is found to be lacking. The five domains for downgrading the certainty of evidence level and the scoring system are summarised in Table I.1.

The first domain is 'risk of bias', which refers to problems related to the experimental design of a study or its execution, for instance, high levels of loss to follow up. The equivalent in our setting would be studies with poor sampling design or studies that were not representative of their targeted population. As described in Appendix B.2, we exclude studies that are not representative, i.e. studies targeting a particular socioeconomic group or healthy or unhealthy individuals. Thus, our studies already are rather rigorous with respect to risk of bias. We have not systematically coded the sampling strategy for each study because sampling methodology has changed dramatically over time, making it very difficult to create a common classification system across all studies. Early studies also

Table I.1: Certainty of Evidence Score Deductions Based on Domains of Certainty in the GRADE System

Certainty of Evidence Domain	CoE Score Deduction
Risk of bias	
Exclusion of non-representative studies	
Unexplained heterogeneity	
$\geq$ 10% of observations outside $\pm$ 0.5 HAZ score from study mean	-1
$\geq$ 10% of observations outside $\pm$ 1 HAZ score from study mean	-2
Indirectness of evidence	
Data present for only one sex	-1
Study includes children over age 5	-1
National coverage but only urban or rural	-1
Subnational coverage (both urban and rural)	-1
Subnational coverage but only urban or rural	-2
Community coverage	-3
Imprecision of the results	
Total study sample size $\geq$ 500 and $<$ 1,000	-1
Total study sample size $\geq$ 100 and $<$ 500	-2
Total study sample size $<$ 100	-3
Mean-only stunting estimates	-1
High probability of publication bias	
Not relevant to our study drawing on descriptive results	

Notes: This table presents the score deductions for different domains of certainty of evidence based on the GRADE System. Study-level certainty-of-evidence scores are presented in the dataset associated with this article described in Appendix J.

Sources: Schünemann et al. (2019).

were less likely to describe their sampling strategy in detail, making it difficult to fully assess the sampling methodology. Finally, while auxologists are quite concerned about the techniques used measure children, these were also not reported systematically, so it was difficult to track this beyond noting whether children at specific ages were measured standing up or supine.

The second domain considered to downgrade the certainty of evidence is 'unexplained heterogeneity', which means studies that produce wide ranges of treatment effects. In our study, we see this where there is heterogeneity in the mean height-for-age Z-scores at different ages. Large heterogeneity in mean HAZ scores by age would imply drastically different stunting rates for children just a few years apart and may suggest that the sampling procedure was not robust or that there may be selection on unobservable characteristics correlated with age as discussed by Schneider (2020). Thus, we deduct one certainty of evidence point if more than 10% of the observations are outside plus or minus 0.5 HAZ score from the study mean and two points if more than 10% of the observations are outside plus or minus 1 HAZ scores from the study mean.[6] While we do not have precise reasons for rejecting these studies, the heterogeneity in mean HAZ scores by age justifies reducing the certainty of evidence in the study.

The third domain is 'indirectness of evidence', which Schünemann et al. (2019, p. 394) define as when studies 'address a restricted version of the main review question in terms of population, intervention, comparator or outcomes'. This is a more familiar problem for our set of studies. There are a number of cases where we only partially observe the data that we would truly like. For instance, height observations may only be available for one sex, which means we are forced to extrapolate from one sex to the other. In addition, the fact that we include children over the age of five means that our population is not exactly what would be ideal for computing the stunting rate. Finally, the varying spatial coverage of the data means that we rarely have nationally representative studies (see Figure K.1). Thus, we deduct certainty of evidence points based on these factors. We deduct one point for studies that only include one sex and another for studies that include children above

[6]These assessments were made before the exclusion of outliers.

the age of five. We deduct different levels of points depending on the spatial coverage of the study with community-level studies losing three points while studies with national coverage of only the urban or rural population loosing only one point (see Table I.1 for details).

The fourth domain considers the imprecision of the results. This is related to the confidence intervals surrounding our estimates. In our meta-analysis, this applies in two ways. First, studies with relatively small sample sizes will obviously have wider confidence intervals around their stunting rates. Thus, we deduct between one and three points if the study total sample size is below certain thresholds. Note that this total sample size only includes children at ages that meet the inclusion and exclusion criteria, so we do not include any adolescents or children under two measured in the sample size. Another source of imprecision in our meta-analysis are studies for which we only have mean heights and for which we impute the standard deviation of height. As discussed in Appendix G.2, this can lead to a small amount of error in the estimated stunting rates and therefore reduces the certainty we can have in our stunting estimates. We deduct one certainty of evidence point for the mean-only studies.

The final domain for downgrading the certainty of evidence in the GRADE System is 'high probability of publication bias'. This seems less relevant to our descriptive study since there is no special incentive to publish one descriptive study rather than another. In fact, the nature of these studies is in showing change over time or comparisons within or across countries. Therefore, there should not be publication bias in the way we might expect when publication is unfortunately linked to the statistical significance of the treatment effect.

The GRADE System also has domains for upgrading the certainty of evidence, but all of these are related specifically to treatment effects, and therefore are not relevant to our descriptive meta-analysis.

Thus, once we have scored each study following the rules in Table I.1, we are left with a certainty score for each study in our meta-analysis. It is important to note the limitations of these scores. The point deductions under each domain are somewhat arbitrary: it is

difficult to assess the relative importance of only having data for one sex and including data for children over age five.[7] This means that the scores are not able to assess certainty in a scalar form but can perhaps help us understand the ordinal ranking of studies by certainty of evidence.

I.2 Certainty of Evidence in our Meta-analysis

This section explores the certainty of evidence across the dataset using the scores defined in the previous section. To demonstrate how the scores work, it might be helpful to start with a few examples. The country with the highest certainty of evidence going back in time is Japan where the Ministry of Education measured all children in school (or a large representative sample of children in school) from the beginning of the twentieth century onward. These data have little unexplained heterogeneity and have a vast sample size, but unfortunately, before 1948, only the mean height of individuals was reported. In addition, the Ministry of Education's focus on children in school means that children under age five were never included in this data (Schneider et al., 2021). Thus, these data are given a certainty of evidence score of eight until standard deviations are reported in the 1940s, when they switch to a score of nine.

For other studies, we can be far less confident in their evidence even when the studies were extremely impressive. For instance, the study of Boston schoolchildren conducted by Bowditch in the 1860s mentioned above in Figure C.1 included more than 30,000 children and presented frequency distributions from which one can compute a standard deviation. However, because it was a community-level study and only reported on children aged five and older, its certainty of evidence score is still only six. While Bowditch's study gives a very precise estimate of stunting in Boston, it is difficult to extrapolate from his study to the rest of the United States.

Figure I.1 presents the sub-study-level stunting rates by birth year, shading each stunting observation by its certainty of evidence score. This figure excludes the JME data to give a better sense of the quality of evidence among studies that we discovered

[7] In practice, score deductions tend to be correlated. For instance, community studies tend to have small sample sizes.

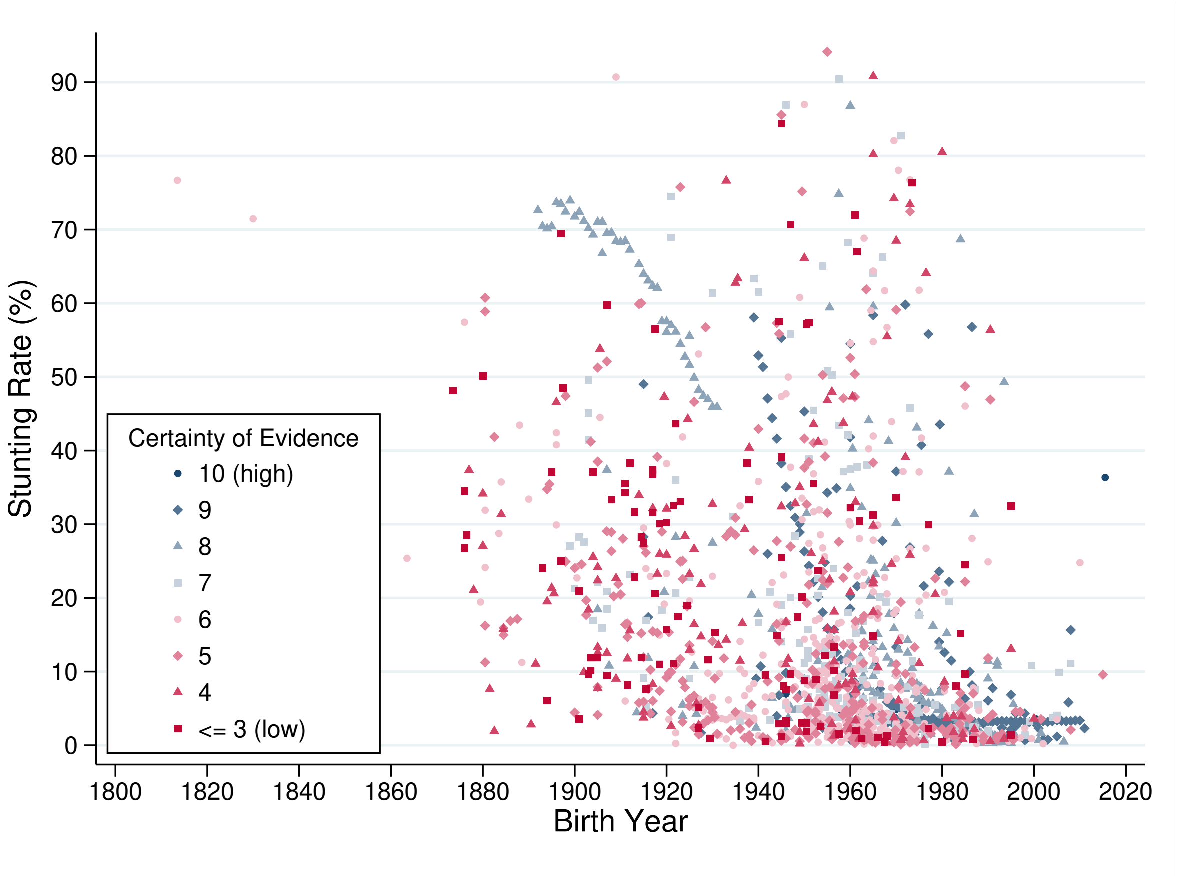

Figure I.1: Certainty of Evidence Scores by Study

Notes: Studies found through the meta-analysis, **excluding** recent studies that are covered in the JME dataset.
Sources: Worldwide Historical Stunting Dataset.

through our meta-analysis. It is clear that the certainty of evidence increases over time, but there are some certainly some studies of relatively high certainty of evidence in the early twentieth century as well.

Figure I.2 shows the change in the certainty of evidence of studies over time including the JME data, highlighting again the improvement in the certainty of evidence over time. However, study-level certainty of evidence is not as important for estimating country-level stunting as the certainty of evidence at the country level. It could be that all the high-quality studies are clustered in a small number of countries rather than being dispersed across countries. Figure I.3 shows the distribution of mean certainty-of-evidence scores at the country level by birth decade. While there is some improvement at the end of the nineteenth century, the median country-level mean certainty-of-evidence score is more or less stable at 6 until the introduction of the JME data in the late twentieth century.

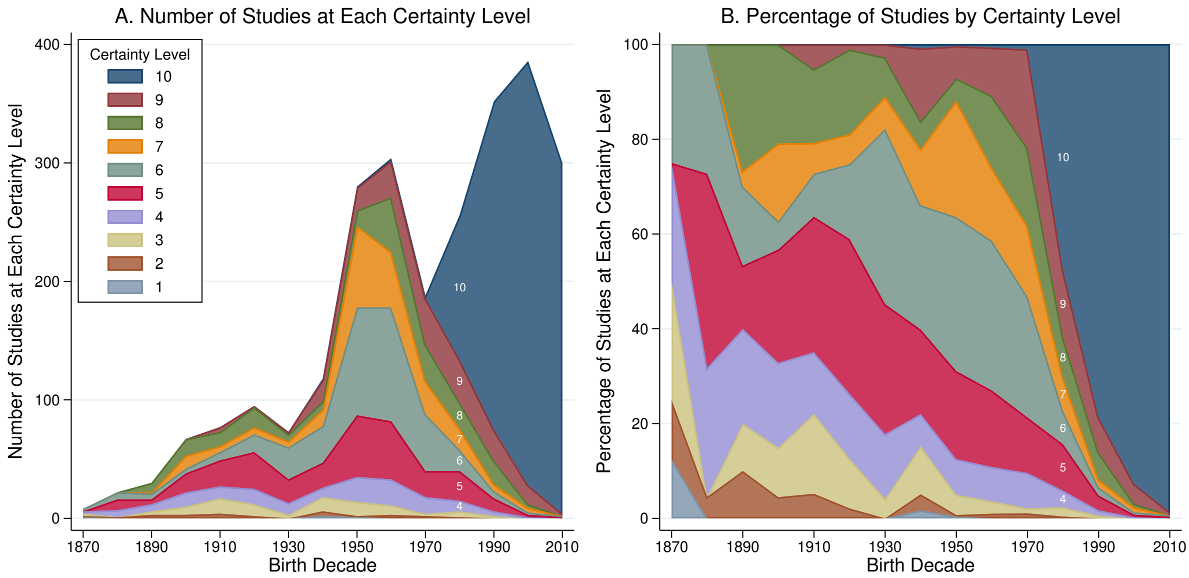

Figure I.2: Certainty of Evidence Scores by Study

Notes: Studies found through the meta-analysis, **including** recent studies that are covered in the JME dataset. Certainty levels are determined based on our adaptation of the GRADE system (see Table I.1.)

Sources: Worldwide Historical Stunting Dataset and UNICEF/WHO/World Bank (2023).

However, there are some improvements when looking at the underlying distribution, with study quality improving at other percentiles of the distribution. These graphs emphasise the heterogeneity of certainty of evidence, especially in the late-nineteenth and early-twentieth centuries, and suggest caution when interpreting the results.

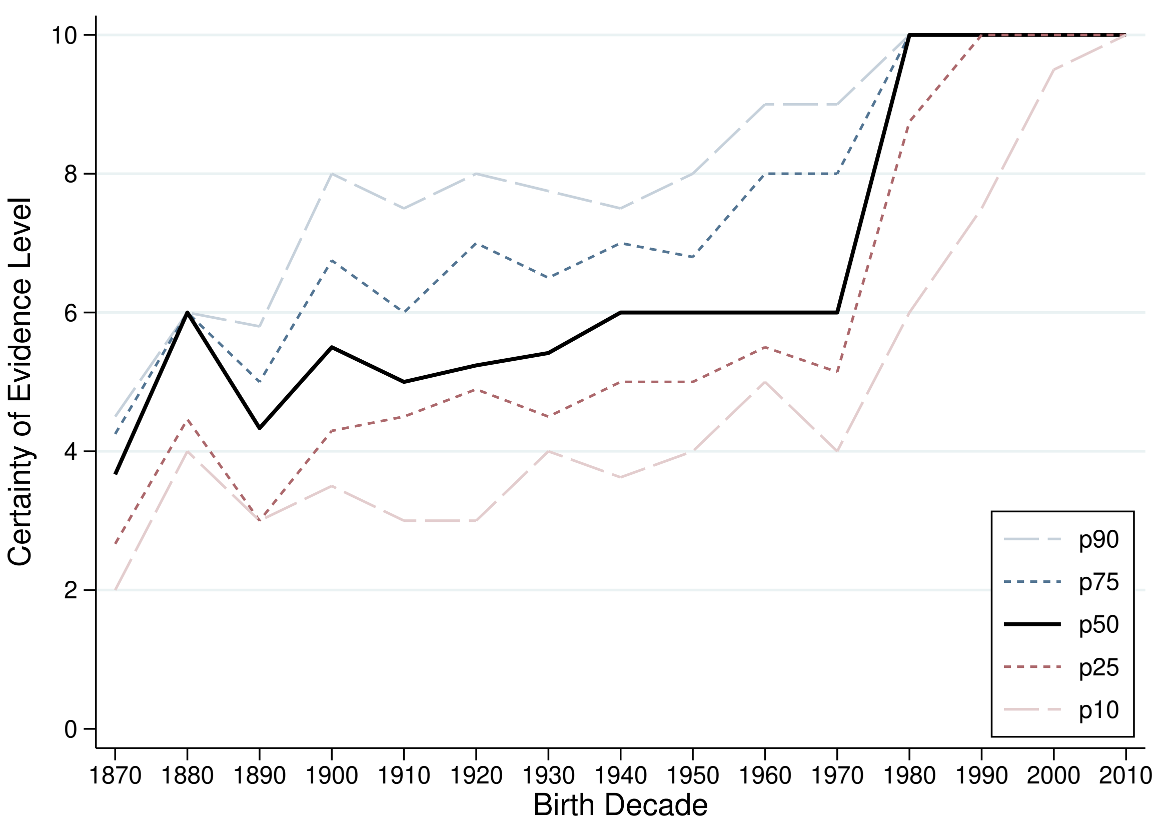

Figure I.3: Distribution of Mean Certainty of Evidence Scores by Country over Time

Notes: Distribution of country mean certainty of evidence scores over time, **including** recent studies that are covered in the JME dataset. Certainty levels are determined based on our adaptation of the GRADE system (see Table I.1.)

Sources: Worldwide Historical Stunting Dataset and UNICEF/WHO/World Bank (2023).

J Data Sources and Stunting Estimates

The sources for all studies in the Worldwide Historical Stunting Database are provided in the Excel file entitled 'WHS Final Data and Bib.xlsx'. These include studies subject to all inclusion and exclusion criteria. In addition, we have provided the final stunting estimate, the sample size and the certainty evidence information for each study so that researchers can scrutinise individual stunting estimates in the future. This file will be published in an open-access repository, e.g. open-ICPSR or Zenodo, after the paper is accepted for publication.

The documentation below serves as a Readme file for the dataset to give the reader a sense of the information included.

J.1 Variable Definitions

Study Characteristics

- country - Country by current international borders
- country_code - Three-letter country code
- dataset - Unique study id code (note that some studies contain more than one stunting estimate because, for instance, they report child heights in more than one year)
- b_year - Average birth year of children in the study
- stunting_rate - Stunting rate for the study. See Appendixes D, E, F and G for detailed description of the calculation of the stunting rate.
- height_n - Sample size of the study used to compute the stunting rate.

Certainty of Evidence Computations: see Appendix I for full details.

- ce_hetero_mean - points deducted because of hererogeneity of mean heights across different ages

- ce_indirect_sex - points deducted for only observing the height of one sex
- ce_indirect_age - points deducted for using observations of height above the age of five
- ce_indirect_space - points deducted for non-nationally representative study
- ce_imprecise_n - points deducted for small sample sizes of the study
- ce_imprecise_nosd - points deducted for estimating a stunting rate with mean height data only
- ce_overall - Overall certainty of evidence score, which is equal to 10 plus the deductions of the sub-component certainty of evidence scores.

Study Bibliographic Details

- typeofstudy - Whether the study is an article, book, official report or archival source.
- author - Author(s) of study
- title - Title of study
- p_year - Publication year of the study
- journal - Journal where study was published
- volume - Volume number of study
- number - Issue number of study
- pages - Page numbers of the study
- publisher - Publisher of study (if book or official report)

K Computing Country by Birth Decade Stunting Rates

K.1 Problems with Aggregating Studies with Disparate Methodologies and Sampling Procedures

One of the fundamental difficulties with meta-analyses of this type is that the underlying studies are extremely heterogeneous. The targeted population of a study may have been as small as Habakkuk (1926)'s study of children living in Barry, Wales or as large as the population of schoolchildren as in the data collected by the Japanese Department for Education across the twentieth century (Schneider and Ogasawara, 2018; Schneider et al., 2021). Studies may focus exclusively on particular sub-national geographic units or exclusively sample urban or rural populations. Given the time period studied here, many studies in the late nineteenth and early twentieth centuries were conducted before modern random sampling techniques had been developed. This means that from early historical periods, there are relatively few nationally representative surveys from which to compute national stunting rates. Instead, we are forced to extrapolate from studies that are not nationally representative.

There are several methods for managing this problem. Bayesian Hierarchical Modelling is currently popular among global health epidemiologists and has been employed to study child stunting going back to the 1980s as well as the historical changes in adult stature between the 1890s and 1990s (Finucane et al., 2015; NCD Risk Factor Collaboration, 2016; Stevens et al., 2012). Bayesian Hierarchical Modelling is an excellent tool for estimating global trends in health indicators over time, but we do not use it in our study for three reasons.

First, the purpose of this article is not to estimate global trends in stunting back to the late nineteenth century. While this might be interesting, we are sceptical that this is possible given the scarcity of data for many countries before 1950. Estimating global stunting rates before that date would require vast amounts of out-of-sample prediction

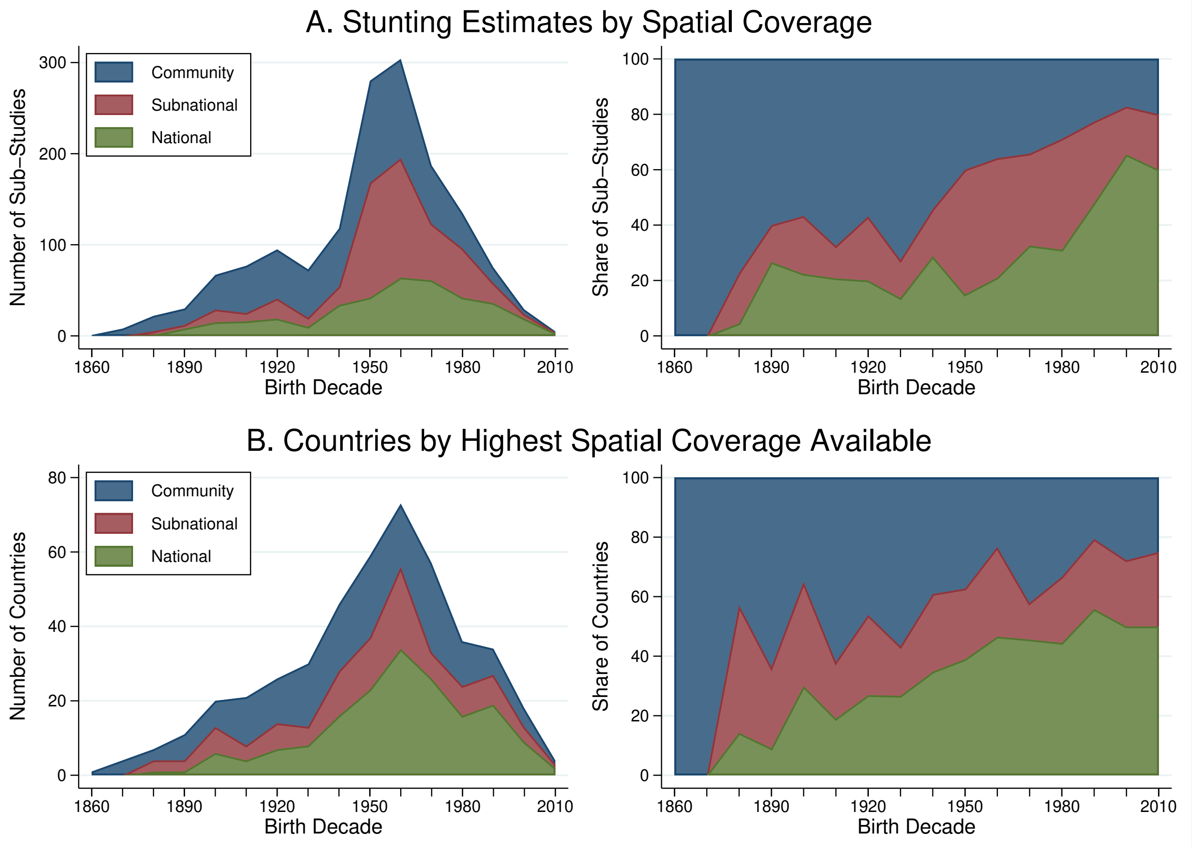

Figure K.1: Spatial Coverage of the Studies in the Meta-analysis

Notes: Studies found through the meta-analysis, **excluding** recent studies that are covered in the JME dataset.

Sources: Worldwide Historical Stunting Dataset.

that we do not feel could be justified. In addition, the balance of survey types is also different from other meta-analyses using Bayesian Hierarchical Modelling. As shown in Figure K.1, nationally representative studies only surpass 50% of total stunting estimates for the birth decades 2000 and later. Most of our data is based on community and sub-national studies, which make extrapolation using Bayesian Hierarchical Modelling more difficult, i.e. there are fewer national representative studies that we can use to define the parameters in the model.

Second, Bayesian Hierarchical Models require (somewhat) stable hierarchies to account for the heterogeneity in the studies. For instance, urban-rural gaps in height need to be stable or at least be changing in a consistent way. Alternatively, stunting estimates for ethnic groups would need to be stable across the period we study. However, the longer the time period analysed, the more unlikely it is that these hierachies are stable. Appendix L

presents data for Kenya that shows that the relative position of different ethnic groups in the height/stunting distribution has changed dramatically over time. In addition, the urban-rural differences in health have changed over time from a rural advantage by many metrics in the nineteenth century to a urban advantage in the mid twentieth century (Szreter and Mooney, 1998). However, these straightforward relationships between urban areas and height were not present in Spain and were also heterogenous within Spain with some areas experiencing urban height advantages rather than penalties and other regions experiencing little difference in height between rural and urban areas (Martínez-Carrión and Moreno-Lázaro, 2007; Ramon-Muñoz and Ramon-Muñoz, 2024). Unstable hierarchies that change over time make Bayesian Hierarchical Modelling unreliable.

Third, the purpose of our study is to highlight the levels of child stunting in the past and show the trajectory of change over time. Thus, we are more interested in highlighting the heterogeneity of stunting across countries rather than predicting a global stunting rate. However, readers should keep in mind that the certainty of evidence improves dramatically for our studies over time, which means that not all stunting estimates should be treated as equally reliable (see Figures I.2 and I.3).

K.2 Our Method for Aggregating Studies

There were two steps to aggregating stunting estimates and studies to create the final dataset. In the first step, we sought to aggregate stunting estimates within a study to make the study as representative of as large a population as possible. For instance, our earliest study of Sri Lankan children (Nichols, 1936) reports the mean heights of boys and girls in different types of schools in Sri Lanka. These schools included the Royal Preparatory Schools, Secondary Schools and Vernacular Schools. By finding a report on education in Sri Lanka in the 1930s, we were able to weight the stunting rates of each school type by the share of children in that type of school and come up with a single stunting estimate for the study. This mattered since 88% of Sri Lankan children were in Vernacular Schools whereas only 11% attended secondary schools and 1% the Royal Preparatory School. In this case, we were able to weight to a nationally representative

sample, but this was not always the case. In a large-scale study conducted by the Indian Council of Medical Research (Indian Council of Medical Research, 1972), not all Indian states were sampled. Still, we weighted the state-level stunting rate estimates by state population so that small states such as Delhi with an urban population of 3.6 million in 1971 would not be counted equally as large states such as Uttar Pradesh with a population of 88.3 million. In the end, the sampled states closely reflect the stunting rate in all states when we can compare with DHS survey from the 1990s onward (see Appendix L.3).

Note that when aggregating stunting estimates with both means and standard deviations, we follow the procedure of creating a joint distribution of HAZ scores as described in Appendix G.1, i.e. Equation 6. When we only have the mean heights of children, we impute the standard deviations to compute a stunting rate for each estimate and then take a weighted average of the stunting rates using population weights to aggregate the estimates to the study level. Not all estimates could be aggregated though as some reflected measurements taken at different points in time but reported together. Finally, we computed a single stunting rate for each study by averaging the stunting rate for girls and boys with equal weighting.[8]

To summarise the analysis to this point, we have taken data from individual studies, computed stunting rates for each study using the methods described above, aggregated stunting estimates where it was possible to find weights for subgroups within the studies and combined study-level estimates for boys and girls. This leaves us with a graph like Figure K.2 for each country (see Appendix O). As can be seen in the case of the United Kingdom, there are often studies with heterogeneous stunting estimates near one another, which would make creating a stunting rate trend by simply connecting the studies rather problematic. Instead, we take the average of the studies by decade based on the mean birth year of children in the study. In taking these averages, we do not attempt to find further weights that would allow us to reach some kind of national average within each decade. In most cases this would not be possible because the studies reflected in the decadal average would not cover an entire country.

[8] We plan to look at sex differences in child stunting in subsequent work.

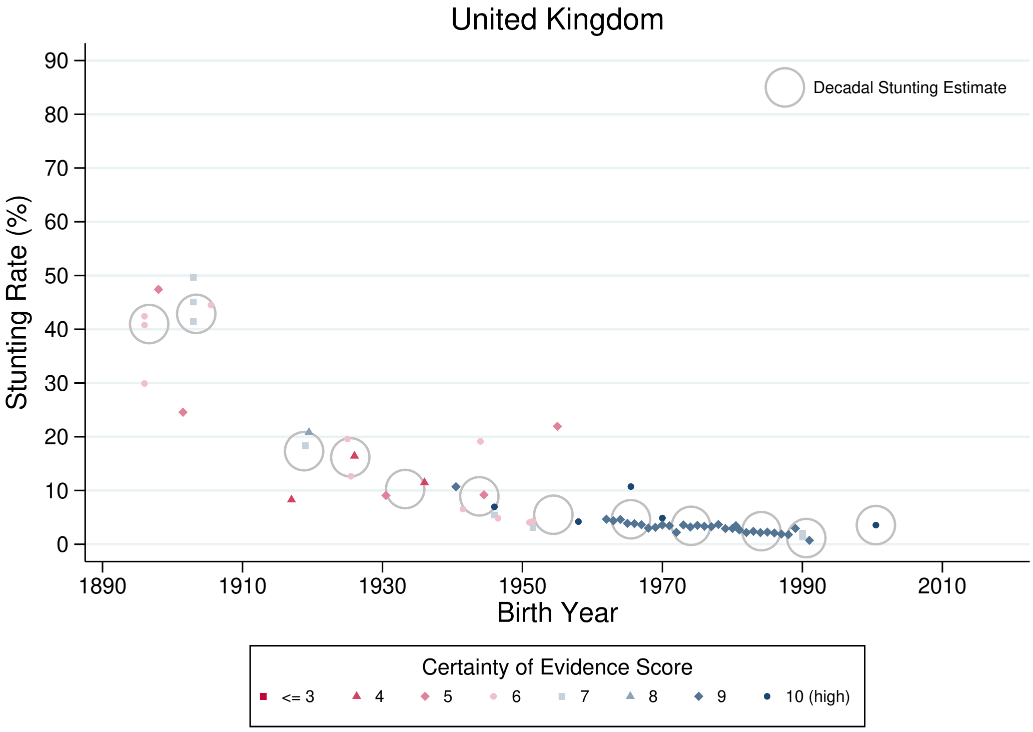

Figure K.2: Study-level Stunting Rate Estimates for Britain

Sources: Worldwide Historical Stunting Dataset and UNICEF/WHO/World Bank (2023).

However, we do deploy the certainty of evidence scores discussed in Appendix I to weight estimates when taking decadal averages. This ensures that small local studies with low certainty of evidence are not weighted equally with large, nationally representative studies, whose study subjects were born in the same decade. Table K.1 displays the weights applied based on the study-level certainty of evidence score. These weights are somewhat arbitrary, but again ensure that several small community studies do not have a stronger influence on the decadal average that a large nationally-representative study. In Figure K.2, the large grey circles depict the decadal averages that are plotted in Figure 3 in the main text. These are plotted for all countries in Appendix O so that readers can scrutinise the trend for each country and revise the data in the future if new studies are discovered or reconstructed from archival sources.

Table K.1: Certainty of Evidence Score-Based Weights for Computing Decadal Average Stunting Rates

Certainty of Evidence Score	Weight
9-10	5
6-8	2
$\leq$5	1

L Assessing Sources of Error in the Studies

As is apparent in Figure 3 in the main text, a number of countries experience inverse-U-shaped trends in stunting over the twentieth century. Stunting starts at a relatively low level, growing to the mid-twentieth century and then falling somewhat after that. This inverse-U-shaped pattern has also been found when analysing information on female adult stature in Africa from the DHS (Moradi, 2010; NCD Risk Factor Collaboration, 2016), so it is not entirely surprising. However, the scale of the reversals of fortune and the low levels of stunting in the early twentieth century, especially in Africa, are more difficult to believe. This appendix explores potential issues with the data that may explain these surprising patterns focussing on four case study countries: Democratic Republic of the Congo, Kenya, India and South Africa. Democratic Republic of Congo reflects how the locations and populations that Europeans measured (and published studies about) tended to be those most engaged with the development process, and therefore are likely to have been substantially positively selected relative to other locations. Kenya shows how the focus of early studies on specific ethnic groups can create large swings in the stunting rate estimates, especially where there is a lot of heterogeneity in stunting across ethnic groups. India shows how the ordinal ranking of stunting rates by state changes across the second half of the twentieth century. Finally, South Africa explores the importance of inequalities across racial groups in determining the stunting trend. Together these case studies emphasise some of the weaknesses with the systematic review methodology, especially when applied to historical studies based on non-representative samples. However, they also highlight the limitations of attempting to model away these problems given that, for instance, the relative position of ethnic groups in the stunting distribution has changed over time. In the end, they caution against taking the dataset and running country-level panel regressions without a careful study of the trends in each country.

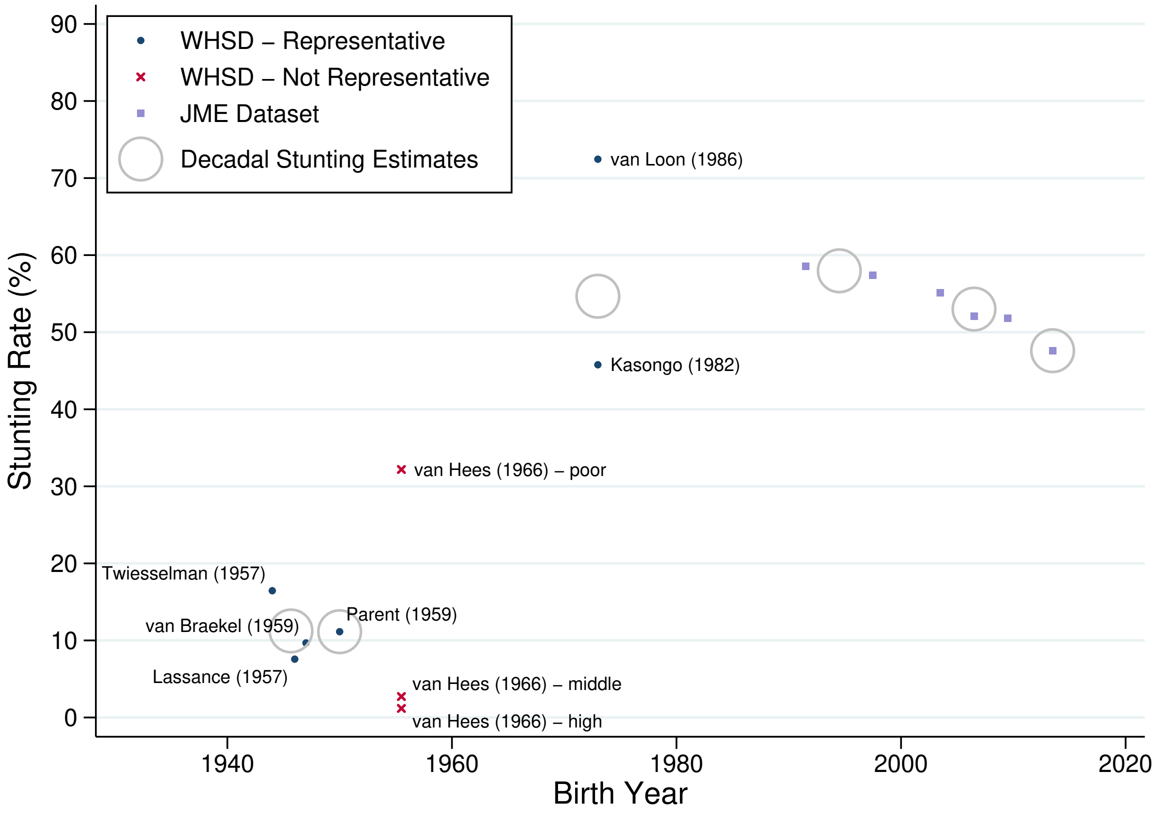

Figure L.1: Stunting rates by study for the Democratic Republic of Congo

Notes: WHSD is the Worldwide Historical Stunting Dataset; JME is the Joint Malnutrition Estimates from Unicef, WHO, and the World Bank.

Sources: Worldwide Historical Stunting Dataset. See Table L.1.

L.1 Democratic Republic of Congo

As seen in Figure 3Y in the main text, the Democratic Republic of Congo experiences one of the most dramatic reversal of fortune with regard to stunting with the stunting rate rising from 11% in the 1940s and 1950s to 55% in the 1970s and then gradually falling to 42% in the 2010s. The low stunting rate figure of 11% is striking and honestly implausible given our understanding of the history of population health in the Democratic Republic of Congo (Almquist, 1994, pp. 126-31). Thus, it is worthwhile taking time to analyse the studies underpinning these decadal averages in detail and to try to understand why the stunting rate could be estimated to be so low.

Figure L.1 and Table L.1 present the studies in detail. There are actually four studies underpinning our low estimates of the child stunting rate in the 1940s and 1950s. These

Table L.1: Study Information for the Democratic Republic of Congo

Study	Mean Birth Year	Stunting Rate (%)	Representative	Location	Coverage	Urban/Rural	Children in Study
Twiesselman (1957)	1944	16.4	Yes	Kinshasa (Leopoldville)	Community	Urban	679
Lassance et al. (1957)	1946	7.6	Yes	Kisangani (Stanleyville)	Community	Urban	300
van Braekel (1959)	1947	9.7	Yes	Lumumbashi (Elizabethville)	Community	Urban	395
Parent (1959)	1950	11.1	Yes	Katanga (Southeast Region)	Subnational	Not clear	1,357
Van Hees et al. (1966) - high	1956	1.2	No	Kinshasa (Leopoldville)	Community	Urban	279
Van Hees et al. (1966) - middle	1956	2.7	No	Kinshasa (Leopoldville)	Community	Urban	463
Van Hees et al. (1966) - poor	1956	32.0	No	Kinshasa (Leopoldville)	Community	Urban	284
Kasongo Project Team (1982)	1973	45.7	Yes	Kasongo (Centre-East Region)	Community	Rural	7,092
van Loon et al. (1986)	1973	72.7	Yes	Kalima (Centre-East Region)	Subnational	Rural	667

Notes: Stunting rates computed following the procedure described above.

Sources: Citations for studies listed in the reference list below.

studies were published in the late 1950s, but reflected children born in the mid to late 1940s and 1950. Three of them were studies of the most important urban centres in the then Belgian colony: Kinshasa (Leopoldville) in the west, Kasangani (Stanleyville) in the northeast and Lumumbashi (Elizabethville) in the southeast (see Figure L.2). The fourth study focussed on workers employed by the main mining company in the southeastern region of Katanga.

The close similarity in the stunting rates across these vast areas of space lends credibility to these estimates, and the authors themselves were sanguine about the representativeness of their data. Lassance et al. (1957, p. 638, translated from French) writes that 'the scales of school biometrics established in the C.E.C. of Stanleyville are probably applicable to the whole of the Congo, at least among the Congolese of the Bantu race living in conditions of life comparable to those of Stanleyville'. Both Lassance et al. (1957) and Parent (1959) claim that the children measured in their studies were representative of the wider children in their target population. Thus, we code them as being representative studies since they were representative of the target population. Note also that most authors compared the Congolese children with Belgian and American children and argued that their growth was similar, so it is not surprising to find low stunting rates in these populations.

However, just how representative these populations were of the wider country is questionable. The three cities listed above made up 8.7% of the population in 1970, and Almquist (1994) argues that cities grew approximately ten-fold between 1940 and 1970. If we approximate the share of these cities in the total population by dividing their 1970 population by 10 and then computing their joint share of the 1940 population, these cities were only 1.8% of the population. Thus, they were a tiny share of the total population.

In addition, by sampling children in urban areas where Europeans were located, the studies sampled mostly individuals who were engaged in the urban, formal economy. Some studies mention that the sample included children of *évolués*, the class of clerks, nurses and teachers, that interacted most with the colonial authorities and also became the new elite after independence (Almquist, 1994, p. 97). Thus, there may have been positive

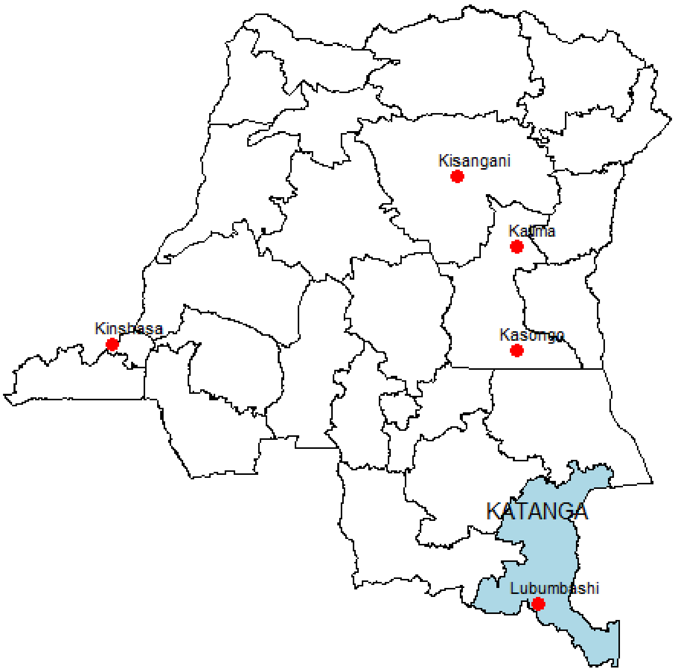

Figure L.2: Study locations for the Democratic Republic of Congo

selection into even the urban samples in spite of the claims made by some authors. We see hints of this if we compare the stunting estimate of Twiesselman (1957) for Kinshasa children with an average birth year of 1944 with the non-representative study by Van Hees et al. (1966) of Kinshasa children with an average birth year of 1956. Van Hees shows large differences in stunting rates between the poor and middle and high class groups, and given that the poor would have outnumbered these other groups, Twiesselman (1957)'s stunting rate seems to be a bit too low and may be positively selected on socioeconomic status.

Urban children would also have had greater access to missionary schooling and hospitals, and there is evidence in other parts of Africa, that missionaries tended to settle in more productive and healthy areas (Jedwab et al., 2022). This may have been less true of Kinshasa and Kisangani, which are primarily located for their importance in trade along the Congo River. Kinshasa is located just above a series of falls on the Congo river, and Kisangani is the farthest navigable point on the Congo River. However, this explanation may be more important for other cities in the Democratic Republic of Congo and also for other countries in Asia and sub-Saharan Africa that experienced colonialism.

Finally, these studies were conducted in the postwar period when the colonial government was being threatened by the independence movement: independence came in 1960. During this period, the colonial state and colonial businesses increased wages and expanded social services for the Congolese people (Smith et al., 1994, pp. 140-41). While these efforts were limited, one can imagine that they were most effective in places where the colonial government had a presence. Parent (1959) lists a number of reasons that children of company employees in the Katanga region would have had relatively good growth: 'Salaries are good. Food is not scarce and, over the years, the public market has been enriched with many products of high nutritional value (protein in particular). In addition, until 1955 these families all received a ration in kind adapted to the composition of the family.... Food supplements are regularly distributed to children, especially skimmed milk, on average 200 cm^3 per school day' (Parent, 1959, p. 40, translated from French).

When rural studies enter our sample in the 1970s, they show much higher stunting rates which are in-line with the JME estimates from the 1990s onward. While there was certainly turmoil in the transition to independence (Lemarchand, 1994), there are few structural reasons to believe that child child stunting rate deteriorated by 40 percentage points between the 1950s and 1970s.

Given that the areas sampled by Europeans were restricted to cities with European presence and children in these cities may also have been positively selected, we must have a large degree of scepticism about what these studies tell us about child stunting in the Democratic Republic of Congo in the 1940s and 1950s. This begs the question, why not simply exclude them from the analysis then. The problem is balancing a close historical analysis on data quality of individual studies and their context with universal inclusion and exclusion criteria across all studies. In the case of Democratic Republic of Congo, these studies should probably be excluded, but in other countries, small community surveys may be more informative. In many cases, it is not possible to conduct as thorough an analysis as what has occurred here, so we err on the side of including these studies. We also do not want to be accused of cherry-picking data that fit our priors

about what stunting rates should have been in various countries at given points in time. While all studies meeting the inclusion and exclusion criteria have been included in our meta-analysis, we do, however, focus our interpretations in the main text on countries and trends that we are confident are correct.

L.2 Kenya

Kenya is another country experiencing an inverse U-shaped path of stunting across the twentieth century. This pattern does not track what previous studies on adult stature have found where mean adult heights are generally observed increasing for much of the twentieth century (Moradi, 2009; NCD Risk Factor Collaboration, 2016). However, Moradi (2009) found considerable variation in mean adult male stature across ethnic groups. Table L.2A reports the findings of Moradi (2009) and extends them to other ethnic groups in order to make sense of the studies in our sample for Kenya. Table L.2B reports trends in female stature which help to fill the time gap between the male stature records and stunting estimates. There were striking differences in height across ethnic groups historically: in 1880, the mean male adult height of the smallest ethnic group, the Digo, was nearly 9 cm shorter than the mean height of the tallest, the Luo. Thus, historical studies that are only representative of one ethnic group may give a very distorted perspective on stunting trends in Kenya over time.

Complicating things even more, the rank order of ethnic groups by nutritional status has changed substantially over time. In the late nineteenth and early twentieth centuries, the Kikuyu ethnic group was one of the shortest in the population, but by 2014, they had the lowest stunting rate of the major ethnic groups. In contrast, in the early twentieth century, the Kalenjin ethnic group was one of the tallest ethnic groups, but by 2014, they had one of the highest stunting rates among the larger ethnic groups, experiencing an extreme relative decline compared to other groups. Thus, the problem is not simply that historical studies may focus on only one ethnic group, it is also very difficult to extrapolate from one ethnic group to the rest of the population.

Looking now at the Kenyan studies in detail, Table L.3 and Figure L.3A present

Table L.2: Mean adult stature of different ethnic groups in Kenya from the late nineteenth century to the 1980s

A. Male Adult Stature

Birth Year<br>Source	c. 1880<br>(Leys and Joyce, 1913)		1902-6<br>(Orr and Gilks, 1931)		c. 1915<br>(Moradi, 2009)		1962 Census<br>(Moradi, 2009)
Ethnic Group	Mean adult height (males)	N	Mean adult height (males)	N	Mean adult height (males)	N	Population Share (%)
Mijikenda (Digo)	162.9	15			-	-	4.8
Meru	-	-			164.0	341	5.1
Kikuyu	164.0	384	164.8	423	165.4	127	19.1
Akamba (Kamba)	165.7	128			167.9	139	10.8
Luhya	169.3	24			169.2	113	12.6
Kalenjin	169.5	49			168.4	52	10.4
Luo	171.8	37			171.9	434	13.3

B. Female Adult Stature

Birth Year<br>Source	1902-6<br>(Orr and Gilks, 1931)		1950s<br>(DHS 1993)		1960s<br>(DHS 1993)		1970s<br>(DHS 2003)		1980s<br>(DHS 2014)	
Ethnic Group	Mean adult height (females)	N	Mean adult height (females)	N	Mean adult height (females)	N	Mean adult height (females)	N	Mean adult height (females)	N
Mijikenda (Digo)			155.1	92	156.3	199	156.7	224	156.4	256
Meru			157.0	93	158.0	180	160.0	108	158.2	187
Kikuyu	152.9	441	157.0	159	157.9	489	159.3	509	159.6	629
Akamba (Kamba)			156.2	113	157.2	339	157.5	288	156.8	431
Luhya			161.4	263	160.9	538	161.5	490	160.3	586
Kalenjin			160.9	181	160.8	521	160.3	311	160.6	822
Luo			161.6	153	162.2	441	163.3	301	162.0	506

Notes: Mean female stature by ethnic group were computed using the sampling weights in each DHS survey. Birth decades in the DHS survey run from 1950 to 1959, and this determines the age groups captured.

Sources: Listed in table, see reference list.

the studies that we have contributed in relation to the data in the JME dataset. We were able to include five studies and four of them were of specific ethnic groups. The earliest study, Orr and Gilks (1931) focussed specifically on the Kikuya ethnic group and shows a relatively low stunting rate at 20.8%. This is surprising given that the Kikuya were among the shorter ethnic groups in the late nineteenth and early twentieth century (see Table L.2). Thus, if extrapolating from this study, we would expect a nationally representative stunting rate to be lower than 33.1%. The next study for children born twenty years later (MacKay and Martin, 1952) focussed solely on the Digo ethnic group and shows a much higher stunting rate of 56.0%. The Digo were a part of the larger Mijikenda ethnic group, which was one of the shortest groups both in the 1880s and in 2014. Thus, it is fair to assume that this stunting estimate is probably too high and a nationally representative rate would be lower. The next three studies are closer in time to when the JME dataset begins for Kenya and are much closer to the JME estimates for the 1970s. Jansen (1984) focusses solely on the Akamba ethnic group, and Moradi (2009) shows that by 1915 they were more or less at the average for the population of Kenya. Hoorweg et al. (1983) focus on the Kikuyu again, but by this point, the stunting rate for the Kikuyu was close to the national average, reflecting the movement of the Kikuyu from one of the more deprived groups to one of the healthiest according to the 2014 DHS (Table L.2.)

Table L.3: Stunting rate estimates for Kenya from studies included in our sample

Short Reference	Birth year	Stunting rate (%)	Ethnic group
Orr and Gilks (1931)	1923	33.1	Kikuyu
MacKay and Martin (1952)	1944	56 .0	Digo
Jansen (1984)	1969	43.8	Akamba
Central Bureau of Statistics (1979)	1974	42.9	All
Hoorweg et al. (1983)	1975	41.4	Kikuyu

Notes: Stunting rates computed following the procedure described above.

Sources: Worldwide Historical Stunting Dataset.

Given the preceding discussion, the ethnic group-specific studies match expectations except for the earliest one by Orr and Gilks (1931), which shows a surprisingly low stunting rate. An increase in the stunting rate between the Orr and Gilks (1931) and

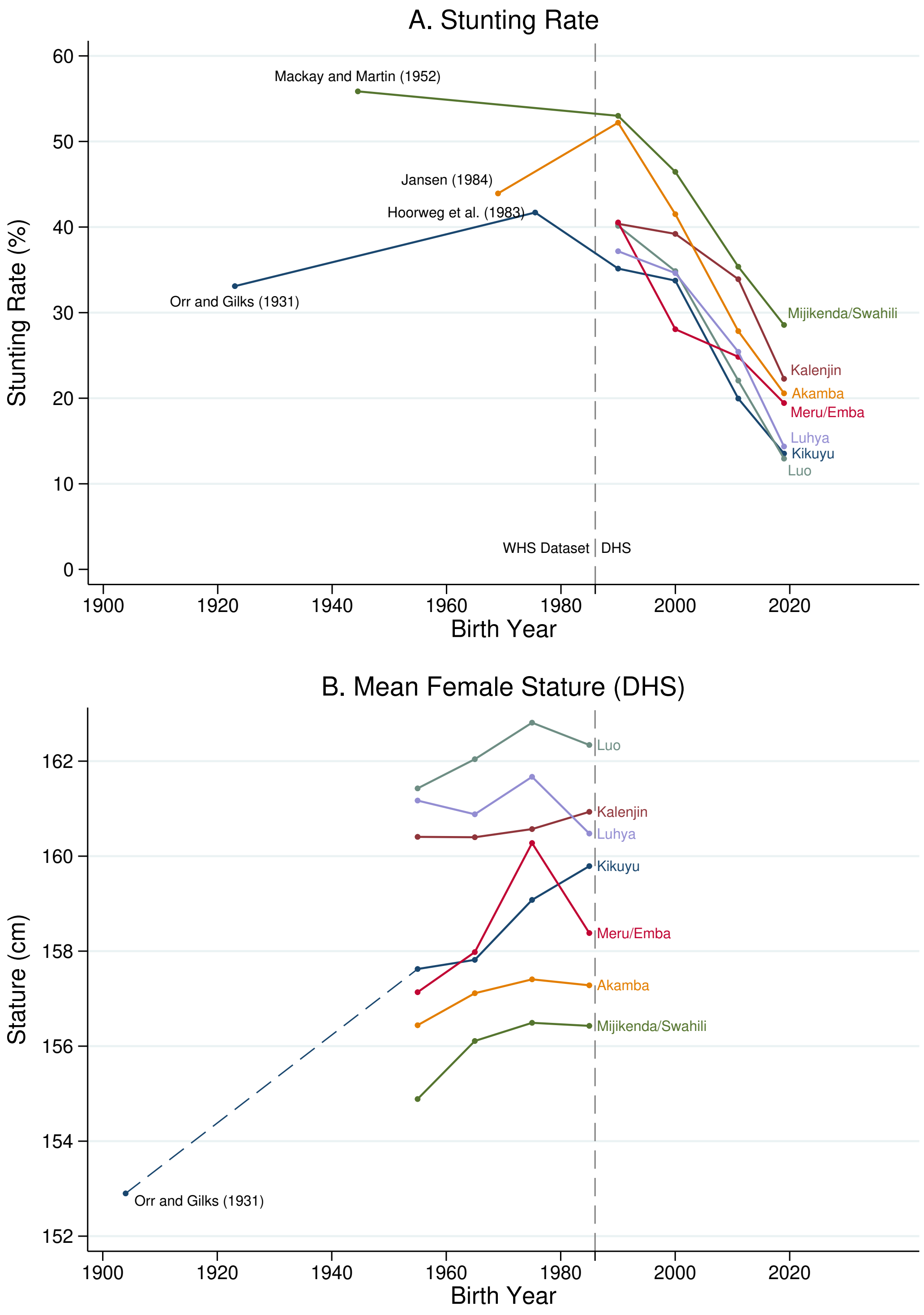

Figure L.3: Kenyan studies over time

Notes: In panel A, the studies to the left of the dashed vertical line are drawn from the Worldwide Historical Stunting Dataset and are computed following the procedures described above. These studies are described in Table L.3. Ethnic group stunting estimates in panel A to the right of the dashed vertical line are from the 1993 and 2014 Demographic and Health Surveys for Kenya and are computed using the sample weights. In panel B, the mean female stature is displayed by ethnic group as in Table L.2. The data come from the 1993, 2003 and 2014 DHS for Kenya again applying standard survey weights.

Sources: Worldwide Historical Stunting Dataset; Kenya Demographic and Health Surveys in 1993, 2003 and 2014.

Hoorweg et al. (1983) studies suggests that mean height should have been falling whereas the trend in female adult stature from Orr and Gilks (1931) to women born in the 1950s and 1960s observed in the 1993 DHS is a significant increase in stature (Figure L.3). Thus, it is worth looking at the Orr and Gilks (1931) study in more detail. Figure L.4 shows that mean HAZ scores for children in the Orr and Gilks study are relatively high close to age five and decline with age afterwards. The sample size is particularly small at the earlier ages with the number of individuals for each age group and sex only reaching 40 for age 8 and greater. When comparing the pre-11 mean HAZ scores with the HAZ scores for adults observed in the Orr and Gilks (1931) survey, the adults have much lower HAZ scores, and these are based on much larger sample sizes. The adult heights also tend to match the pre-11 means for the Hoorweg et al. (1983) study. Thus, it appears that there is selection on unobservables for the younger children in the Orr and Gilks (1931) survey, which is creating bias in the stunting estimate. It is difficult to say exactly what this bias might be: perhaps there is systematic measurement error in the children's ages for instance. However, it does raise suspicion about the Orr and Gilks (1931) stunting estimate.

This example also highlights the limitations of the methodology used in this study. In the case of Kenya, it was possible to cross reference this specific study with a number of other studies and surveys distinguishing different ethnic groups, but this method could not be replicated for every country in the dataset, mostly because of lack of data. Thus, there is still important work to be conducted by local historians to improve upon the estimates we make here.

L.3 India

As a very large country that has a long history of high levels of child stunting, India's history of child stunting is particularly important to study. As with the historical data for most countries, the Indian studies in our dataset vary in geographical scope quite considerably. While we do include one study with very large sample sizes and national coverage (the Indian Medical Research Council Study), the remaining studies in our data-

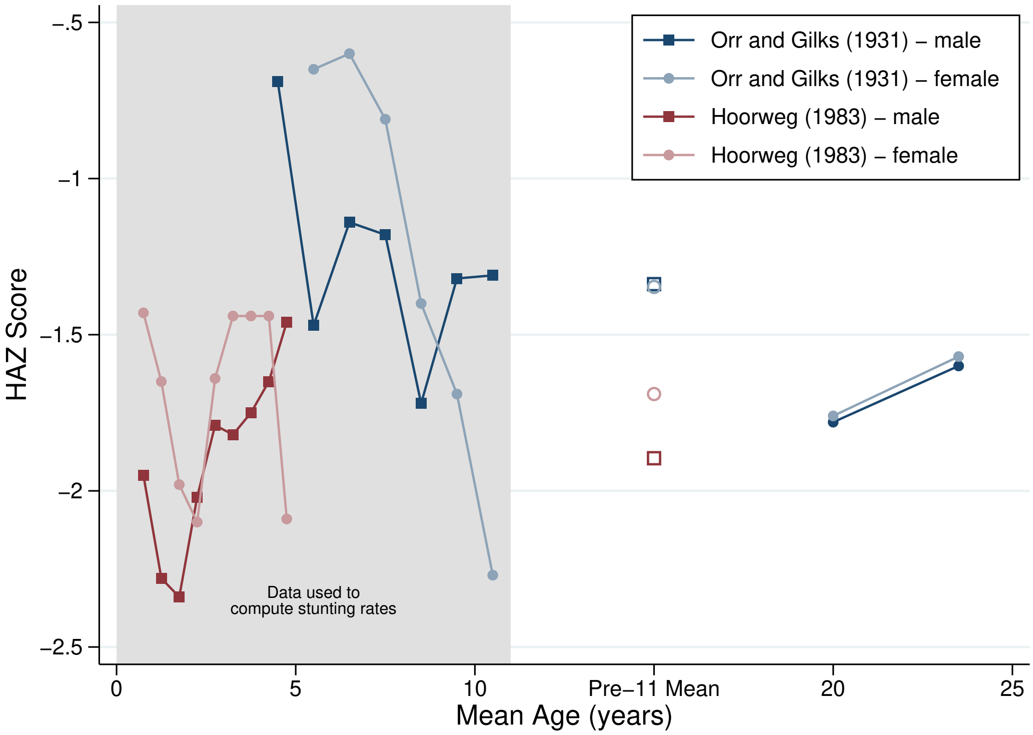

Figure L.4: Height-for-age Z-scores of Kikuyu children and young adults from two studies

Notes: Height-for-age Z-scores computed using the WHO child growth standard (WHO, 2006) and the WHO growth reference for school-aged children and adolescents (de Onis et al., 2007).

Sources: Orr and Gilks (1931); Hoorweg et al. (1983).

set before the earliest data from the JME come from more local or regional studies. This Appendix sub-section attempts to make sense of these varied studies by first focussing on the national studies available and then exploring the smaller scale studies and what they can tell us about stunting in India. It illustrates the potential limitations of extrapolating national patterns from regional data, especially in contexts marked by significant regional disparities that are then translated into distinct trends in regional stunting rates.

L.3.1 Indian Council of Medical Research Study

Any long-run analysis of height trends in India must begin with the Indian Council of Medical Research (ICMR) study (Indian Council of Medical Research, 1972). This was the first large-scale study of child growth and was conducted in the early to mid 1960s across 11 states. The study measured 127,866 children aged 0 to 21, providing a very large sample from which to estimate population parameters. The states included in the sample were Andhra Pradesh, Delhi (urban only), Jammu & Kashmir, Kerala, Madha Pradesh, Madras, Maharashtra, Orissa/Odisha (urban only), Punjab, Rajasthan and Uttar Pradesh. These states are drawn from across India aside from the Northeastern region and represent 53.0% of the population. The ICMR study was conducted in both urban and rural areas, but it oversampled from urban areas: 65.8% of children in the sample were drawn from urban areas whereas only 19.9% of the population of India was urban according to the 1971 census. The ICMR used different sampling strategies in urban and rural areas. It is worth discussing these sampling strategies at length.

Rural areas: In rural areas, the ICMR study used 'a three-stage stratified design' to select households for the sample (Indian Council of Medical Research, 1972, p. 37). The sample frame was based on district census data and was stratified further into large and small villages. They sampled approximately one in 200 villages. 'All eligible children in the selected villages were included in the sample. Children were asked to assemble in the village school or some central place in the village for clinical examination and body measurements' (Indian Council of Medical Research, 1972, p. 4). The report suggests that 'past experience had indicated that the response rate among children was about 20 per

cent of the population in a village' (Indian Council of Medical Research, 1972, p. 37).

Urban areas: In urban areas, the ICMR study conducted stratified sampling based on city size, but the sample frame was created from a list of schools rather than from district census data. 'This method was resorted to because of ease of availability and moreover, schools could also be stratified broadly according to socio-economic status of their alumni as they cater to different sections of the society selectively' (Indian Council of Medical Research, 1972, p. 38). The discussion of how the schools were selected and how stratification by socioeconomic status occurred is rather confusing and unclear. The study was aware that sampling in schools would prevent them from studying children not of school age, so they adopted the following procedures to capture these children:

> The children aged under 5 years and some over 15 years who did not attend any school were also examined. In large cities this could be achieved with the help of public health authorities who instructed the persons concerned to attend the maternity and child health centres. The social organizations existing in the cities were asked to pursuade (*sic*) the well-to-do classes to send in their children for examination at such places as were convenient to them. An alternative procedure in towns where such organizations did not exist was to adopt purposive sampling. A list of areas predominantly inhabited by the lower, middle and upper socioeconomic classes was made and one block was selected from each and all the houses included in it were included in the sample (Indian Council of Medical Research, 1972, p. 38).

Clearly, the study designers were attempting to create the most representative sample possible, but the execution of the study and the analysis were flawed in many ways. Particularly troubling is the different sampling procedures for children of school age and outside school age in urban areas. There is also a concern that children who remained in school at older ages could be positively selected on socioeconomic status. This has been shown to bias the height-by-age profile in a number of historical contexts including the United States and Japan (Schneider, 2020).

To see whether any bias might be present, Figure L.5 plots the HAZ by age for the ICMR data with the blue line representing children whose exact age was verified during the study and the maroon line indicating the pattern for all children: the pattern for both lines are nearly identical, so measurement error in ages does not seem to bias mean HAZ scores in the data. The children follow the typical pattern of growth faltering in the first two years with mean HAZ scores falling from around zero to -3 at age 2.5. However, after

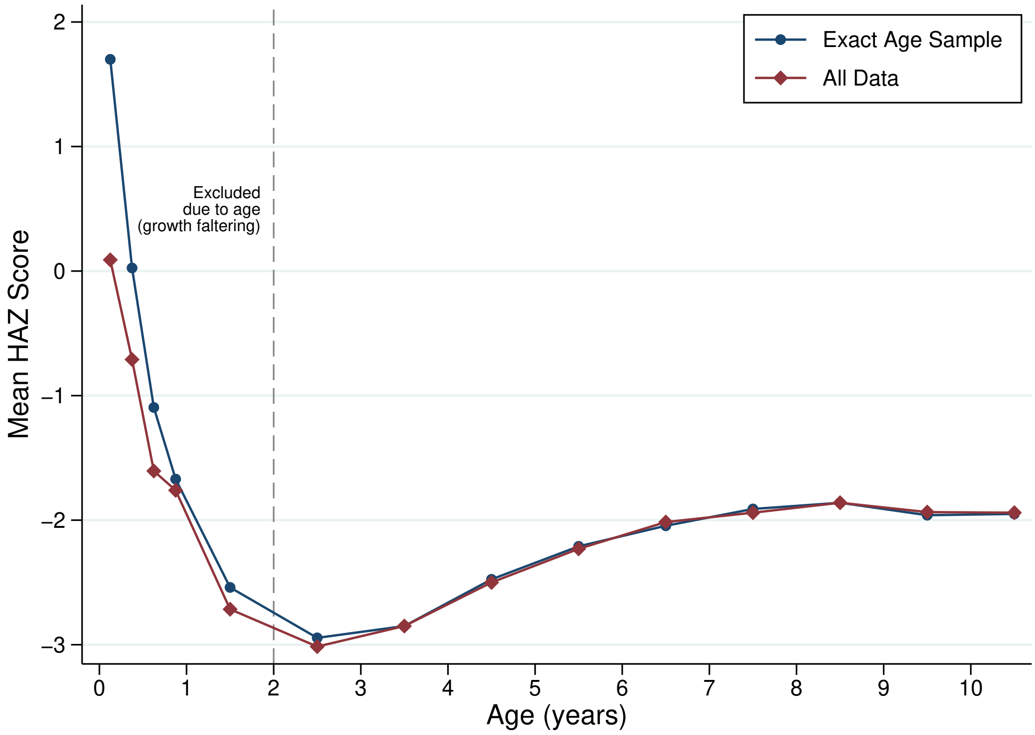

Figure L.5: HAZ-by-age profile for Indian children in the ICMR study

Notes: HAZ is height-for-age Z-score computed against the WHO 2006 growth standard and 2007 growth reference.

Sources: Indian Council of Medical Research (1972).

age 2.5, the children appear to experience catch-up growth climbing approximately 1 HAZ score point relative to healthy children. This catch-up growth is problematic because it violates our assumption that growth faltering occurs in the first two years and HAZ scores (and stunting rates) are mostly stable after age two.

The question, then, is what is driving this increase in HAZ scores between age two and mid-childhood. There are four potential possibilities, which will be discussed in turn: 1) selection on observable characteristics which change over age; 2) selection on unobservable characteristics from the study design; 3) selective culling of short children progressing with each age; and 4) real catch-up growth of Indian children.

Selection on observables: One of the biggest flaws of the ICMR study was that there was no attempt to reweight the individuals measured to make them representative of the country (or even the sampled states) as a whole in the final report. For instance, the overall heights by ages reported by the ICMR are the average of the rural and urban

samples weighting all sampled individuals equally in spite of the fact as mentioned above that the study heavily oversampled urban areas. While the ICMR report includes height-by-age summary tables for a number of characteristics including urban/rural, state, socioeconomic status and religion, unfortunately, crosstabs combining more than one of these categories are never provided, making it difficult to retroactively apply sample weights derived from the census. Still one can plot the HAZ-by-age profiles by these characteristics to get a sense of whether the pattern was common across all groups. Figure L.6 plots the HAZ-by-age profile for urban and rural children, for each of the 11 states, for children of different socioeconomic backgrounds and for different religions. The increase in HAZ between age two and mid-childhood is present in all subcategories. Thus, while the composition of the sample does change on observable characteristics to a small degree, the changing composition of the sample by age cannot explain the pattern.

Selection on unobservables: It is also possible that the catch-up growth observed is driven by selection on unobservables, i.e. even within the observed categories presented above, there is a shift toward healthier children between age two and mid-childhood. The strongest evidence that this may have occurred is the use of schools as sampling frames in urban areas. If children attending school were on average healthier than those not attending school, then even within the same religion and SES groups, there would be upward bias when shifting from populations measured at home to those being measured in schools. This seems to have been the case in urban Odisha where the mean HAZ score increased from -3.75 at age 2.5 to -1.01 at age 6.5 (Figure L.6B). Population-level catch-up growth of this magnitude in only a few years is biologically implausible, and the increase of 1.41 HAZ scores between ages 4.5 and 5.5 suggests that there were likely to be differences in the characteristics of children measured at home and in schools. However, only Odisha shows such a strong increase between ages 4.5 and 5.5. Most other states show continuous increases or sharp increases at other ages. Most puzzlingly, the pattern of catch-up growth is present in rural areas, which according to the ICMR report, never used schools as sampling frames. Thus, while there is some evidence to support selection on unobservables, the selection is not straightforwardly linked to the sampling procedure.

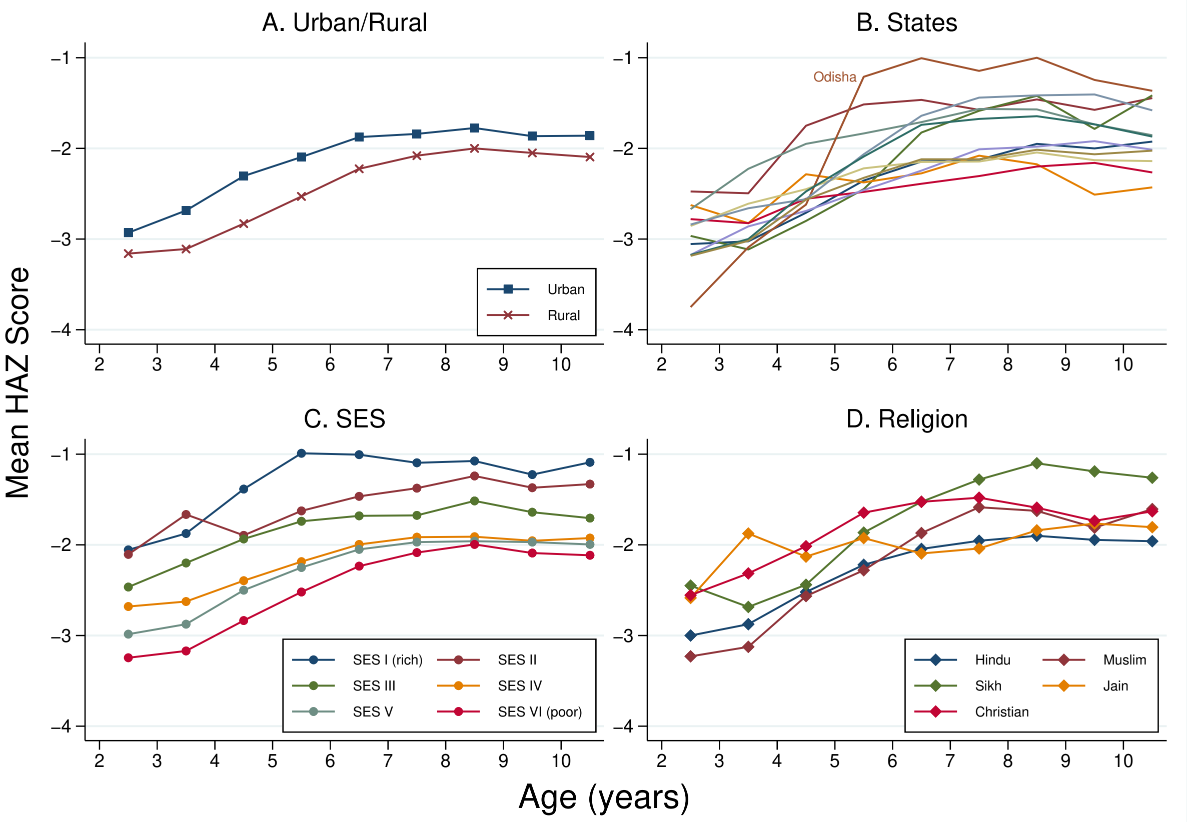

Figure L.6: HAZ-by-age profile for Indian children in the ICMR study by certain characteristics

Notes: HAZ is height-for-age Z-score computed against the WHO 2006 growth standard and 2007 growth reference.

Sources: Indian Council of Medical Research (1972).

Selective mortality: A third possibility is that the increasing mean HAZ scores by age are driven by the selective mortality of the shortest individuals, leaving taller survivors at each subsequent ages (Bozzoli et al., 2009; Deaton, 2007). However, this seems very unlikely. Mortality rates are relatively low between age two and mid-childhood, limiting the effects of survival bias at those ages. In addition, Alderman et al. (2011) show that mortality was not selective enough with respect to height in India to explain large increases in HAZ scores across age.

Real catch-up growth: The final possibility is that the catch-up growth around 1 HAZ score is real and reflects improving health conditions for Indian children between age two and mid-childhood. Prentice et al. (2013) found catch-up growth of this type in children in the Gambia and also among participants in longitudinal studies in Brazil, Guatemala, the Philippines and South Africa, but the scale of the catch-up was substantially lower than in the ICMR study with increases of 0.17, 0.63, 0.15 and 0.71 HAZ scores respectively. The longitudinal nature of the data helps assuage fears that selection of different children by age were driving these results. However, these findings do not necessarily mean that the catch-up growth observed in the ICMR is real. Prentice et al. (2013) found very little catch-up growth in the longitudinal study for India that they analysed. Likewise, Leroy et al. (2015) and Lundeen et al. (2014) argue that this catch-up is simply a product of the the dispersion of height increasing with age and that the difference between the mean height of children and the reference does not change.

In the end, the documentation for the ICMR study is not detailed enough to be able to determine whether the catch-up growth was real or driven by selection on unobservables, or perhaps a combination of both. Further archival work could perhaps uncover the individual-level data for the study or greater detail in how the study was executed across the states, but without this more detailed work, it is not possible to definitively confirm or reject selection on unobservables in the data.

To calculate a stunting rate from the ICMR study, we proceed as follows:

As mentioned above, because means and standard deviations of height are not reported by state and urban/rural status, we cannot simply apply census weights to reweight

the data. However, if we assume that the ratios of urban to rural heights by sex at each age were equal to the ratio in all states in the ICMR by sex at each age and that the urban and rural shares of the sample by sex at each age were equal to the shares in all states included in the ICMR by sex at each age, then it is possible to compute the mean heights of urban and rural children by sex at each age in each state. The assumption that the urban and rural shares would be the same is justified since the ICMR had specific targets for urban and rural samples at a ratio of 5 to 3. Whether urban-rural differences in height were the same across space is unknowable from the ICMR study, but it seems better to allow for differences between urban and rural areas given the oversampling in the ICMR of urban areas. Weighting for the differences in urban and rural areas across states in this way increases the stunting rate by 3.3 percentage points relative to weighting by state population alone.

The bigger issue is whether to include all ages in the stunting calculation, assuming the catch-up growth at later ages was real, or to simply include children under age five to facilitate comparisons with the DHS data that makes up the later nationally representative JME datasets. This makes a big difference in the stunting rate: the stunting rate for children aged 2 to 10.99 is 59.3% whereas the stunting rate for children aged 2 to 4.99 is 72.4%, a 13.1 percentage point difference. Again, there is no straightforward way to pick one of these versus the other. Therefore, we decided to follow our general inclusion and exclusion criteria and use the stunting rate for age 2 to 10.99.

A final worry about the ICMR study is that the set of states in the ICMR was not representative of India as a whole. To assess whether these states were representative, we use the more recent DHS data to compute stunting rates for all of India and also just the states that were included in the ICMR. Figure L.7 presents the results, which show that at least in the past twenty years, the stunting rate in the states included in the ICMR sample were were very similar to, if slightly below those for India as a whole. Thus, there is no reason to believe that differences in the composition of states included in the sample between the ICMR and DHS dataset biasing the comparisons.

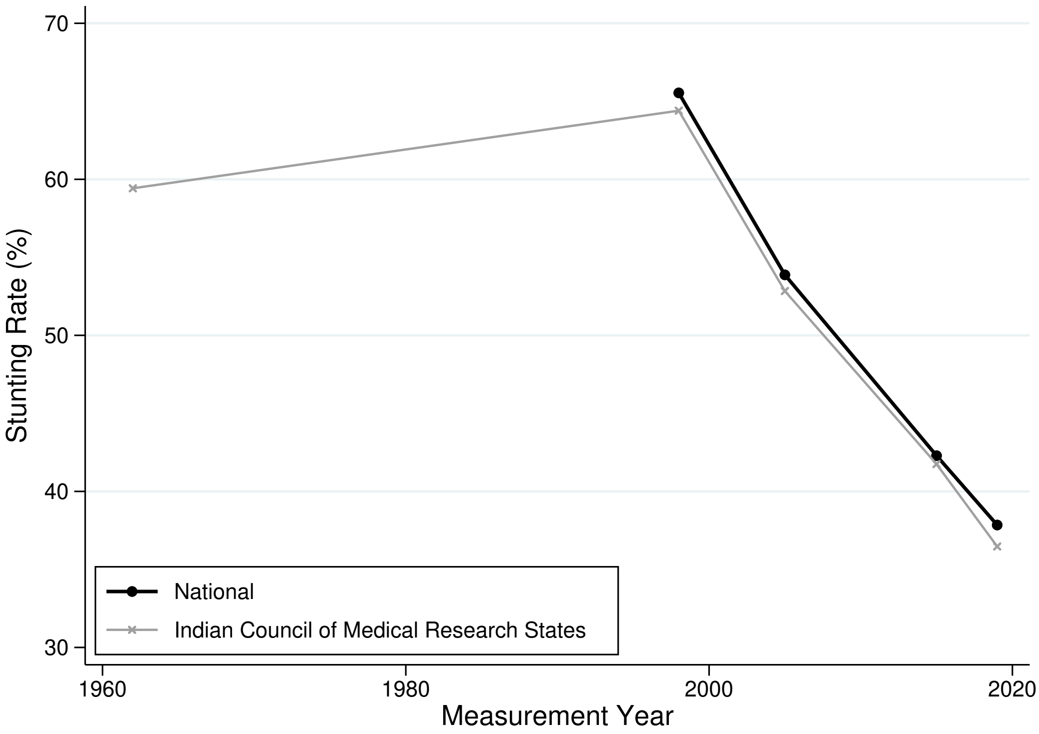

Figure L.7: Stunting Rates in states covered by the Indian Medical Research Council (IMCR) study compared with all states in later DHS studies

Notes: States covered by the ICMR study were Andhra Pradesh, Delhi, Jammu & Kashmir, Kerala, Madha Pradesh, Madras, Maharashtra, Orissa (Odisha), Punjab, Rajasthan and Uttar Pradesh. The ICMR states are weighted by their population in 1971. National sample weights are applied to the DHS stunting rates.

Sources: Indian Council of Medical Research (1972); India Demographic and Health Surveys in 1998-9, 2005, 2015 and 2019.

L.3.2 Stunting Trends Across States

When trying to contextualise smaller local studies, it is helpful to explore the state-level pattern of stunting over time. Figure L.8 presents trends in child stunting rates for the Indian states included in the ICMR. Clearly, stunting increased in most states between birth cohorts of the late-1950s and mid-1990s. Only Kerala, Tamil Nadu and Maharashtra saw declines. In addition, while there was considerable stability in the rank order of states by stunting rate since the 1990s, the rank order of states from the 1950s changed quite dramatically. Kerala and Tamil Nadu moved from being states with very high stunting rates to states with some of the lowest. Maharashtra also improved its relative ordinal position between the 1950s and 1990s. These changing patterns again highlight the difficulty of assuming stable hierarchies over time in order to predict a national stunting trend.

Finally, as we have shown in the previous appendix, studies that come from varying ethnic groups or regions can alter the national estimated trend. This is why it is helpful to explore the state level pattern of stunting in India over time. Figure L.9 presents stunting trends in six states included in the ICMR and containing at least one other local study along with the DHS trend from the 1990s onward. Most small-scale local studies corroborate the inverted U-shaped trend in stunting rates observed in the aggregated data. Although this pattern is puzzling it remains a subject for future researchers to understand the reasons behind the increase in India's stunting rates from the 1950s to the 1990s. However, it is worth delving into specific studies in greater detail to determine the factors contributing to variations when compared to the ICMR data. These disparities could stem from variances within states or differences in the socioeconomic status of the families in the samples.

For instance, consider Punjab: both Singh et al. (1987) and Naik et al. (1975) reported stunting rates lower than those indicated by the ICMR. Singh et al. (1987) focused on urban children in Patiala city, who had access to education and healthcare services, while Naik et al. (1975) examined children from rural schools with an existing mid-day meal program. The disparity between these studies and the ICMR data likely reflects variations

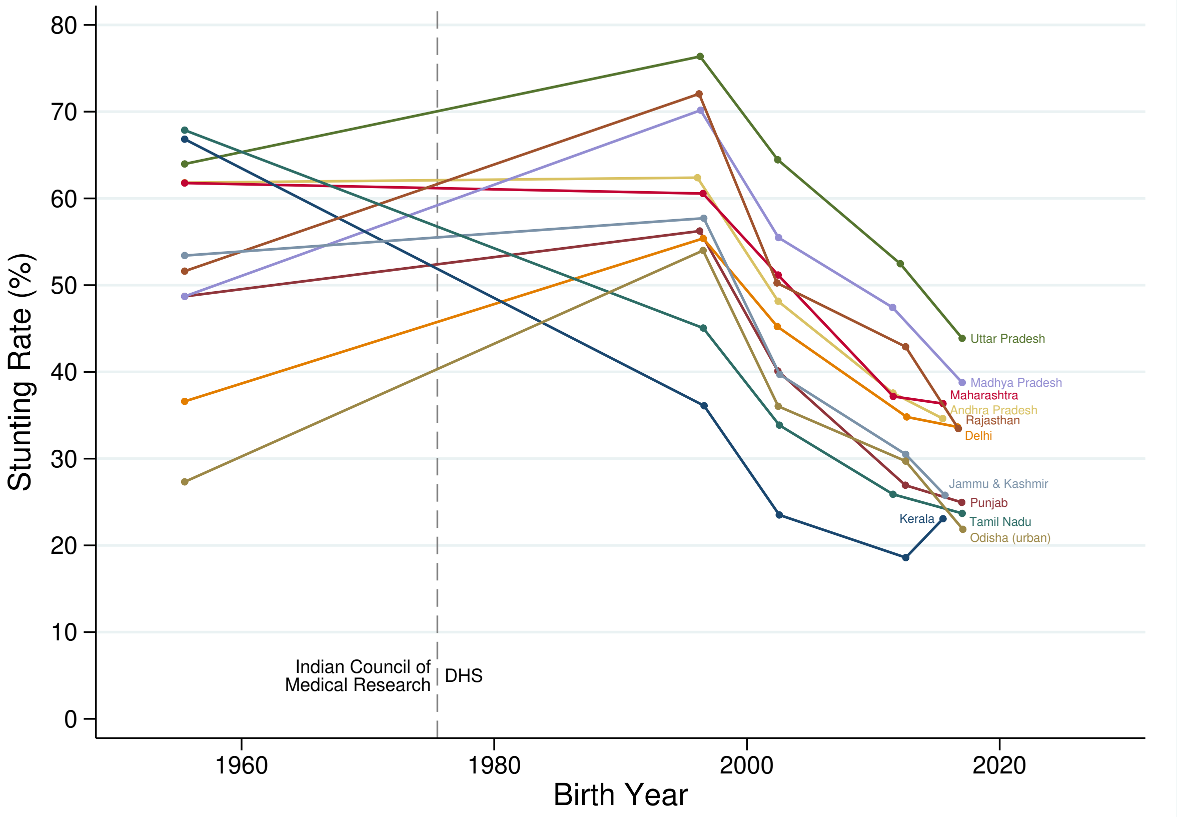

Figure L.8: Stunting Rates in states covered by the Indian Medical Research Council (IMCR) study compared with all states in later DHS studies

Notes: State sample weights are applied to the DHS stunting rates.

Sources: Worldwide Historical Stunting Dataset; India Demographic and Health Surveys in 1998-9, 2005, 2015 and 2019.

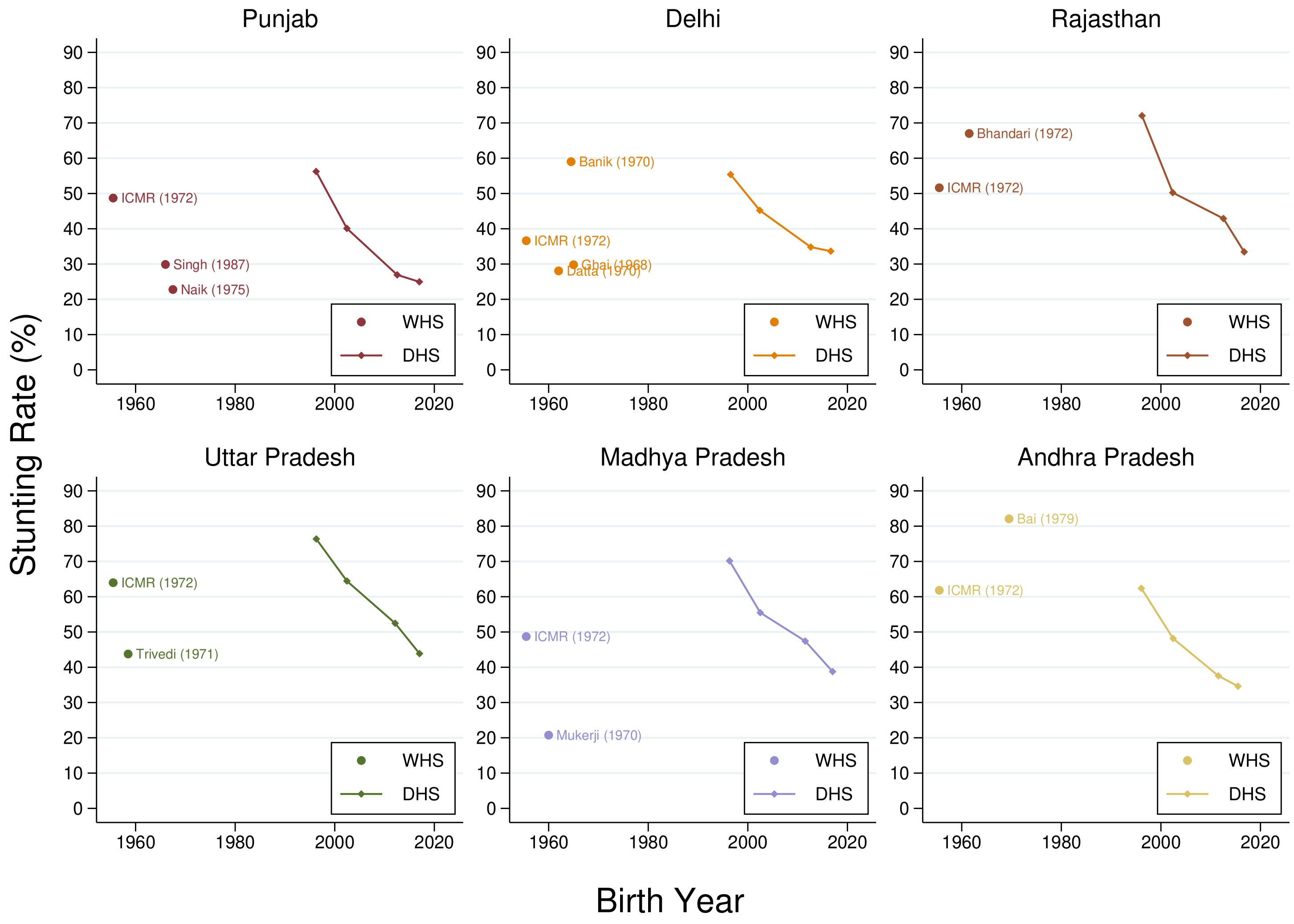

Figure L.9

Notes: Full reference provided in attached Excel file. See Appendix J.

Sources: Worldwide Historical Stunting Dataset; India Demographic and Health Surveys in 1998-9, 2005, 2015 and 2019.

in the socioeconomic backgrounds of the individuals sampled. Similarly, in Rajasthan, Bhandari et al. (1972)'s data included large rural families of low socioeconomic status residing in villages near Udaipur city, potentially accounting for a higher stunting rate compared to the ICMR data. Likewise, the sample from Bai et al. (1979) in Andhra Pradesh corresponds to rural children living in the Kambadur described as a "backward and drought-prone area" by the author.

A last example can be drawn from Mukerji (1970) data in Madhya Pradesh, which included 671 children from two private schools and 329 children from a public school in the city of Jabalpur. Again, it is likely that these children belonged to families with a higher socioeconomic background compared to the more comprehensive sample of the ICMR. Additionally, it might be important to consider variations within the state. For example, the stunting rates in Madhya Pradesh in 2016 ranged from 19% to 51% (Kim et al., 2021).

In conclusion, a more detailed view of the historical studies available for India shows how important regional differences can be and also that these regional differences do not necessarily remain stable over time. For these reasons, we prefer an approach that emphasises the differences in certainty of evidence across studies rather than trying to combine all data using a Bayesian Hierarchical Model.

L.4 South Africa

Related to the discussion of ethnic groups in Kenya above, differences in child stunting between different racial groups may create bias in our understanding of the stunting trend over time, especially if studies only covered one racial group in the population. This could apply in a number of countries such as the United States, New Zealand, Cuba and South Africa. However, it is important to note that in order for racial differences to have a strong impact on the national-level stunting rate, at least one of three features must be true: 1) the racial minority must make up a large share of the population; 2) the data must be drawn from the minority exclusively; and/or 3) there must be very large differences in stunting rates across racial groups. In the case of the United States, the Black population

share was relatively low varying between 10% and 12% of the population. The racial differences in stunting were also relatively small both in the United States and in New Zealand. Thus, racial categorisation is unlikely to severely bias population estimates of the stunting rate in these cases.

However, the previous conditions could be true in the case of South Africa. While whites made up a small share of the population, probably 10%, there were large racial differences in stunting rates. According to our evidence, child stunting had been eradicated among the White population of South Africa by the 1920s whereas the stunting rates among the Black and Coloured[9] population were much higher. Unfortunately, the earliest representative data that we have for South Africa is for the White population, which makes the national stunting trend unreliable. Figure L.10 presents all studies with their certainty of evidence score and the decadal stunting estimates. The trajectory of stunting rates in South Africa in this figure is confusing, jumping from very low values to higher ones across the twentieth century. The purpose of this appendix is to assess why the data for South Africa presents such an odd pattern and whether this is driven by racial coverage within each study. It also illustrates how racial differences might bias population estimates of the stunting rate in countries with different racial groups.

Figure L.11 presents the studies in our dataset distinguishing between racial groups covered by each study. Note that studies that could be aggregated by racial group using population weights in Figure L.10 have been disaggregated to show racial differences in stunting rates. Figure L.11 confirms that the low stunting rates in the decadal trend in the 1920s and 1930s come from studies that only cover the White population. However, the low stunting rate in the 1980s comes from a study that only covers the Black population (Shamssain, 1991), and the relatively low stunting rate in the 1960s also only comes from studies of the Black population (various regional estimates from Walker and Walker 1977). Thus, it is clear that the complications in the trend are not solely driven by the differential coverage of racial groups in each study.

[9] In the South African context, 'Coloured' refers to a distinct racial group mainly in the Cape Province that are descendants of European, African, Asian, and the indigenous Khoisan people. While the term is offensive in some countries, people in this racial group self-identify as 'Coloured' today, so it is necessary and appropriate to use this term in our discussion.

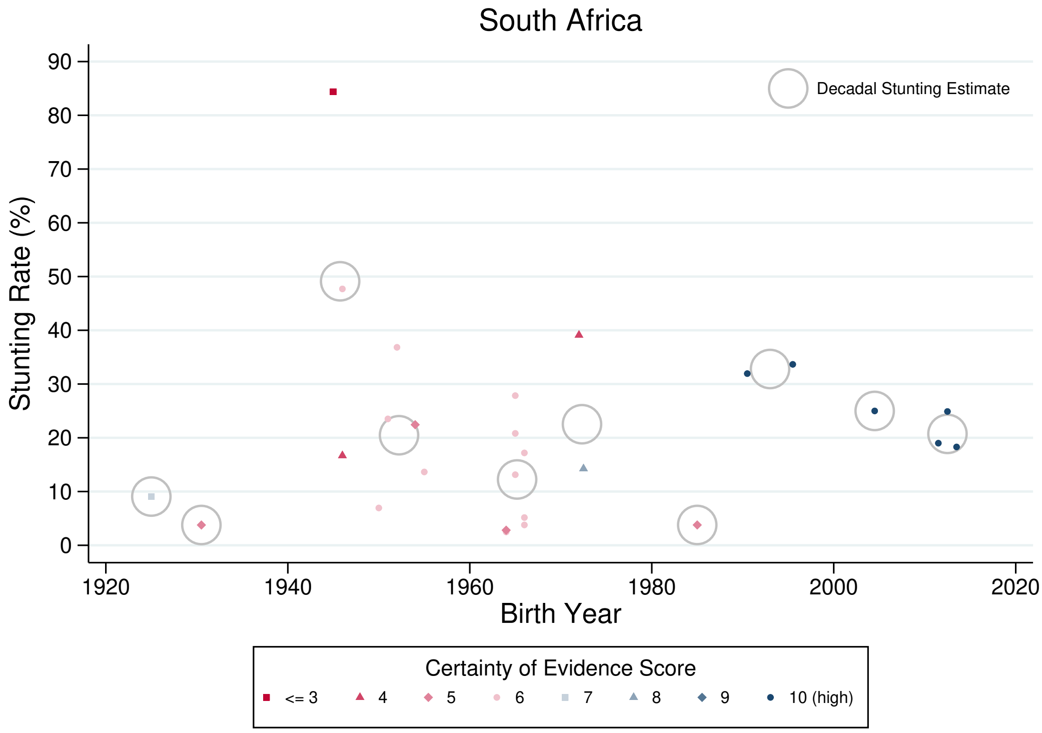

Figure L.10: South Africa Study-Level Stunting Rates and Certainty of Evidence Scores

Sources: Worldwide Historical Stunting Dataset.

Looking at Figure L.11, it is clear that most of the heterogeneity is among the Black population. The White population had a relatively low stunting rate from the early twentieth century and the variation among the Coloured and Indian populations was smaller. Where studies covered more than one racial group, we do see a clear racial gradient: the White population always had a lower stunting rate than the other racial groups. The Indian population seems to have been been in an intermediate position between the White and Black and Coloured populations whereas the Coloured population was sometimes equal to the black population (Smit et al., 1967) and other times in a worse position (Kotzé et al., 1982).

Most puzzling is that where the Black population was sampled in the same location in the 1950s and 1960s, reflected by solid lines connecting two observations (Walker and Walker, 1977), the stunting rate fell between the 1950s and 1960s in all cases. This is puzzling since this is the period of intensifying oppression of the Black population after the formalisation of Apartheid in 1948. While other health indicators did improve for

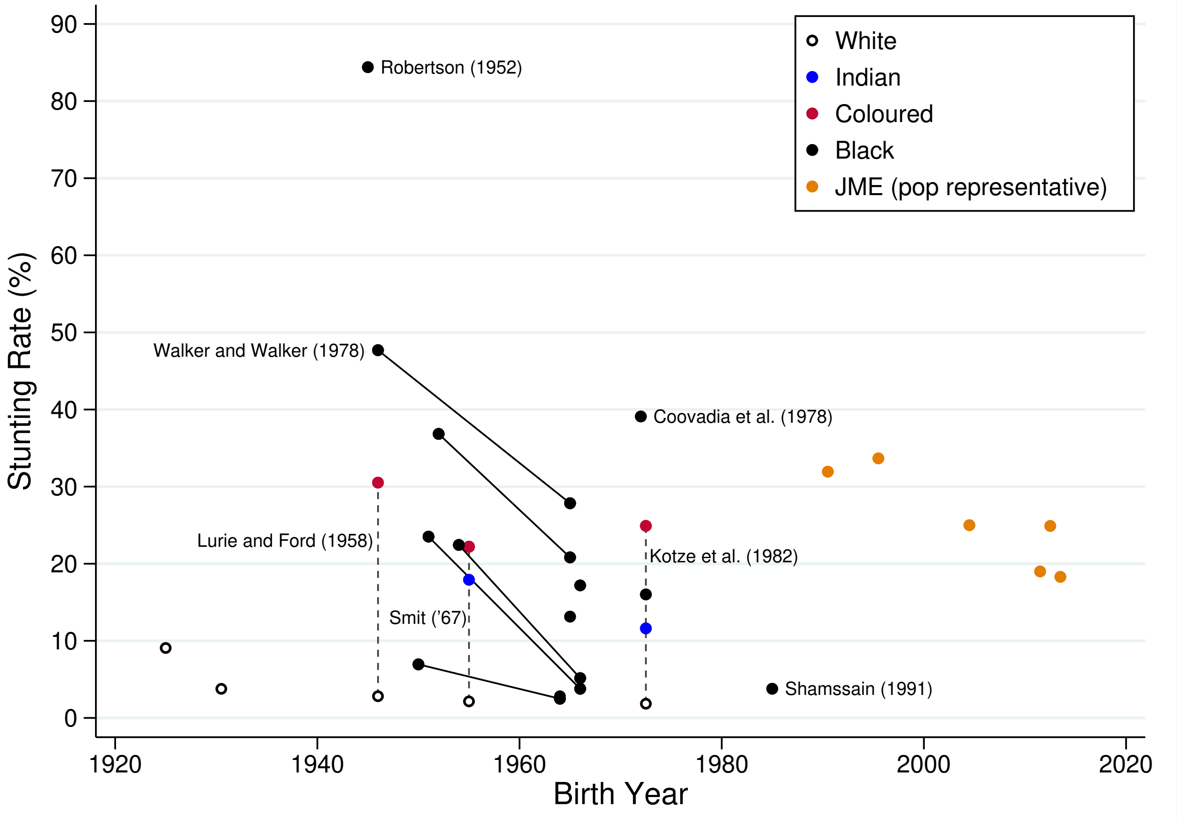

Figure L.11: Stunting Rates in South Africa

Notes: In the South African context, 'Coloured' refers to a distinct racial group mainly in the Cape Province that are descendants of European, African, Asian, and the indigenous Khoisan people. JME is the Joint Malnutrition Estimates from Unicef, WHO, and the World Bank. Points connected by solid lines are from Walker and Walker (1977) and represent the same geographic location over time. Studies connected with vertical dash lines reflect different racial groups being measured in the same study. Studies with multiple race groups have been aggregated in Figure L.10 using the population weights for the population covered by the study.

Sources: Worldwide Historical Stunting Dataset.

the Black population, for instance mortality rates fell in the second half of the twentieth century, the fact that these rates were lower in the 1960s than those observed with the nationally representative studies in the JME in the 1990s suggests that they may suffer from bias. For instance, there may have been positive selection on SES of Black children into the studies even though this was not clear from the studies themselves.

Table L.4 presents characteristics for the studies in Figure L.11 and highlights that the vast majority of the data comes from children measured in schools. While school attendance for the White population was near universal, the attendance for the other racial groups was much lower. This may suggest why across the board, the stunting rate

estimates are lower for Black schoolchildren than for children measured in creches (Coovadia et al., 1978; Robertson, 1952). Still there is a declining stunting rate among Black schoolchildren from the 1940s to 1980s. This could reflect improving health conditions, but it could also be a product of the selection of Black children into schools.

The later studies from Walker and Walker (1977) were all conducted in 1975 and 1976. The urban studies in Walker were for a 'peri-urban' area near Johannesburg and for the township of Soweto where there was mass protest in June 1976 against the apartheid regime. Both of these samples were taken in 1976, though Walker and Walker (1977) does not specify whether the studies were conducted before or after the Soweto uprising began. Walker's data shows stunting rates in Johannesburg and Soweto falling from c. 23% for children born in the early 1950s to c. 4% in the mid-1960s. It is difficult to explain this fall in the stunting rate given the conditions in Soweto at the time, and therefore, it seem most likely that this is a product of selection of children into schools rather than an actual indication of population health. The low levels of stunting for Black children in Kotzé et al. (1982) and Shamssain (1991) also suggest that those Black children remaining in schools were likely positively selected for health status, and therefore, these stunting rates are underestimates for the Black population as a whole.

Overall, the data for South Africa is particularly heterogeneous and difficult to interpret, emphasising how difficult it is to construct nationally representative data in contexts where inequality was particularly high. The complexity of the South African data is partly driven by different racial coverage across studies but also by changing selection into the samples, which is difficult to capture with our general inclusion and exclusion criteria. Clearly, further work, perhaps based on archival records, is required to reconstruct more plausible trends in child stunting for South Africa.

Table L.4: Studies providing stunting rate estimates for South Africa

Study	Birth Year	Race	Spatial Coverage	Urban/Rural	Sample Location
Lurie and Ford (1935)	1925	White	Cape Province	Both	Schools
Kark and le Riche (1944)	1931	White	Pretoria	Urban	Schools
Robertson (1952)	1945	Black	Native township outside of Pretoria	Urban	Creche
Lurie and Ford (1988)	1946	White, Coloured	Cape Town	Urban	Schools
Walker and Walker (1977)	1946-66	Black	Five rural and two urban centres	Both	Schools
Smit et al. (1967)	1955	All	Pretoria	Urban	Schools
Coovadia et al. (1978)	1972	Black	Umlazi-Lamontville area (suburban Durban)	Urban	Schools, Creches and Baby Clinics
Kotzé et al. (1982)	1973	All	All South Africa	Both	Schools
Shamssain (1991)	1985	Black	Umtata	Urban	Schools

Notes: Full references for the studies are given in the reference list.

Sources: Worldwide Historical Stunting Dataset.

M Replication of Main Results Excluding Studies with Low Certainty of Evidence

One of the main limitations of our study is that many historical studies were not nationally representative, perhaps studying one community. This raises questions about the extent to which the results are truly comparable since they may be biased by small community surveys, especially for earlier periods. To assuage fears that these small community studies are biasing our main findings, we reproduce Figures 3 and 4 from the main text excluding all community studies and only keeping studies with a certainty of evidence score of seven or higher (see Appendix I for details). This leads us to drop 70.3% of stunting stunting estimates (1,060 stunting estimates of the 1,508 in the final analytical sample). Of course the, JME dataset is highly reliable so we add this back into get 1,499 stunting estimates with a certainty of evidence score of seven or higher. Figures M.1 and M.2 show the replicated results. While there are some differences in the trends for individual countries, the main results and trends in stunting for countries discussed in the main text are qualitatively similar, highlighting that our main conclusions do not rely on studies where the certainty of evidence is low.

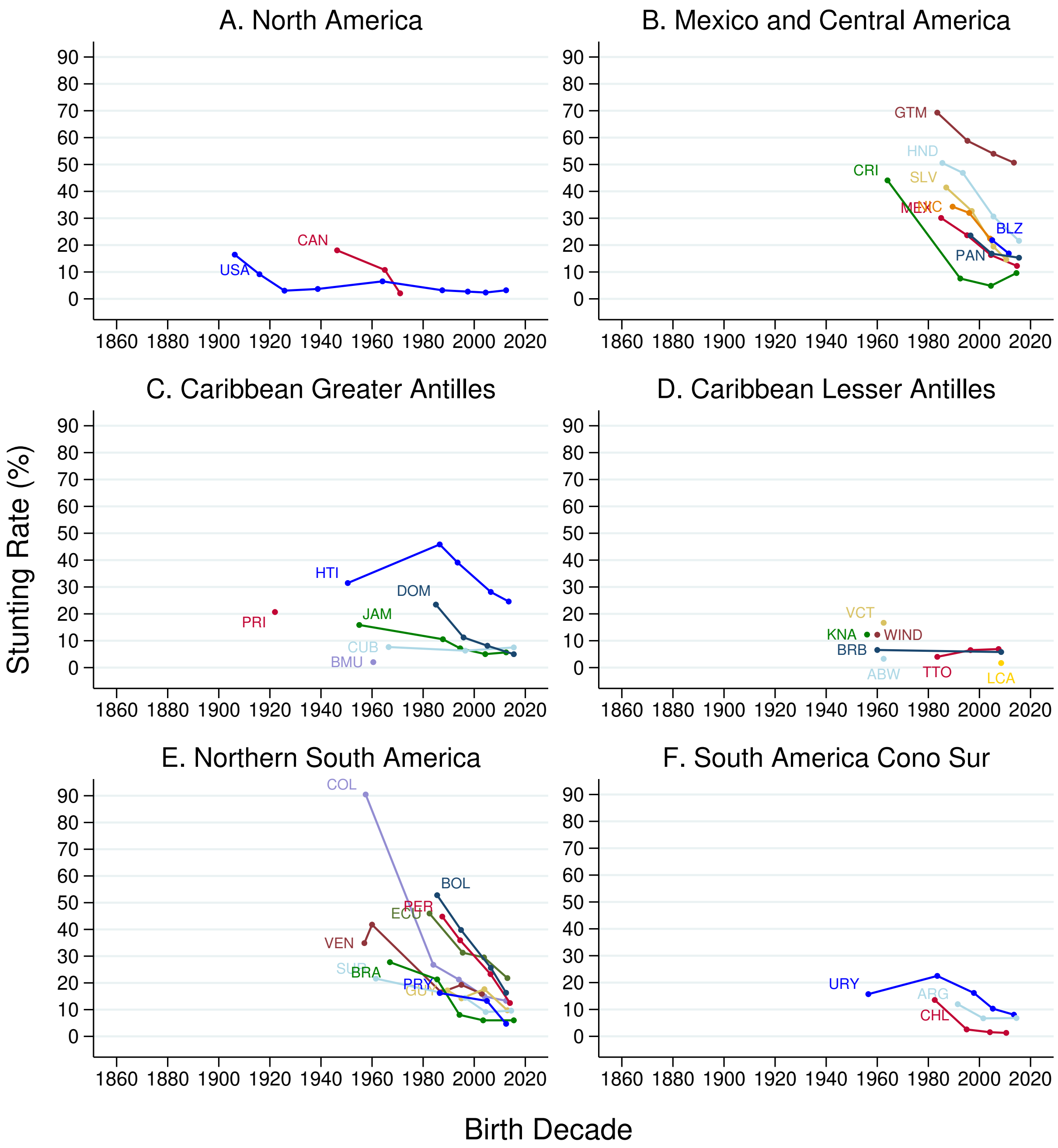

Figure M.1: Country-Level Time Series with High Certainty of Evidence: Americas

Notes: The figures plot decadal mean stunting rates for all countries organised by geographic areas. They replicated Figure 3 in the main text, excluding community studies and studies with certainty of evidence scores of 6 or less (see Appendix I for details). Three-letter codes are standard country codes.

Sources: Worldwide Historical Stunting Database and UNICEF/WHO/World Bank (2023).

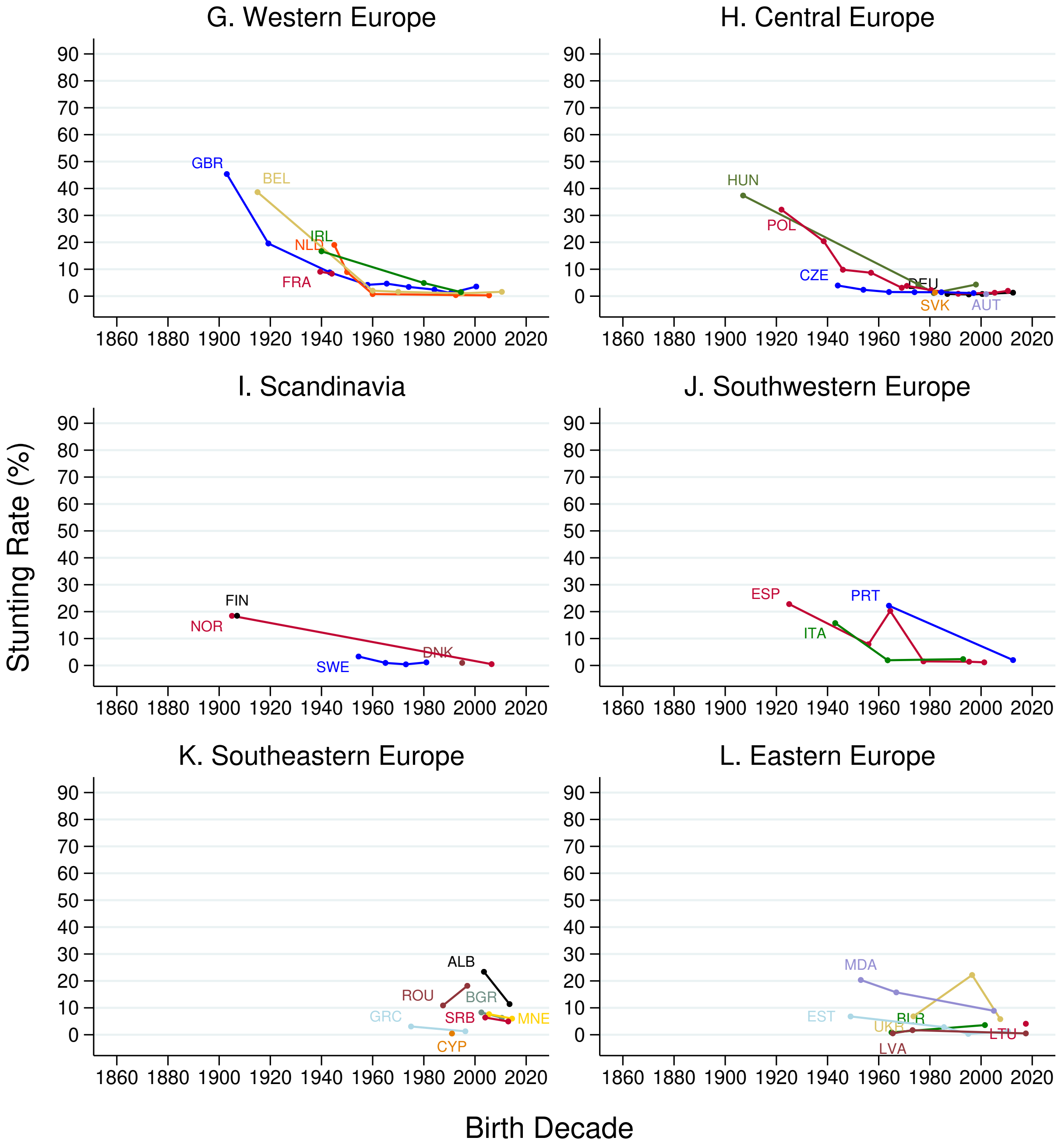

Figure M.1: (Cont.) Country-Level Time Series with High Certainty of Evidence: Europe

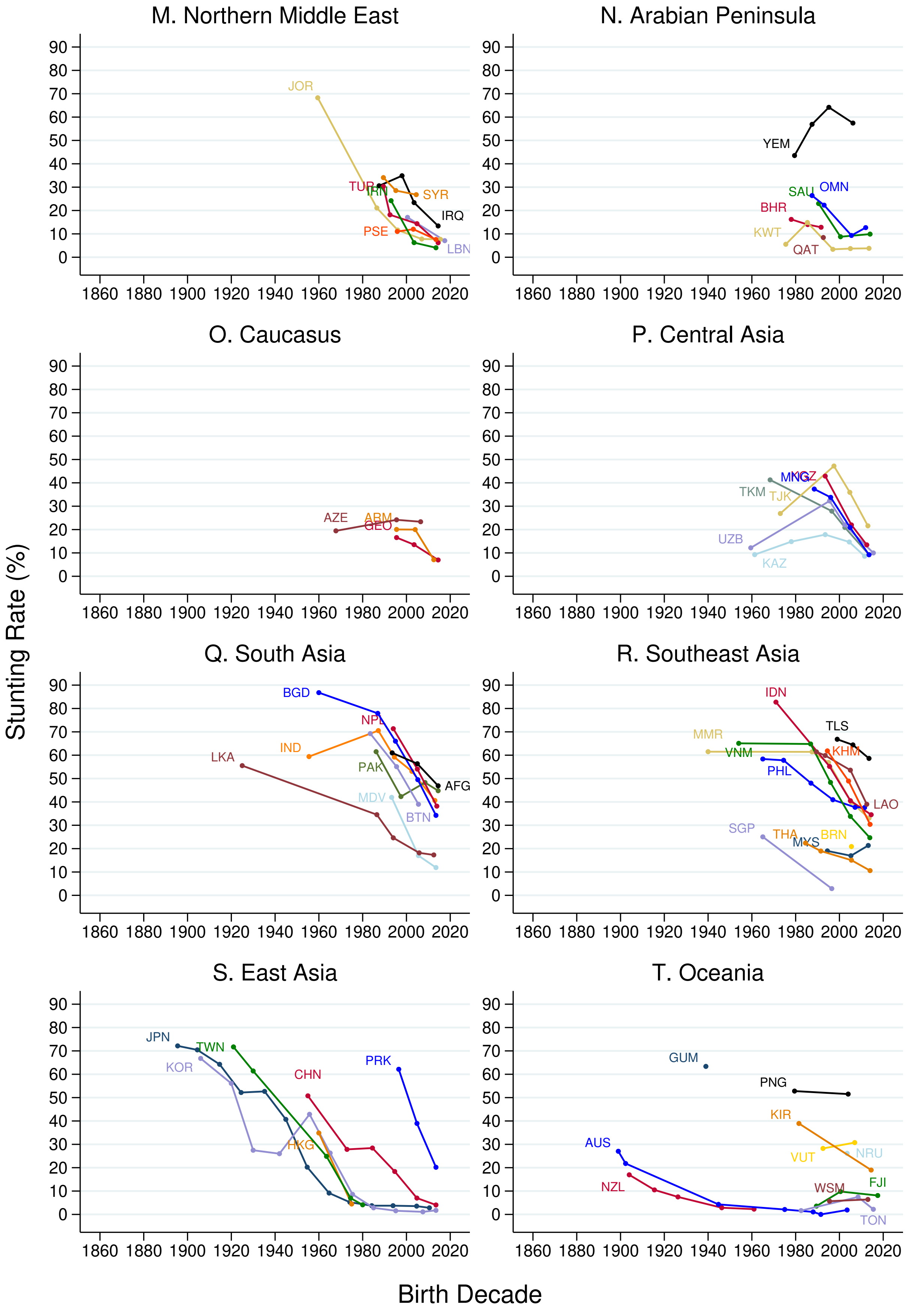

Figure M.1: (Cont.) Country-Level Time Series with High Certainty of Evidence: Asia

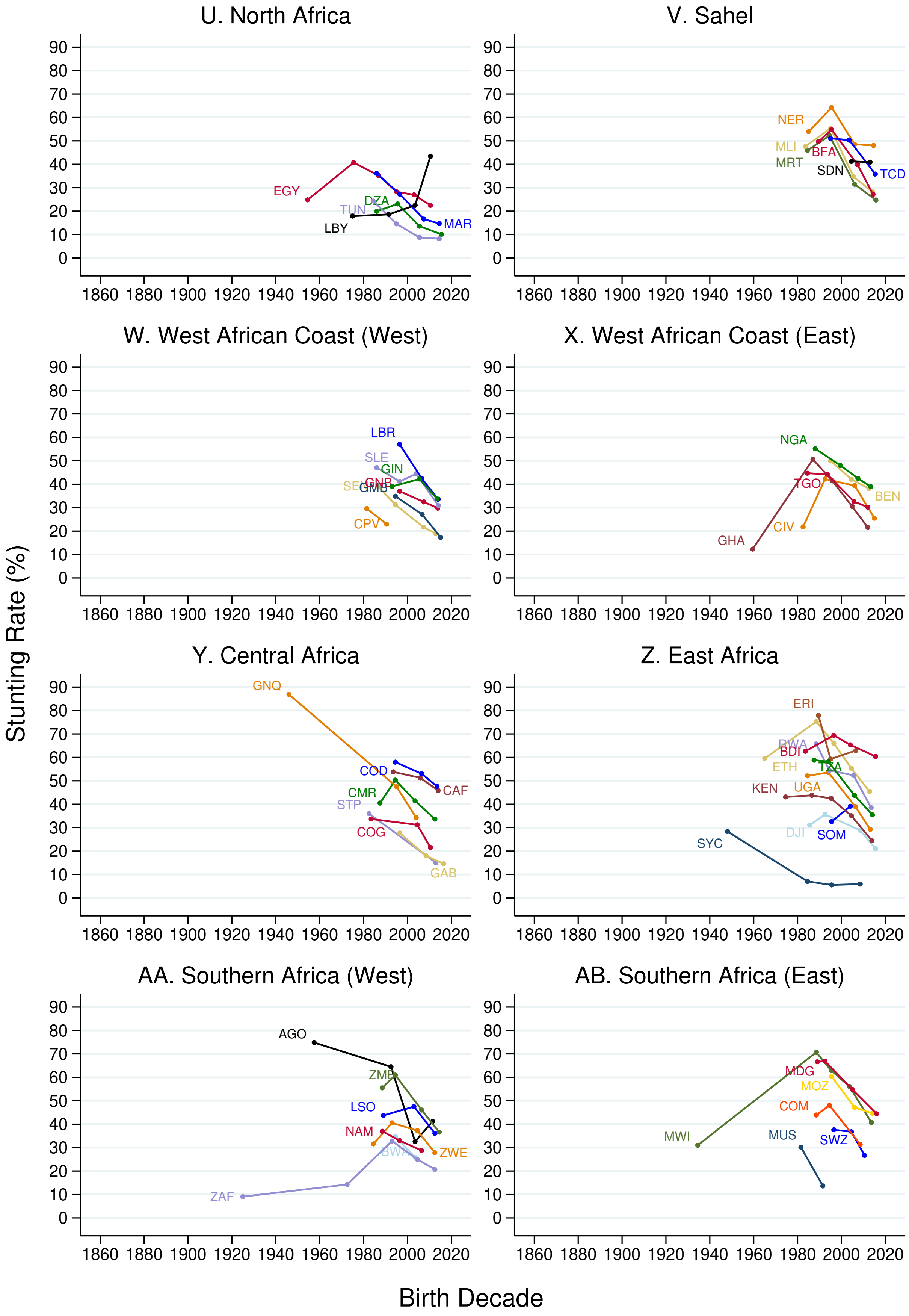

Figure M.1: (Cont.) Country-Level Time Series with High Certainty of Evidence: Africa

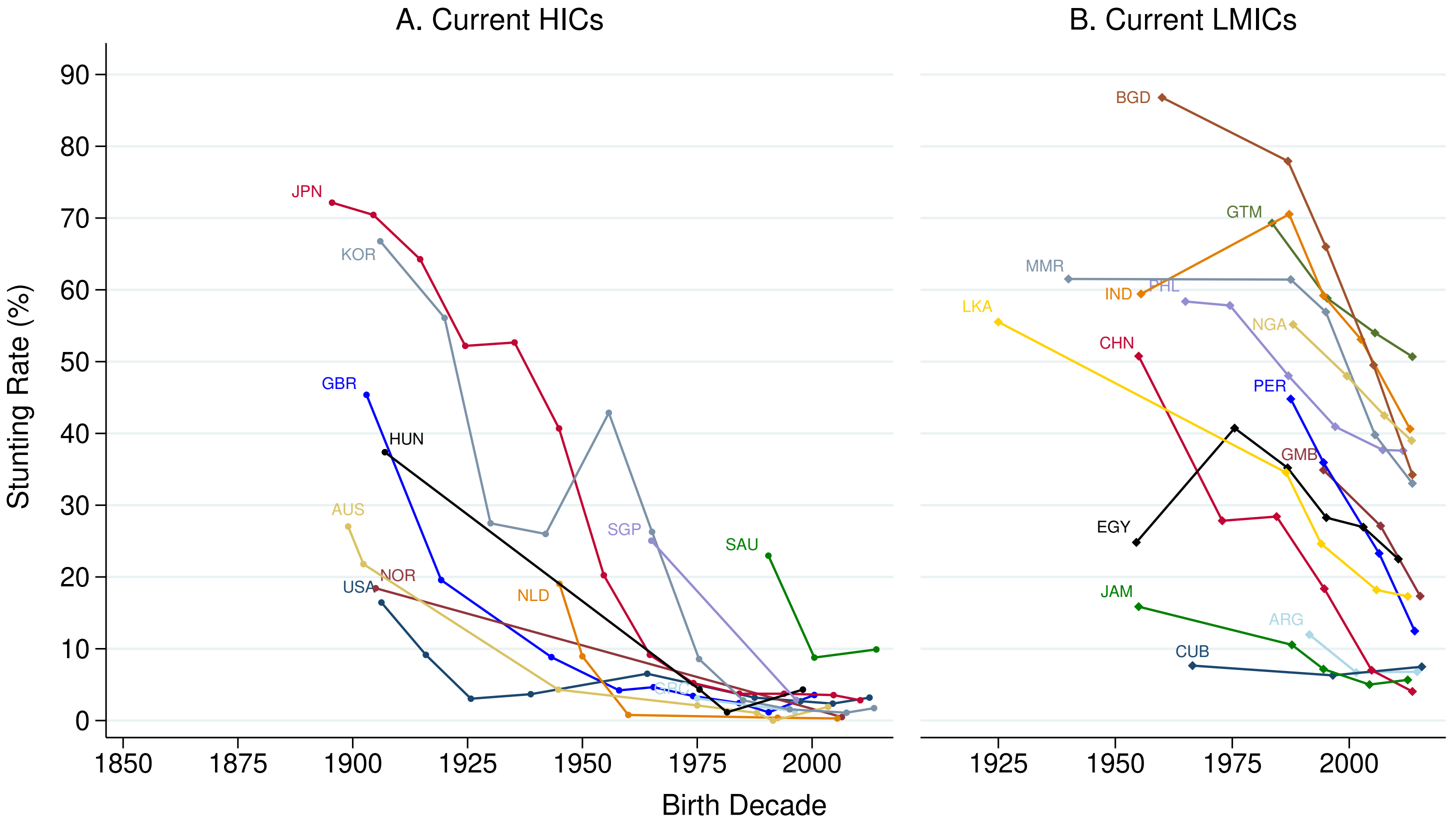

Figure M.2: Comparison of Historical Stunting Trends in HICs and LMICs: High Certainty of Evidence

Notes: This figure replicated Figure 4 in the main text, excluding community studies and studies with certainty of evidence scores of 6 or less (see Appendix I for details). Three-letter codes are standard country codes. The scale of both the x and y-axes in both panels are the same so that the rate of stunting decline can be compared across the two panels.

Sources: Worldwide Historical Stunting Database and UNICEF/WHO/World Bank (2023).

N World Maps of Stunting Rates over Time

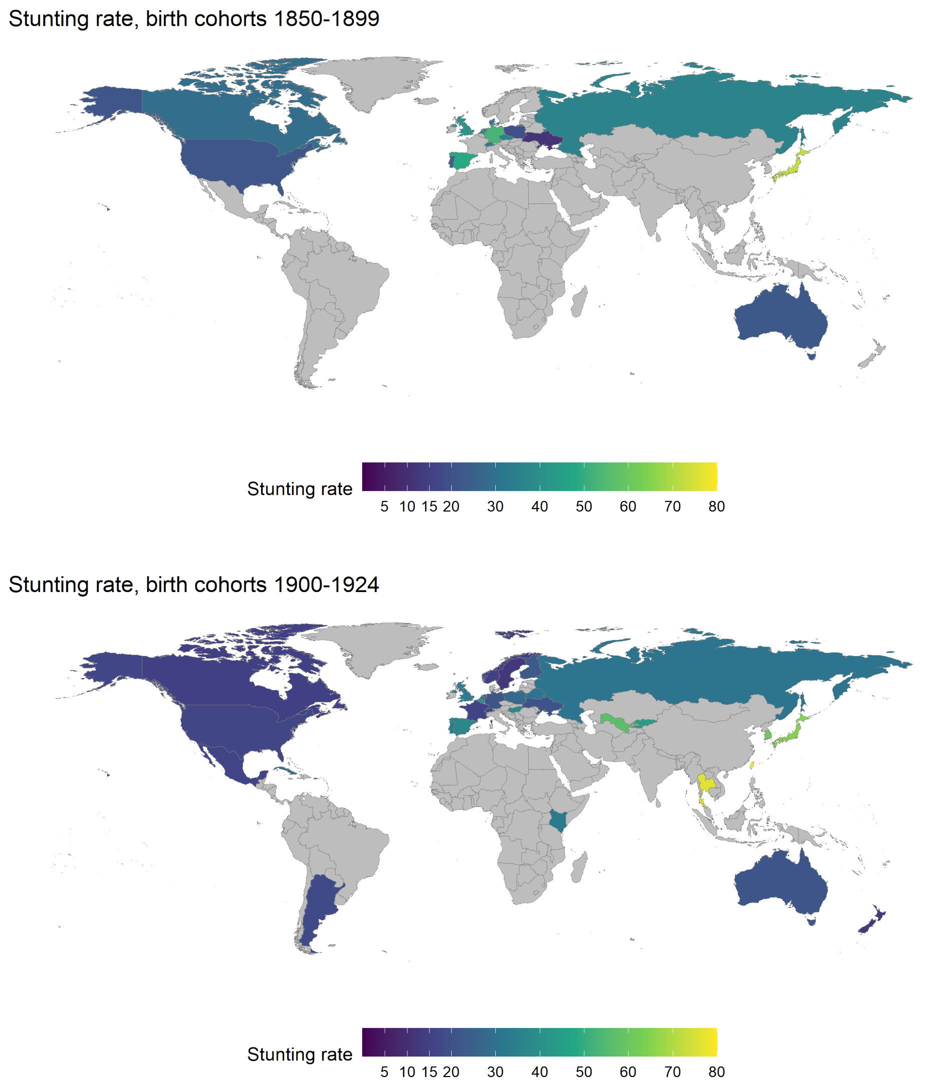

Figure N.1: Global Child Stunting, Birth Cohorts 1850-2020

Sources: Worldwide Historical Stunting Database and UNICEF/WHO/World Bank (2023).

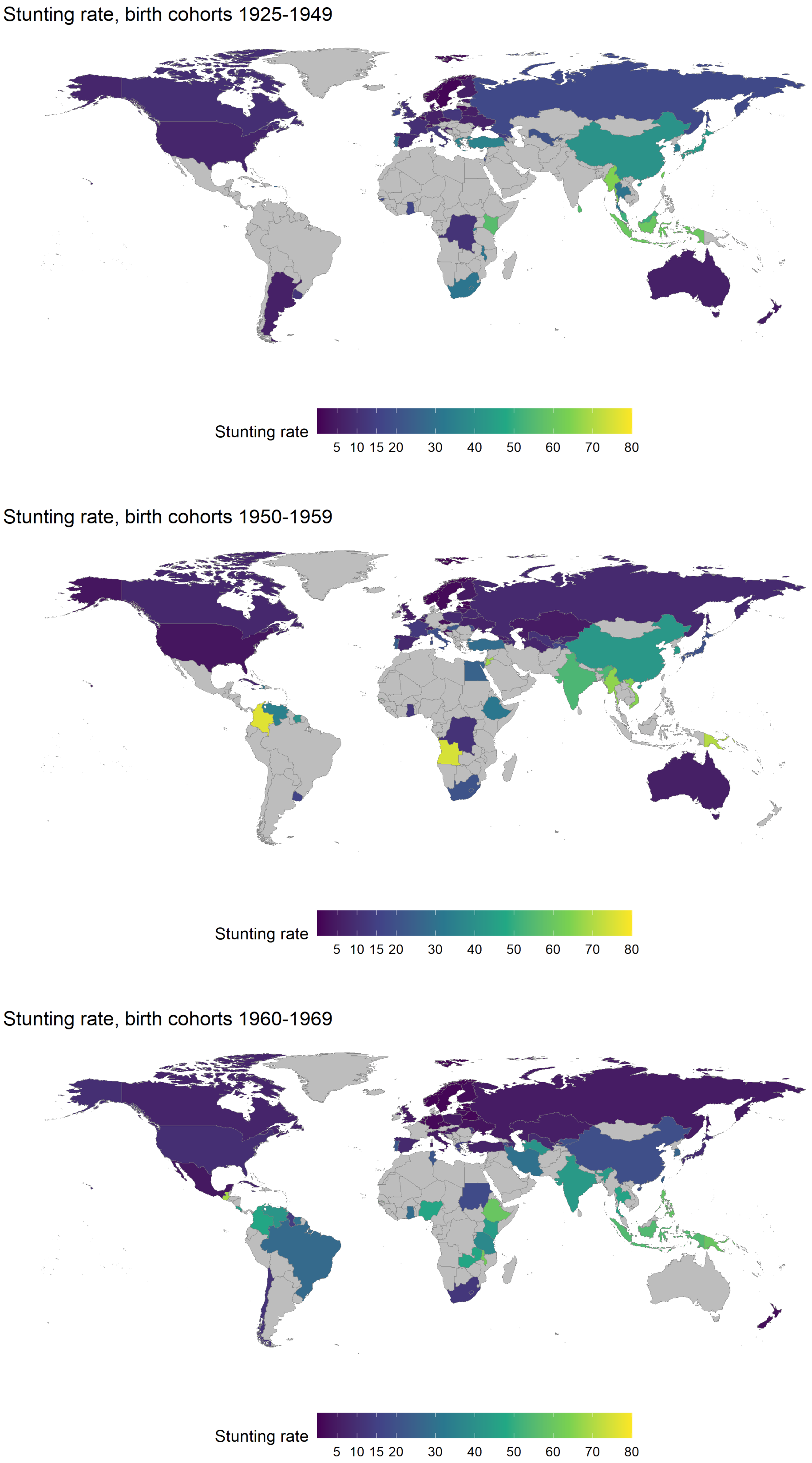

Figure N.1: (Cont.) Global Child Stunting, Birth Cohorts 1850-2020

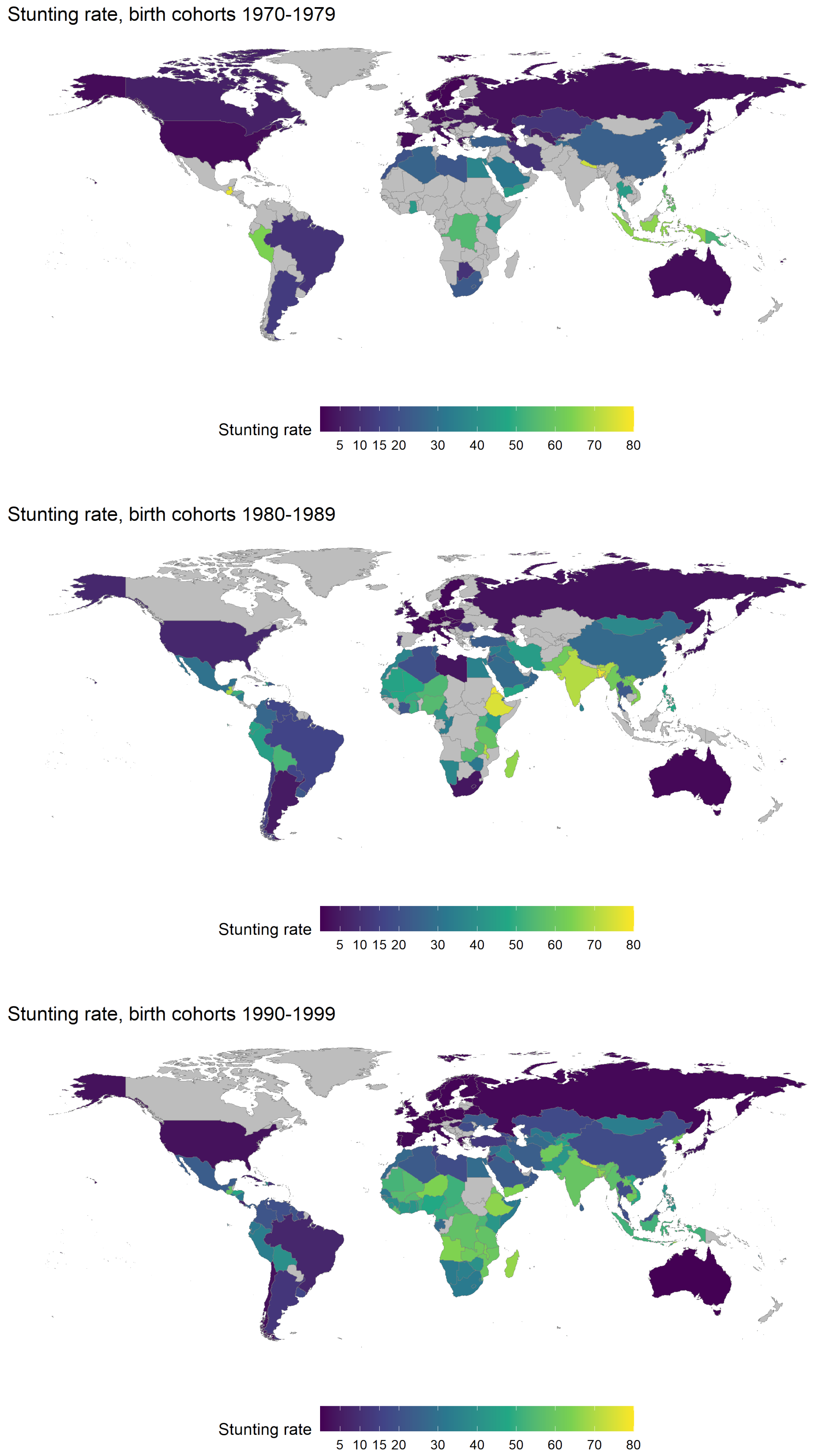

Figure N.1: (Cont.) Global Child Stunting, Birth Cohorts 1850-2020

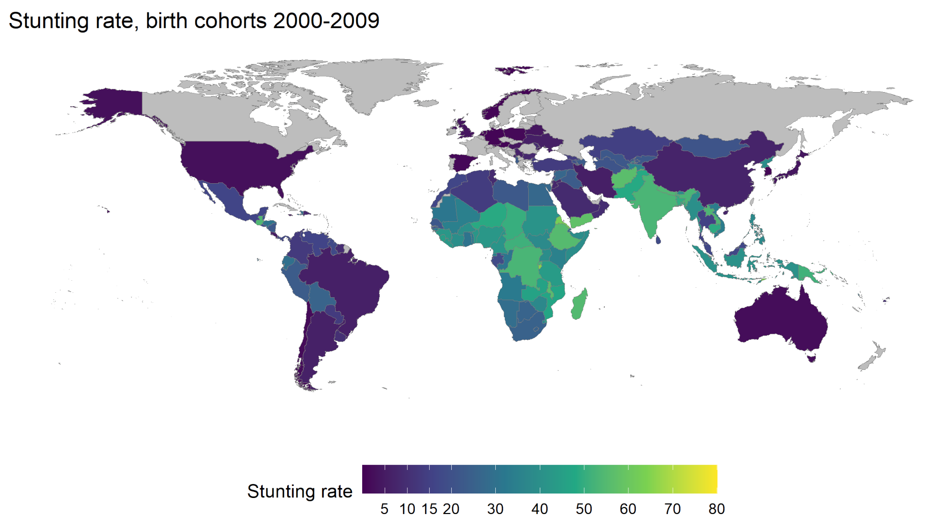

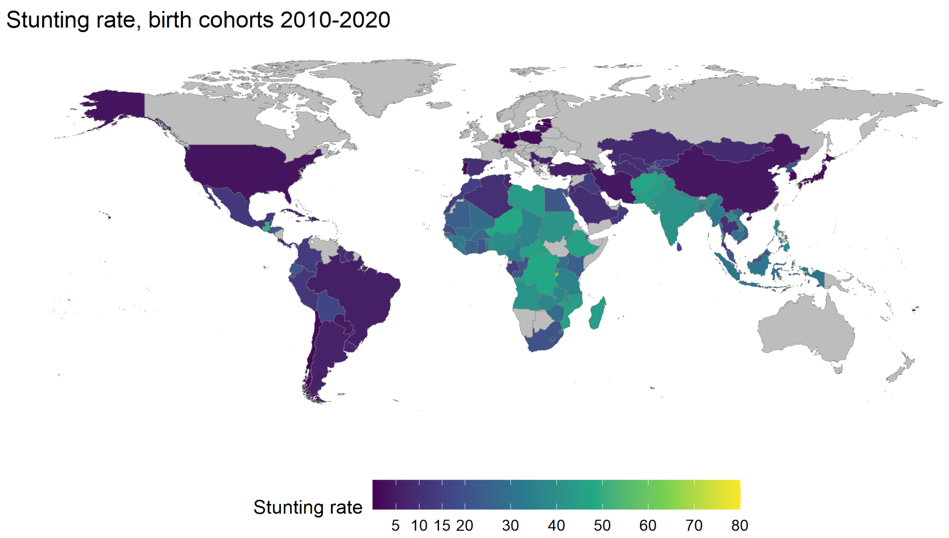

Figure N.1: (Cont.) Global Child Stunting, Birth Cohorts 1850-2020

O Country Study-Level Stunting Rate Graphs

Figures O.1 to O.122 show all studies included in our analysis along with their certainty of evidence score. This allows individual researchers to assess the certainty of evidence underpinning the decadal stunting rates for each country, and by comparing with the data described in Appendix J, be able to look at the further characteristics of each study.

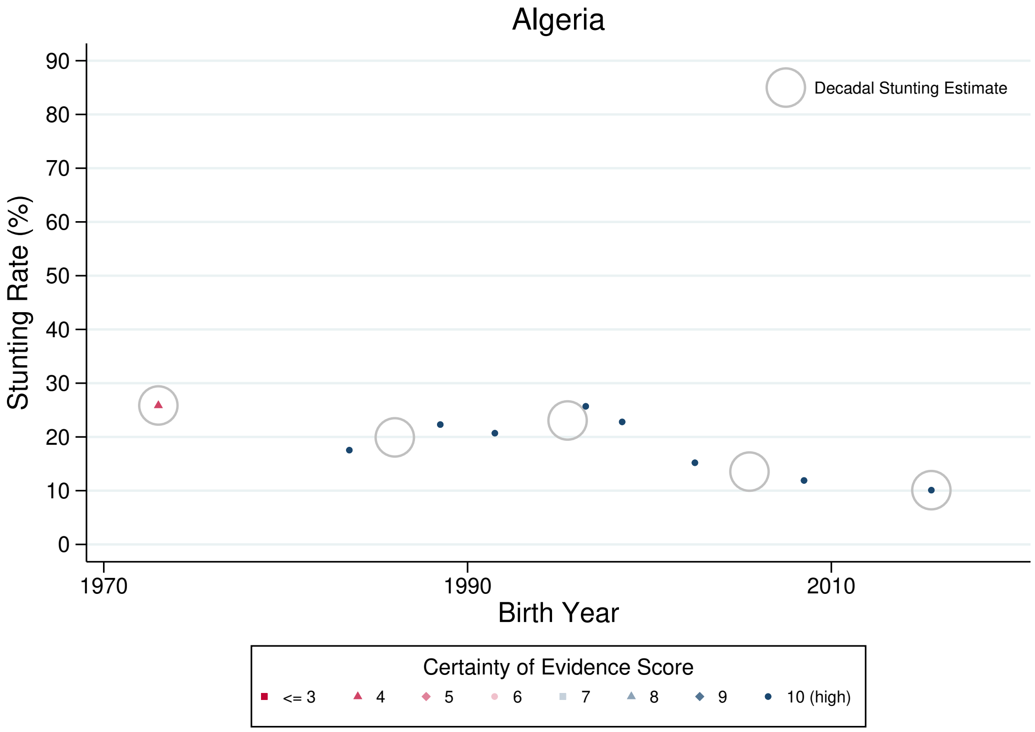

Figure O.1: Algeria Study-Level Stunting Rates and Certainty of Evidence Scores

Sources: Worldwide Historical Stunting Dataset.

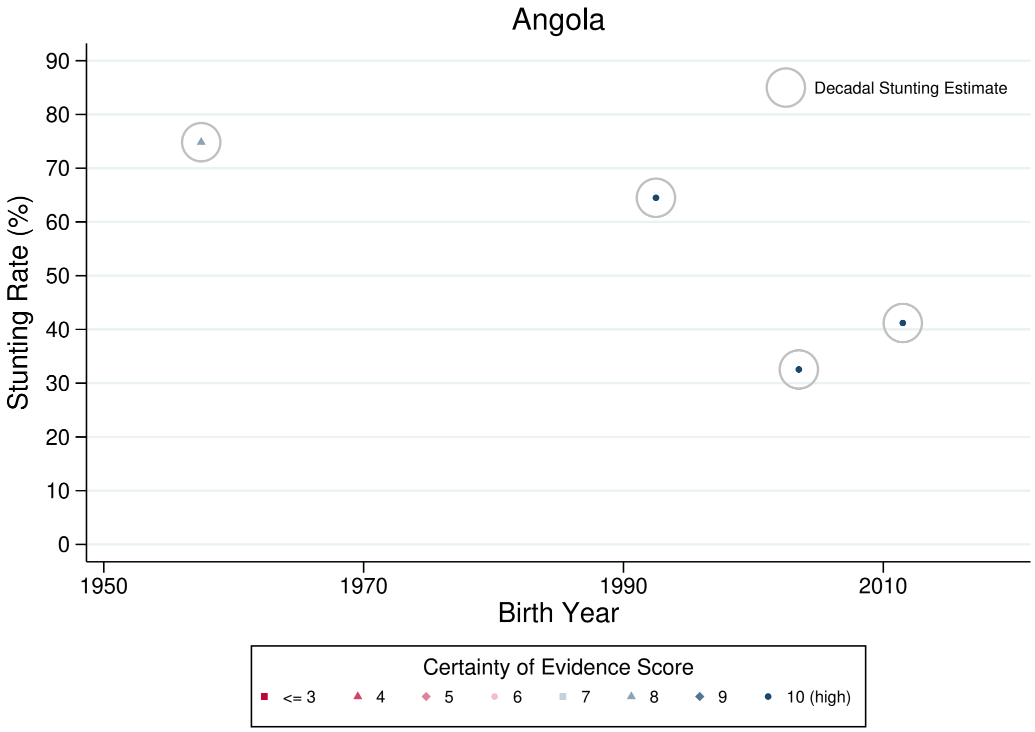

Figure O.2: Angola Study-Level Stunting Rates and Certainty of Evidence Scores

Sources: Worldwide Historical Stunting Dataset.

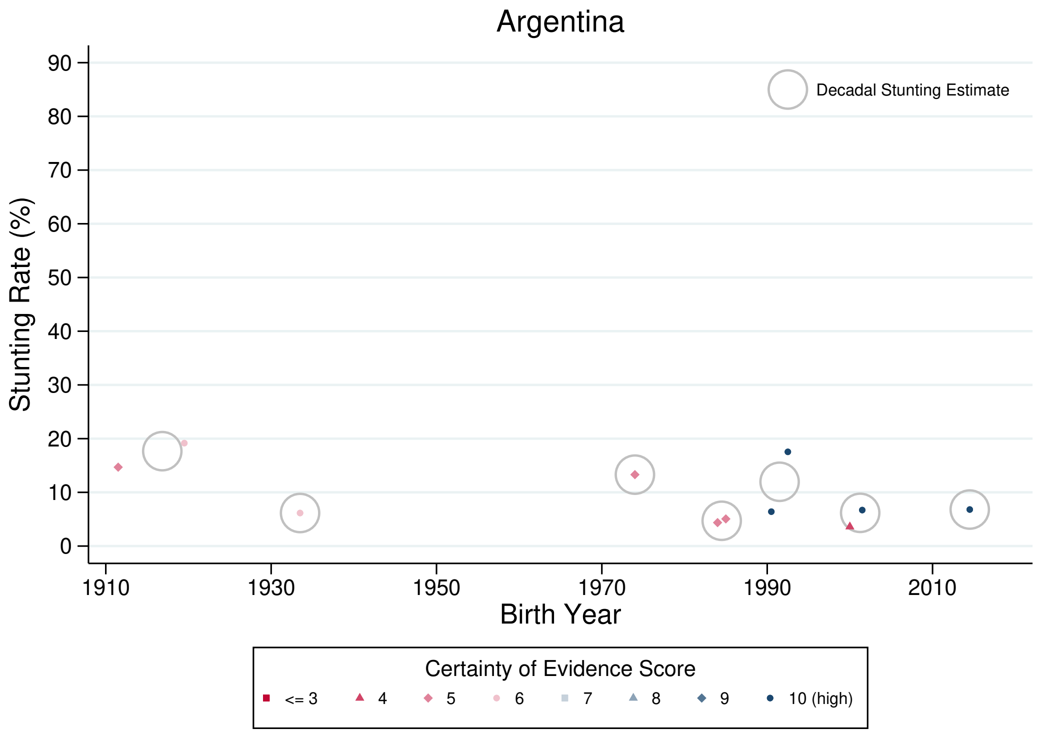

Figure O.3: Argentina Study-Level Stunting Rates and Certainty of Evidence Scores

Sources: Worldwide Historical Stunting Dataset.

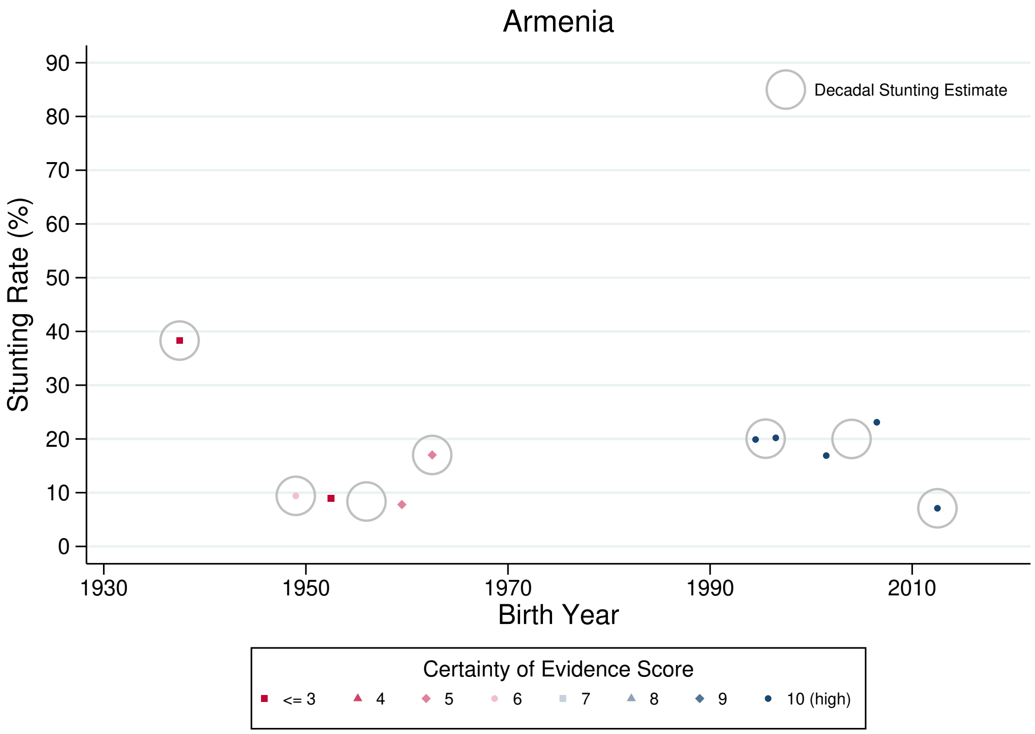

Figure O.4: Armenia Study-Level Stunting Rates and Certainty of Evidence Scores

Sources: Worldwide Historical Stunting Dataset.

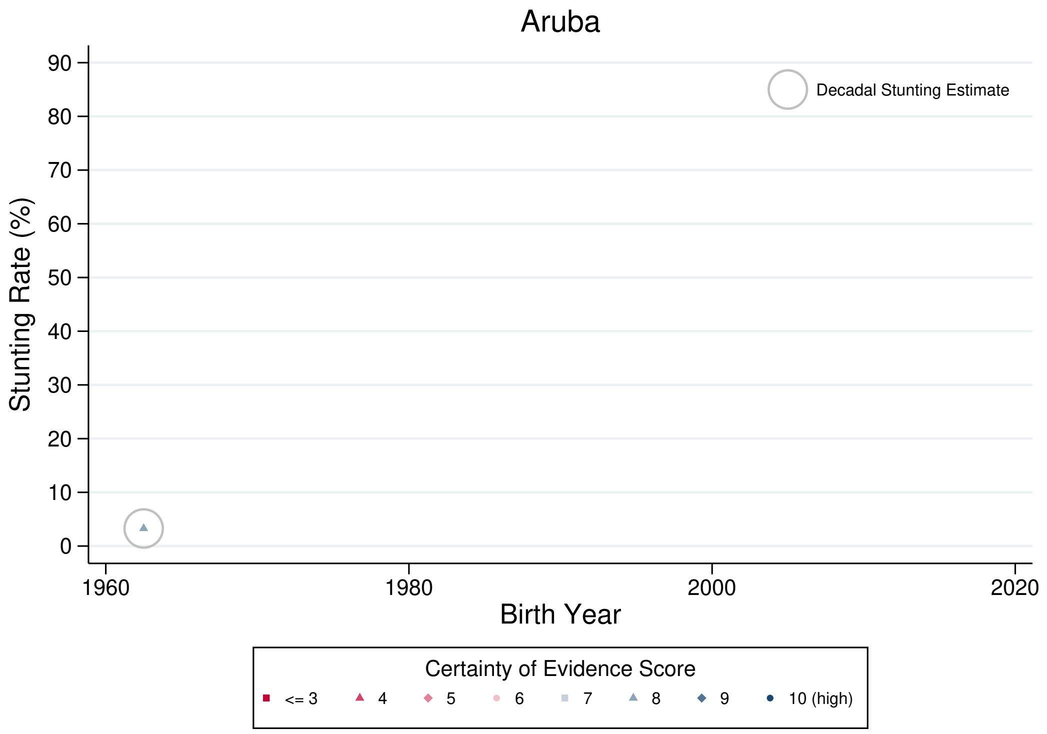

Figure O.5: Aruba Study-Level Stunting Rates and Certainty of Evidence Scores

Sources: Worldwide Historical Stunting Dataset.

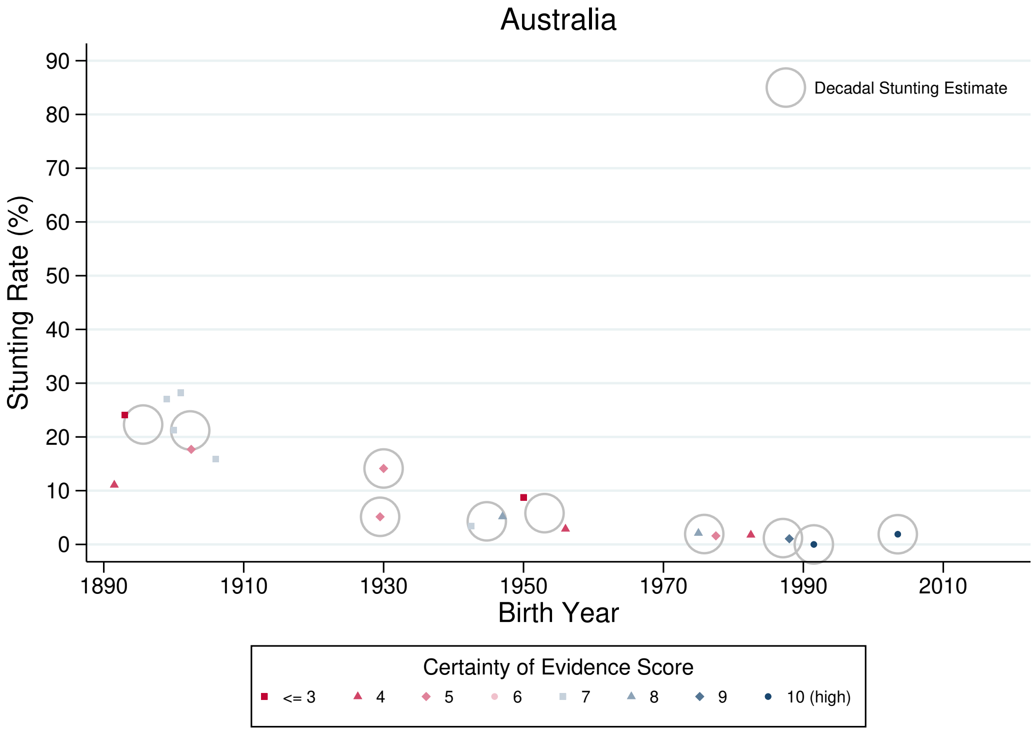

Figure O.6: Australia Study-Level Stunting Rates and Certainty of Evidence Scores

Sources: Worldwide Historical Stunting Dataset.

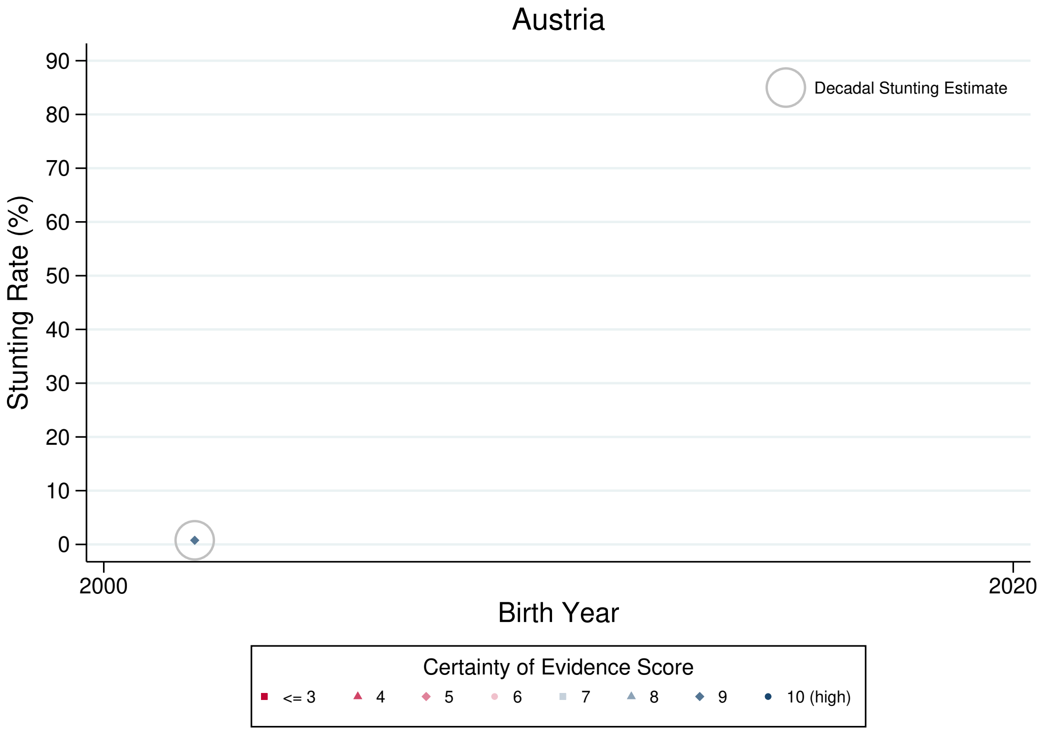

Figure O.7: Austria Study-Level Stunting Rates and Certainty of Evidence Scores

Sources: Worldwide Historical Stunting Dataset.

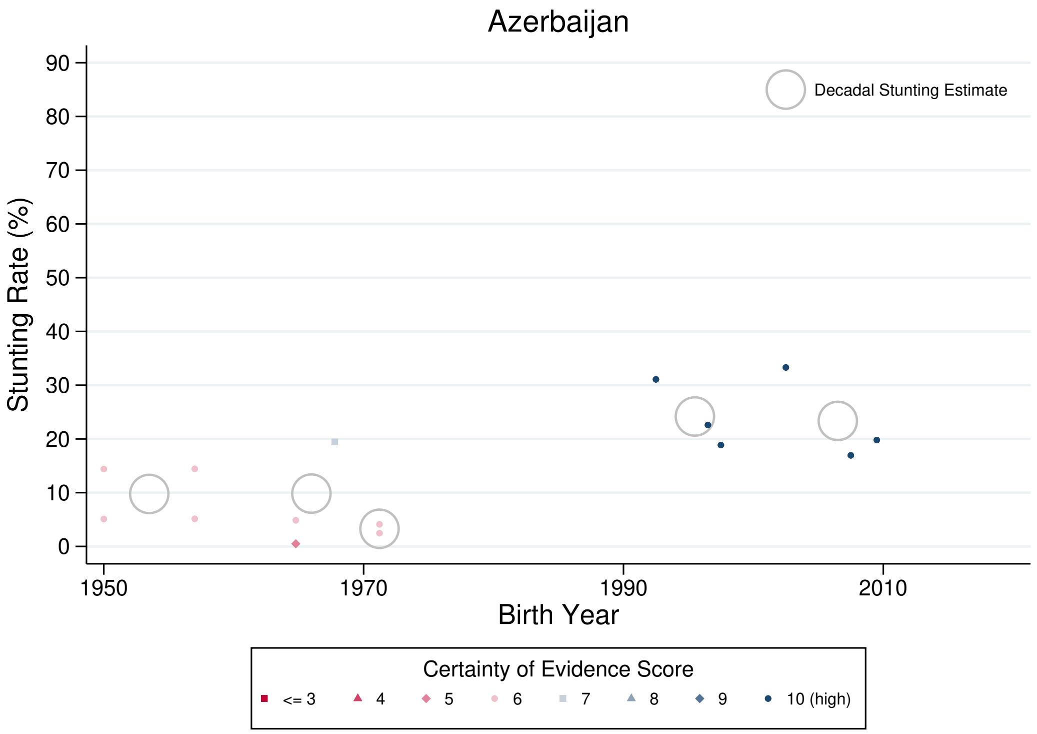

Figure O.8: Azerbaijan Study-Level Stunting Rates and Certainty of Evidence Scores

Sources: Worldwide Historical Stunting Dataset.

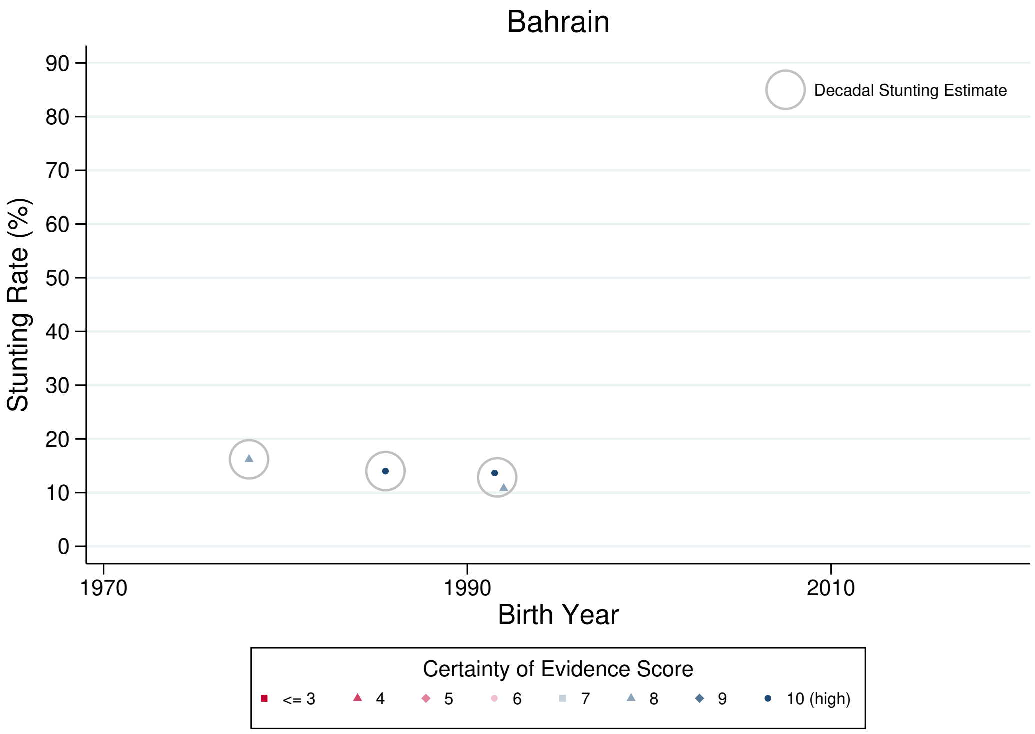

Figure O.9: Bahrain Study-Level Stunting Rates and Certainty of Evidence Scores

Sources: Worldwide Historical Stunting Dataset.

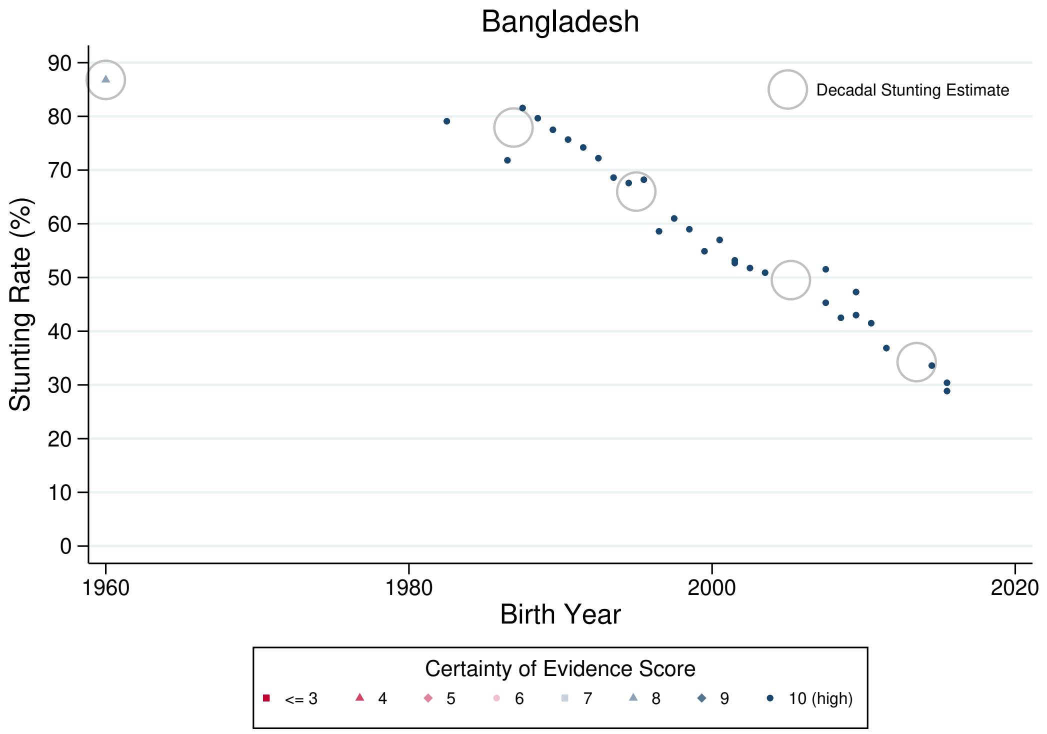

Figure O.10: Bangladesh Study-Level Stunting Rates and Certainty of Evidence Scores

Sources: Worldwide Historical Stunting Dataset.

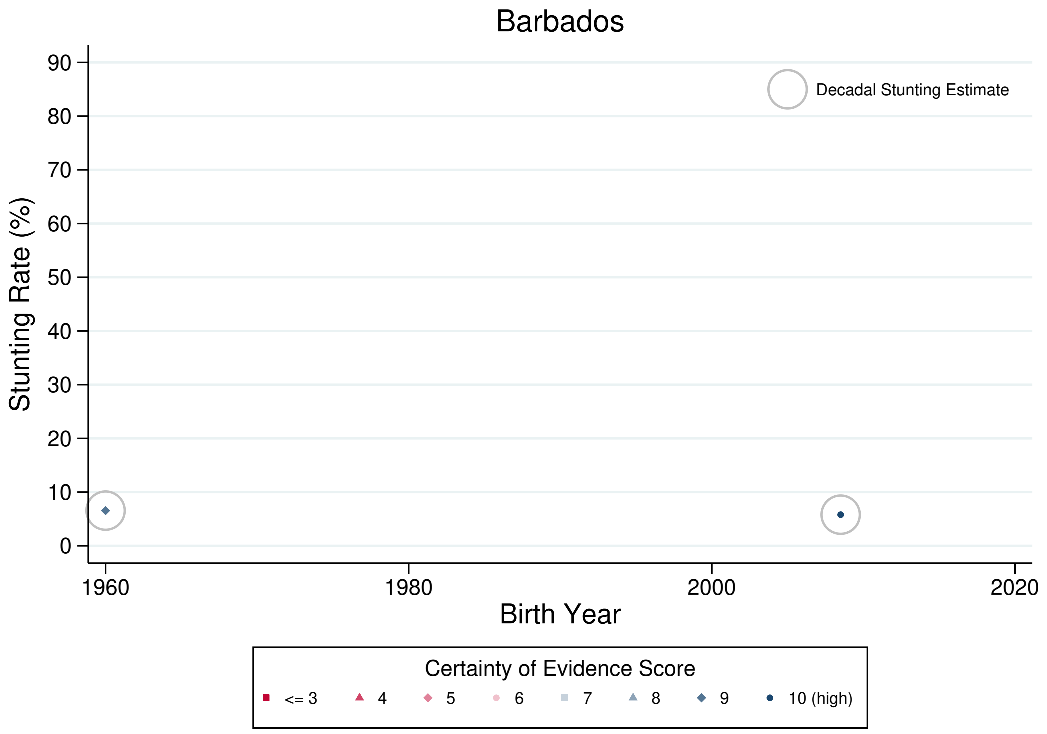

Figure O.11: Barbados Study-Level Stunting Rates and Certainty of Evidence Scores

Sources: Worldwide Historical Stunting Dataset.

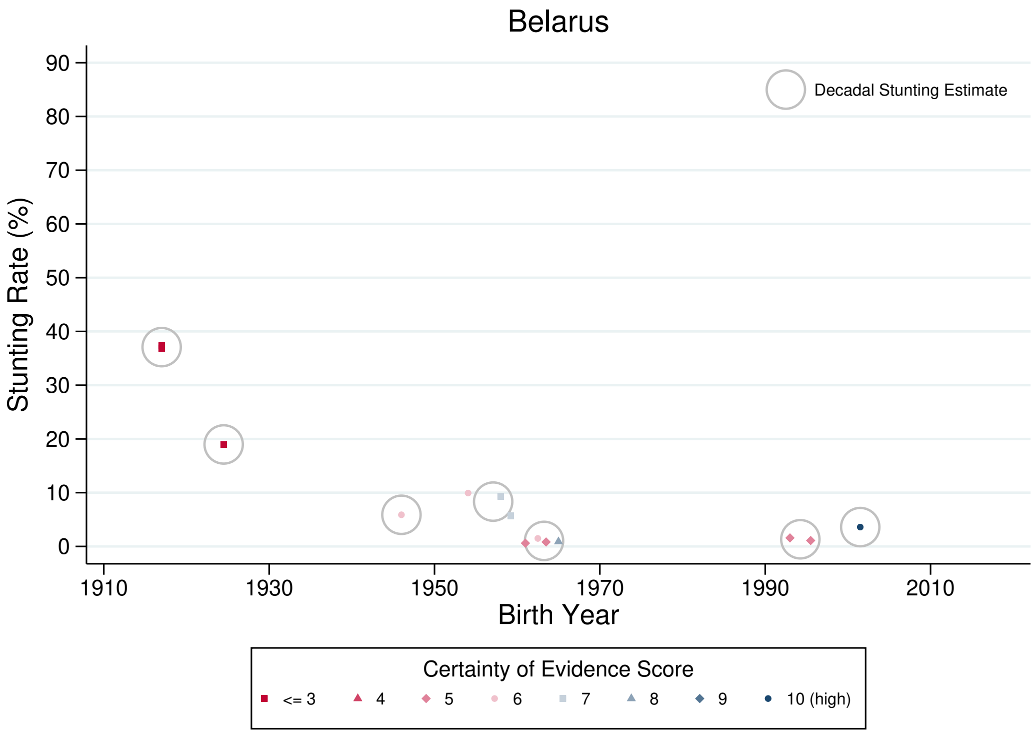

Figure O.12: Belarus Study-Level Stunting Rates and Certainty of Evidence Scores

Sources: Worldwide Historical Stunting Dataset.

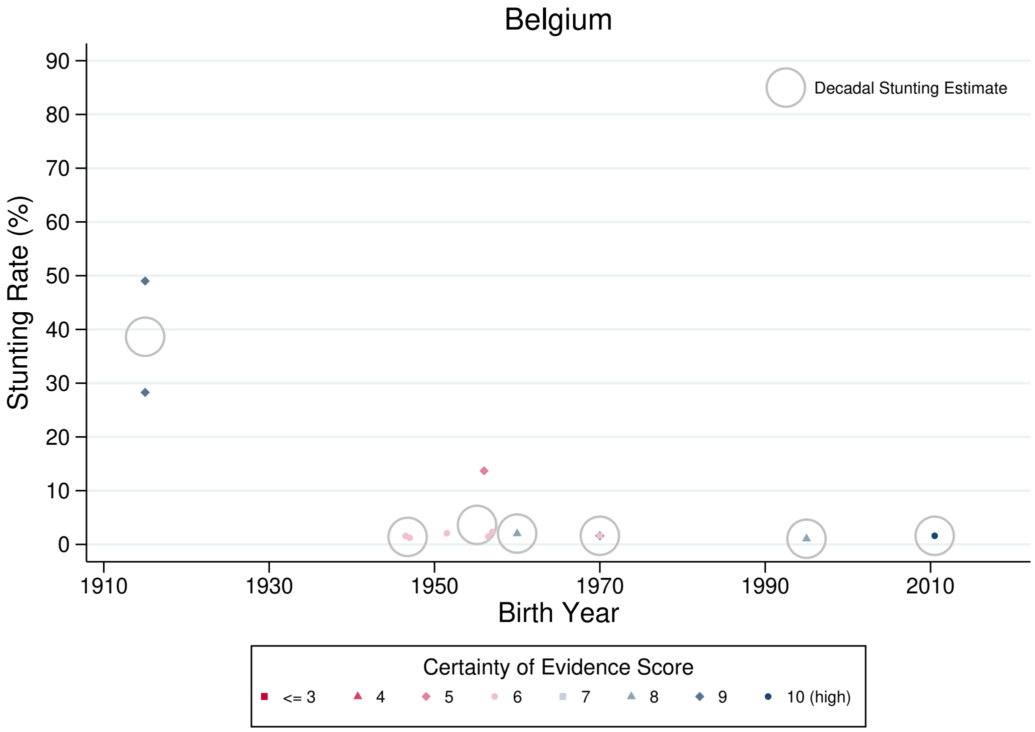

Figure O.13: Belgium Study-Level Stunting Rates and Certainty of Evidence Scores

Sources: Worldwide Historical Stunting Dataset.

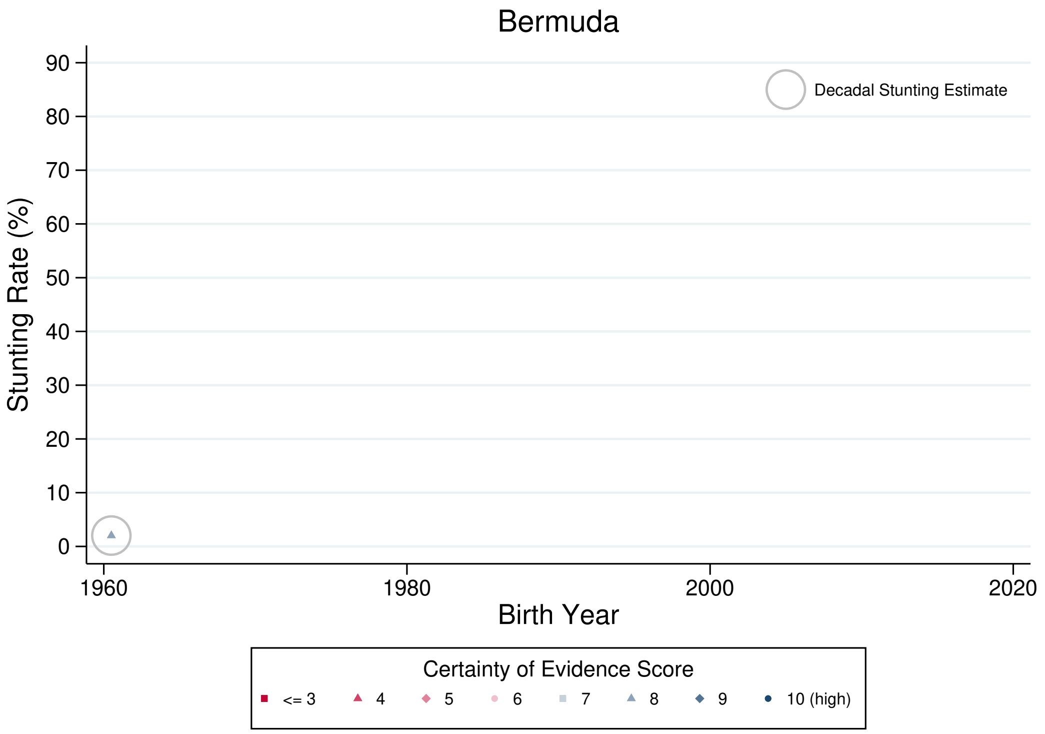

Figure O.14: Bermuda Study-Level Stunting Rates and Certainty of Evidence Scores

Sources: Worldwide Historical Stunting Dataset.

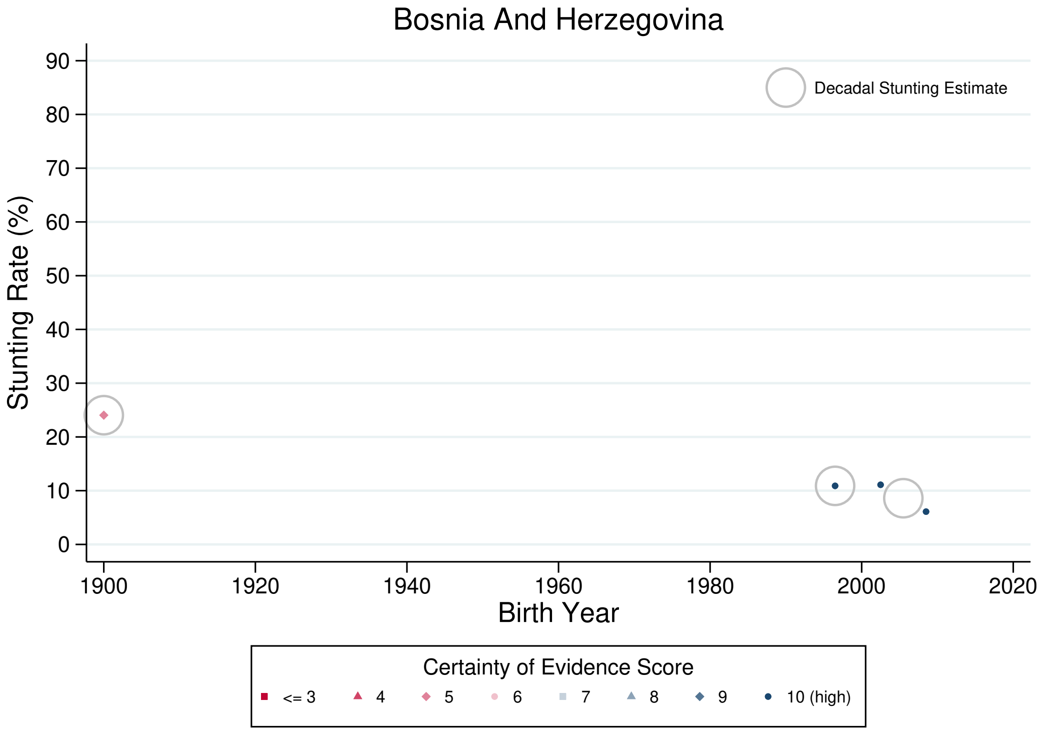

Figure O.15: Bosnia Study-Level Stunting Rates and Certainty of Evidence Scores

Sources: Worldwide Historical Stunting Dataset.

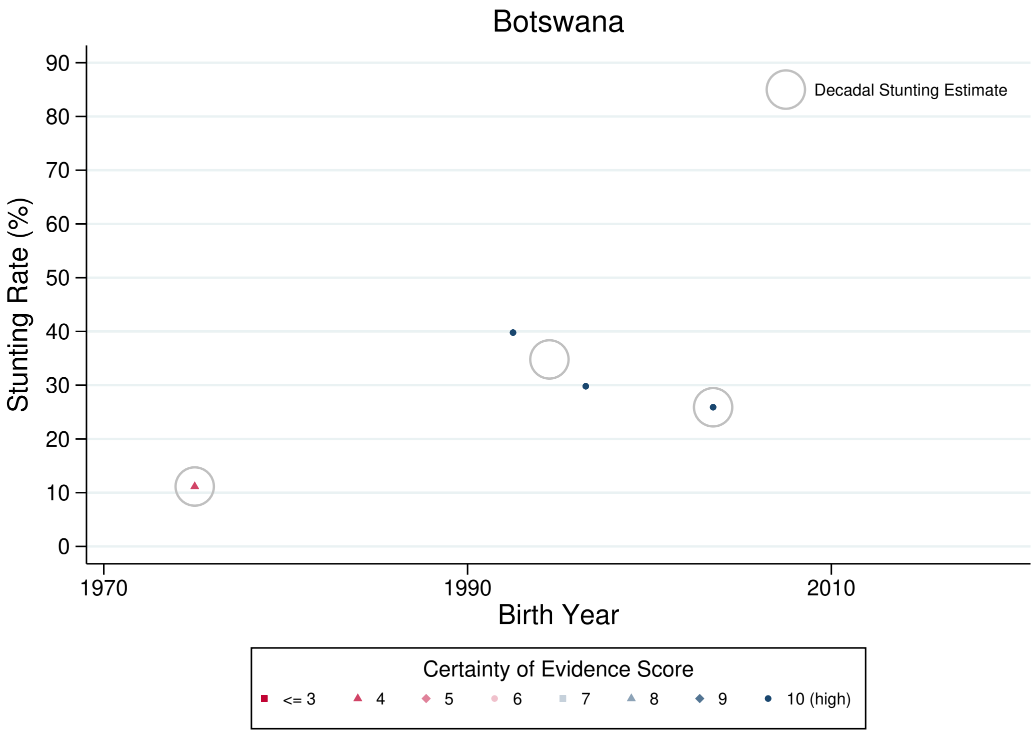

Figure O.16: Botswana Study-Level Stunting Rates and Certainty of Evidence Scores

Sources: Worldwide Historical Stunting Dataset.

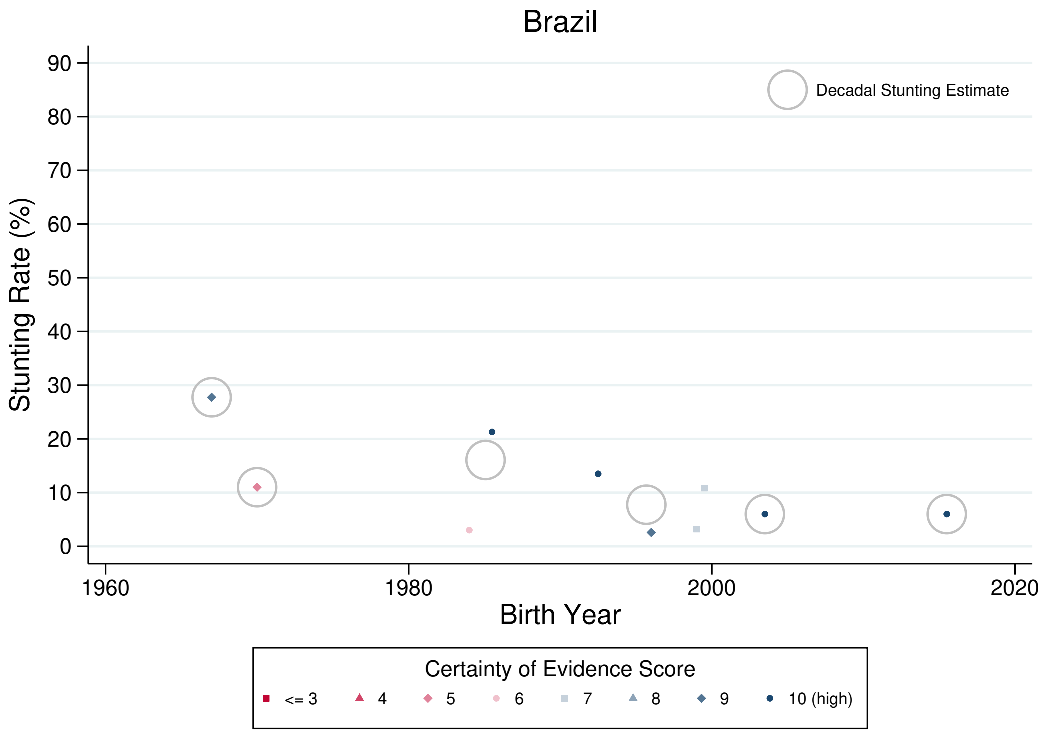

Figure O.17: Brazil Study-Level Stunting Rates and Certainty of Evidence Scores

Sources: Worldwide Historical Stunting Dataset.

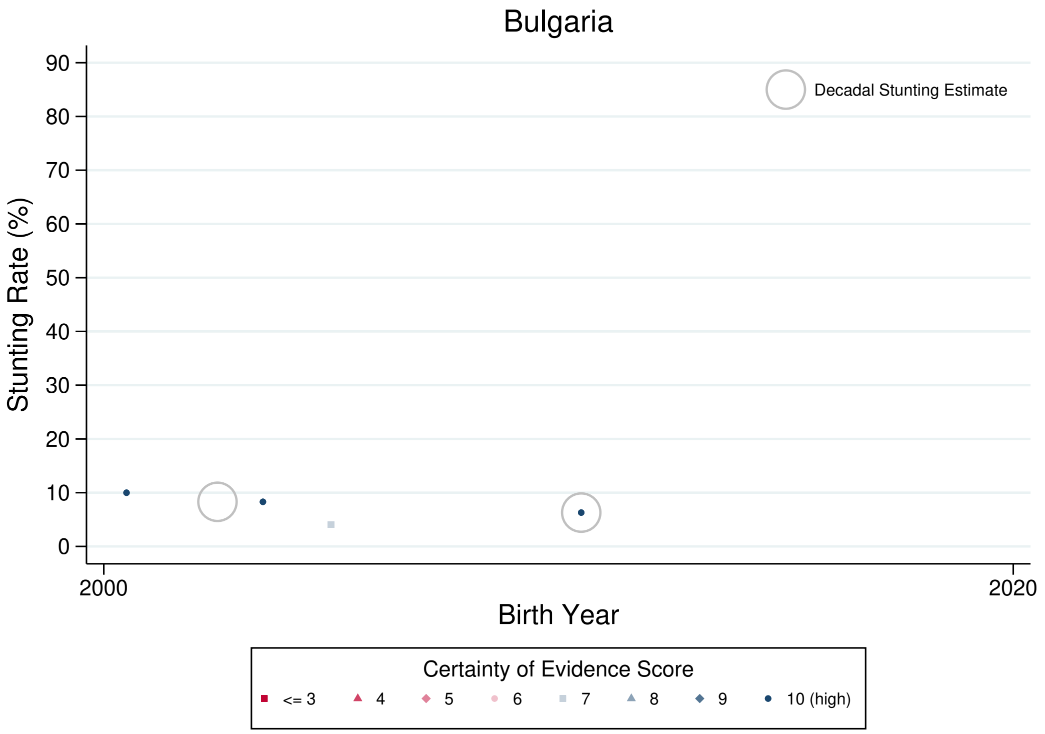

Figure O.18: Bulgaria Study-Level Stunting Rates and Certainty of Evidence Scores

Sources: Worldwide Historical Stunting Dataset.

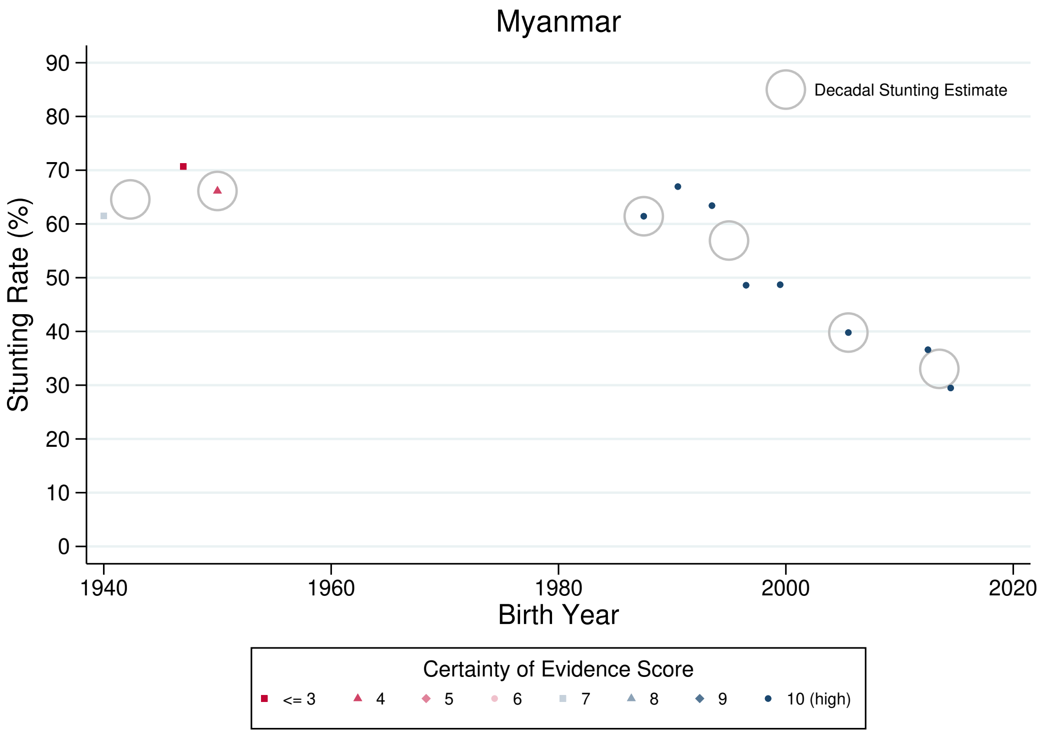

Figure O.19: Burma Study-Level Stunting Rates and Certainty of Evidence Scores

Sources: Worldwide Historical Stunting Dataset.

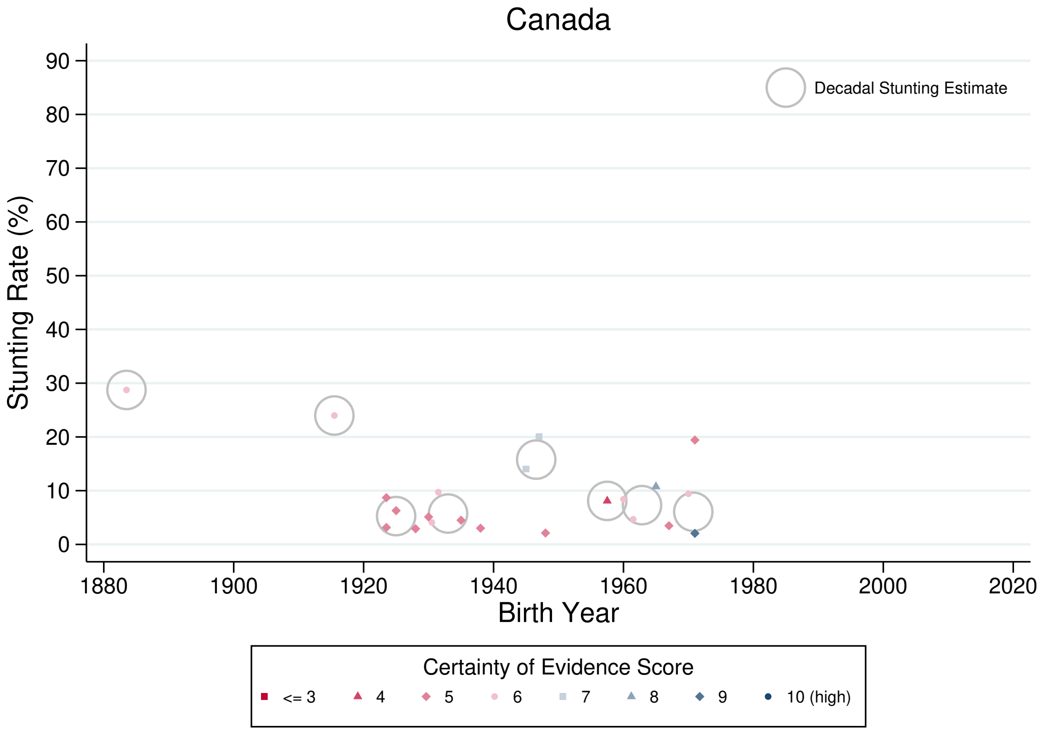

Figure O.20: Canada Study-Level Stunting Rates and Certainty of Evidence Scores

Sources: Worldwide Historical Stunting Dataset.

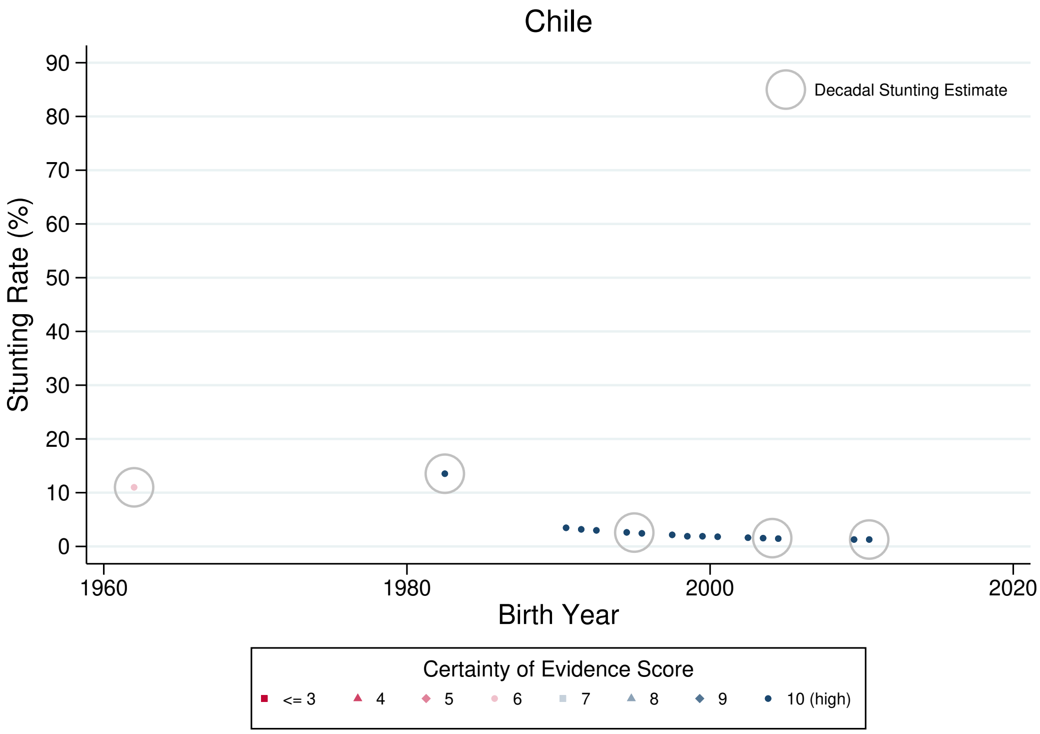

Figure O.21: Chile Study-Level Stunting Rates and Certainty of Evidence Scores

Sources: Worldwide Historical Stunting Dataset.

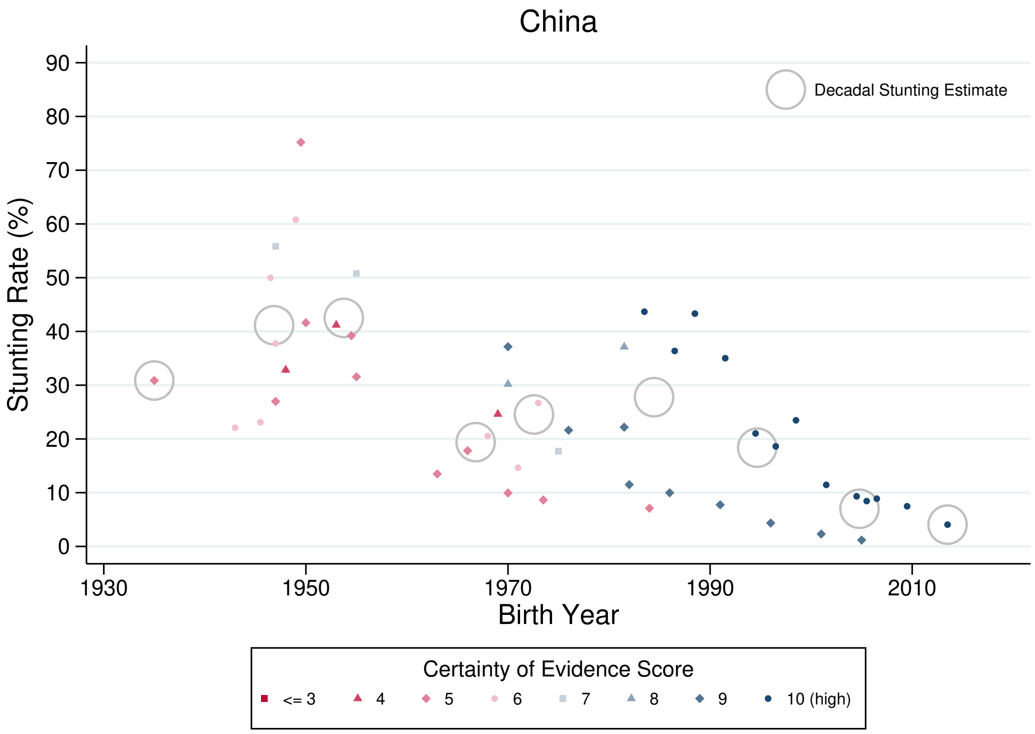

Figure O.22: China Study-Level Stunting Rates and Certainty of Evidence Scores

Sources: Worldwide Historical Stunting Dataset.

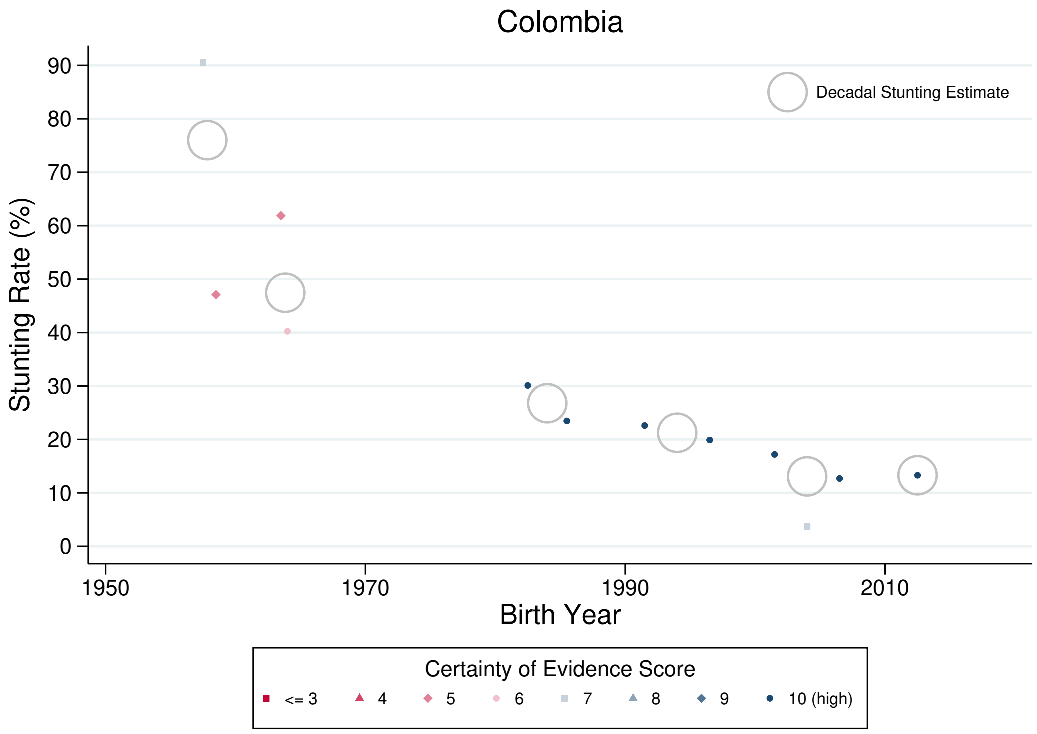

Figure O.23: Colombia Study-Level Stunting Rates and Certainty of Evidence Scores

Sources: Worldwide Historical Stunting Dataset.

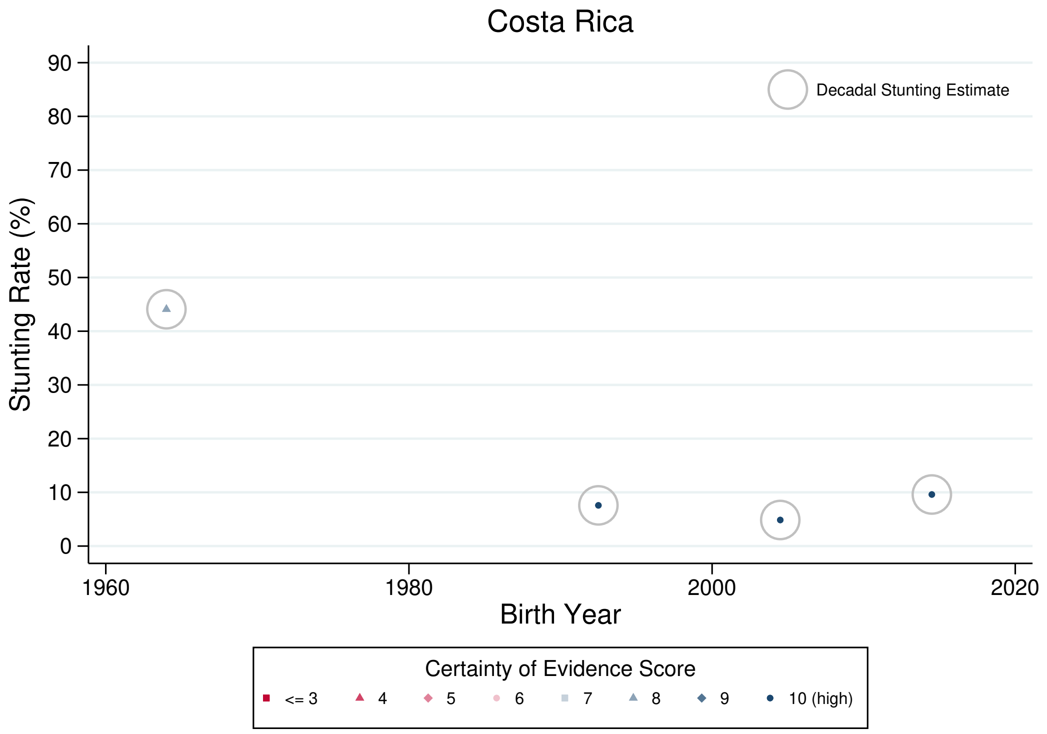

Figure O.24: Costarica Study-Level Stunting Rates and Certainty of Evidence Scores

Sources: Worldwide Historical Stunting Dataset.

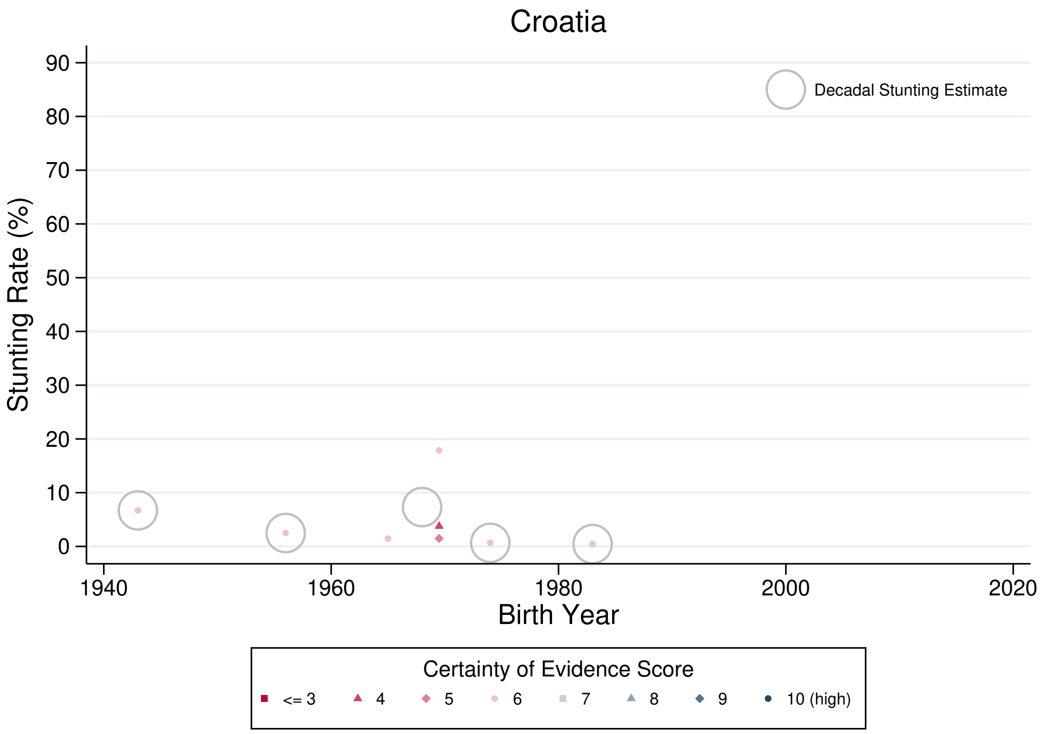

Figure O.25: Croatia Study-Level Stunting Rates and Certainty of Evidence Scores

Sources: Worldwide Historical Stunting Dataset.

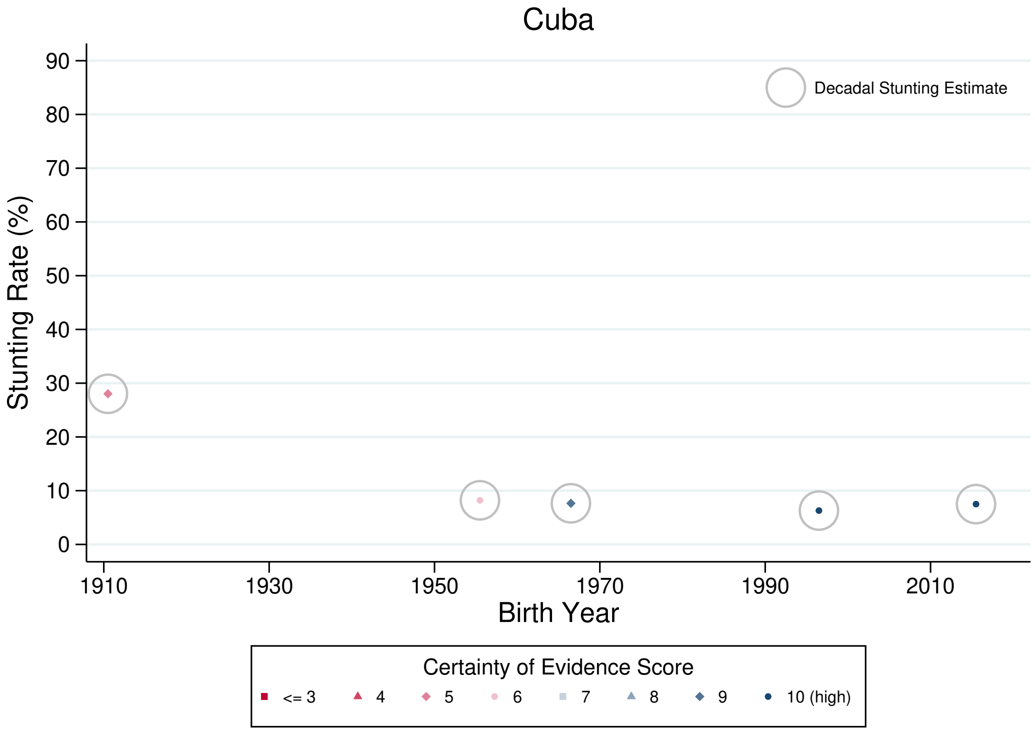

Figure O.26: Cuba Study-Level Stunting Rates and Certainty of Evidence Scores

Sources: Worldwide Historical Stunting Dataset.

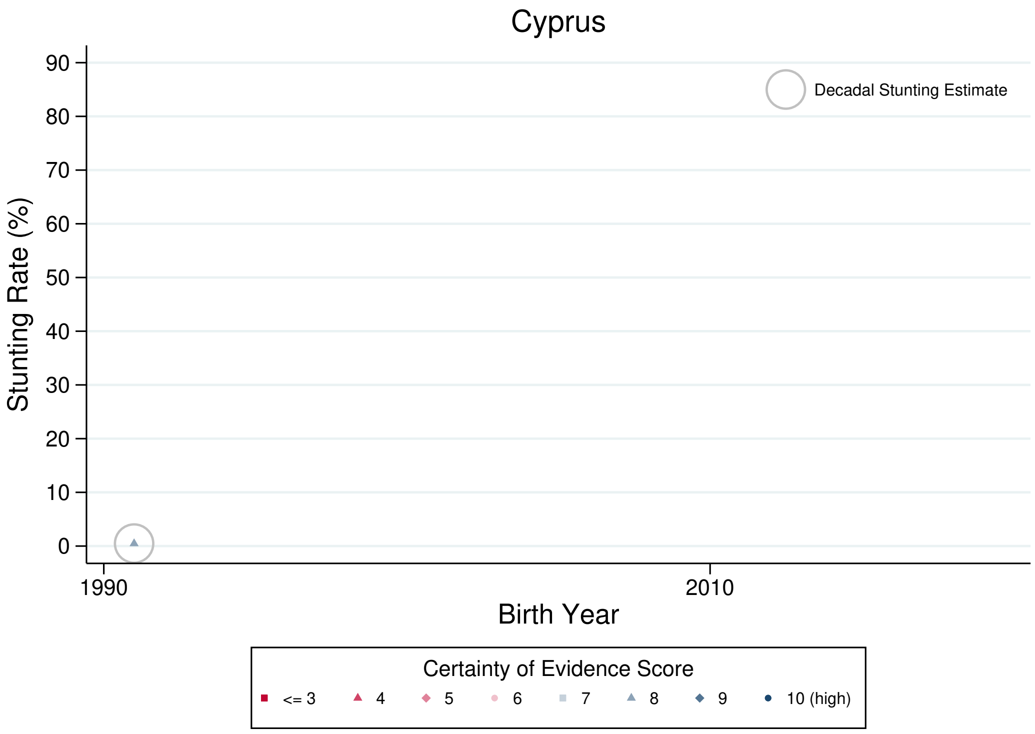

Figure O.27: Cyprus Study-Level Stunting Rates and Certainty of Evidence Scores

Sources: Worldwide Historical Stunting Dataset.

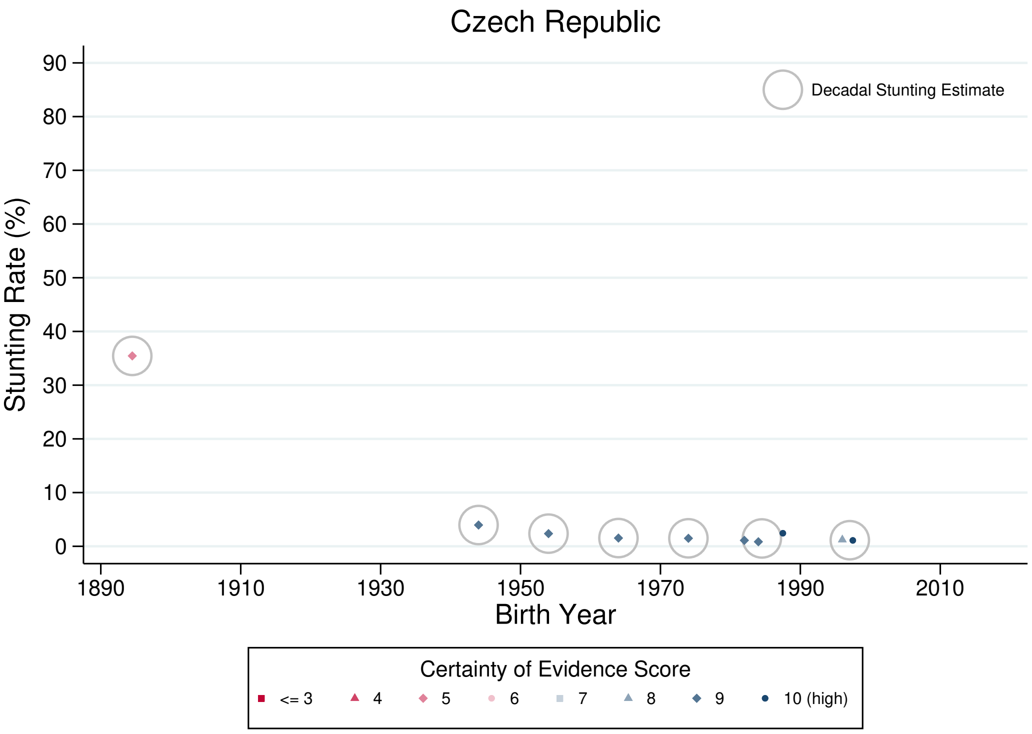

Figure O.28: Czech Republic Study-Level Stunting Rates and Certainty of Evidence Scores

Sources: Worldwide Historical Stunting Dataset.

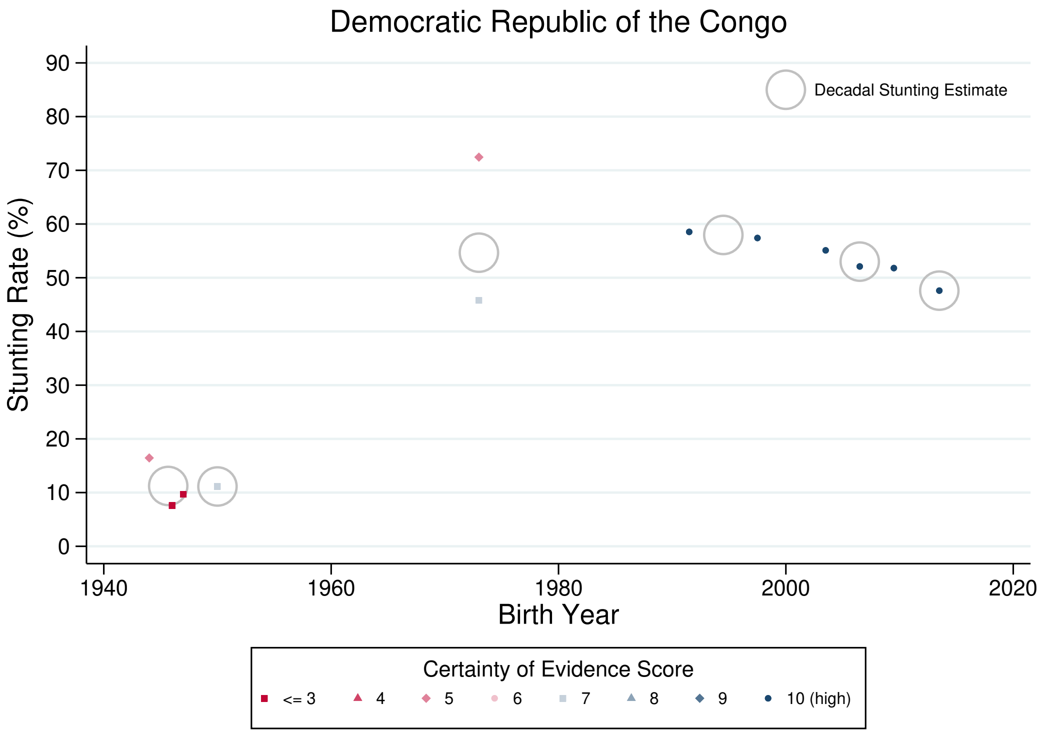

Figure O.29: Democratic Republic of the Congo Study-Level Stunting Rates and Certainty of Evidence Scores

Sources: Worldwide Historical Stunting Dataset.

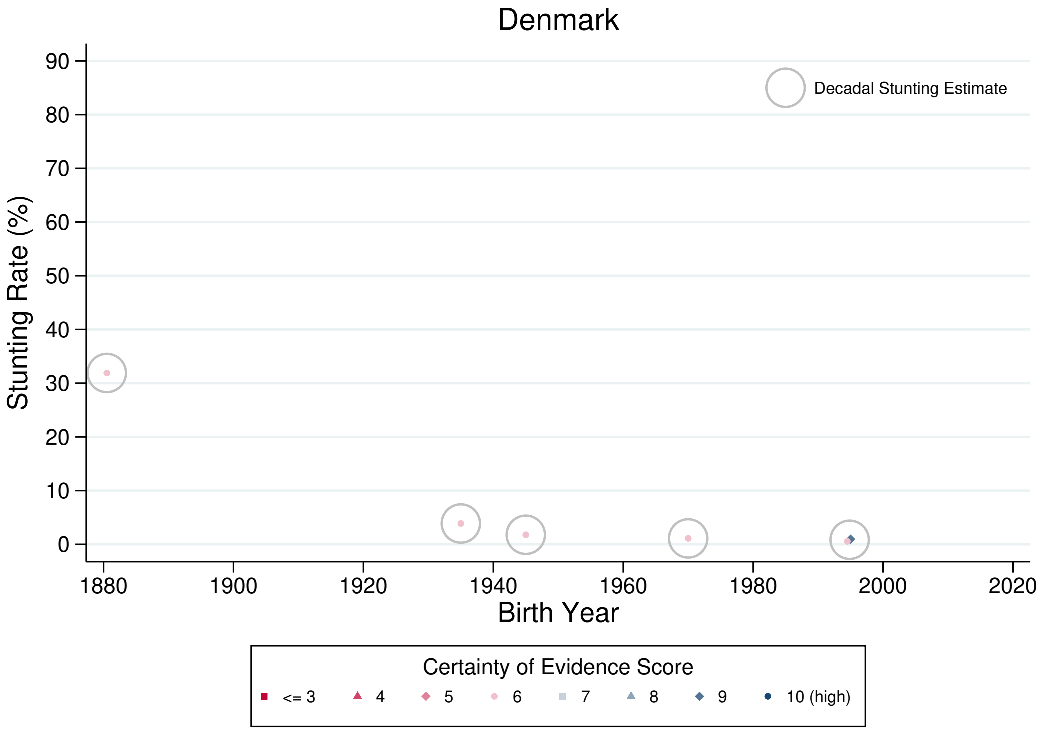

Figure O.30: Denmark Study-Level Stunting Rates and Certainty of Evidence Scores

Sources: Worldwide Historical Stunting Dataset.

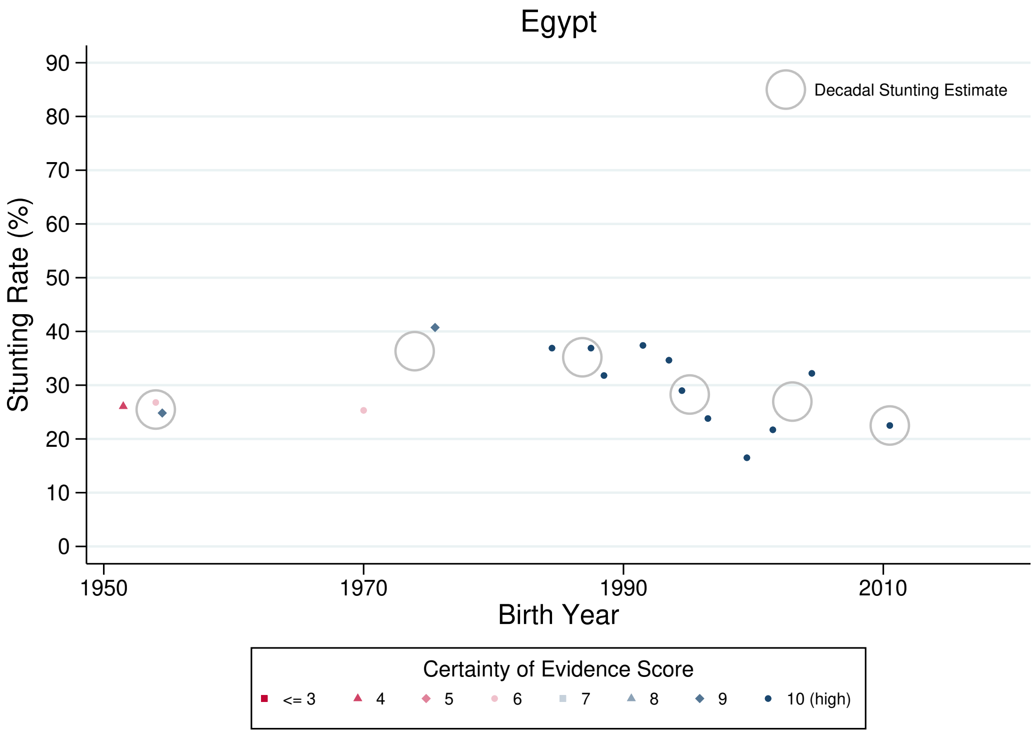

Figure O.31: Egypt Study-Level Stunting Rates and Certainty of Evidence Scores

Sources: Worldwide Historical Stunting Dataset.

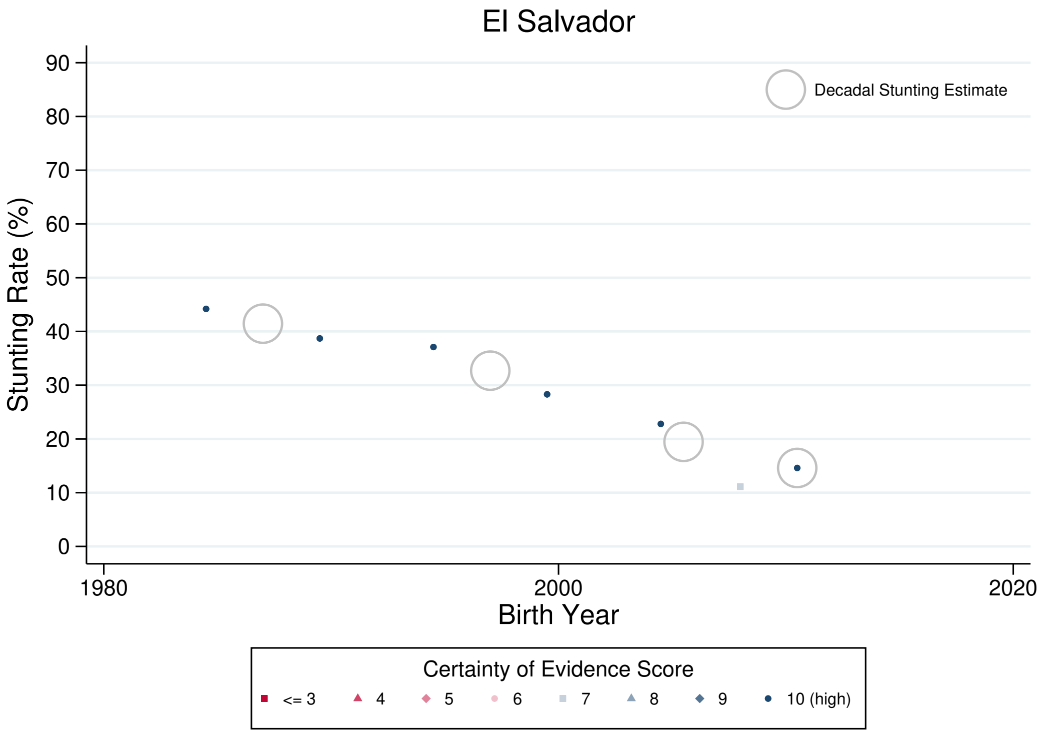

Figure O.32: El Salvador Study-Level Stunting Rates and Certainty of Evidence Scores

Sources: Worldwide Historical Stunting Dataset.

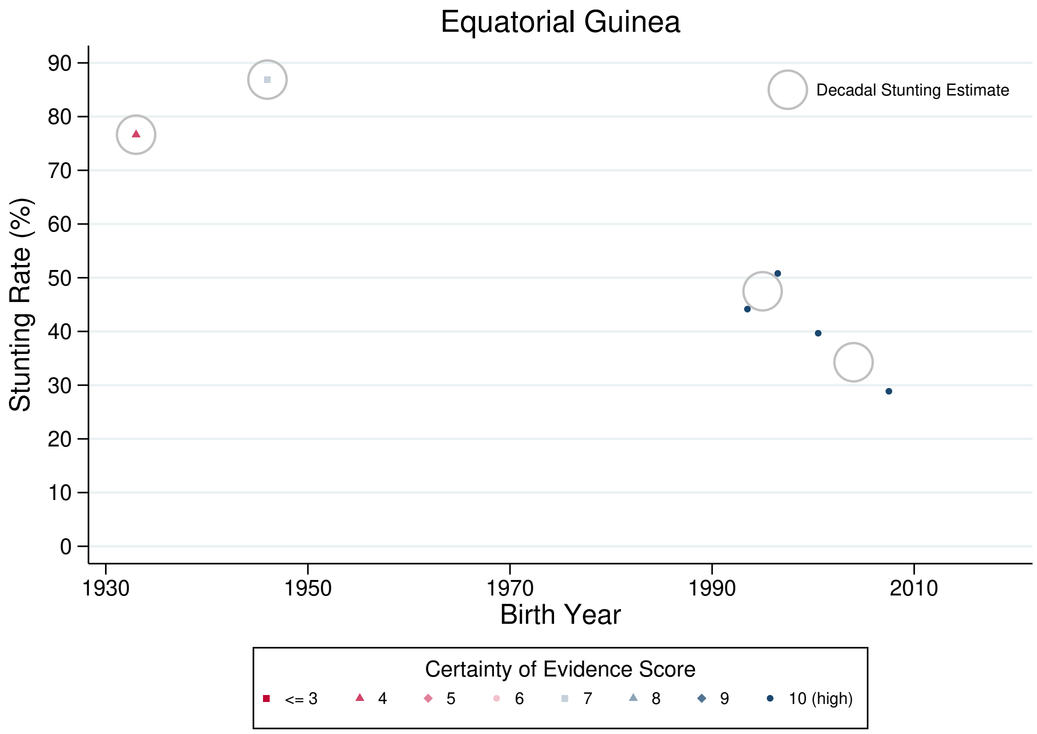

Figure O.33: Equatorial Guinea Study-Level Stunting Rates and Certainty of Evidence Scores

Sources: Worldwide Historical Stunting Dataset.

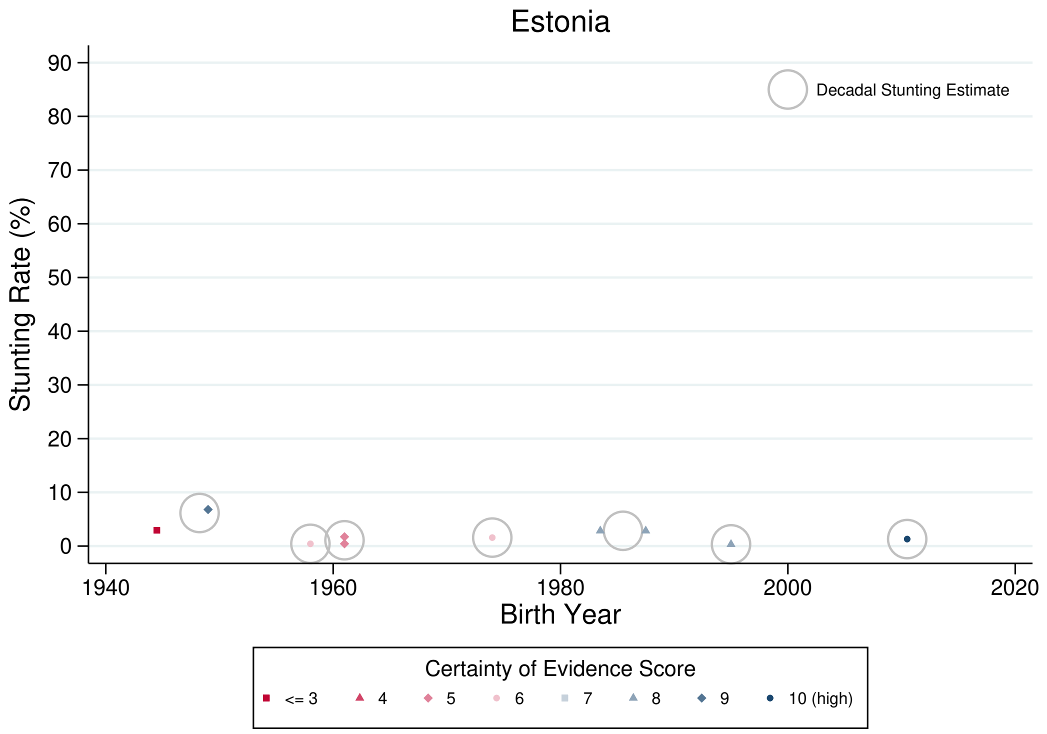

Figure O.34: Estonia Study-Level Stunting Rates and Certainty of Evidence Scores

Sources: Worldwide Historical Stunting Dataset.

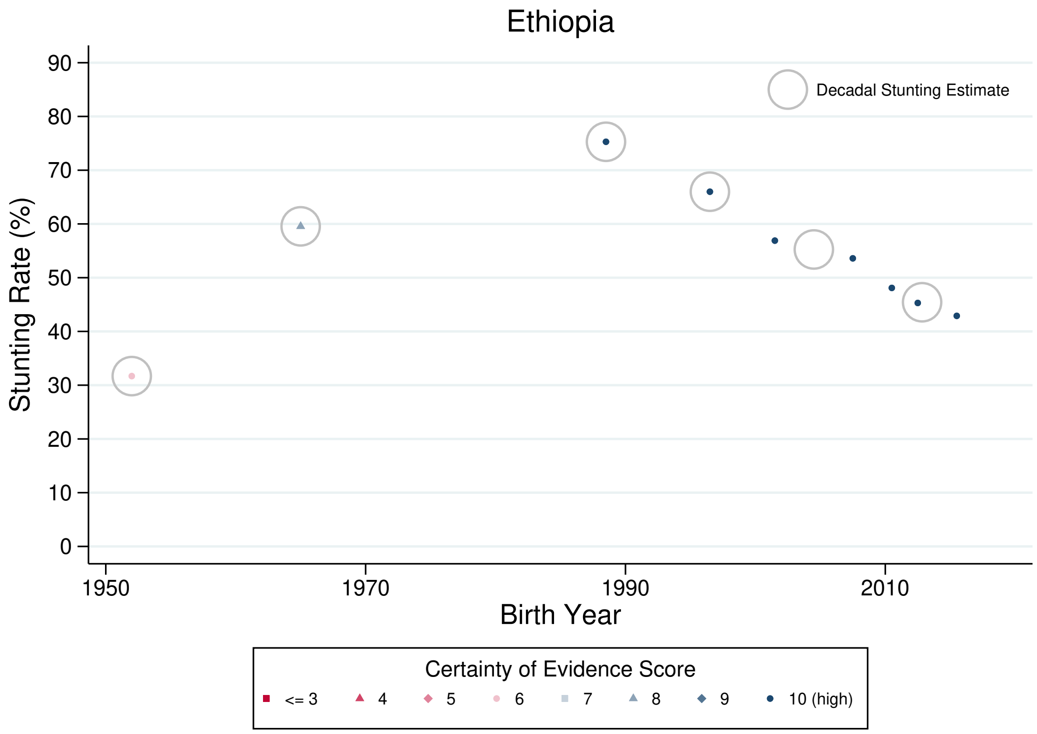

Figure O.35: Ethiopia Study-Level Stunting Rates and Certainty of Evidence Scores

Sources: Worldwide Historical Stunting Dataset.

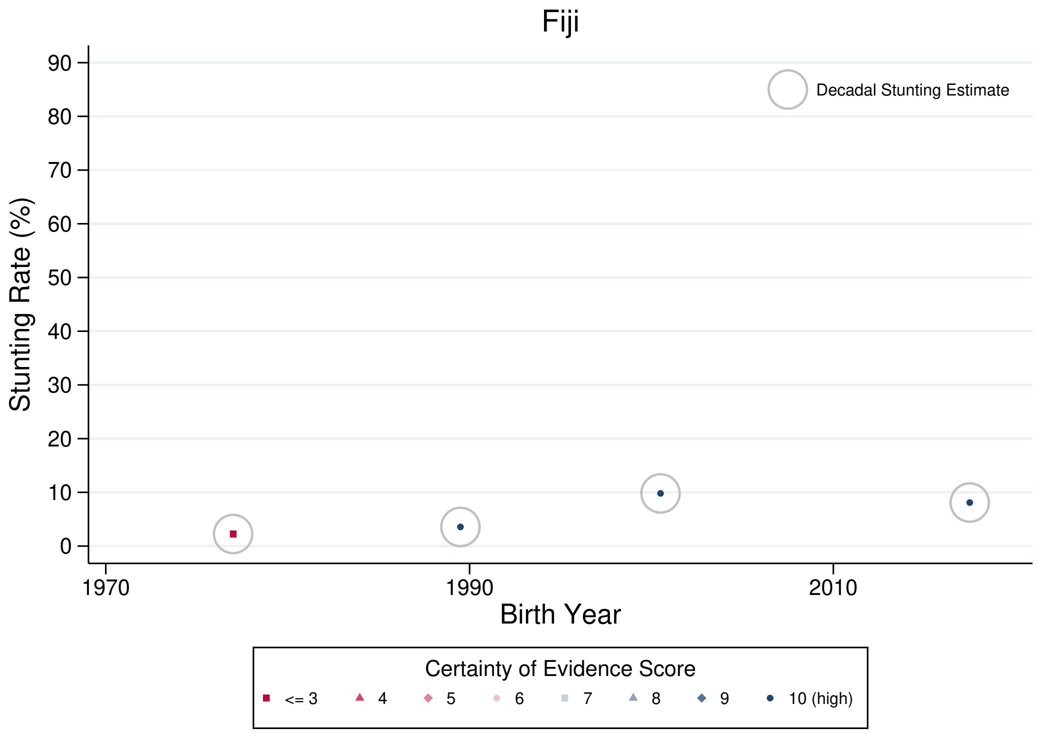

Figure O.36: Fiji Study-Level Stunting Rates and Certainty of Evidence Scores

Sources: Worldwide Historical Stunting Dataset.

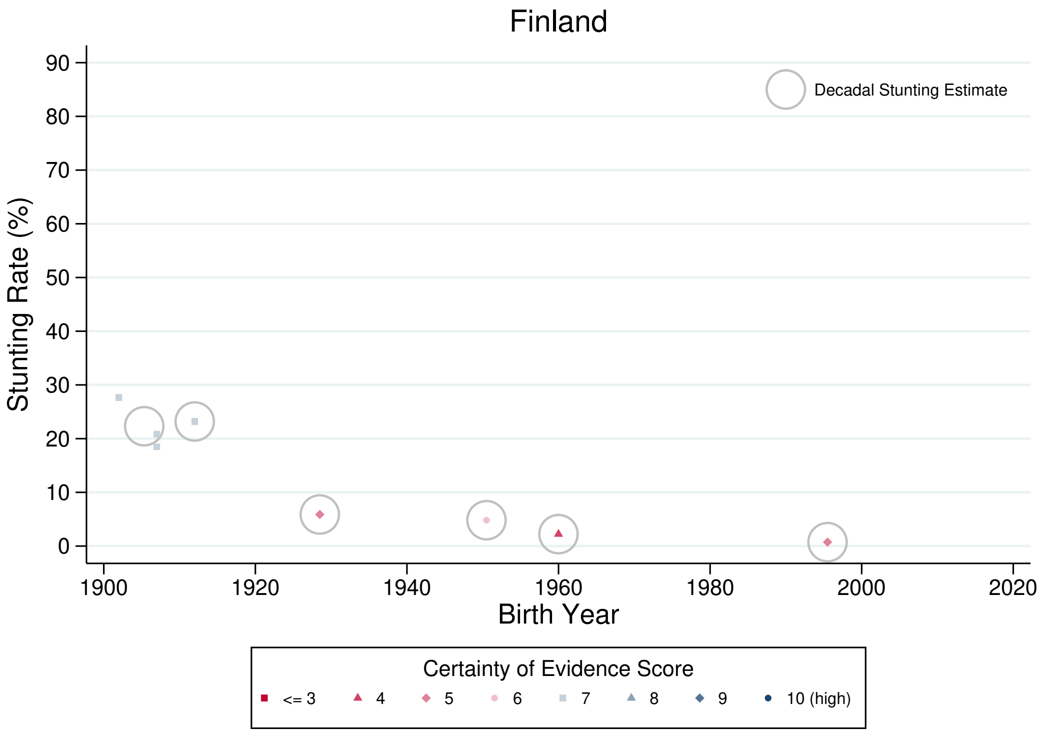

Figure O.37: Finland Study-Level Stunting Rates and Certainty of Evidence Scores

Sources: Worldwide Historical Stunting Dataset.

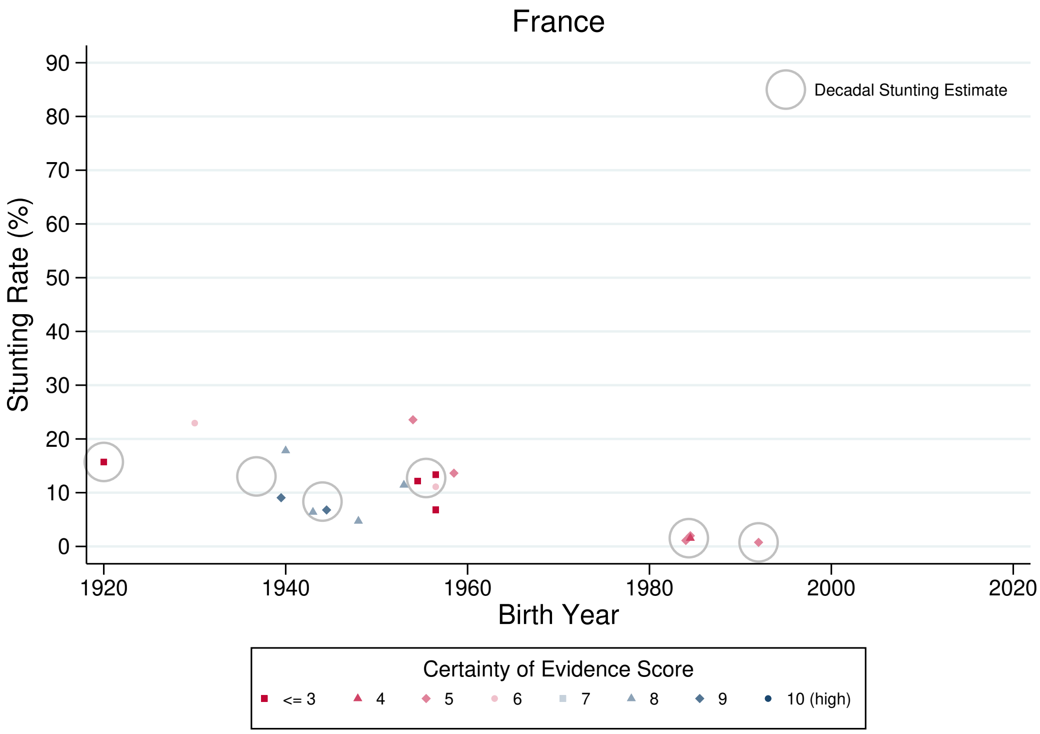

Figure O.38: France Study-Level Stunting Rates and Certainty of Evidence Scores

Sources: Worldwide Historical Stunting Dataset.

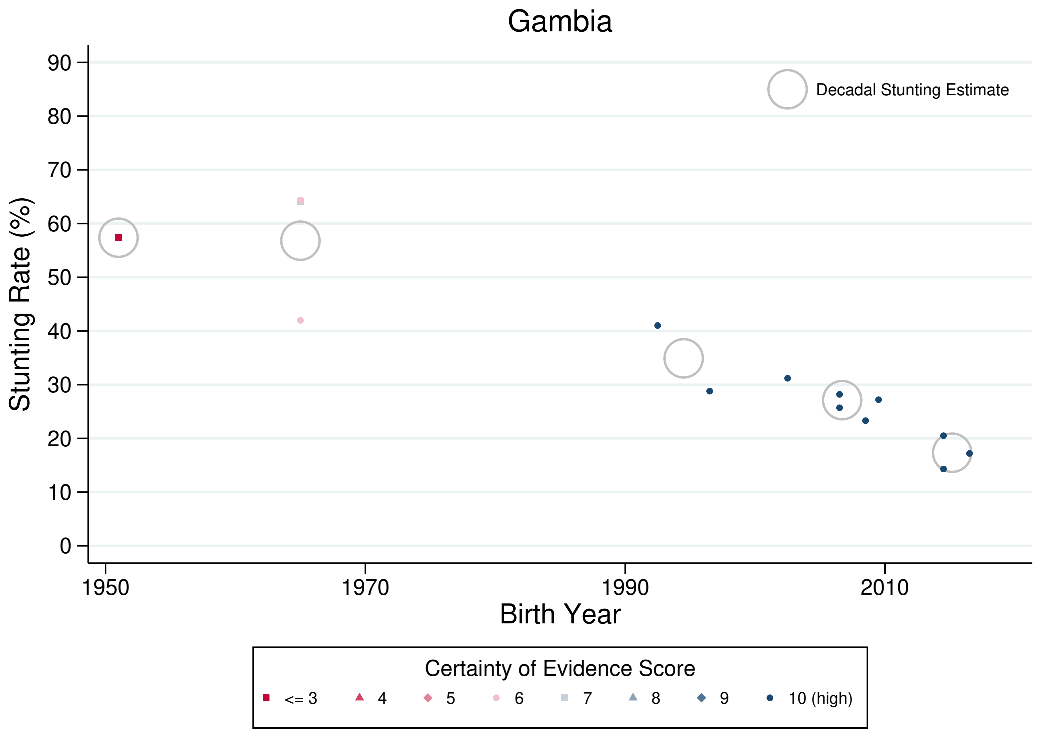

Figure O.39: Gambia Study-Level Stunting Rates and Certainty of Evidence Scores

Sources: Worldwide Historical Stunting Dataset.

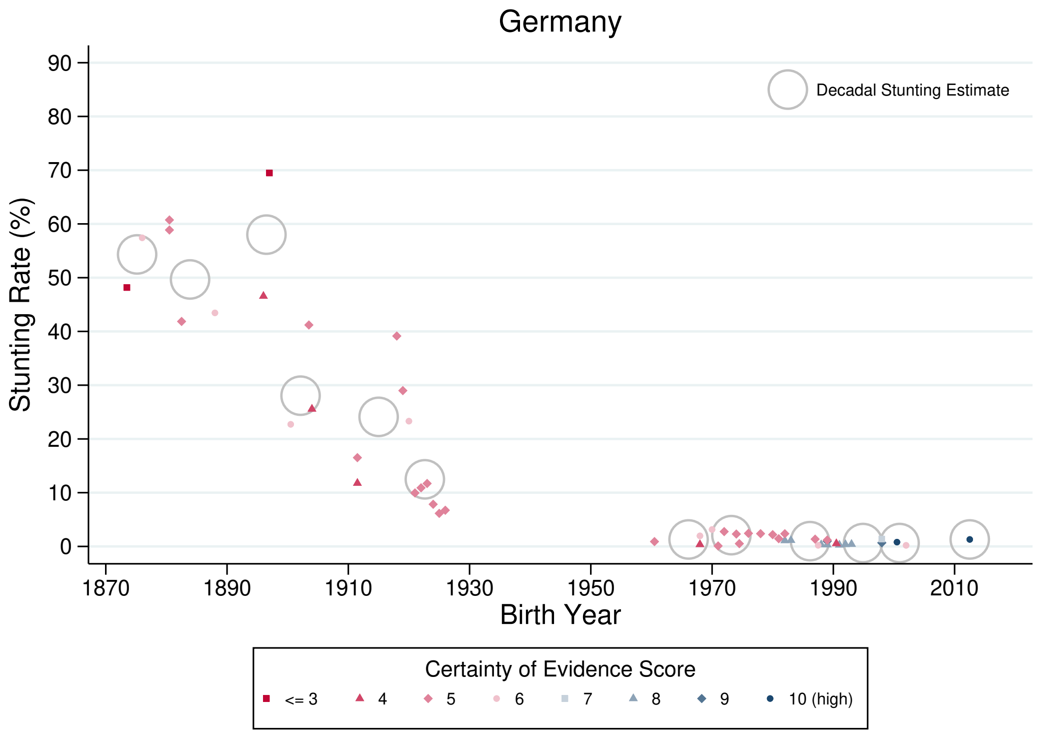

Figure O.40: Germany Study-Level Stunting Rates and Certainty of Evidence Scores

Sources: Worldwide Historical Stunting Dataset.

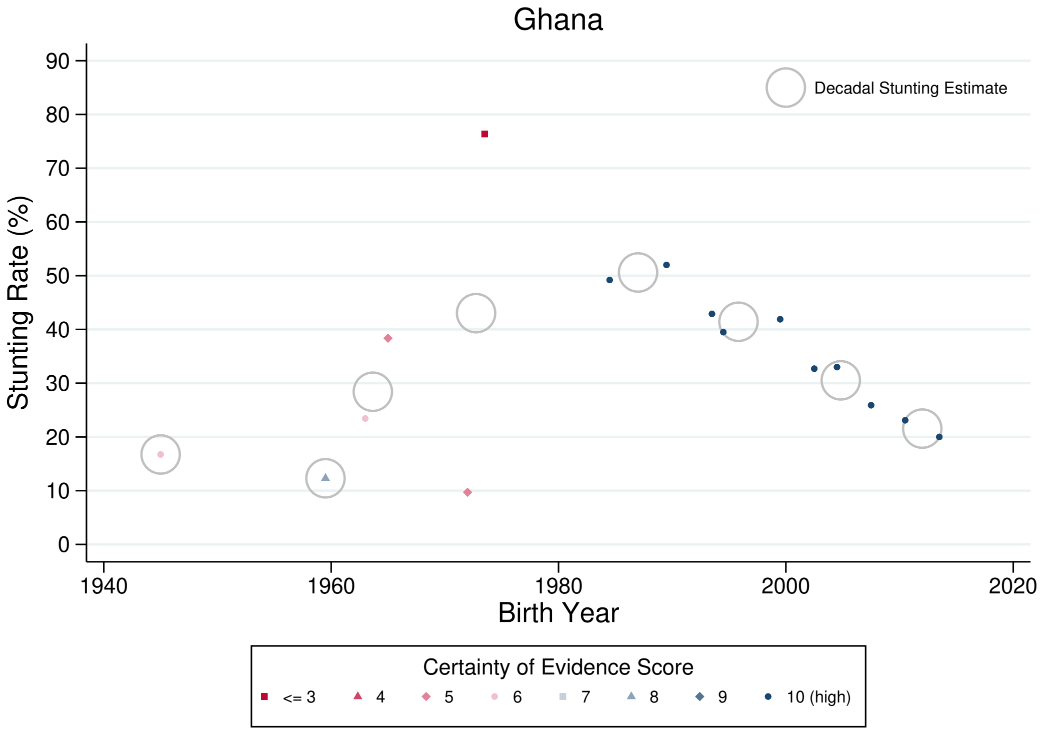

Figure O.41: Ghana Study-Level Stunting Rates and Certainty of Evidence Scores

Sources: Worldwide Historical Stunting Dataset.

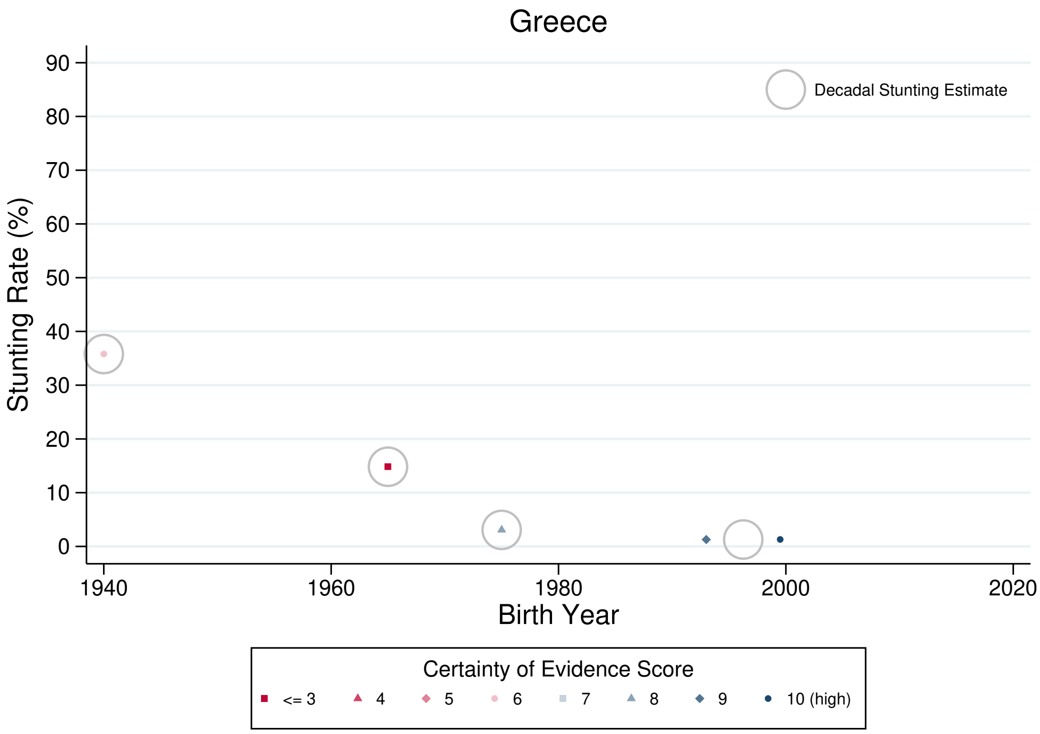

Figure O.42: Greece Study-Level Stunting Rates and Certainty of Evidence Scores

Sources: Worldwide Historical Stunting Dataset.

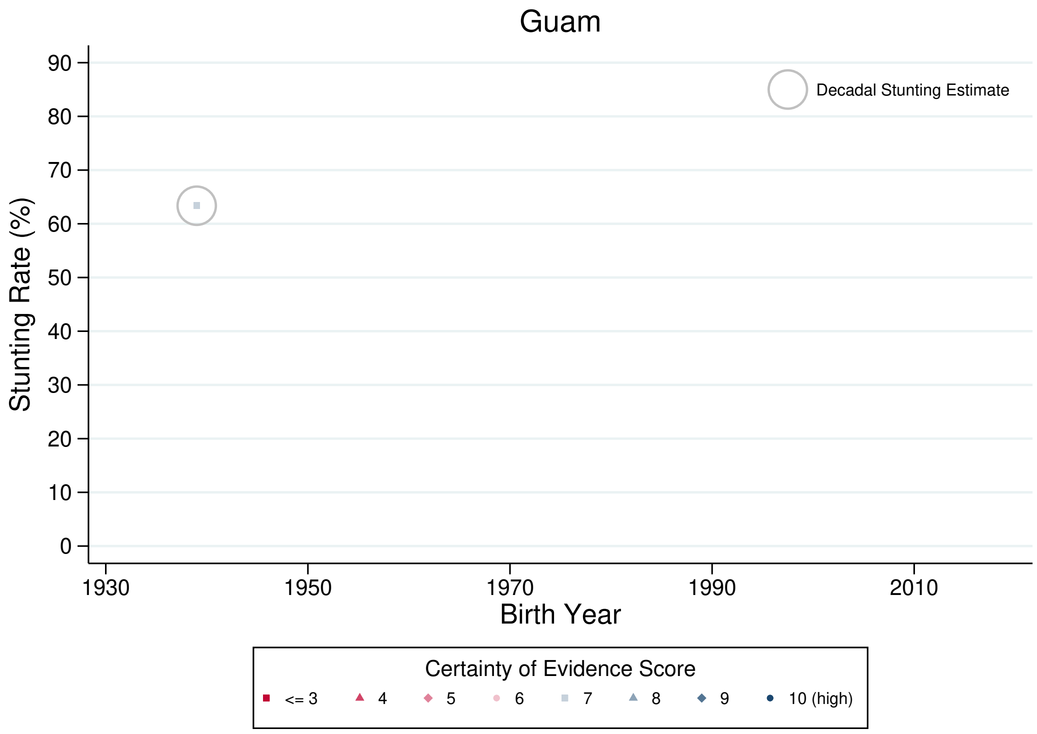

Figure O.43: Guam Study-Level Stunting Rates and Certainty of Evidence Scores

Sources: Worldwide Historical Stunting Dataset.

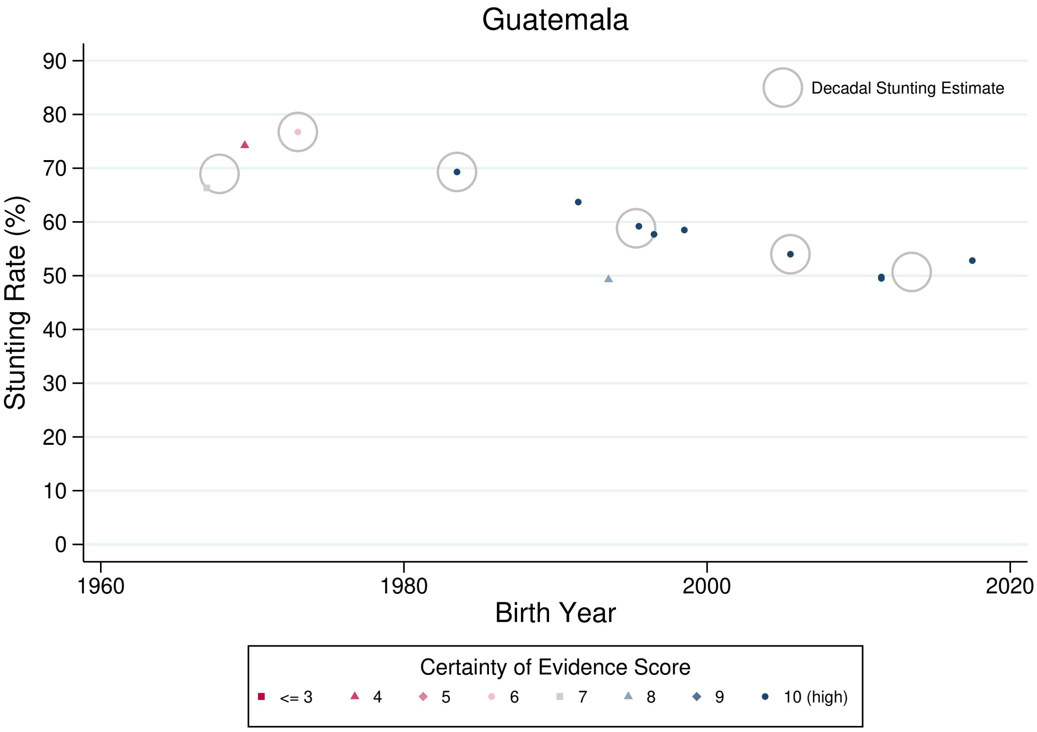

Figure O.44: Guatemala Study-Level Stunting Rates and Certainty of Evidence Scores

Sources: Worldwide Historical Stunting Dataset.

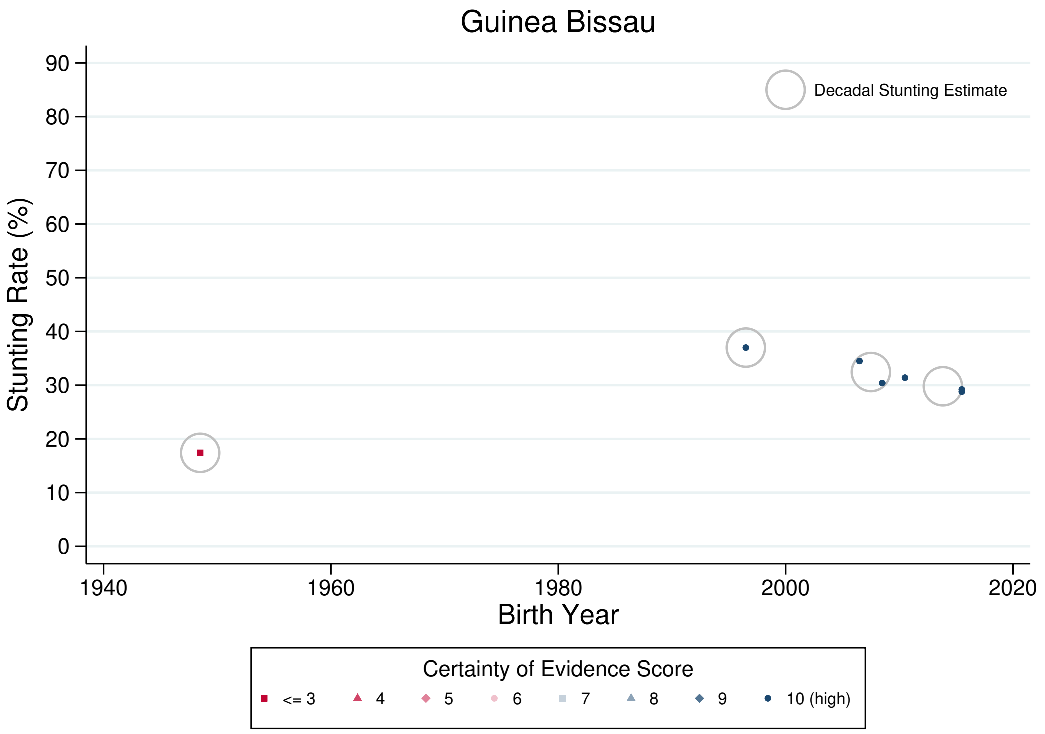

Figure O.45: Guinea Bissau Study-Level Stunting Rates and Certainty of Evidence Scores

Sources: Worldwide Historical Stunting Dataset.

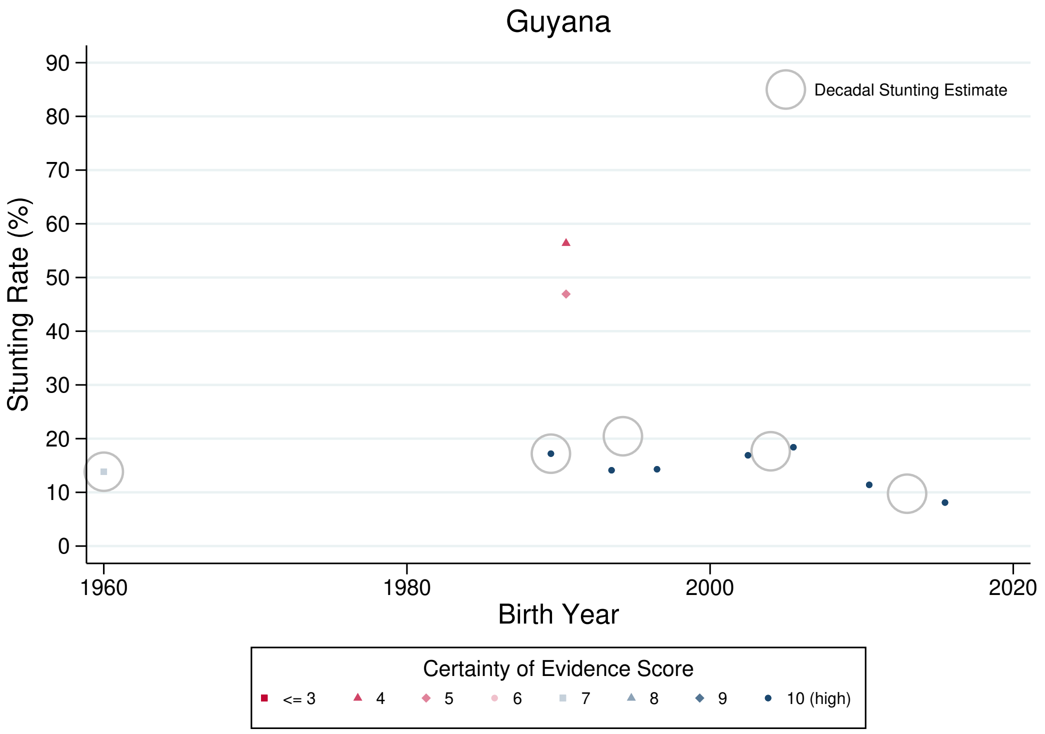

Figure O.46: Guyana Study-Level Stunting Rates and Certainty of Evidence Scores

Sources: Worldwide Historical Stunting Dataset.

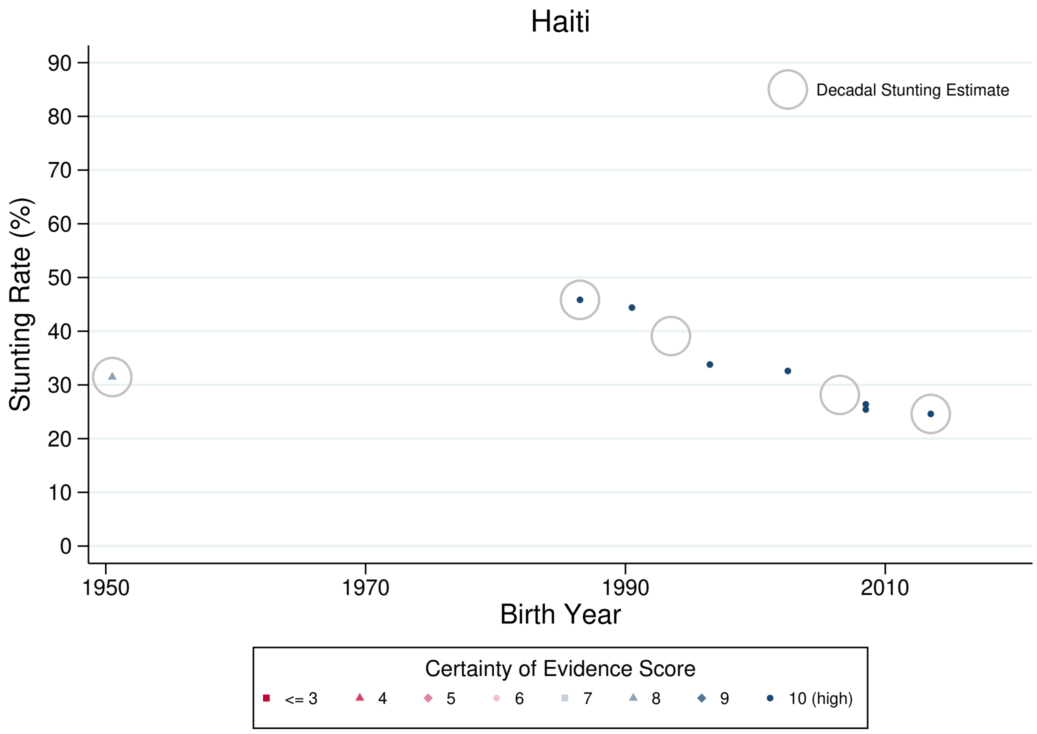

Figure O.47: Haiti Study-Level Stunting Rates and Certainty of Evidence Scores

Sources: Worldwide Historical Stunting Dataset.

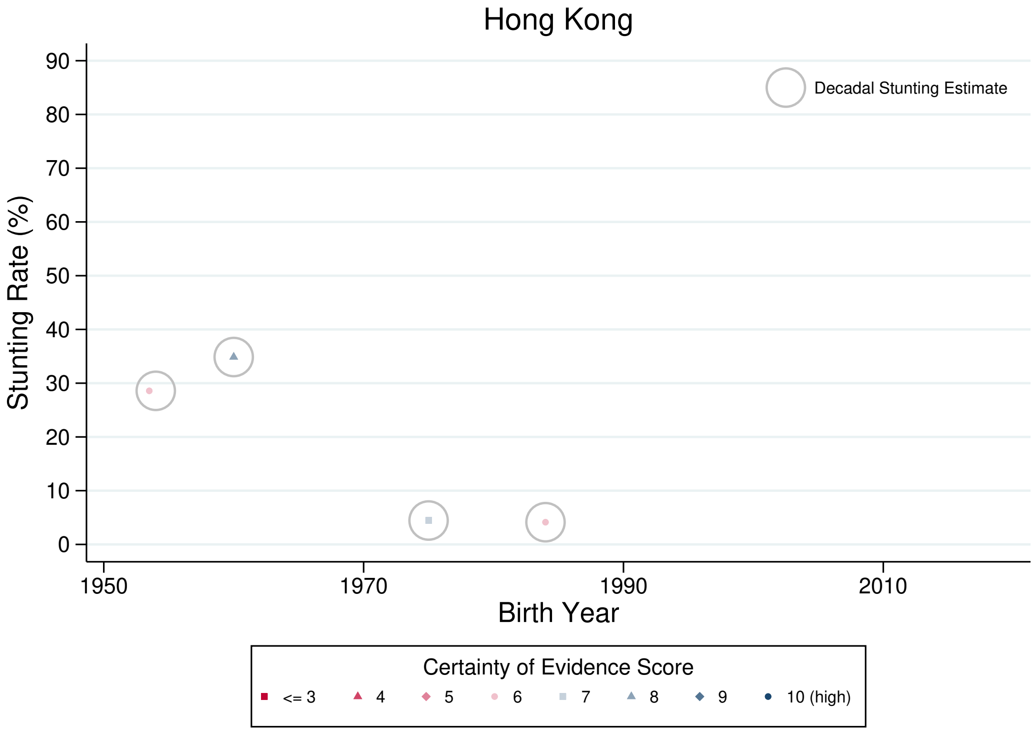

Figure O.48: Hong Kong Study-Level Stunting Rates and Certainty of Evidence Scores

Sources: Worldwide Historical Stunting Dataset.

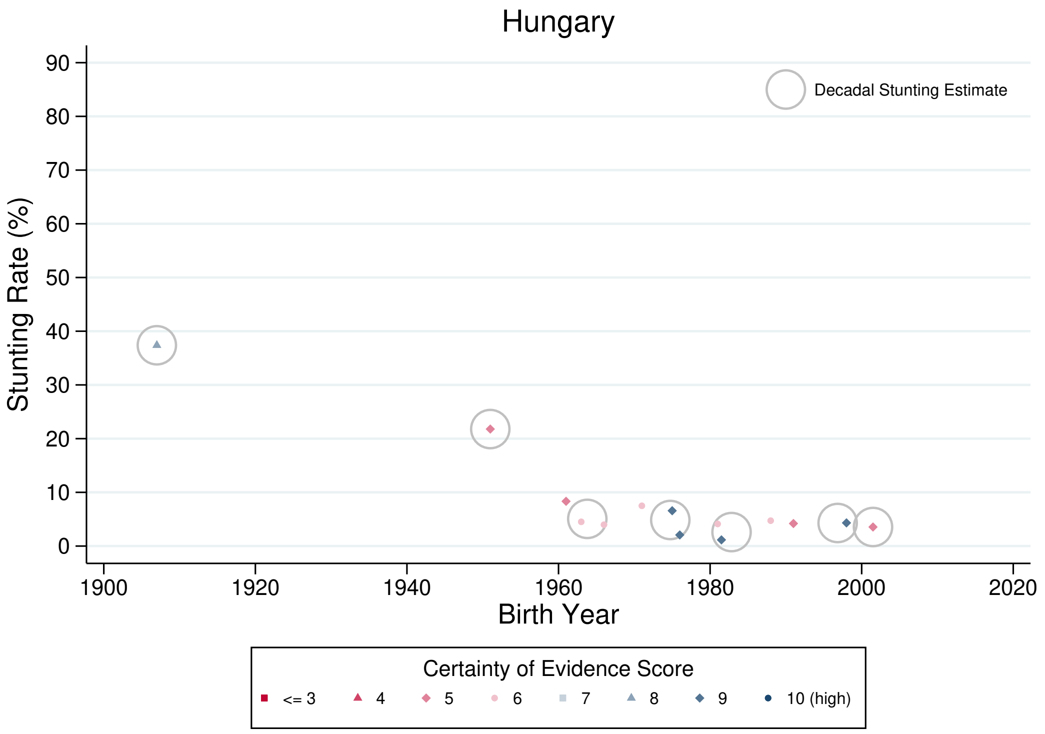

Figure O.49: Hungary Study-Level Stunting Rates and Certainty of Evidence Scores

Sources: Worldwide Historical Stunting Dataset.

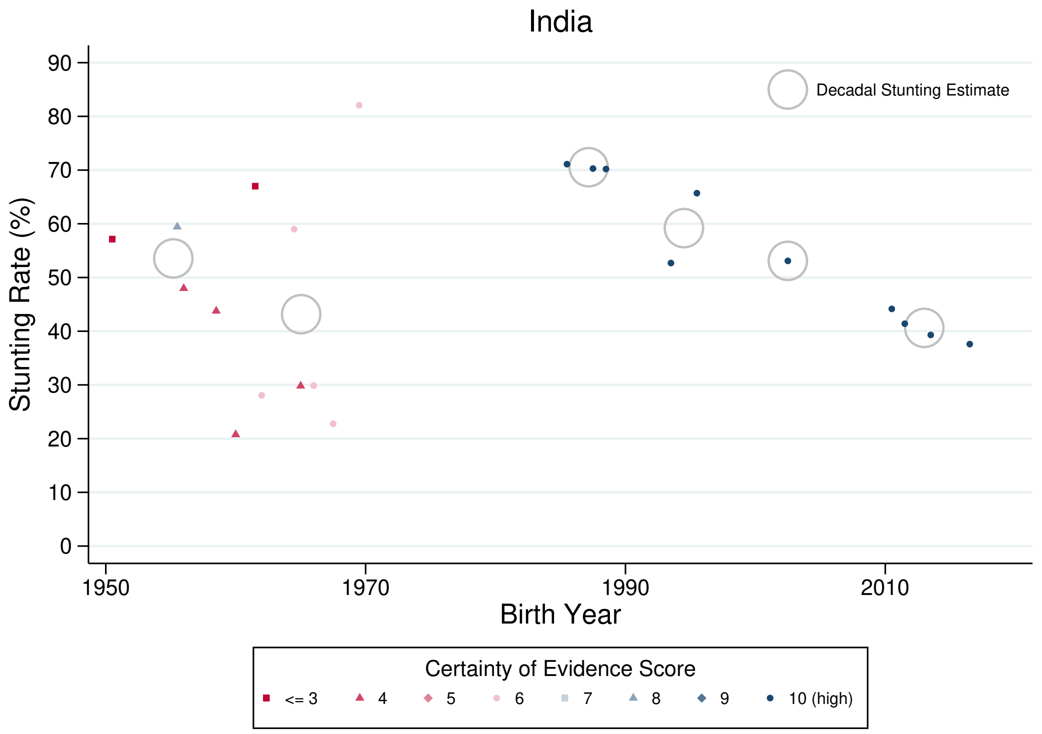

Figure O.50: India Study-Level Stunting Rates and Certainty of Evidence Scores

Sources: Worldwide Historical Stunting Dataset.

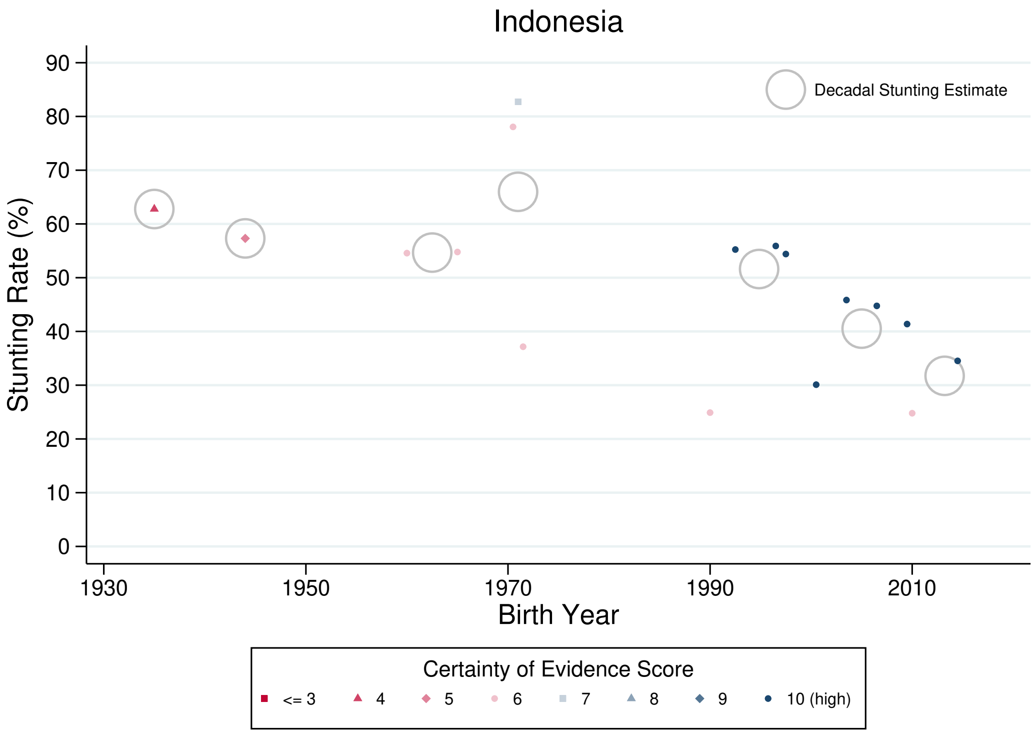

Figure O.51: Indonesia Study-Level Stunting Rates and Certainty of Evidence Scores

Sources: Worldwide Historical Stunting Dataset.

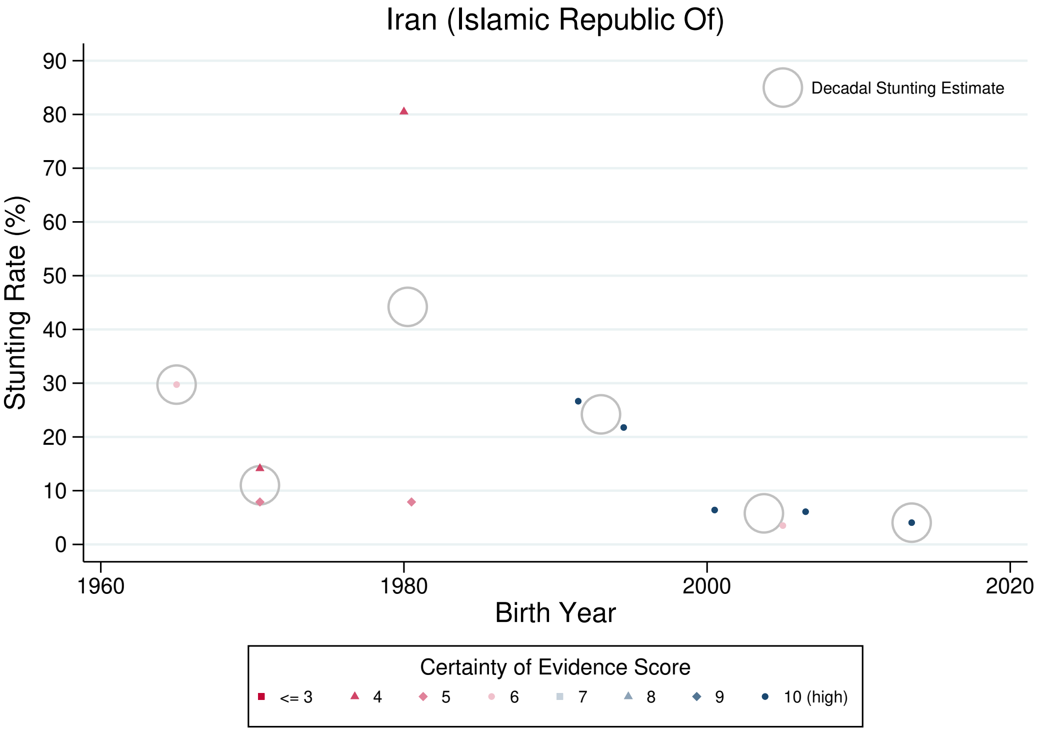

Figure O.52: Iran Study-Level Stunting Rates and Certainty of Evidence Scores

Sources: Worldwide Historical Stunting Dataset.

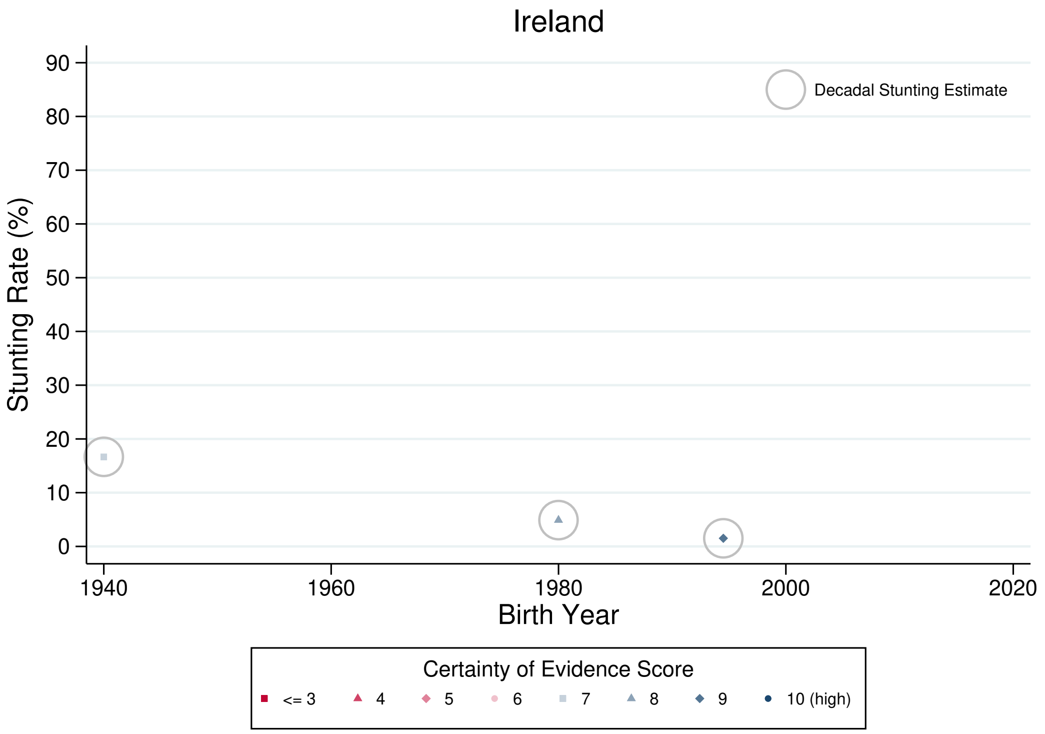

Figure O.53: Ireland Study-Level Stunting Rates and Certainty of Evidence Scores

Sources: Worldwide Historical Stunting Dataset.

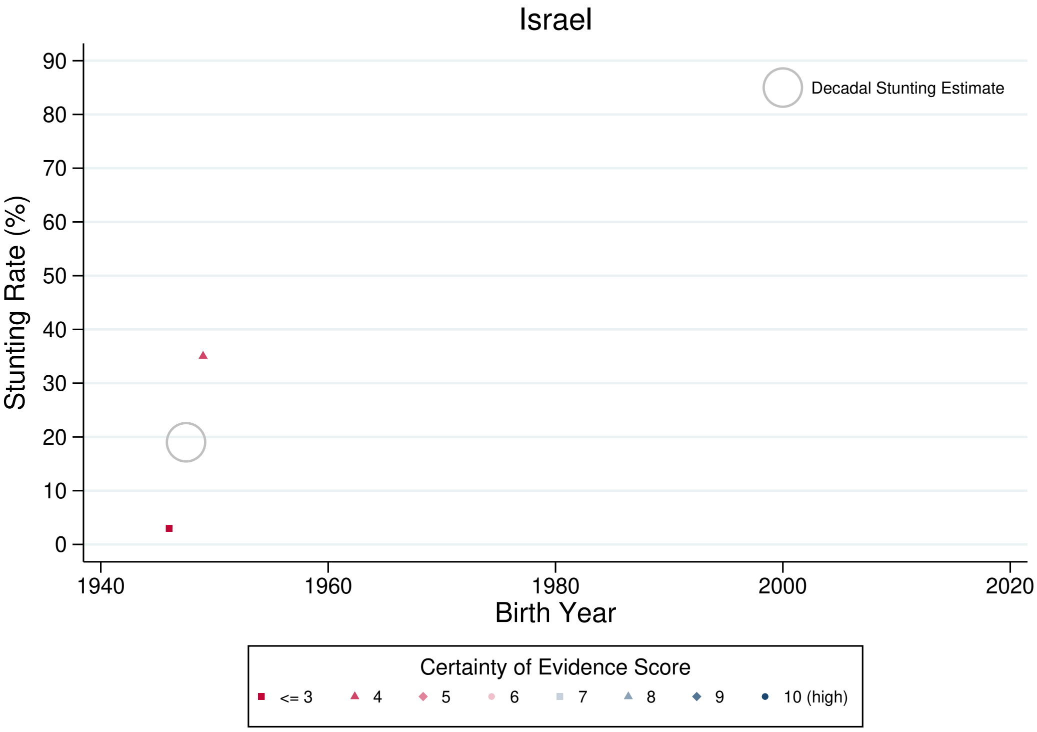

Figure O.54: Israel Study-Level Stunting Rates and Certainty of Evidence Scores

Sources: Worldwide Historical Stunting Dataset.

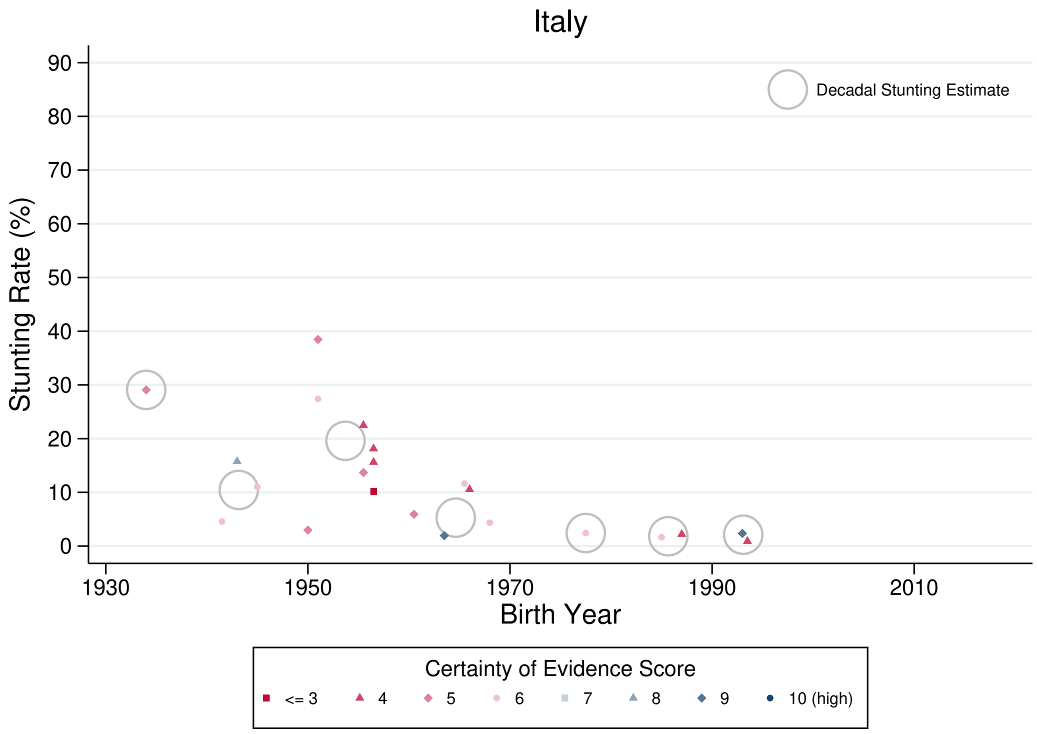

Figure O.55: Italy Study-Level Stunting Rates and Certainty of Evidence Scores

Sources: Worldwide Historical Stunting Dataset.

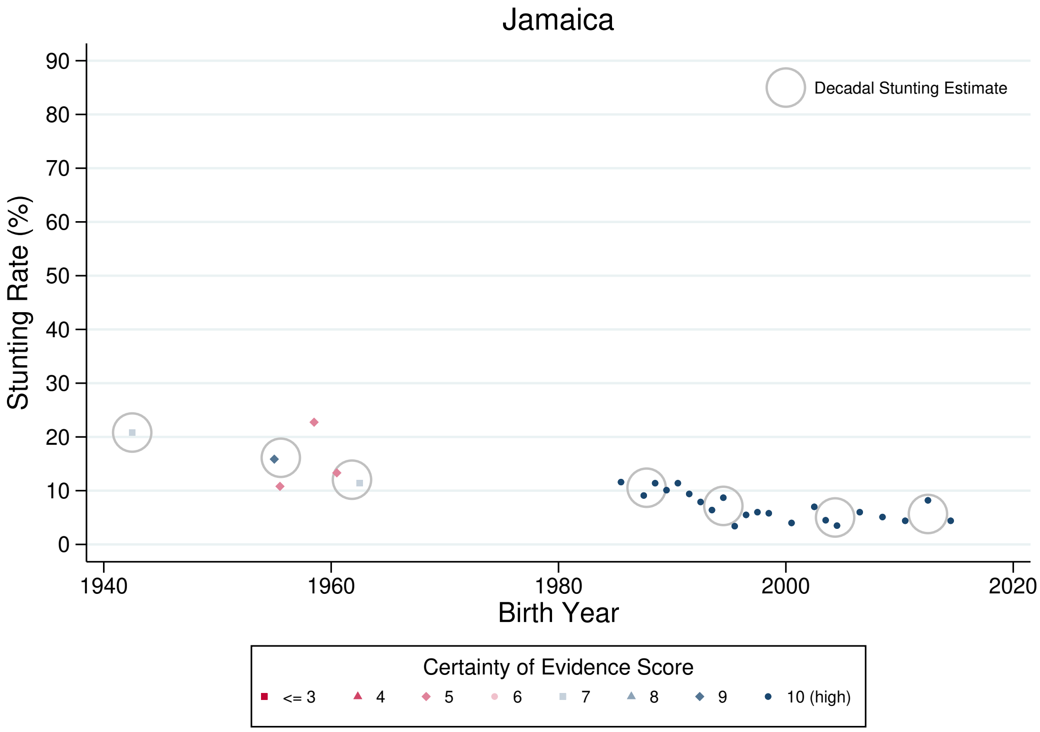

Figure O.56: Jamaica Study-Level Stunting Rates and Certainty of Evidence Scores

Sources: Worldwide Historical Stunting Dataset.

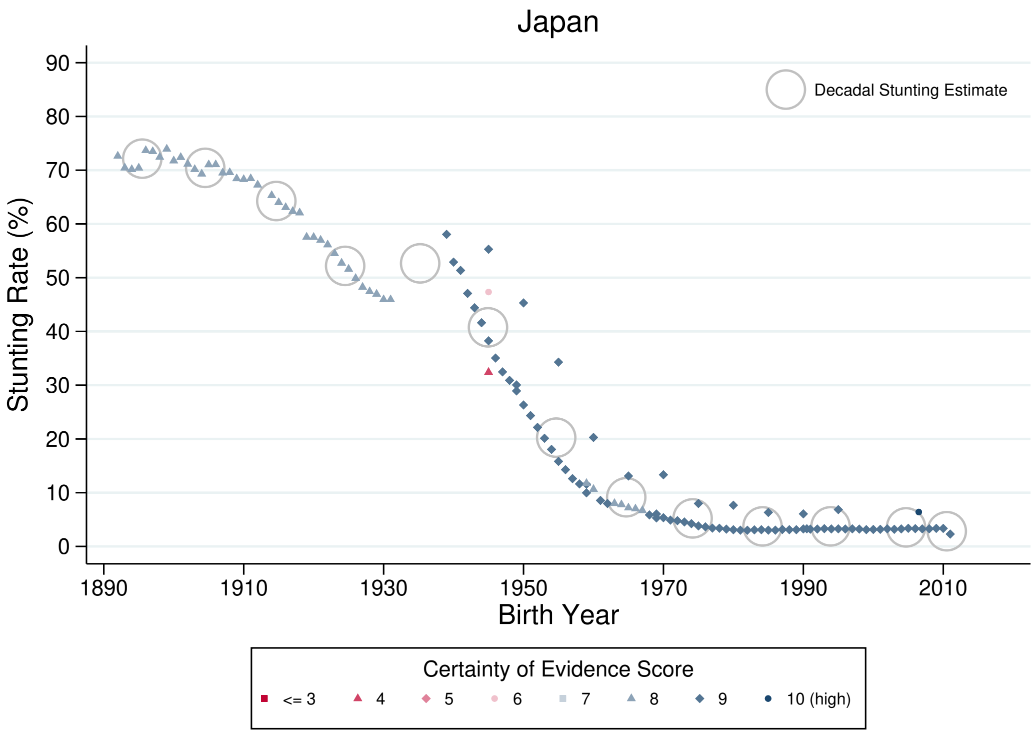

Figure O.57: Japan Study-Level Stunting Rates and Certainty of Evidence Scores

Sources: Worldwide Historical Stunting Dataset.

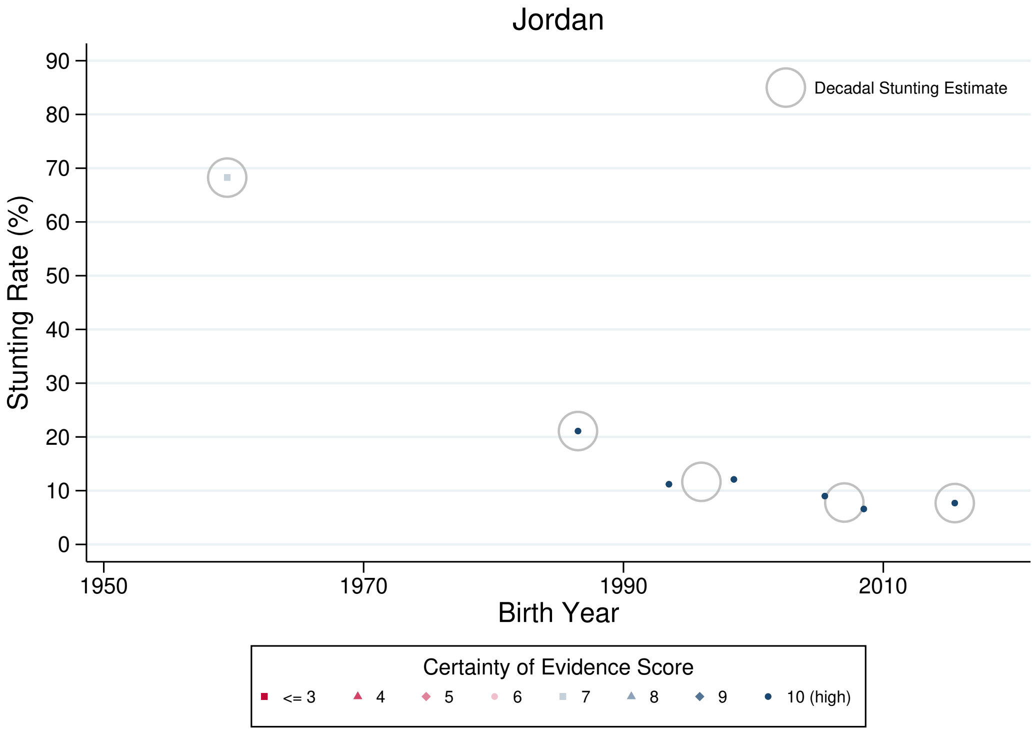

Figure O.58: Jordan Study-Level Stunting Rates and Certainty of Evidence Scores

Sources: Worldwide Historical Stunting Dataset.

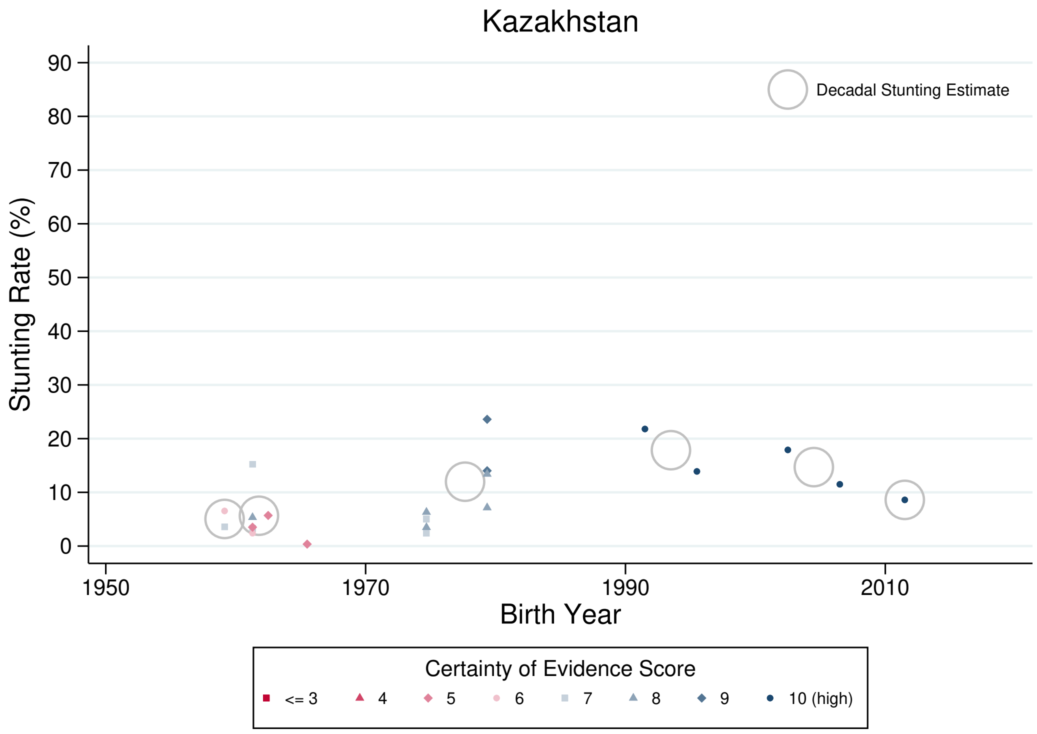

Figure O.59: Kazakhstan Study-Level Stunting Rates and Certainty of Evidence Scores

Sources: Worldwide Historical Stunting Dataset.

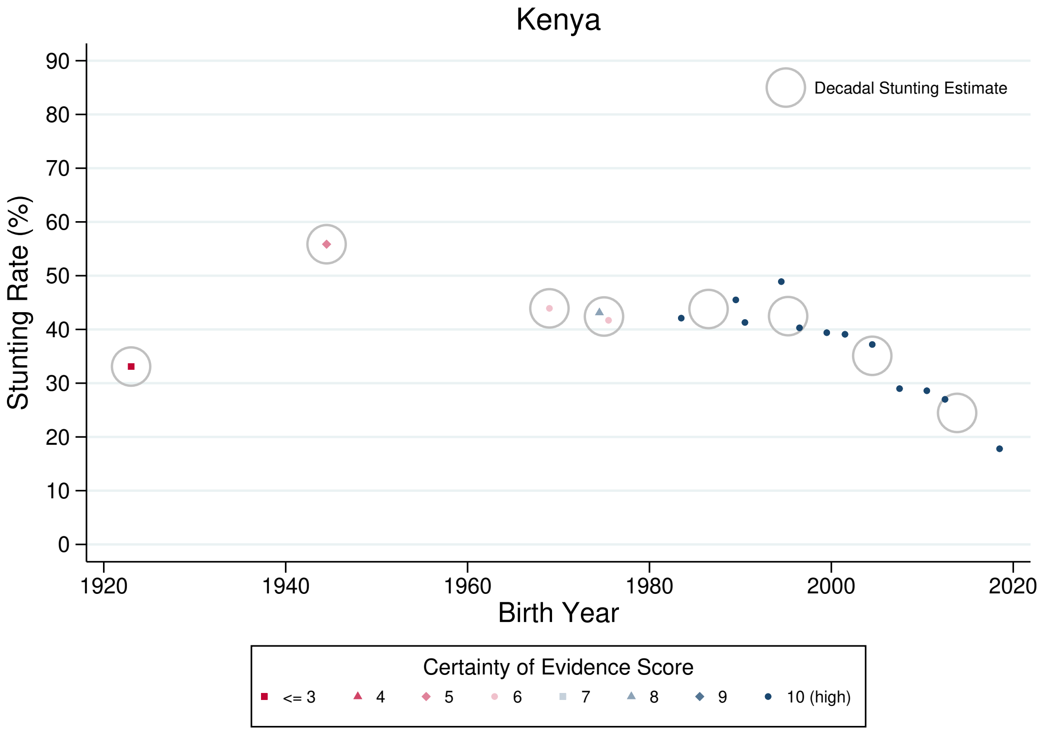

Figure O.60: Kenya Study-Level Stunting Rates and Certainty of Evidence Scores

Sources: Worldwide Historical Stunting Dataset.

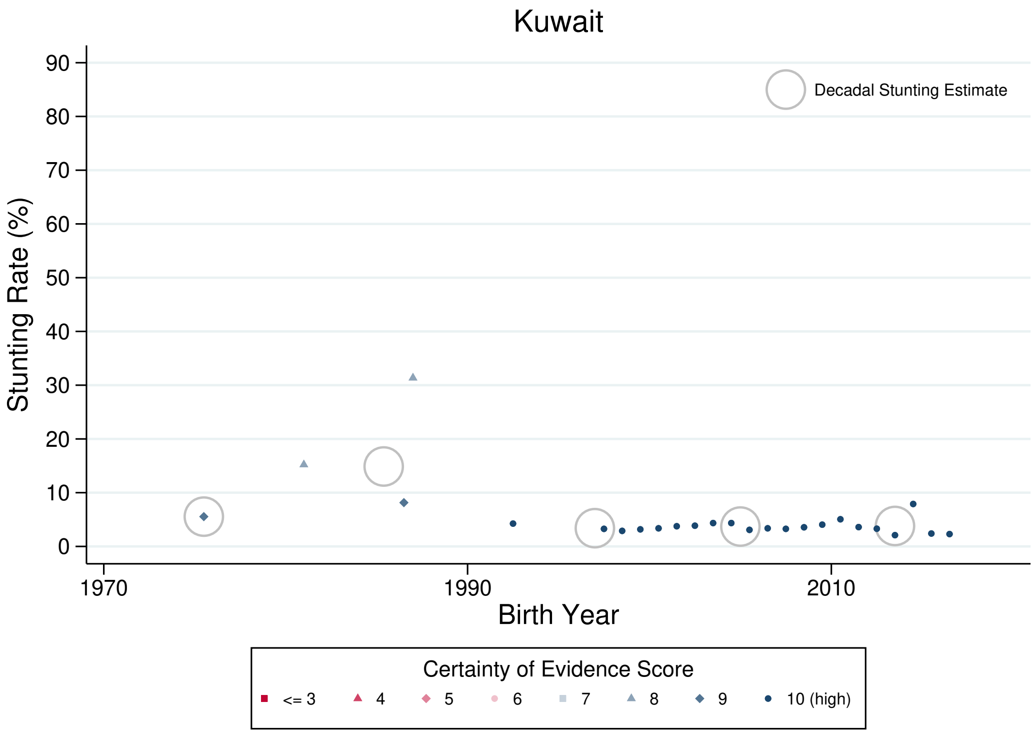

Figure O.61: Kuwait Study-Level Stunting Rates and Certainty of Evidence Scores

Sources: Worldwide Historical Stunting Dataset.

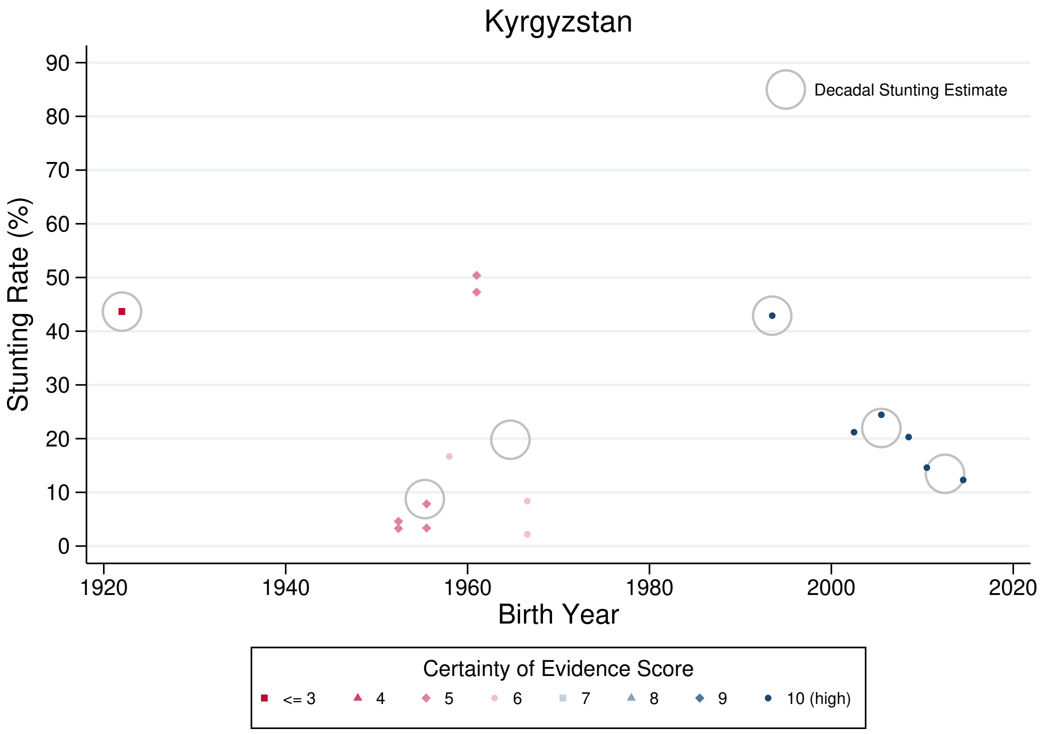

Figure O.62: Kyrgyzstan Study-Level Stunting Rates and Certainty of Evidence Scores

Sources: Worldwide Historical Stunting Dataset.

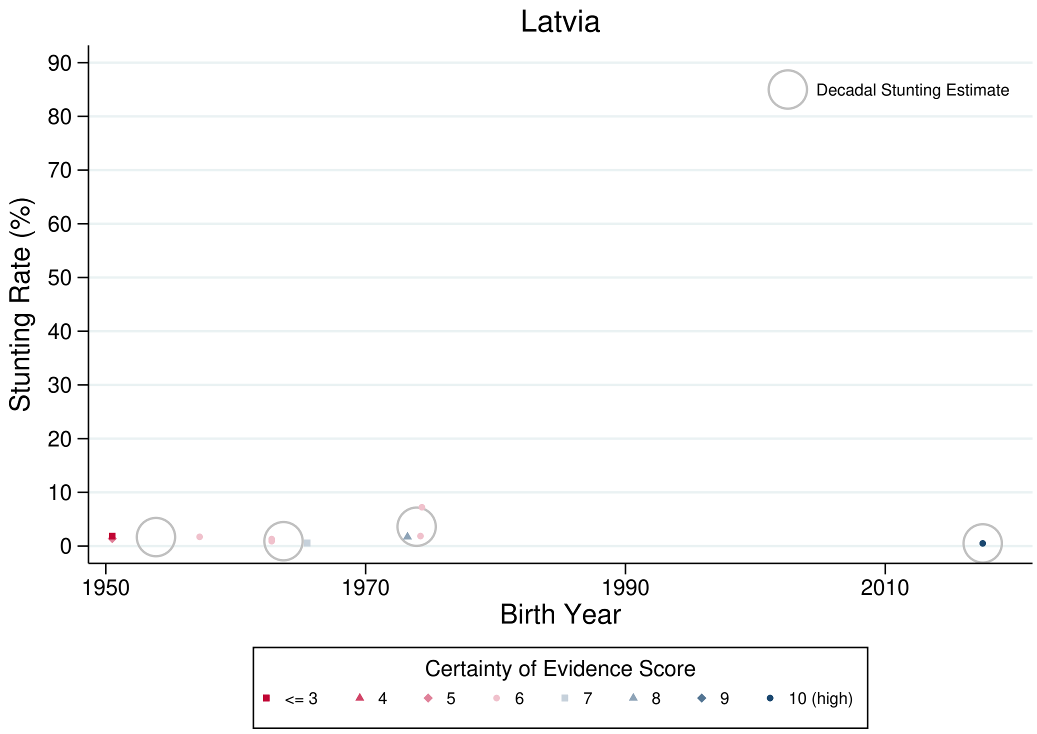

Figure O.63: Latvia Study-Level Stunting Rates and Certainty of Evidence Scores

Sources: Worldwide Historical Stunting Dataset.

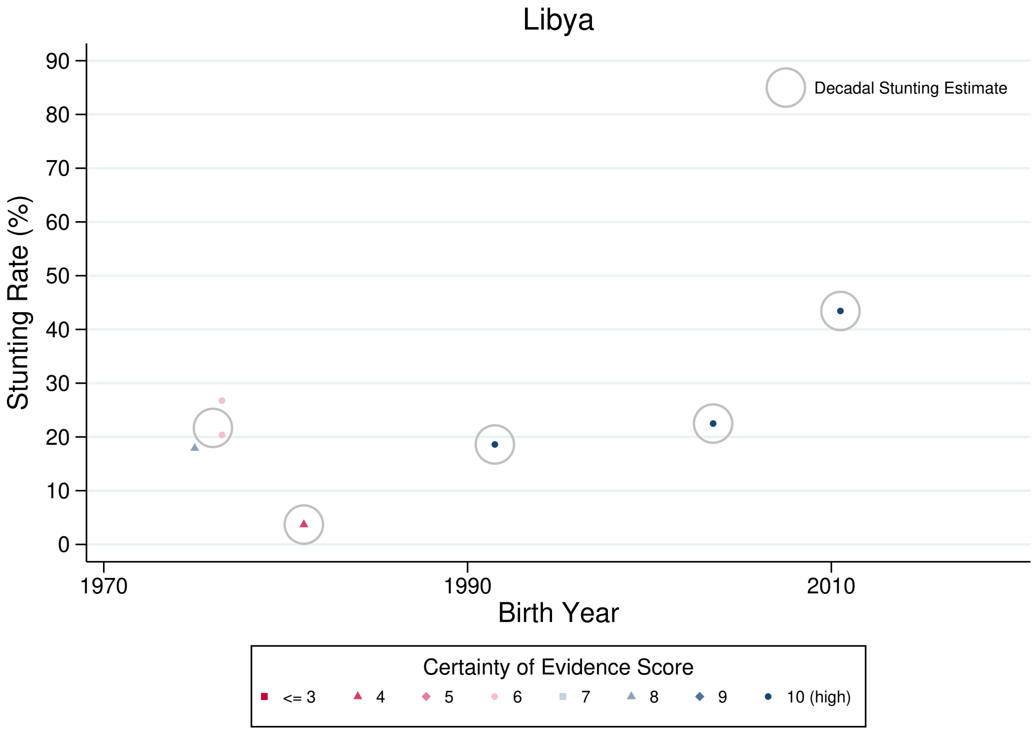

Figure O.64: Libya Study-Level Stunting Rates and Certainty of Evidence Scores

Sources: Worldwide Historical Stunting Dataset.

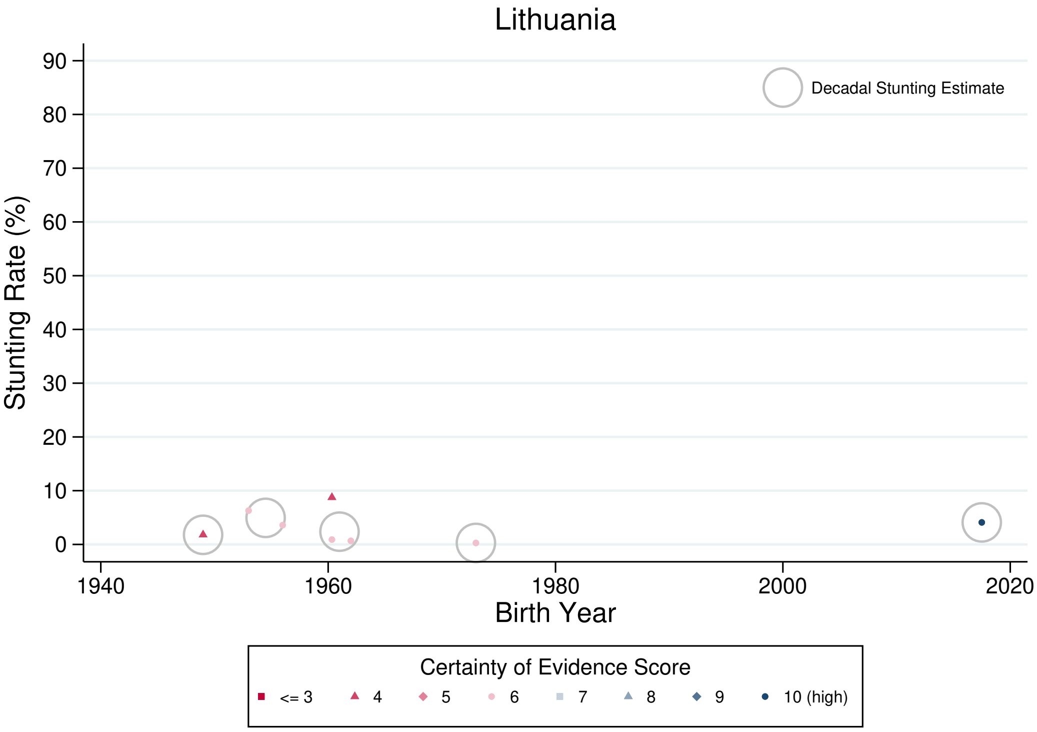

Figure O.65: Lithuania Study-Level Stunting Rates and Certainty of Evidence Scores

Sources: Worldwide Historical Stunting Dataset.

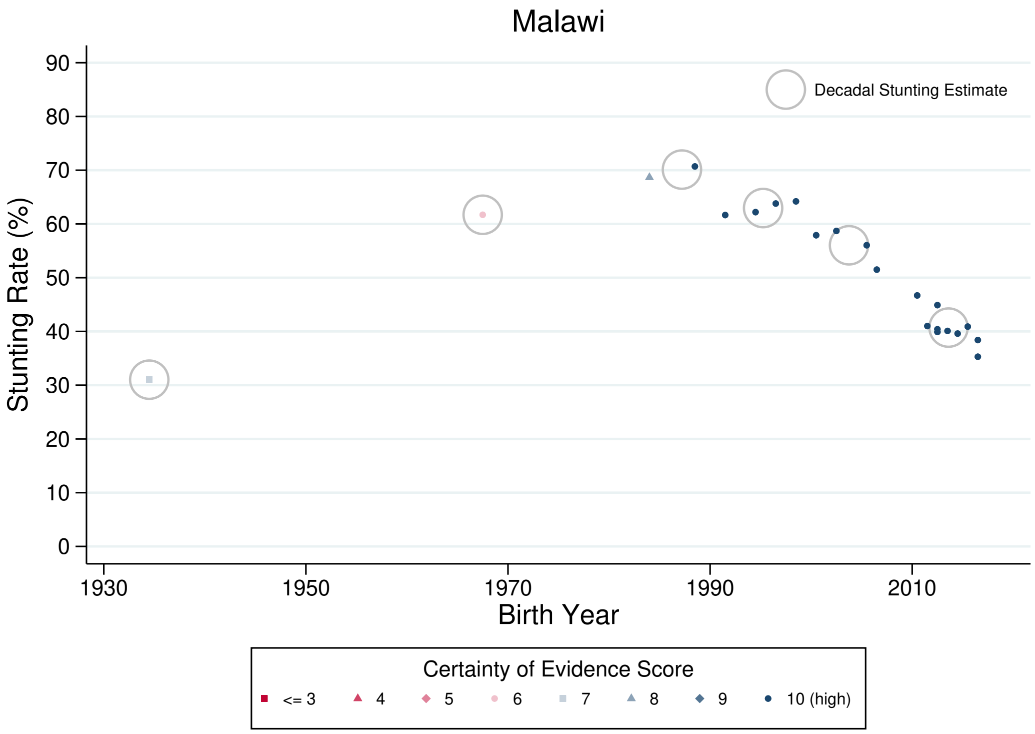

Figure O.66: Malawi Study-Level Stunting Rates and Certainty of Evidence Scores

Sources: Worldwide Historical Stunting Dataset.

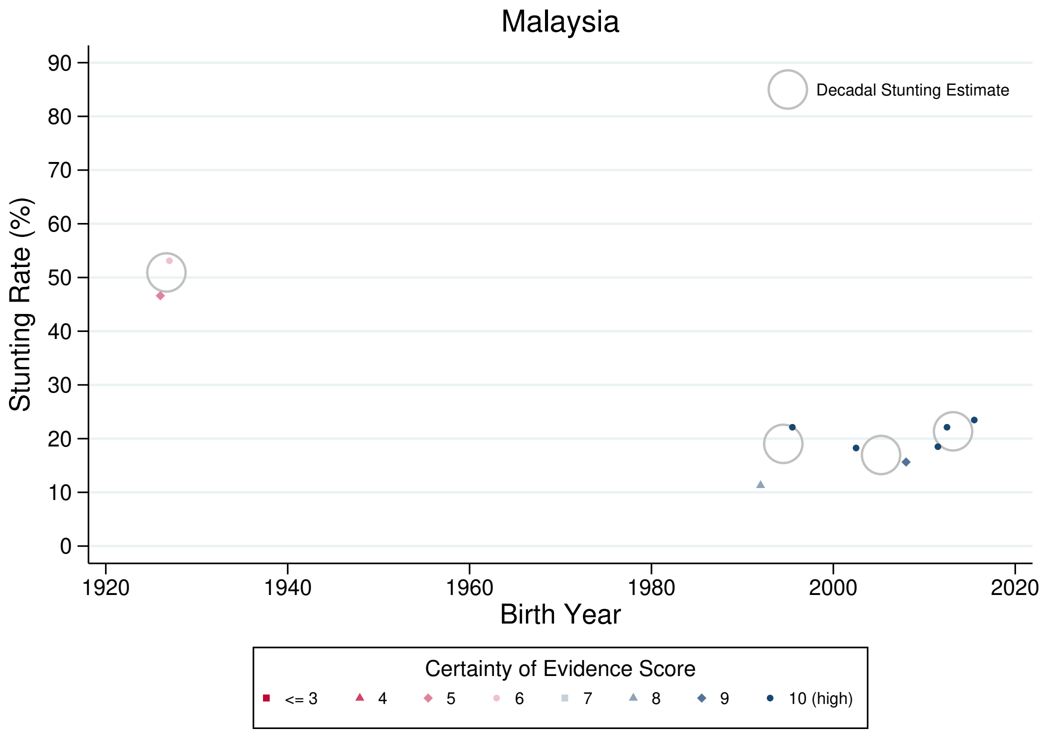

Figure O.67: Malaysia Study-Level Stunting Rates and Certainty of Evidence Scores

Sources: Worldwide Historical Stunting Dataset.

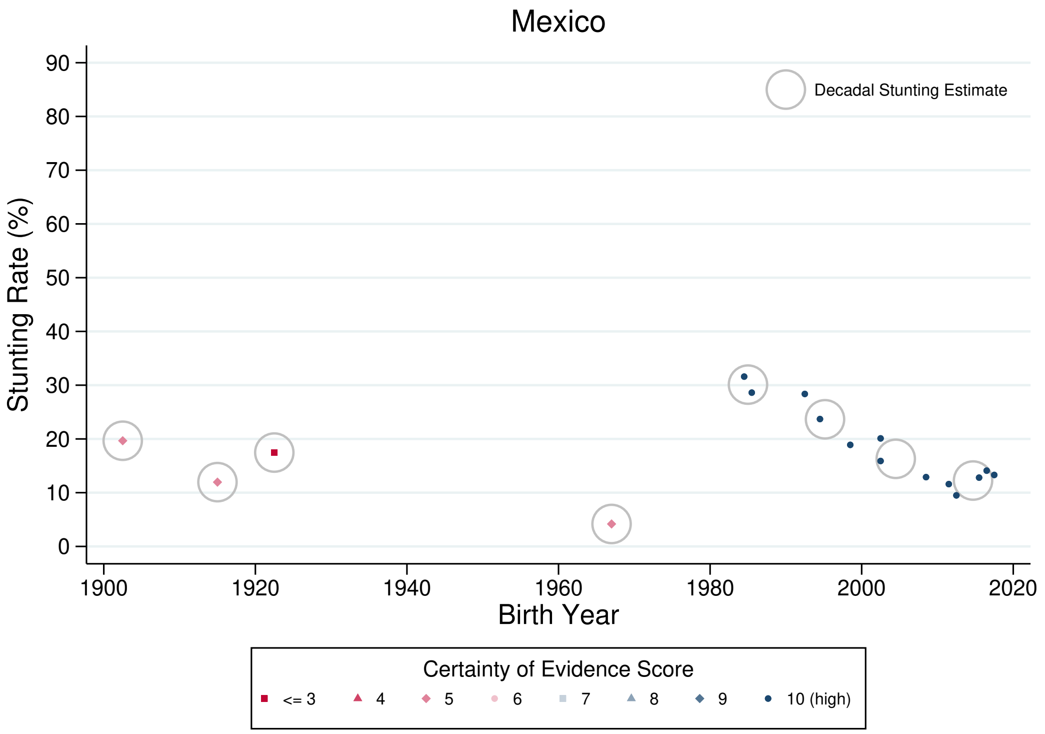

Figure O.68: Mexico Study-Level Stunting Rates and Certainty of Evidence Scores

Sources: Worldwide Historical Stunting Dataset.

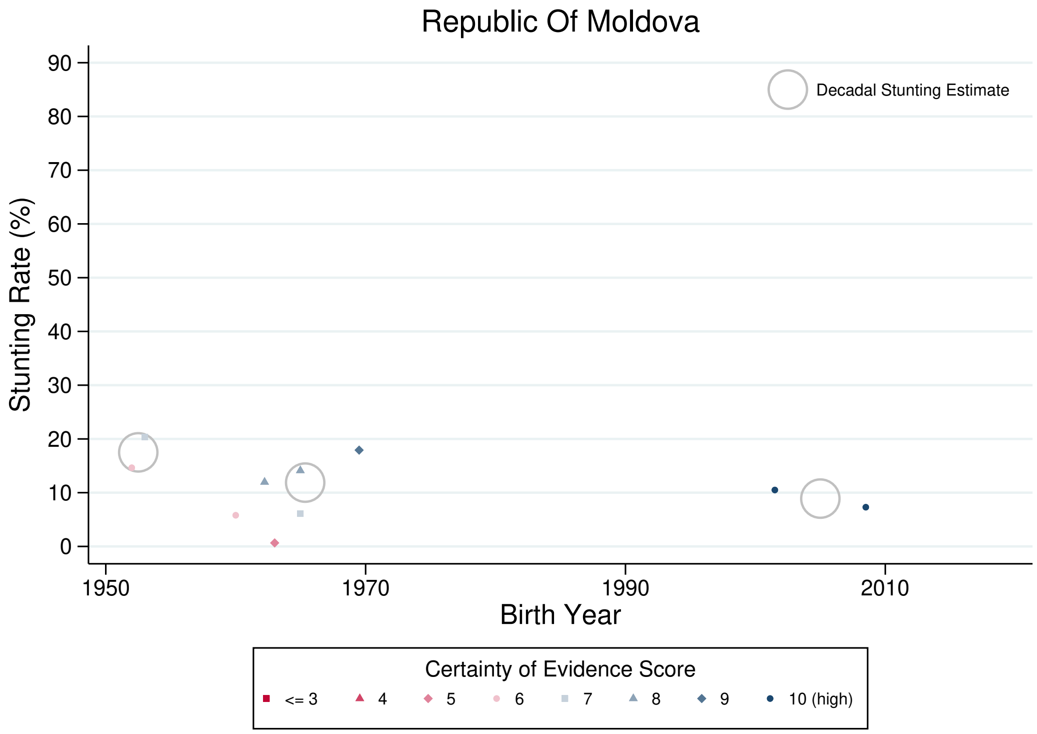

Figure O.69: Moldova Study-Level Stunting Rates and Certainty of Evidence Scores

Sources: Worldwide Historical Stunting Dataset.

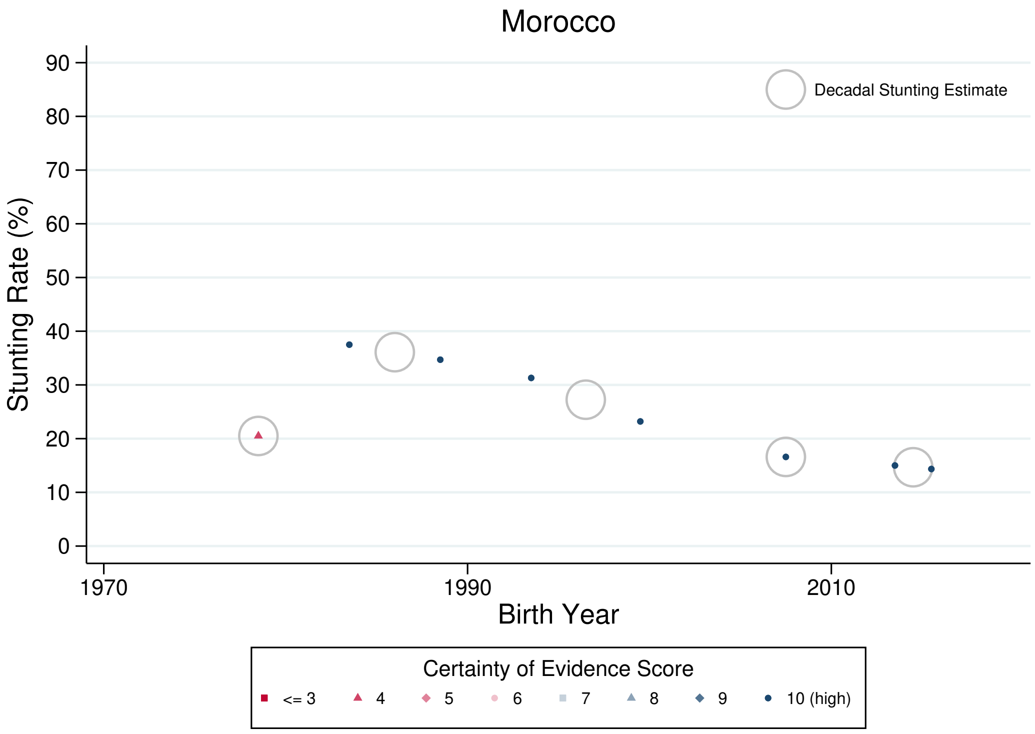

Figure O.70: Morocco Study-Level Stunting Rates and Certainty of Evidence Scores

Sources: Worldwide Historical Stunting Dataset.

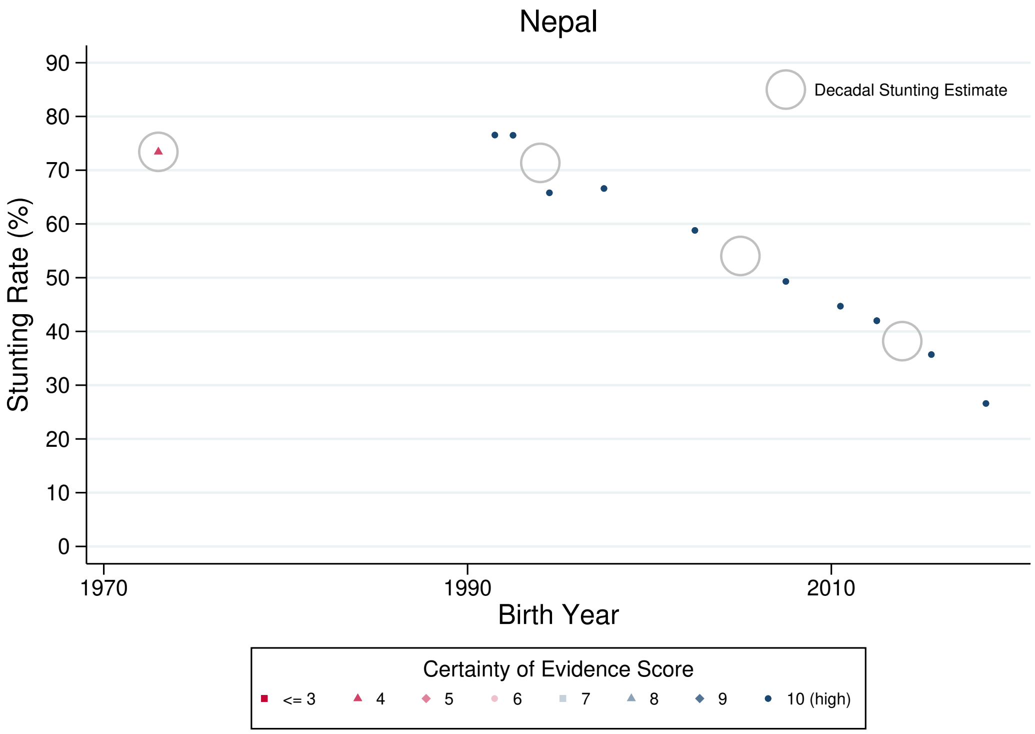

Figure O.71: Nepal Study-Level Stunting Rates and Certainty of Evidence Scores

Sources: Worldwide Historical Stunting Dataset.

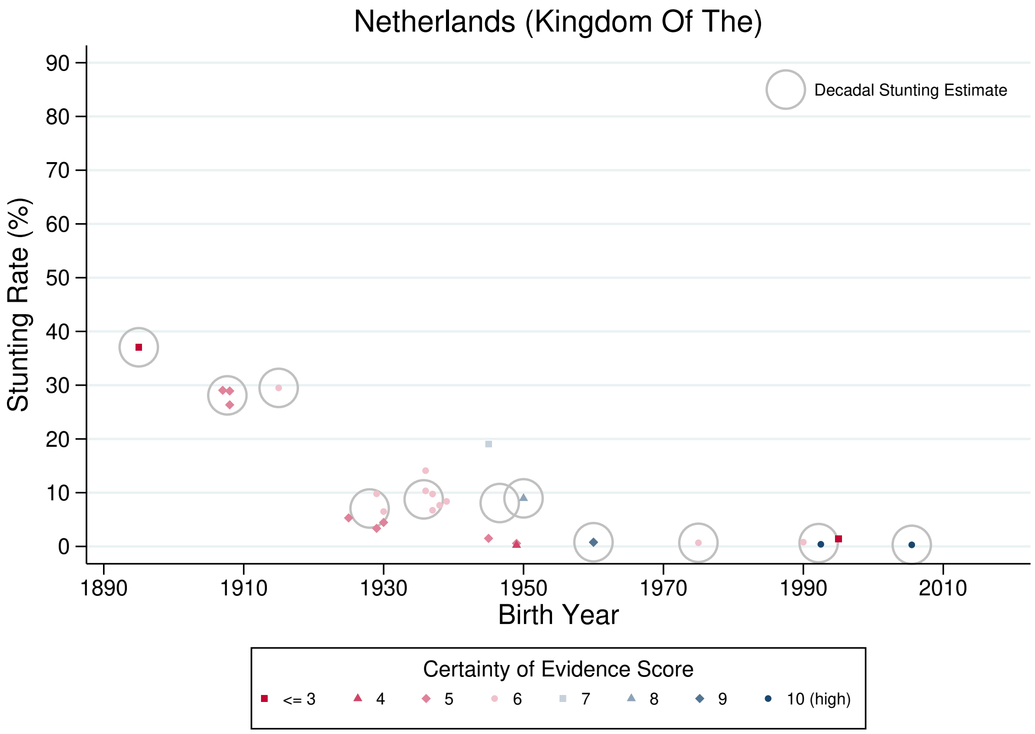

Figure O.72: Netherlands Study-Level Stunting Rates and Certainty of Evidence Scores

Sources: Worldwide Historical Stunting Dataset.

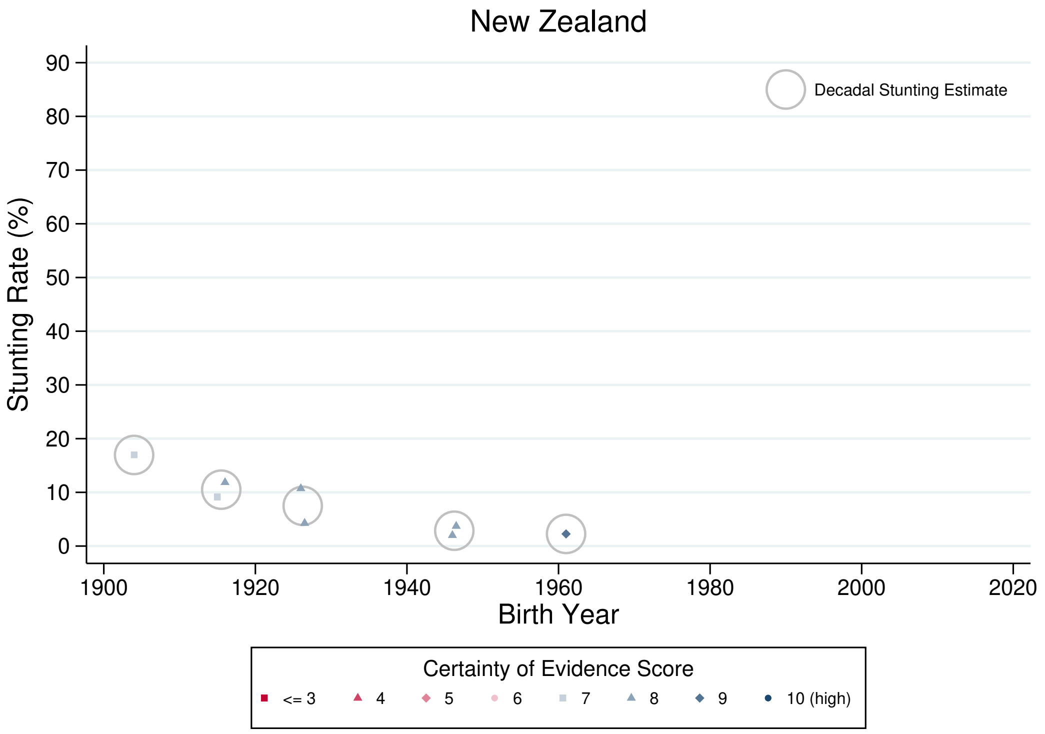

Figure O.73: New Zealand Study-Level Stunting Rates and Certainty of Evidence Scores

Sources: Worldwide Historical Stunting Dataset.

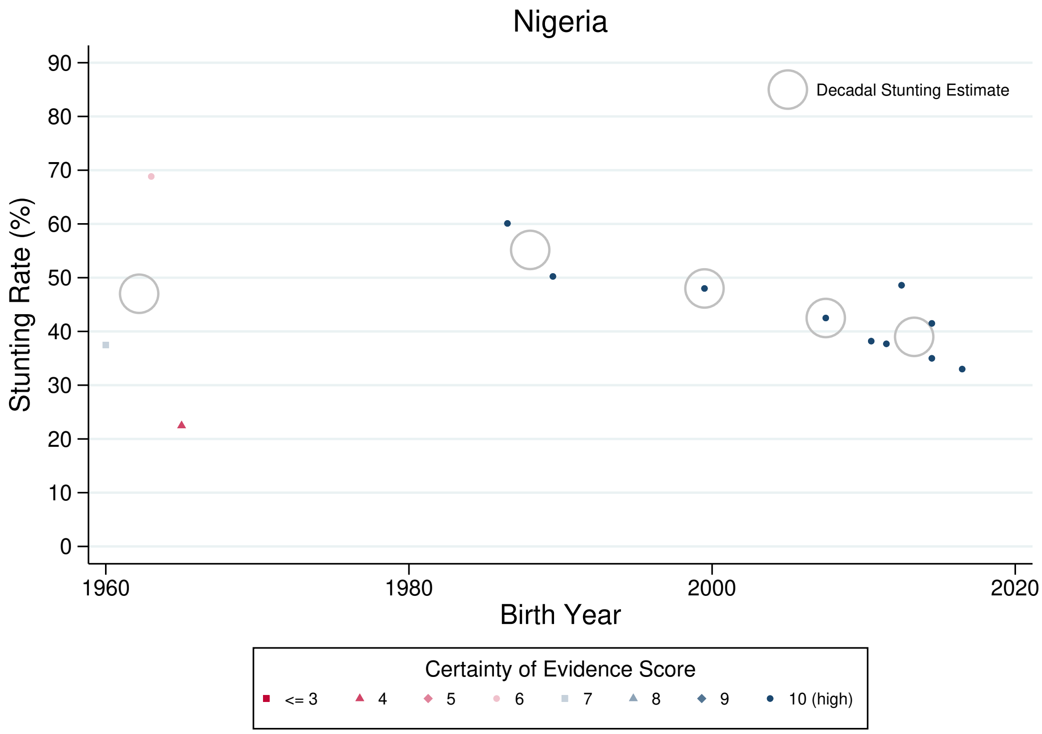

Figure O.74: Nigeria Study-Level Stunting Rates and Certainty of Evidence Scores

Sources: Worldwide Historical Stunting Dataset.

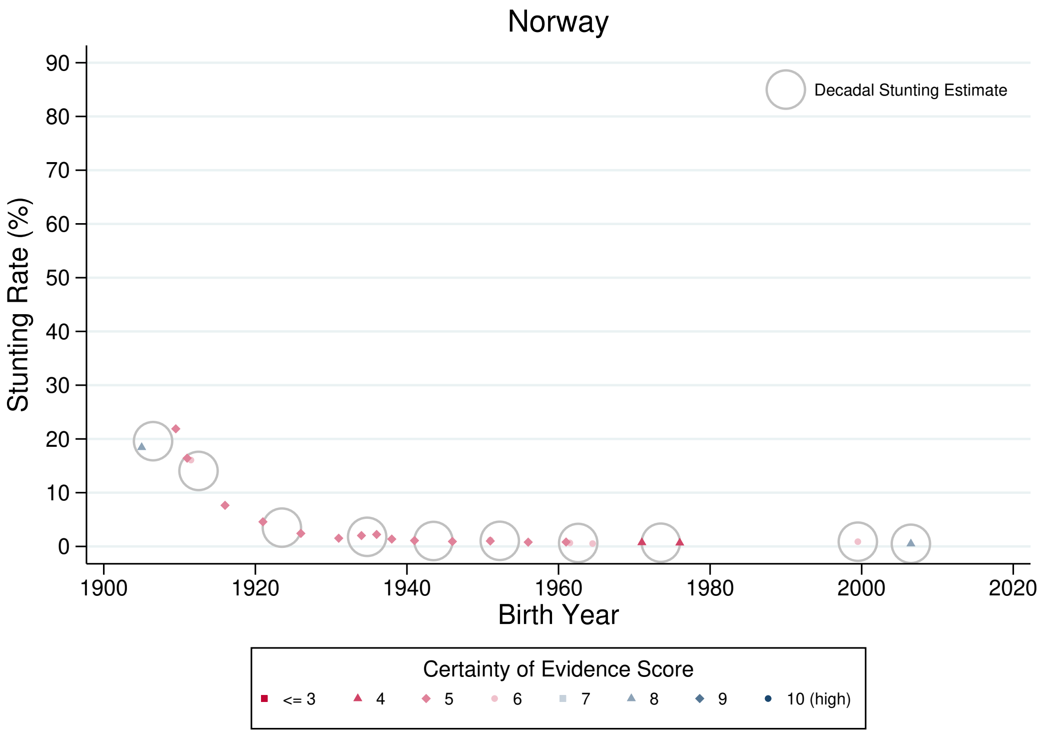

Figure O.75: Norway Study-Level Stunting Rates and Certainty of Evidence Scores

Sources: Worldwide Historical Stunting Dataset.

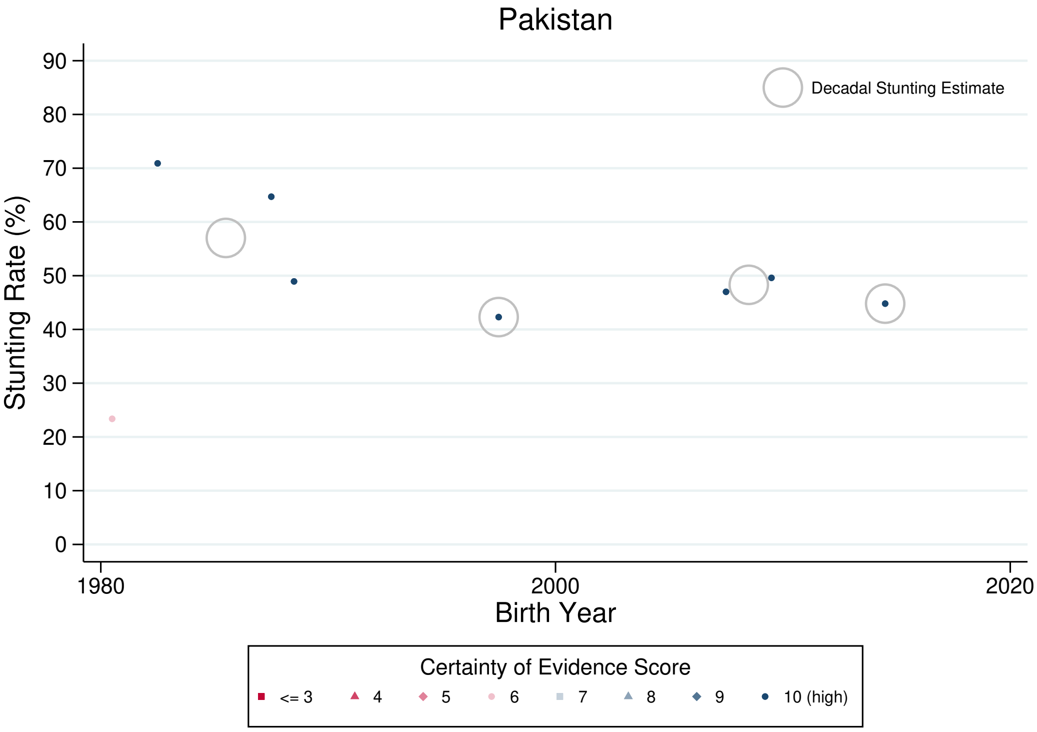

Figure O.76: Pakistan Study-Level Stunting Rates and Certainty of Evidence Scores

Sources: Worldwide Historical Stunting Dataset.

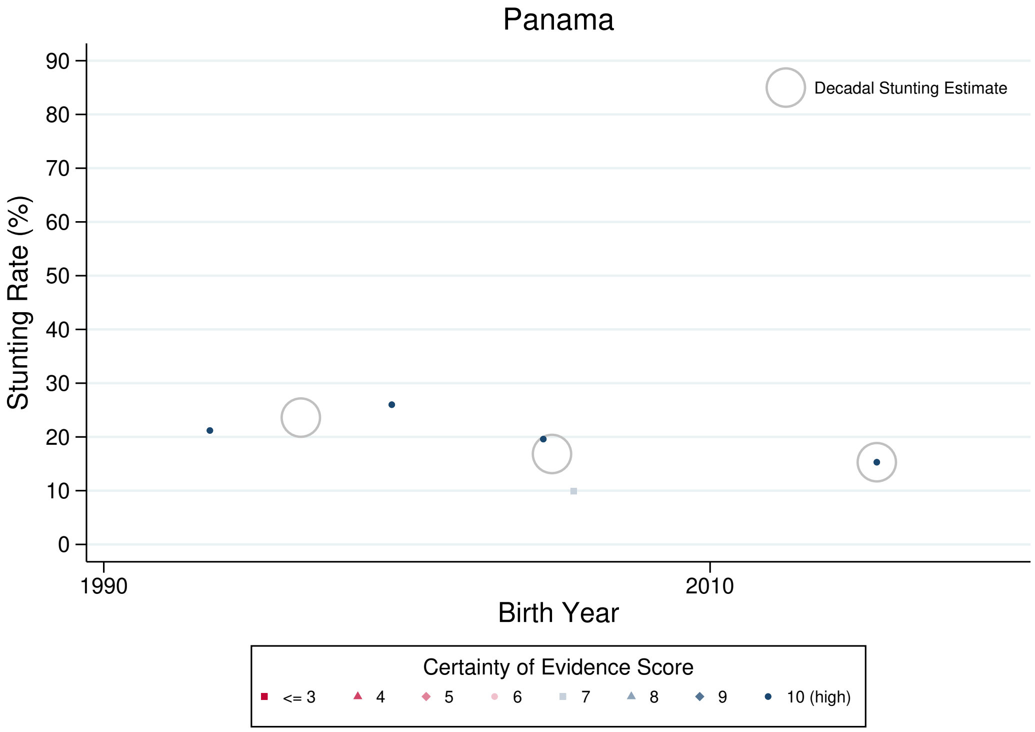

Figure O.77: Panama Study-Level Stunting Rates and Certainty of Evidence Scores

Sources: Worldwide Historical Stunting Dataset.

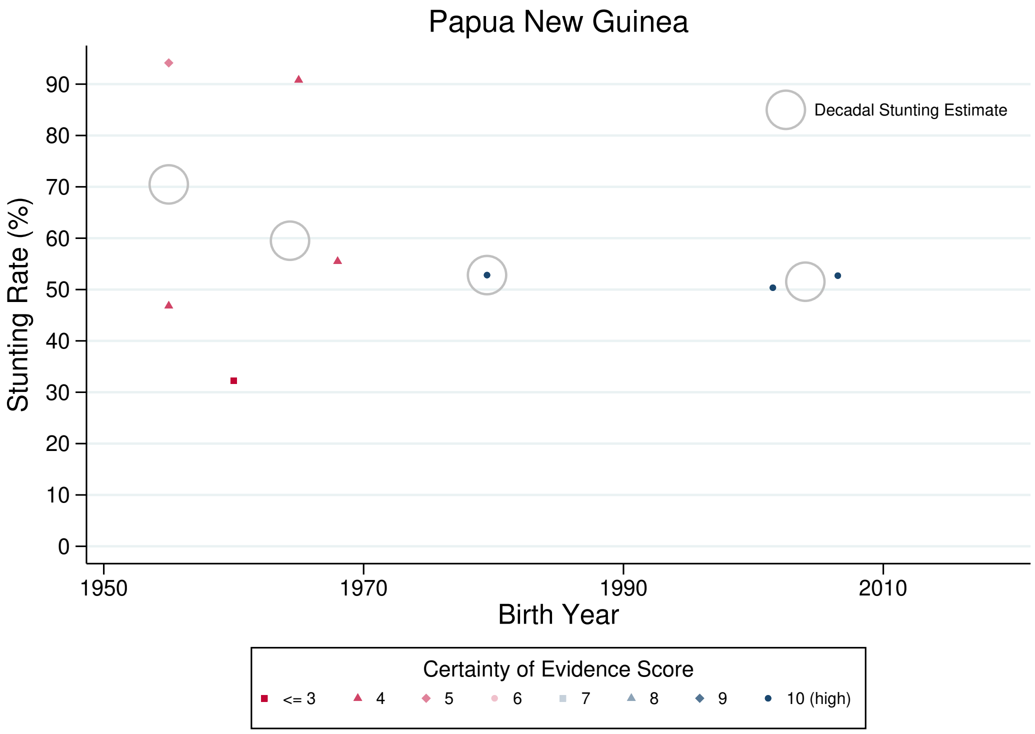

Figure O.78: Papua New Guinea Study-Level Stunting Rates and Certainty of Evidence Scores

Sources: Worldwide Historical Stunting Dataset.

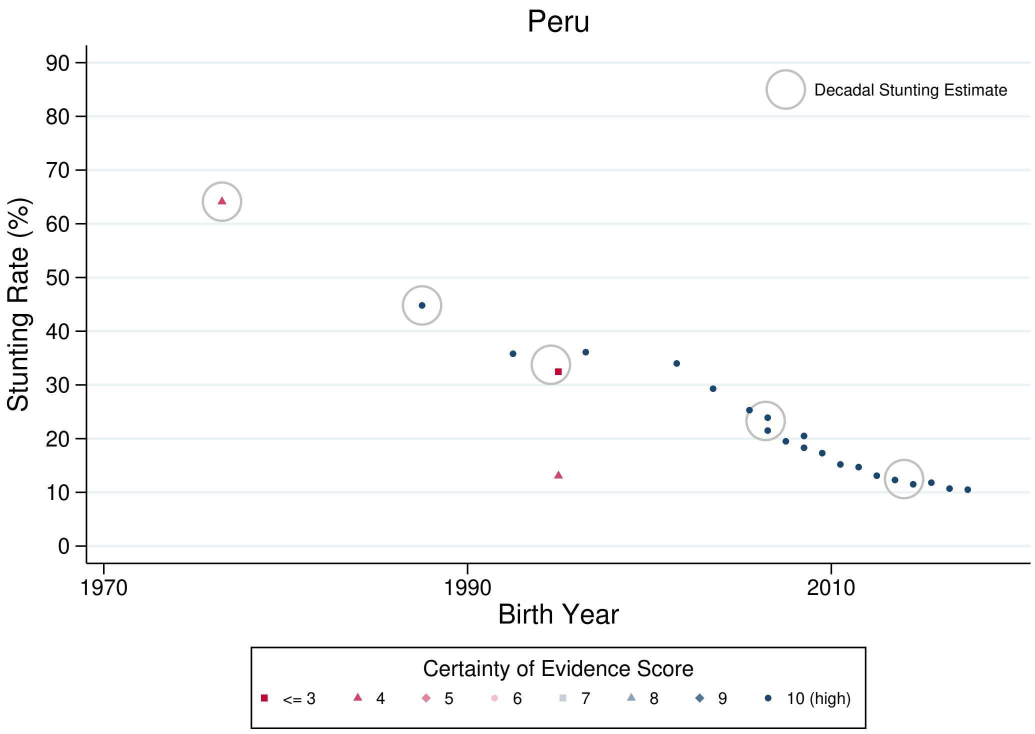

Figure O.79: Peru Study-Level Stunting Rates and Certainty of Evidence Scores

Sources: Worldwide Historical Stunting Dataset.

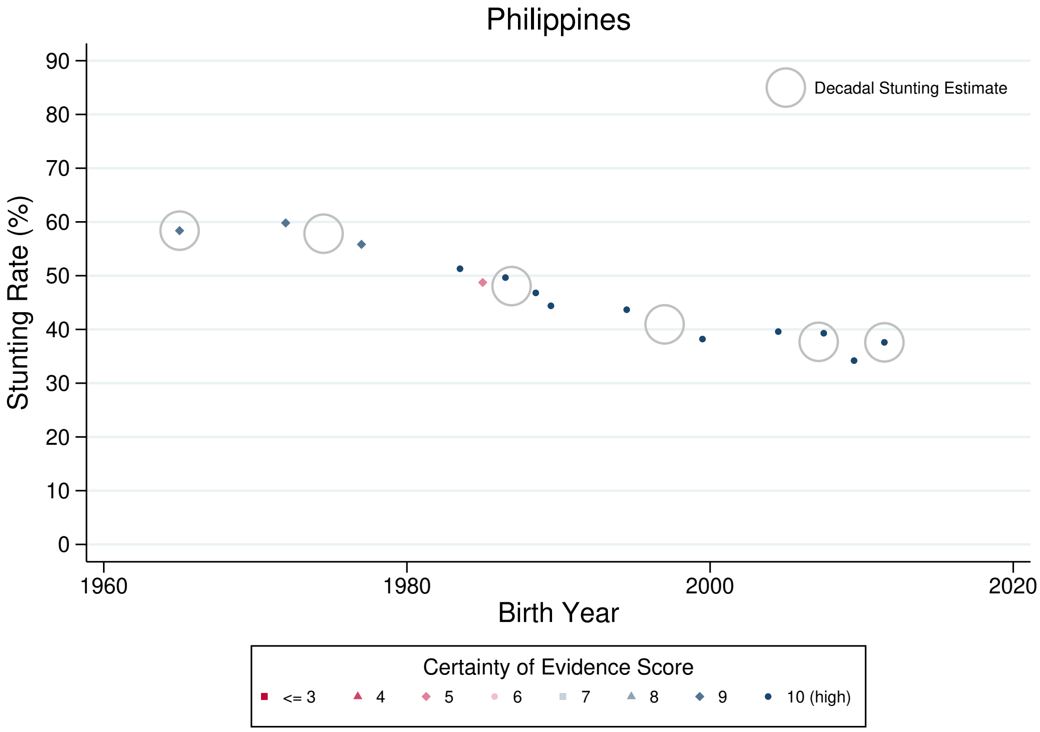

Figure O.80: Philippines Study-Level Stunting Rates and Certainty of Evidence Scores

Sources: Worldwide Historical Stunting Dataset.

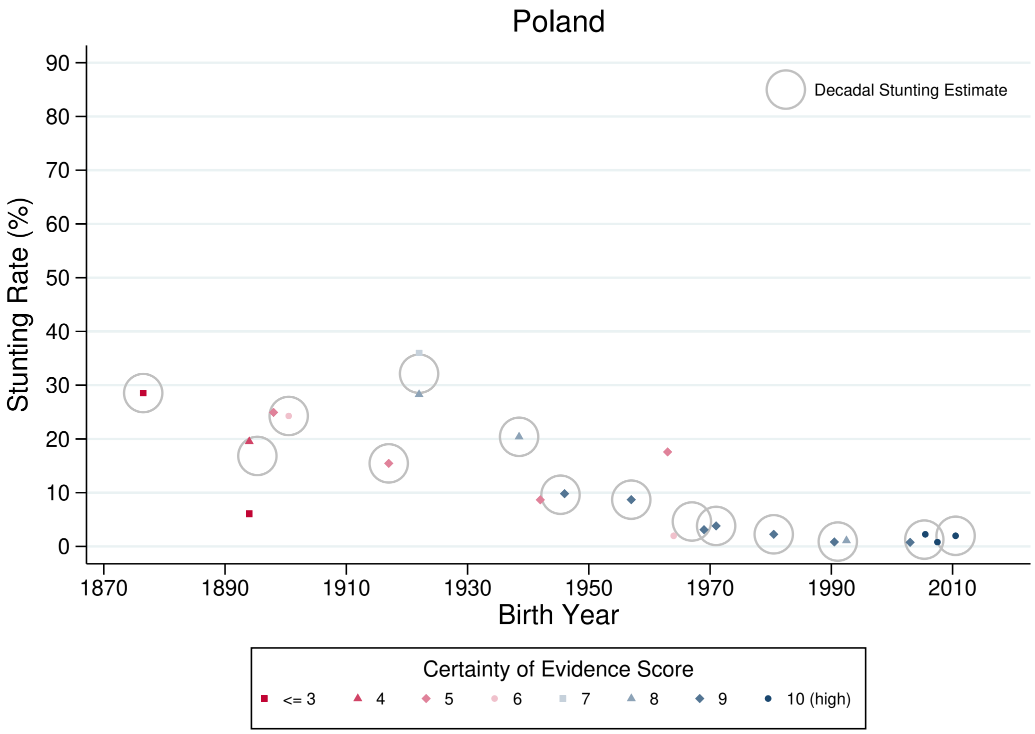

Figure O.81: Poland Study-Level Stunting Rates and Certainty of Evidence Scores

Sources: Worldwide Historical Stunting Dataset.

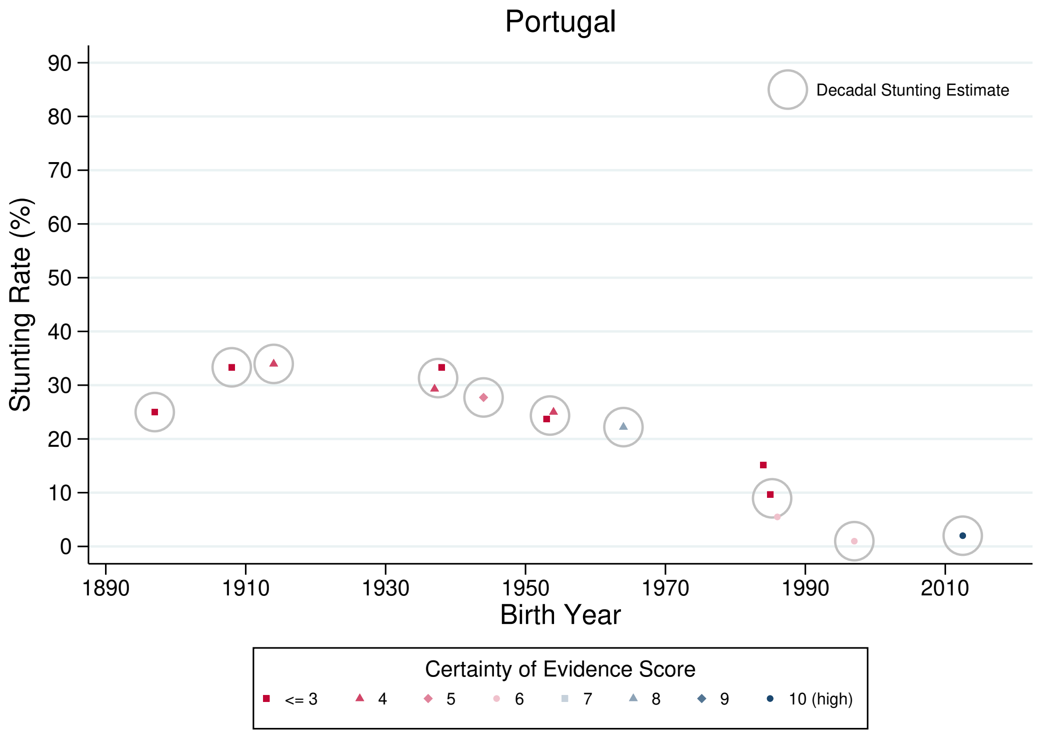

Figure O.82: Portugal Study-Level Stunting Rates and Certainty of Evidence Scores

Sources: Worldwide Historical Stunting Dataset.

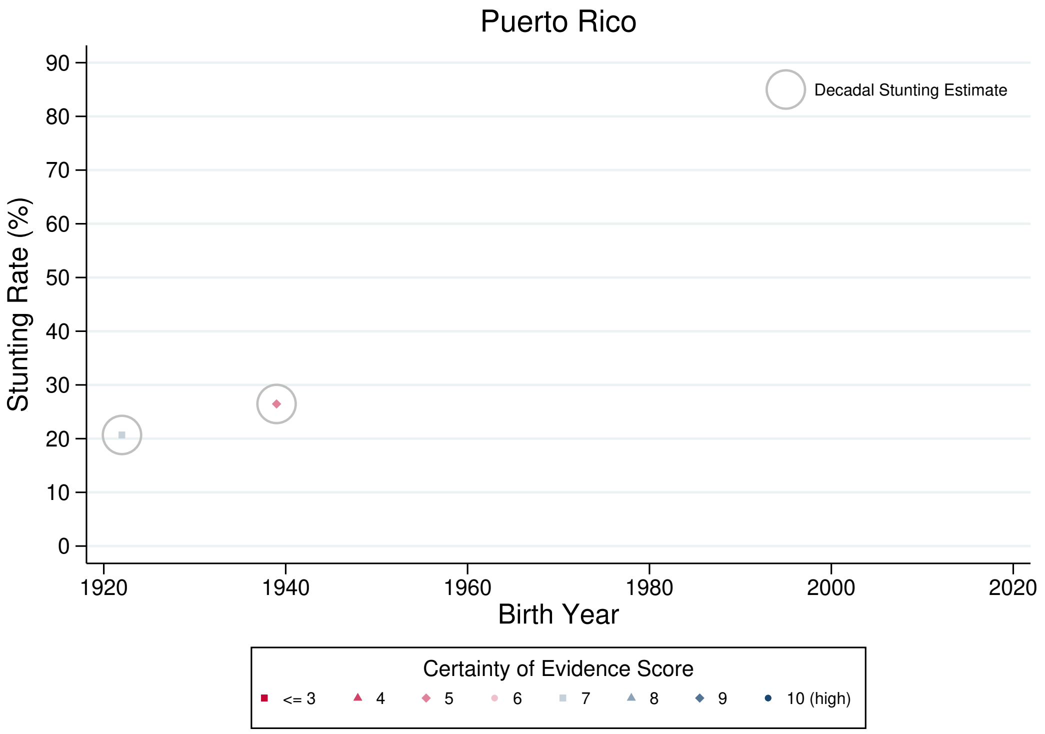

Figure O.83: Puerto Rico Study-Level Stunting Rates and Certainty of Evidence Scores

Sources: Worldwide Historical Stunting Dataset.

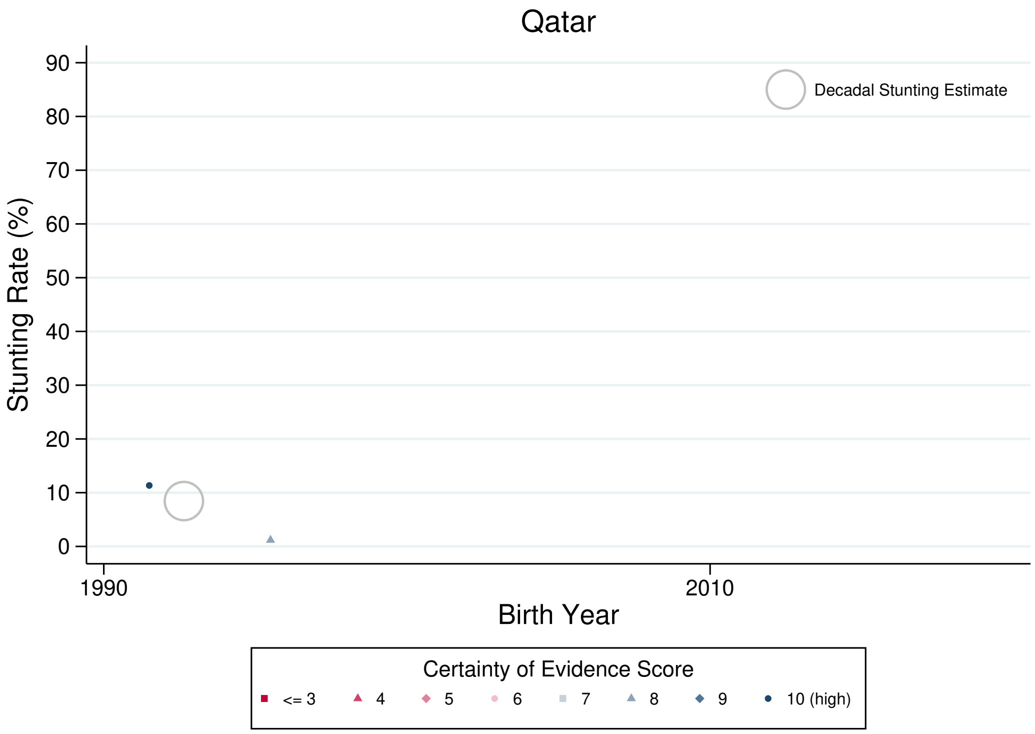

Figure O.84: Qatar Study-Level Stunting Rates and Certainty of Evidence Scores

Sources: Worldwide Historical Stunting Dataset.

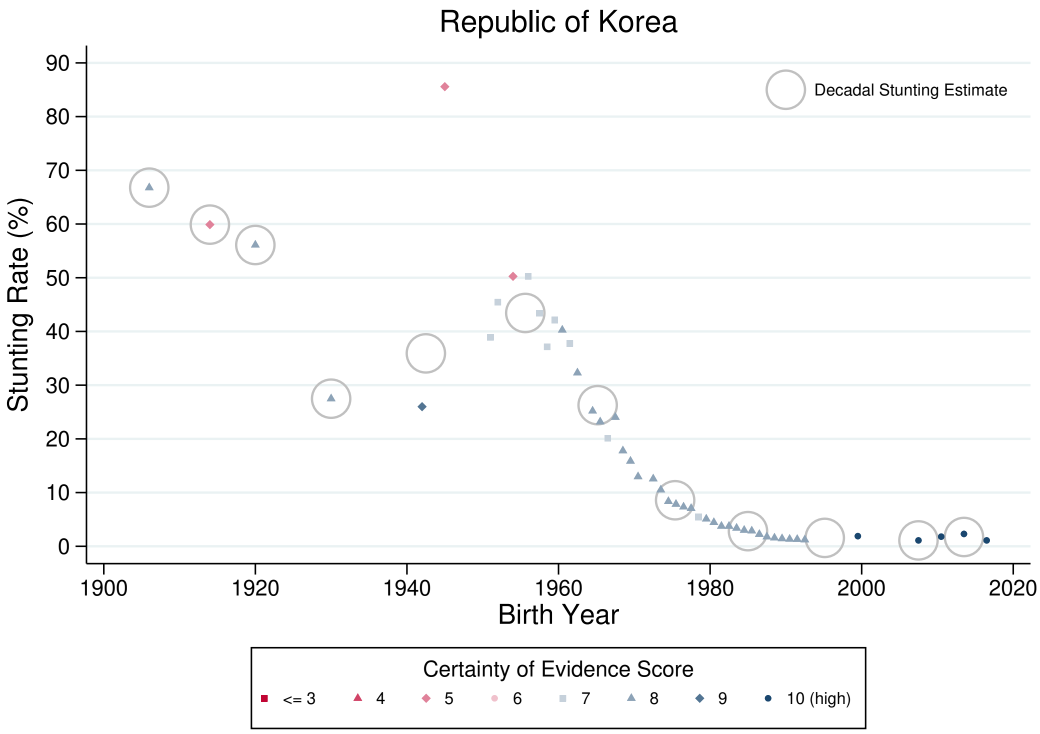

Figure O.85: Republic of Korea Study-Level Stunting Rates and Certainty of Evidence Scores

Sources: Worldwide Historical Stunting Dataset.

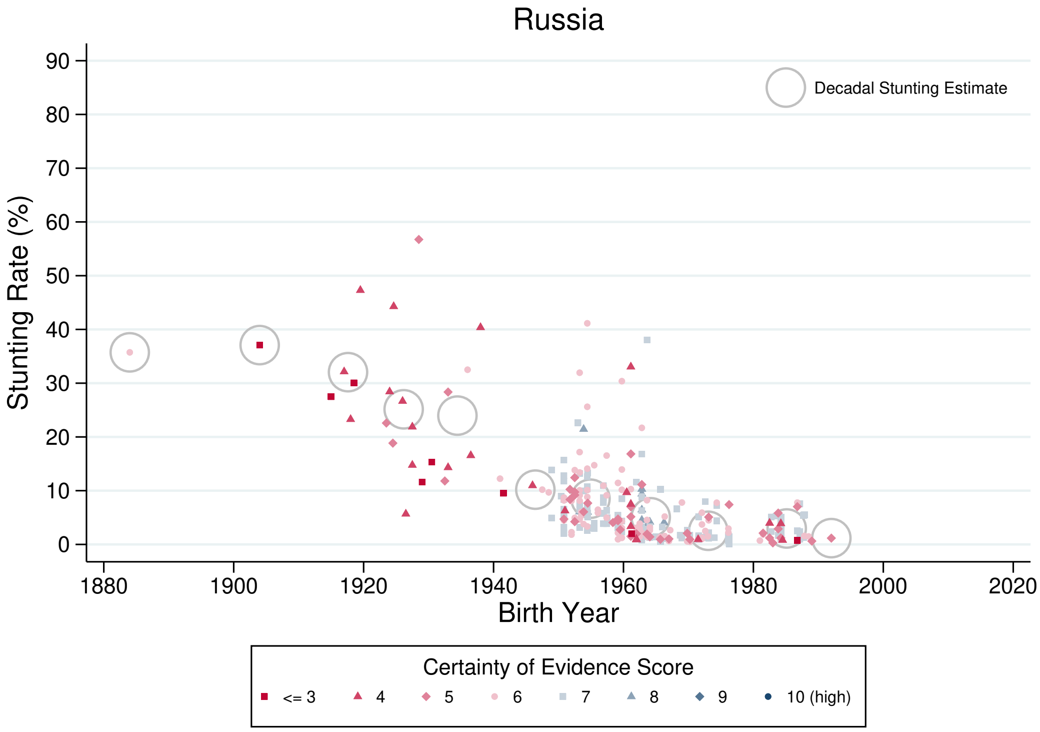

Figure O.86: Russia Study-Level Stunting Rates and Certainty of Evidence Scores

Sources: Worldwide Historical Stunting Dataset.

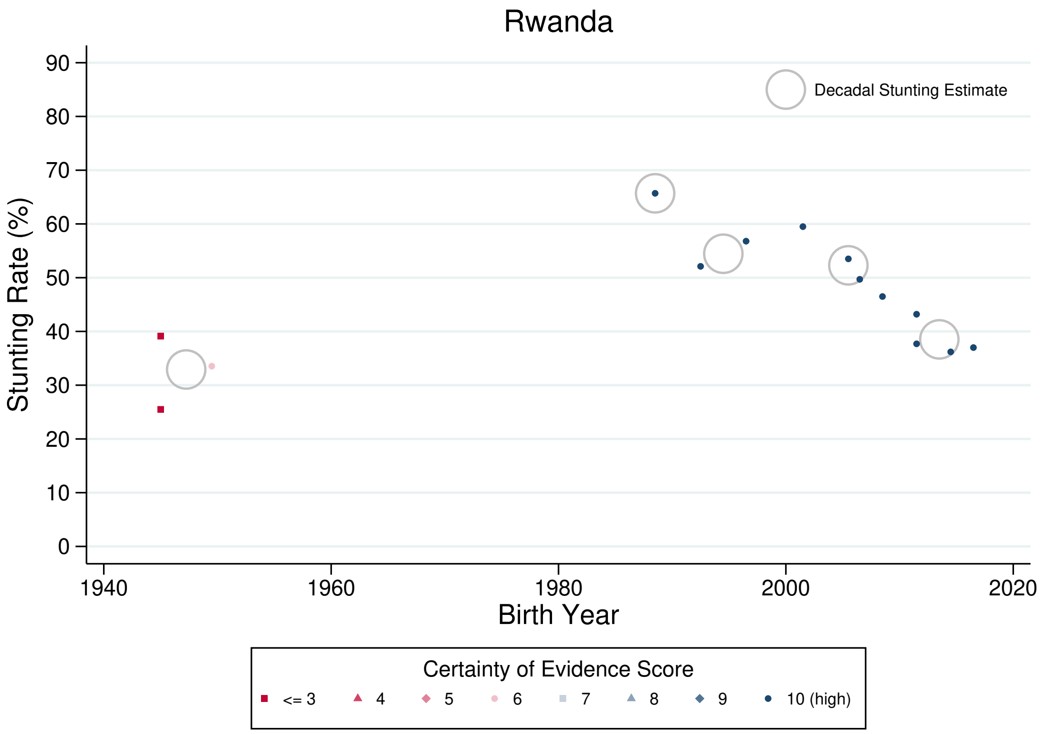

Figure O.87: Rwanda Study-Level Stunting Rates and Certainty of Evidence Scores

Sources: Worldwide Historical Stunting Dataset.

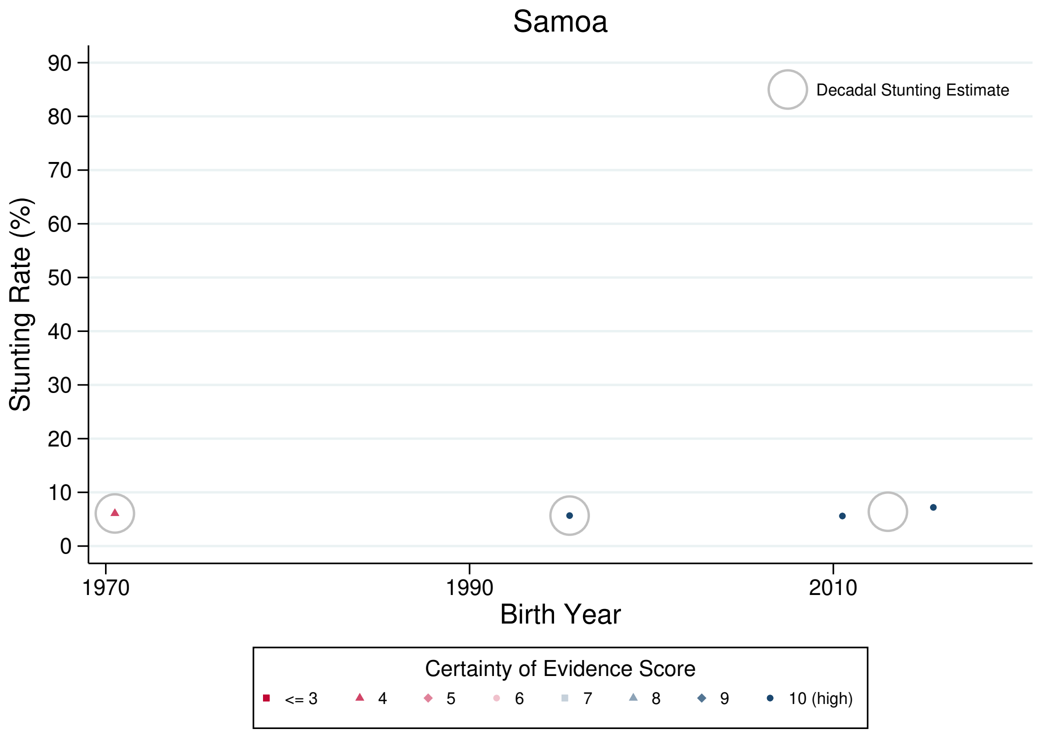

Figure O.88: Samoa Study-Level Stunting Rates and Certainty of Evidence Scores

Sources: Worldwide Historical Stunting Dataset.

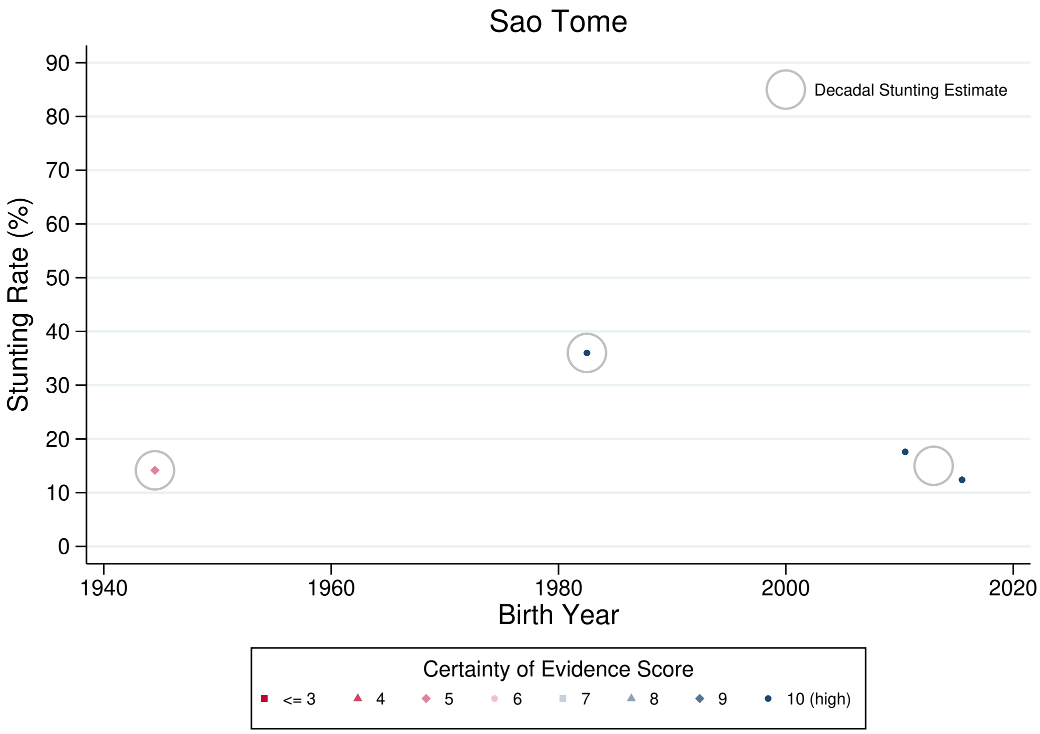

Figure O.89: Sao Tome Study-Level Stunting Rates and Certainty of Evidence Scores

Sources: Worldwide Historical Stunting Dataset.

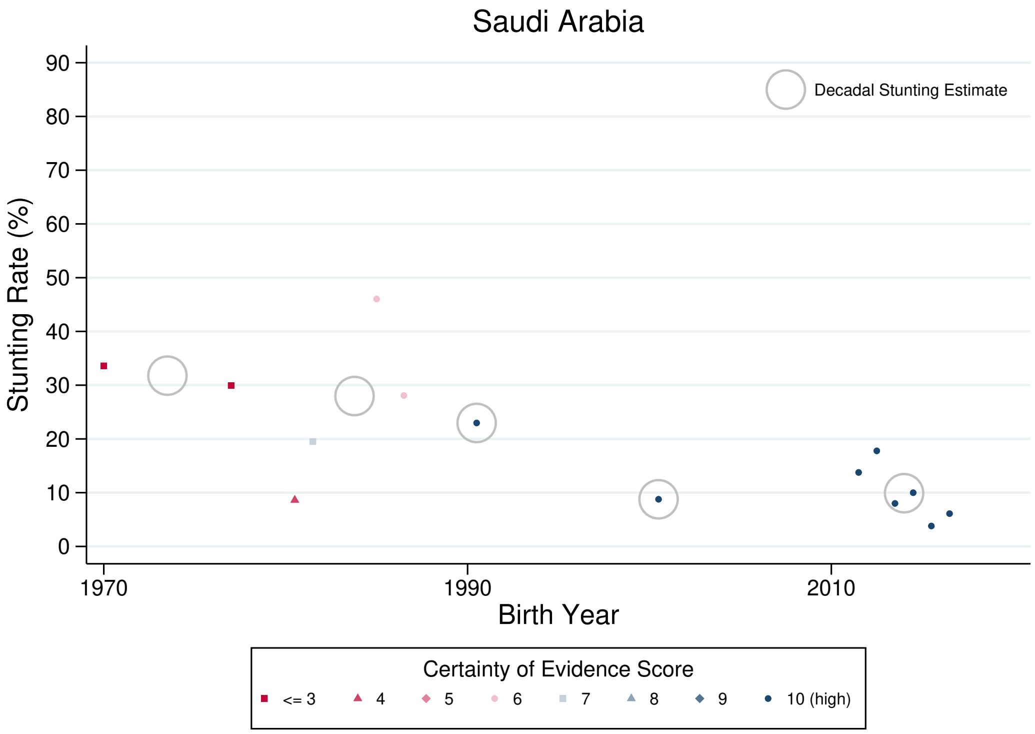

Figure O.90: Saudi Arabia Study-Level Stunting Rates and Certainty of Evidence Scores

Sources: Worldwide Historical Stunting Dataset.

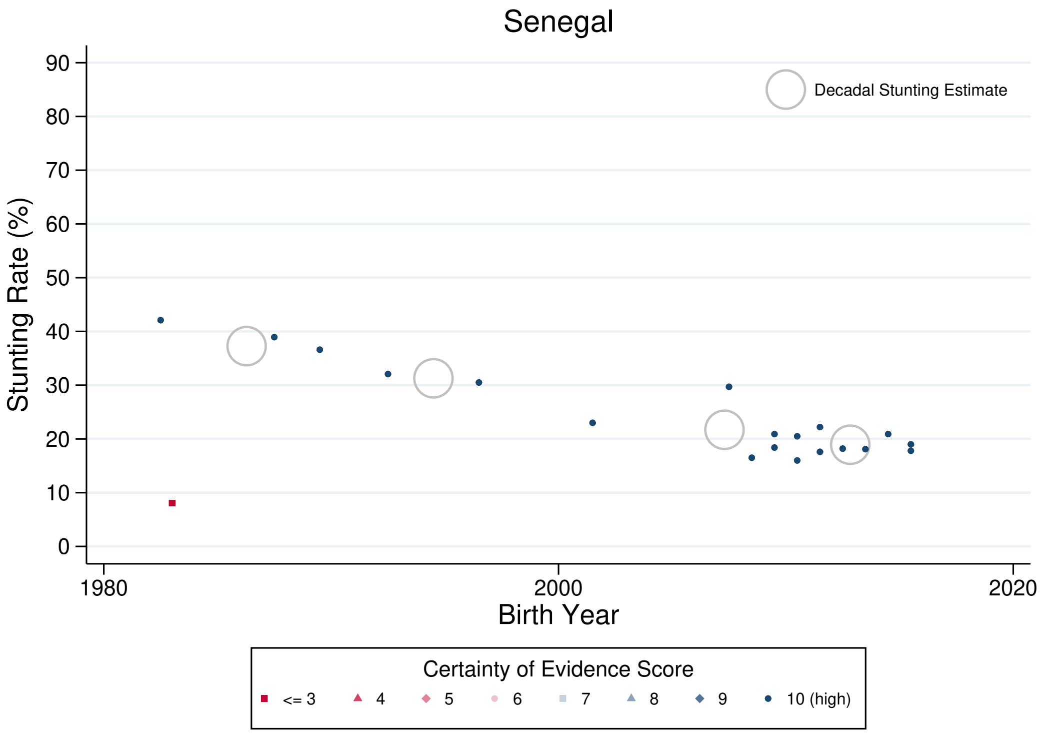

Figure O.91: Senegal Study-Level Stunting Rates and Certainty of Evidence Scores

Sources: Worldwide Historical Stunting Dataset.

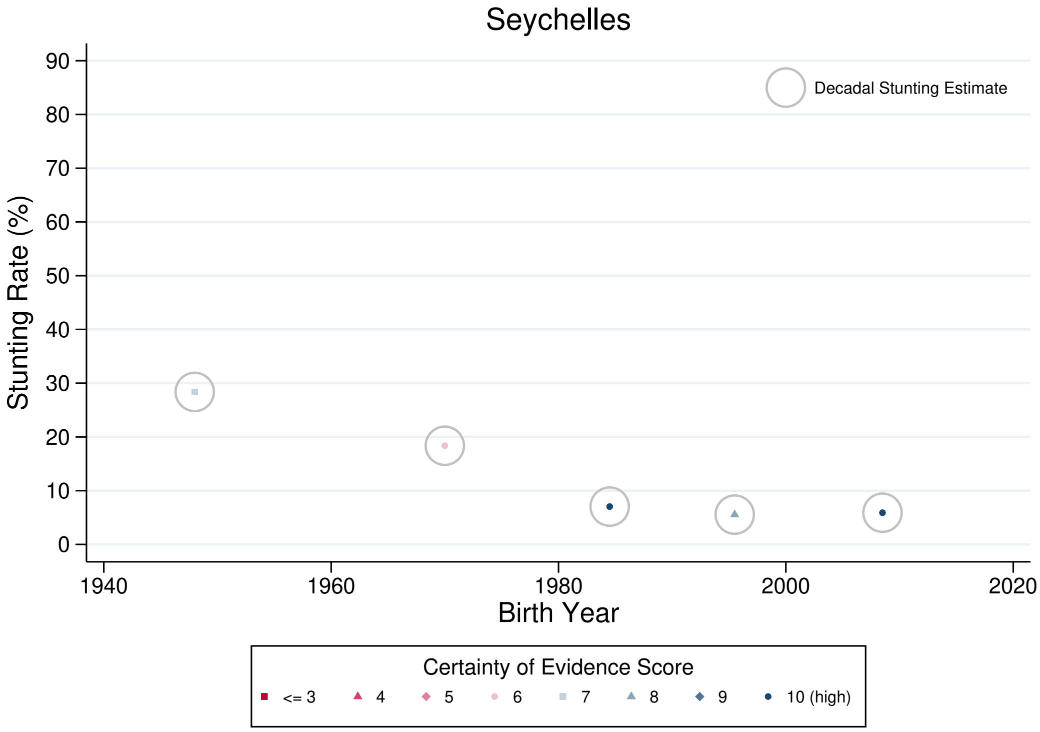

Figure O.92: Seychelles Study-Level Stunting Rates and Certainty of Evidence Scores

Sources: Worldwide Historical Stunting Dataset.

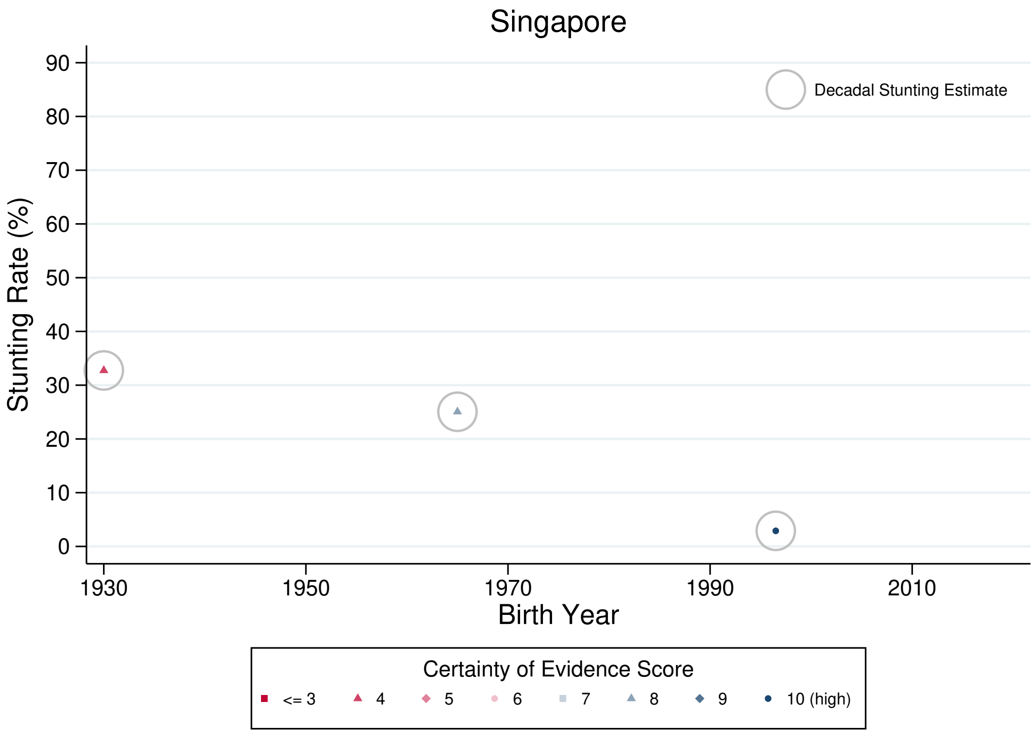

Figure O.93: Singapore Study-Level Stunting Rates and Certainty of Evidence Scores

Sources: Worldwide Historical Stunting Dataset.

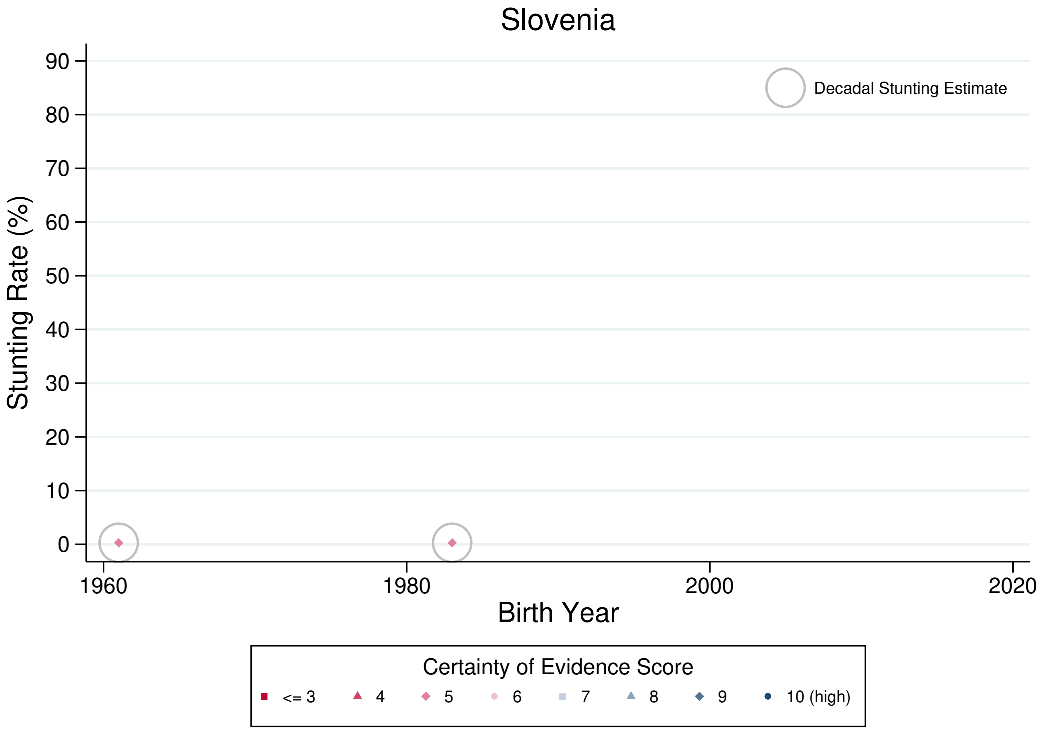

Figure O.94: Slovenia Study-Level Stunting Rates and Certainty of Evidence Scores

Sources: Worldwide Historical Stunting Dataset.

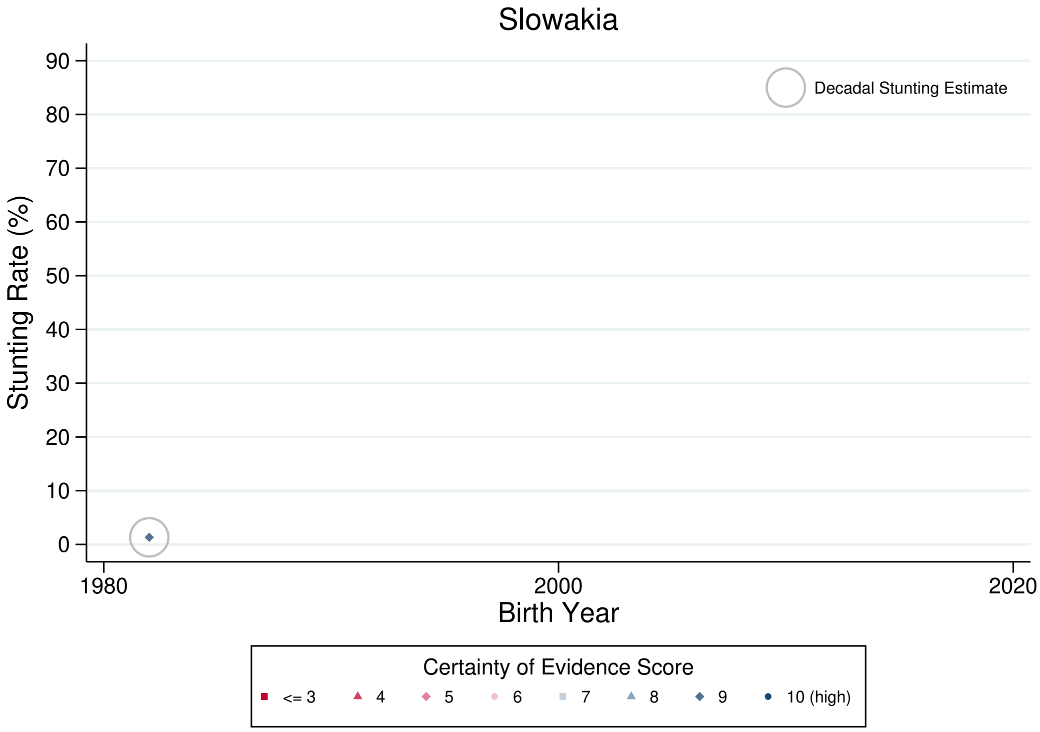

Figure O.95: Slowakia Study-Level Stunting Rates and Certainty of Evidence Scores

Sources: Worldwide Historical Stunting Dataset.

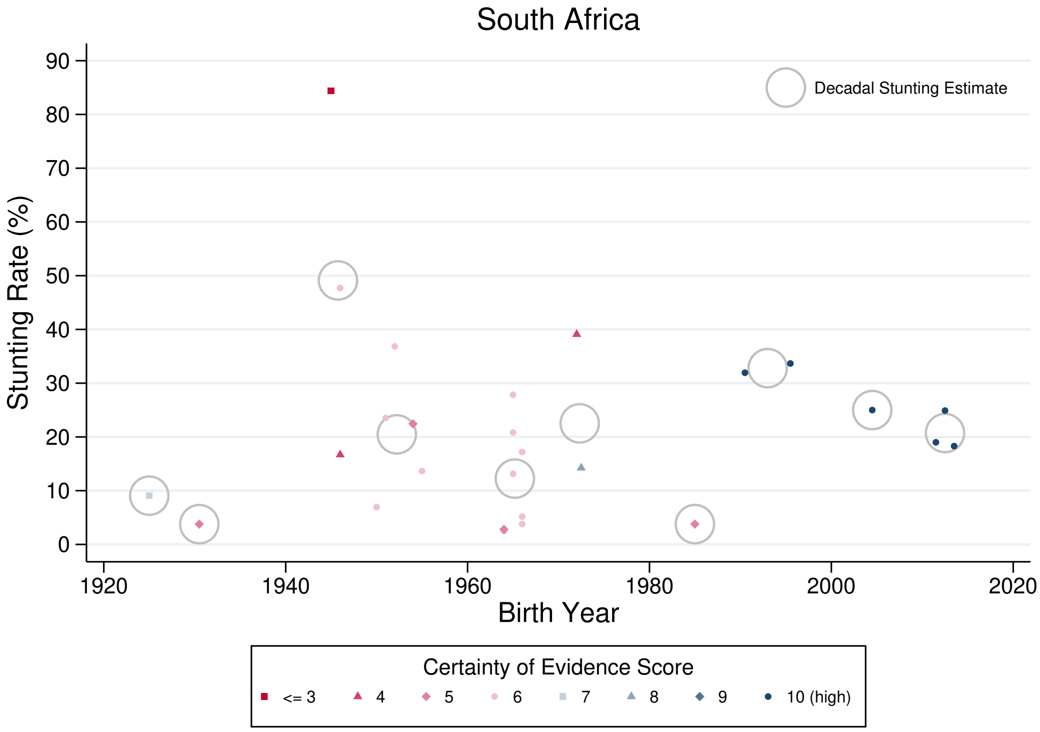

Figure O.96: South Africa Study-Level Stunting Rates and Certainty of Evidence Scores

Sources: Worldwide Historical Stunting Dataset.

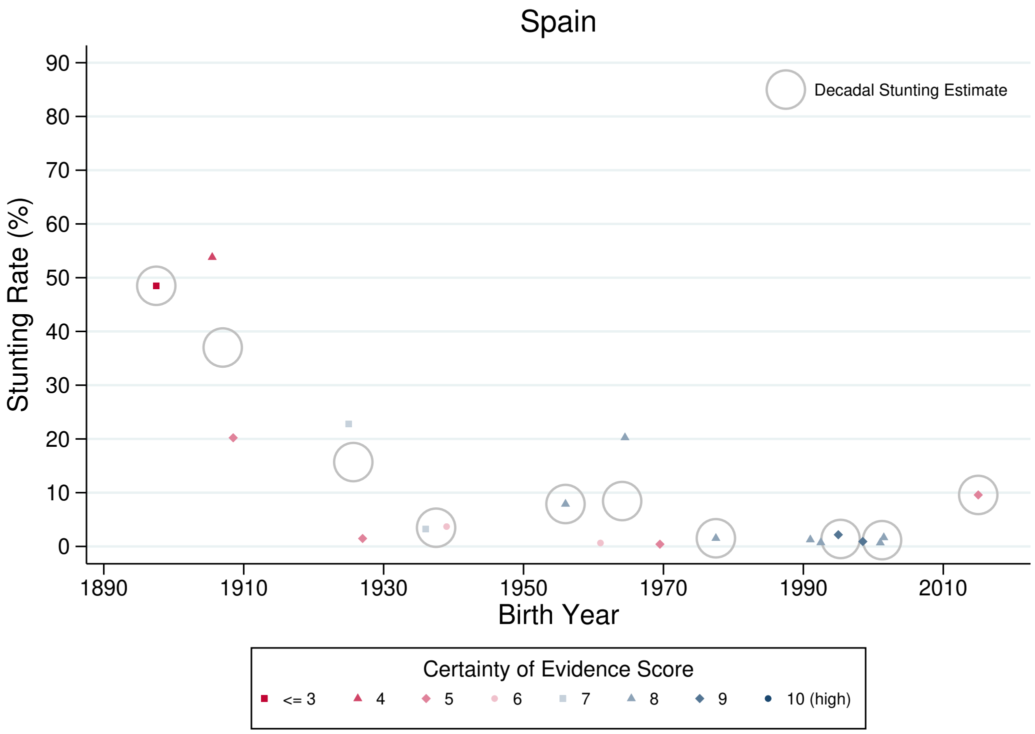

Figure O.97: Spain Study-Level Stunting Rates and Certainty of Evidence Scores

Sources: Worldwide Historical Stunting Dataset.

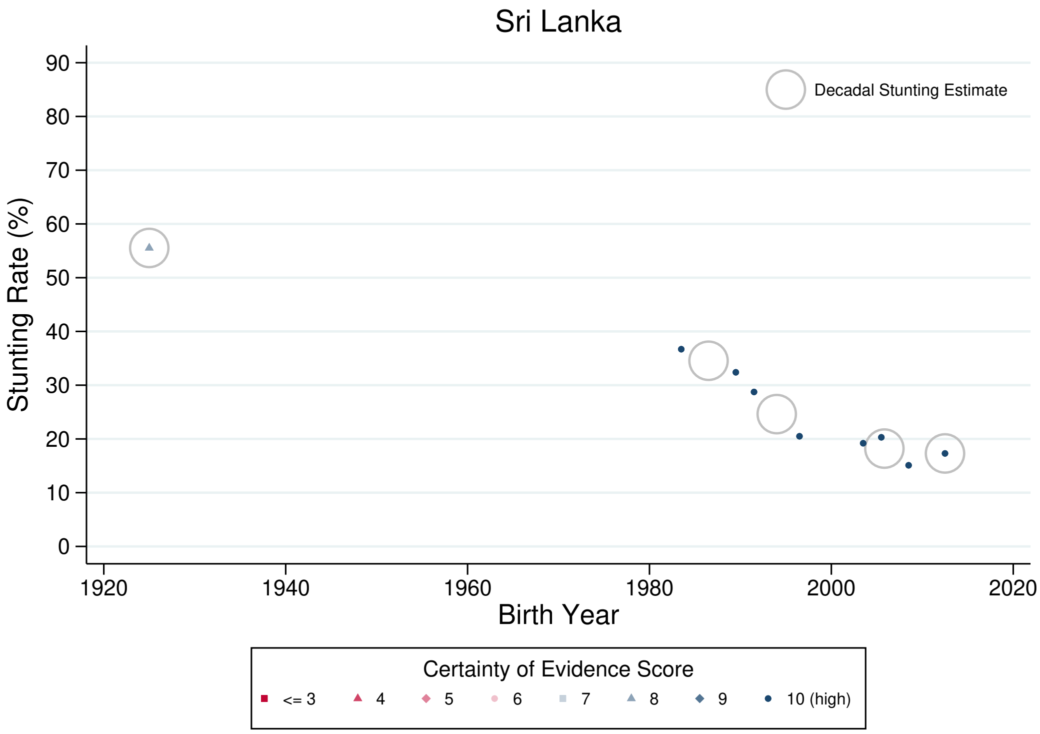

Figure O.98: Sri Lanka Study-Level Stunting Rates and Certainty of Evidence Scores

Sources: Worldwide Historical Stunting Dataset.

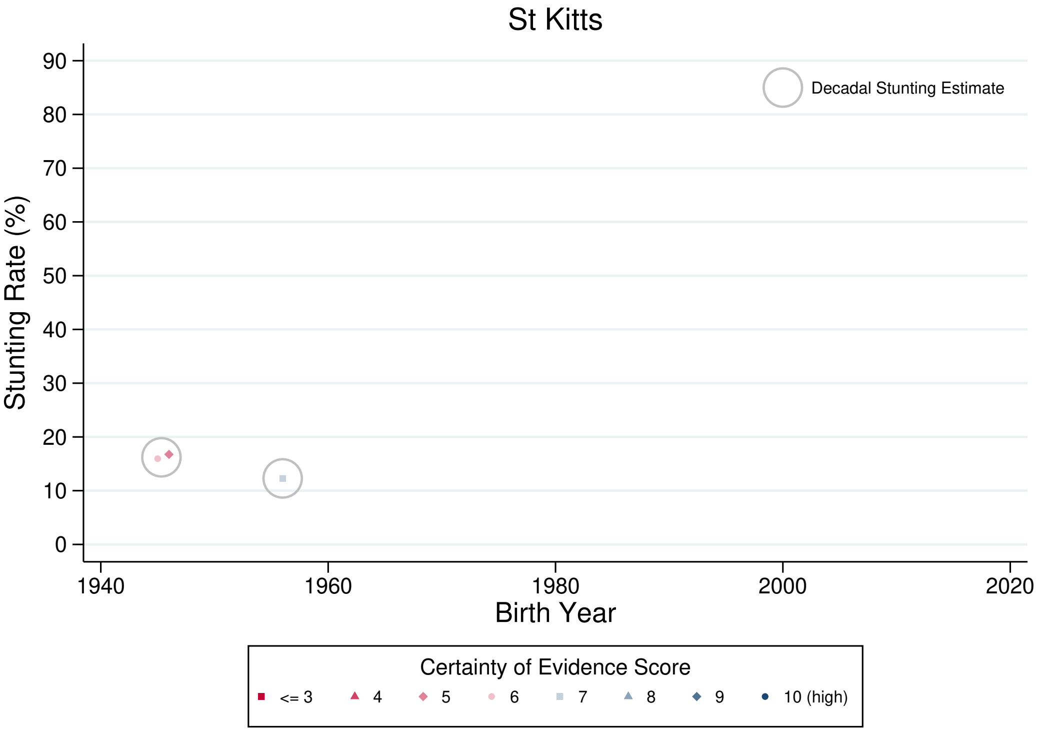

Figure O.99: St Kitts Study-Level Stunting Rates and Certainty of Evidence Scores

Sources: Worldwide Historical Stunting Dataset.

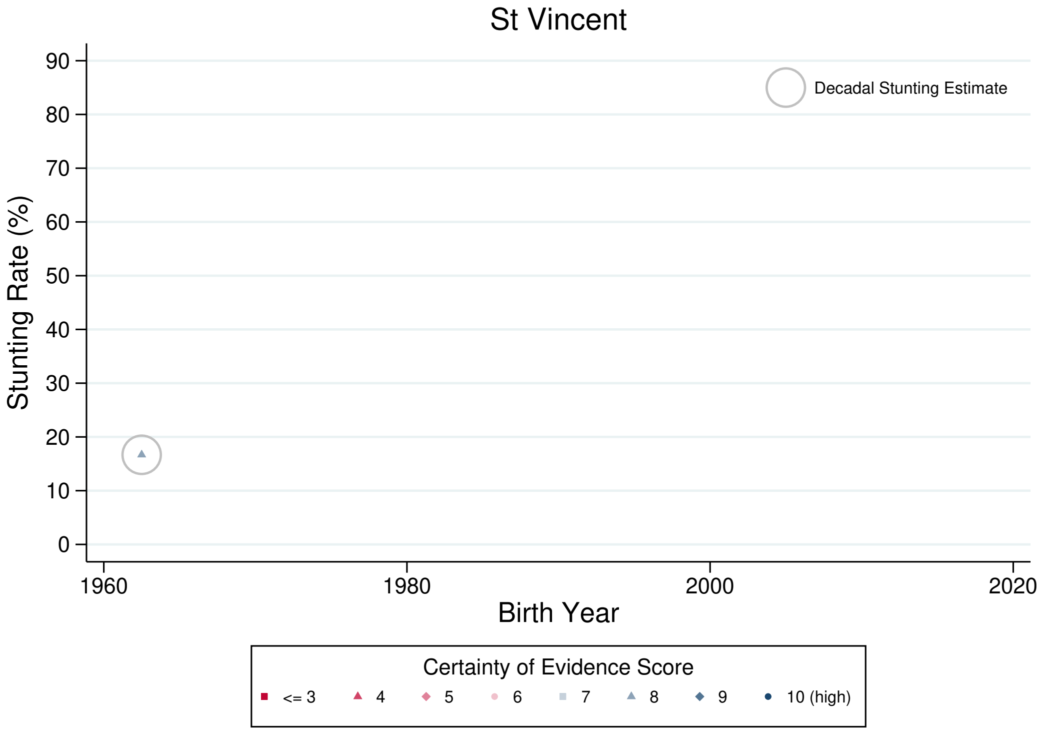

Figure O.100: St Vincent Study-Level Stunting Rates and Certainty of Evidence Scores

Sources: Worldwide Historical Stunting Dataset.

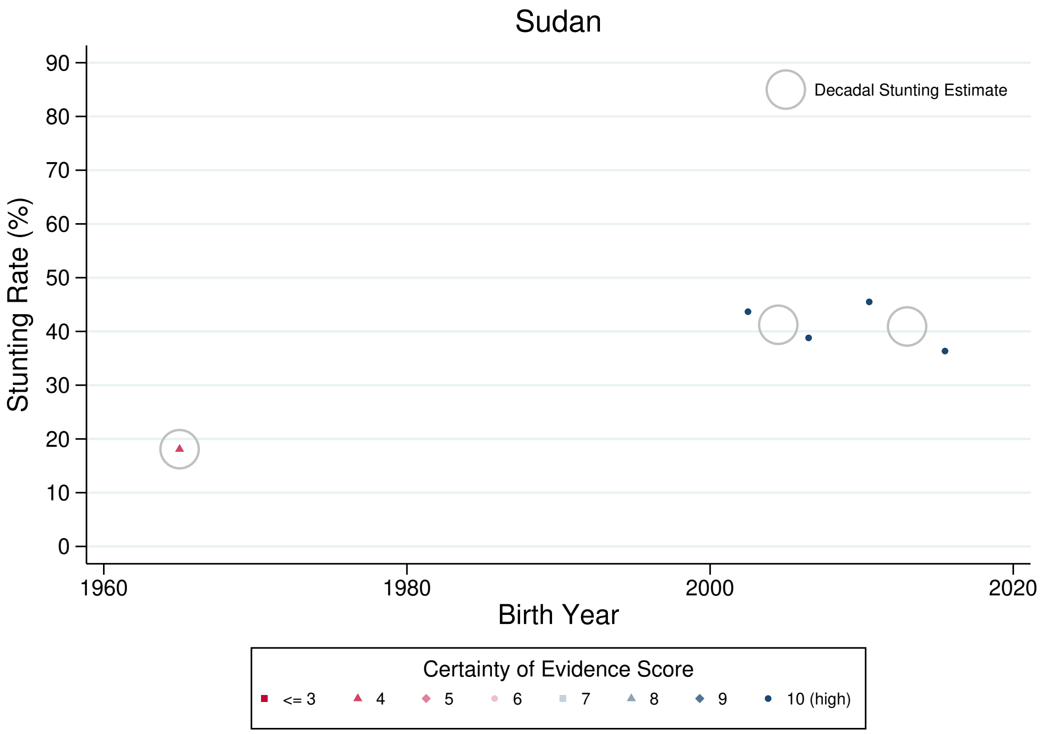

Figure O.101: Sudan Study-Level Stunting Rates and Certainty of Evidence Scores

Sources: Worldwide Historical Stunting Dataset.

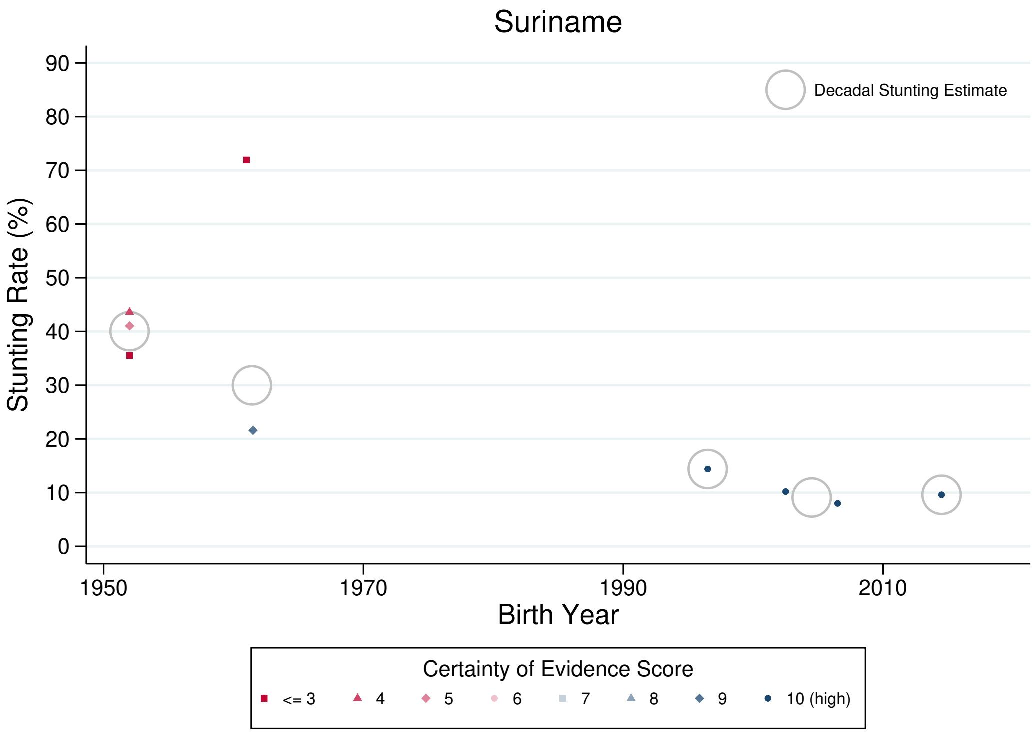

Figure O.102: Suriname Study-Level Stunting Rates and Certainty of Evidence Scores

Sources: Worldwide Historical Stunting Dataset.

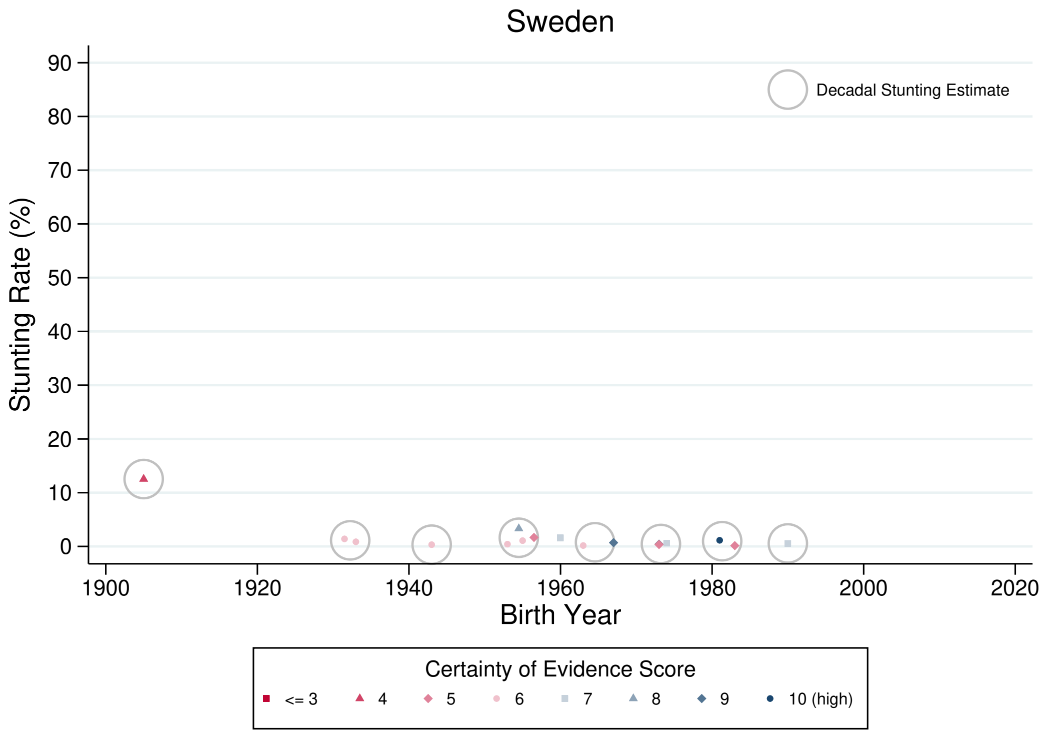

Figure O.103: Sweden Study-Level Stunting Rates and Certainty of Evidence Scores

Sources: Worldwide Historical Stunting Dataset.

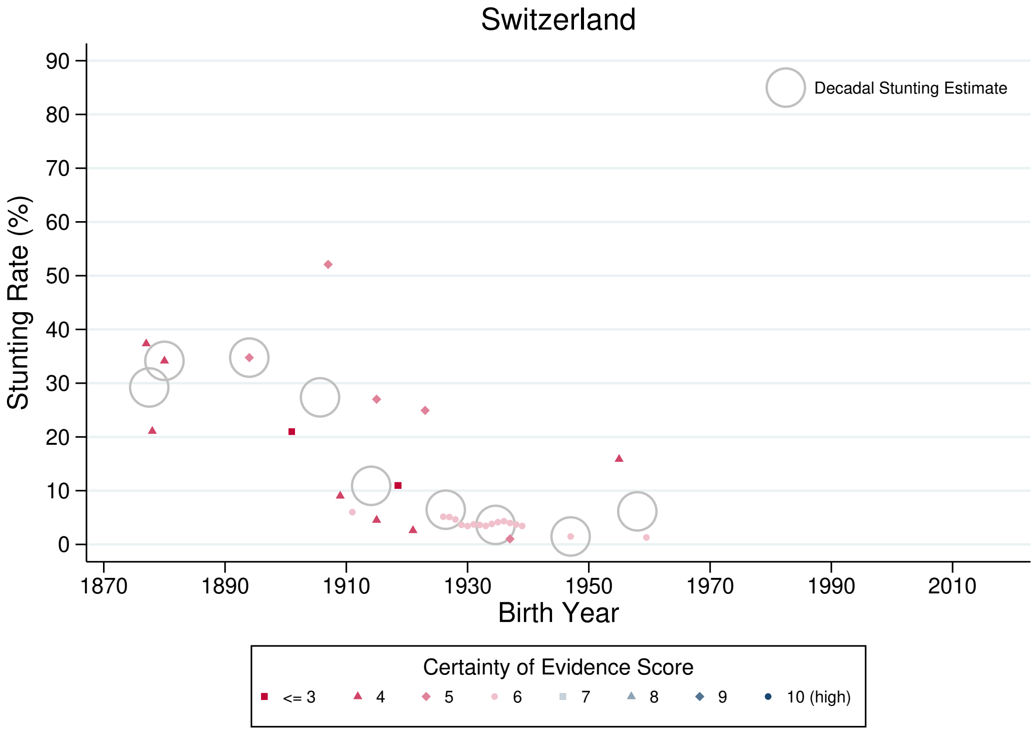

Figure O.104: Switzerland Study-Level Stunting Rates and Certainty of Evidence Scores

Sources: Worldwide Historical Stunting Dataset.

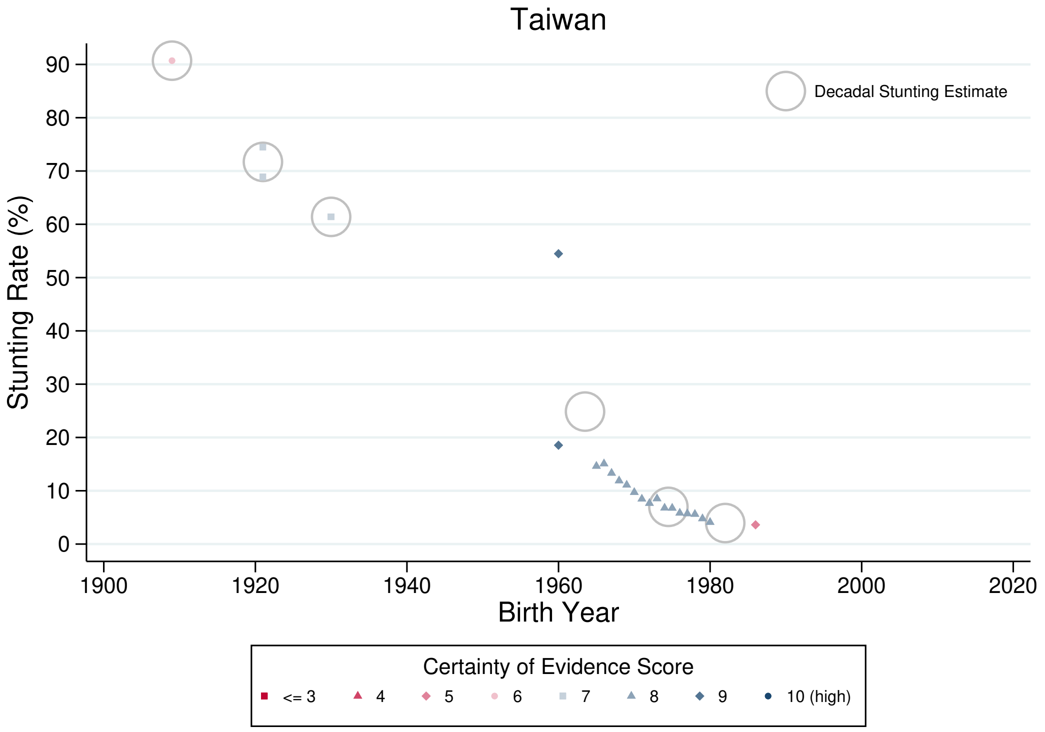

Figure O.105: Taiwan Study-Level Stunting Rates and Certainty of Evidence Scores

Sources: Worldwide Historical Stunting Dataset.

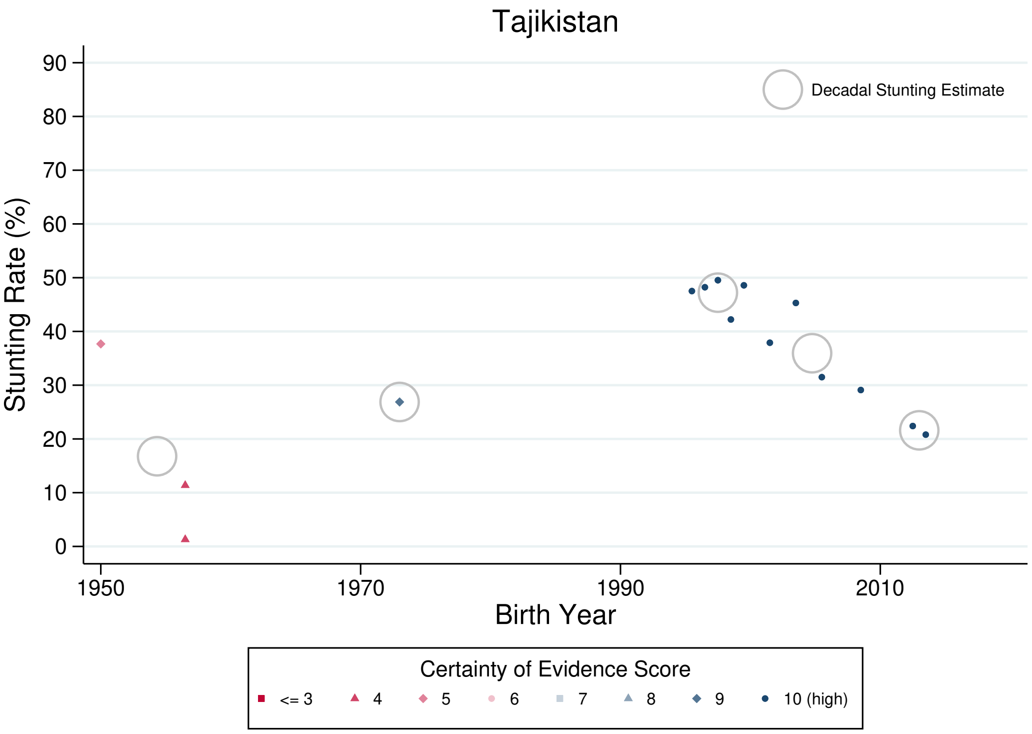

Figure O.106: Tajikistan Study-Level Stunting Rates and Certainty of Evidence Scores

Sources: Worldwide Historical Stunting Dataset.

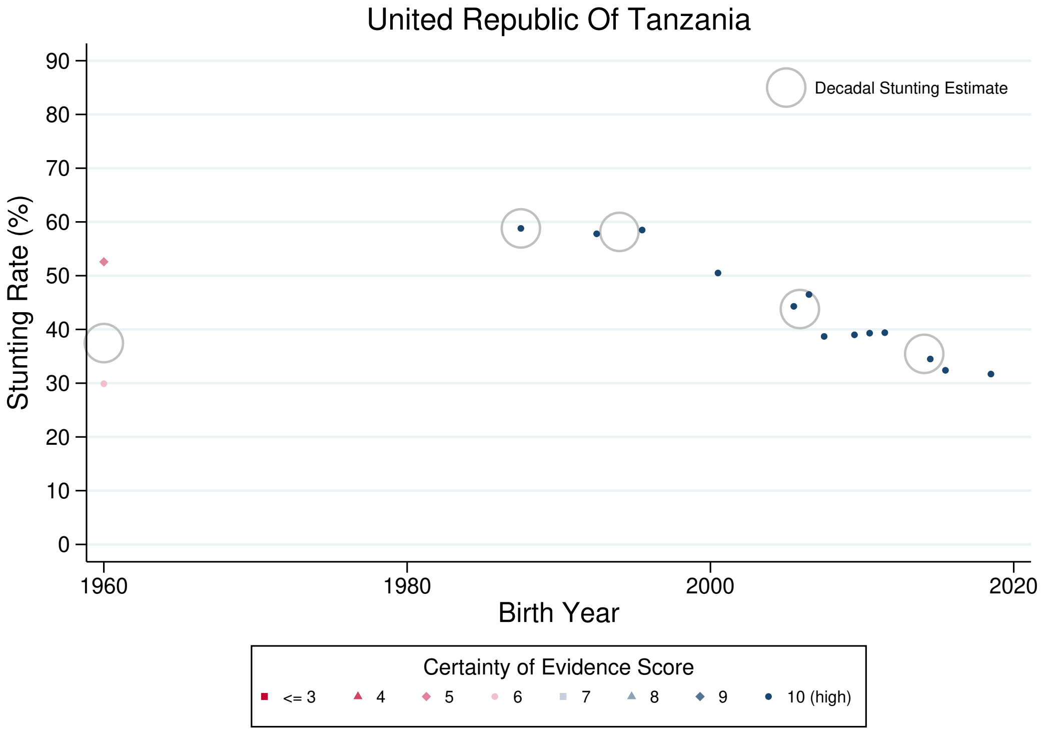

Figure O.107: Tanzania Study-Level Stunting Rates and Certainty of Evidence Scores

Sources: Worldwide Historical Stunting Dataset.

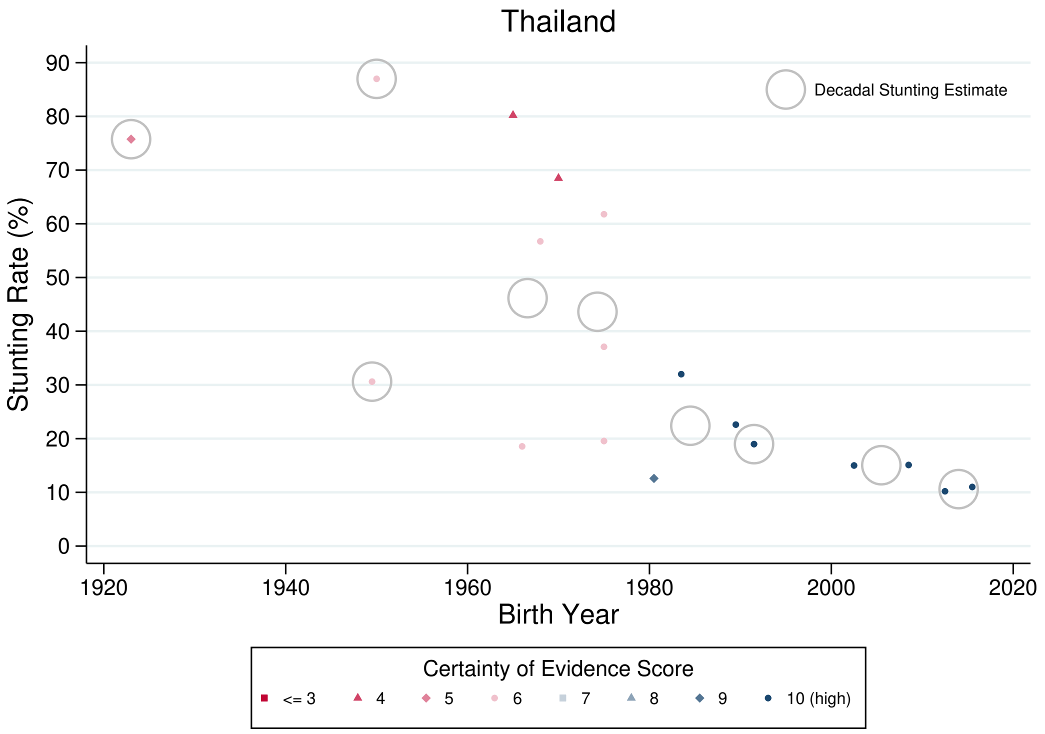

Figure O.108: Thailand Study-Level Stunting Rates and Certainty of Evidence Scores

Sources: Worldwide Historical Stunting Dataset.

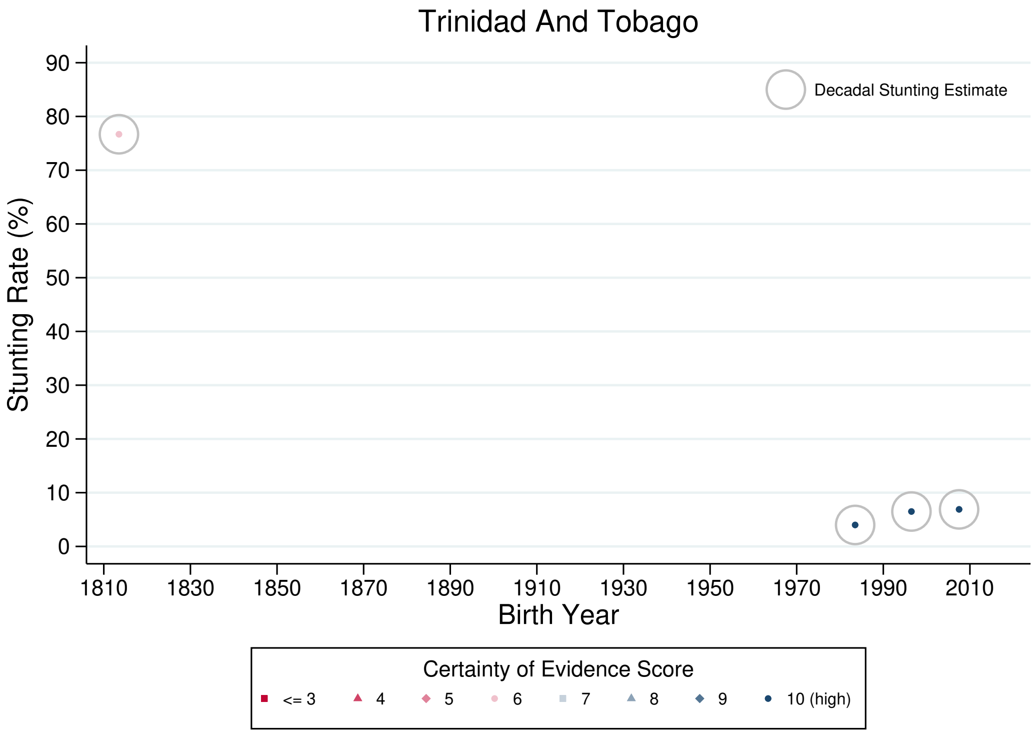

Figure O.109: Trinidad Study-Level Stunting Rates and Certainty of Evidence Scores

Sources: Worldwide Historical Stunting Dataset.

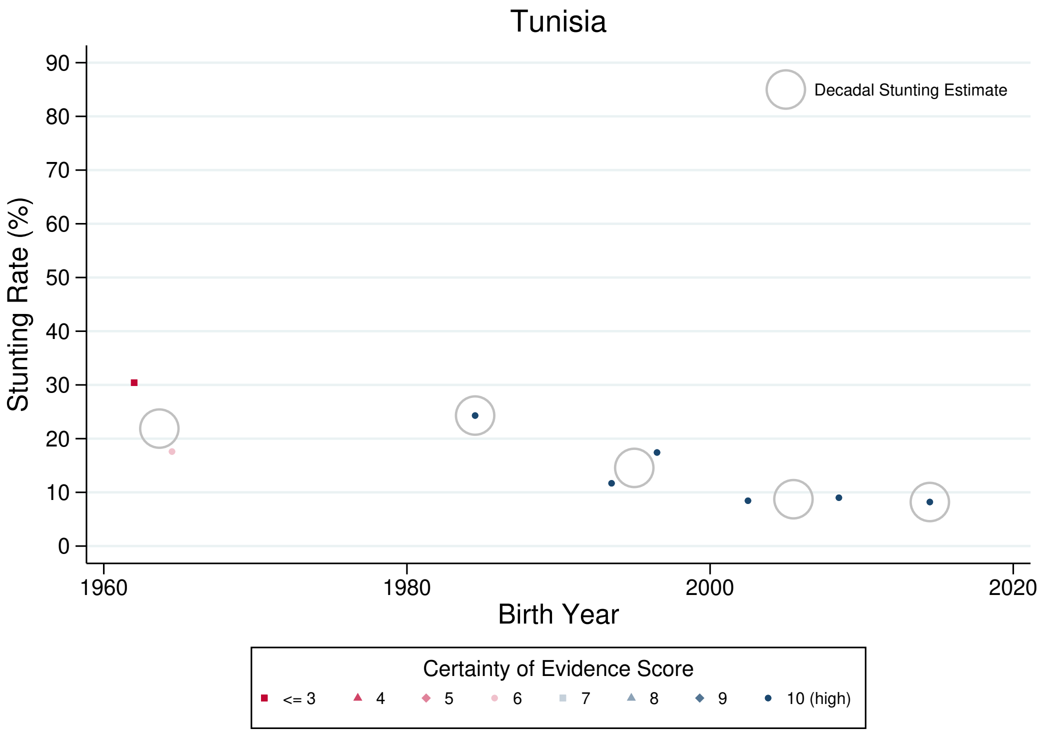

Figure O.110: Tunisia Study-Level Stunting Rates and Certainty of Evidence Scores

Sources: Worldwide Historical Stunting Dataset.

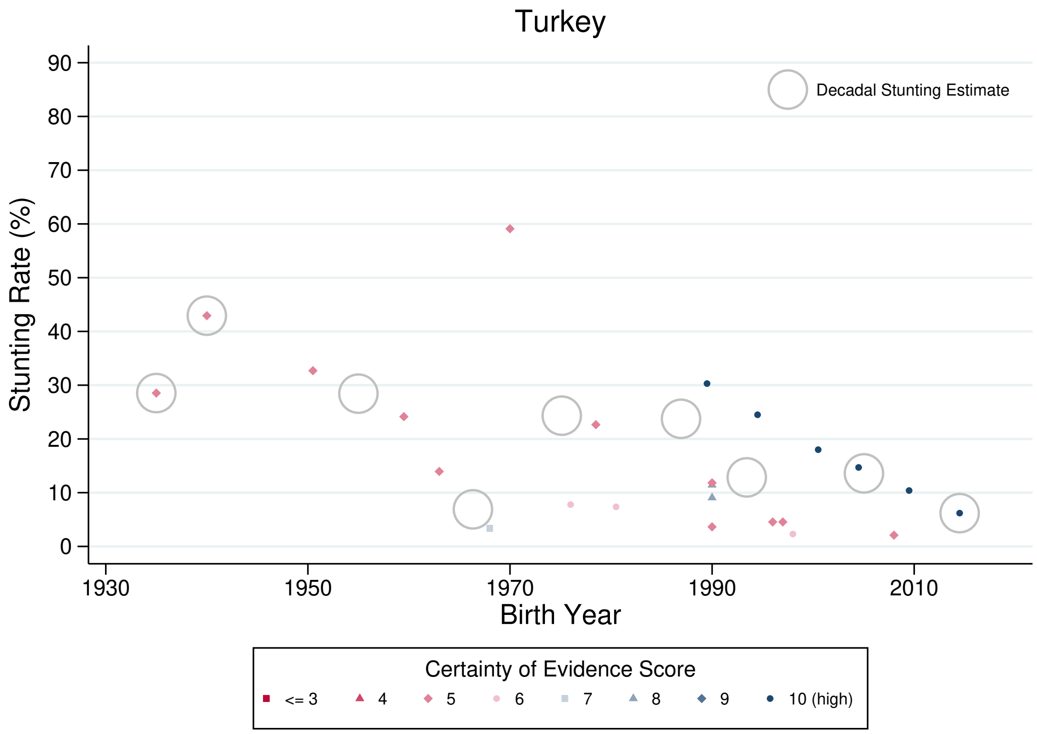

Figure O.111: Turkey Study-Level Stunting Rates and Certainty of Evidence Scores

Sources: Worldwide Historical Stunting Dataset.

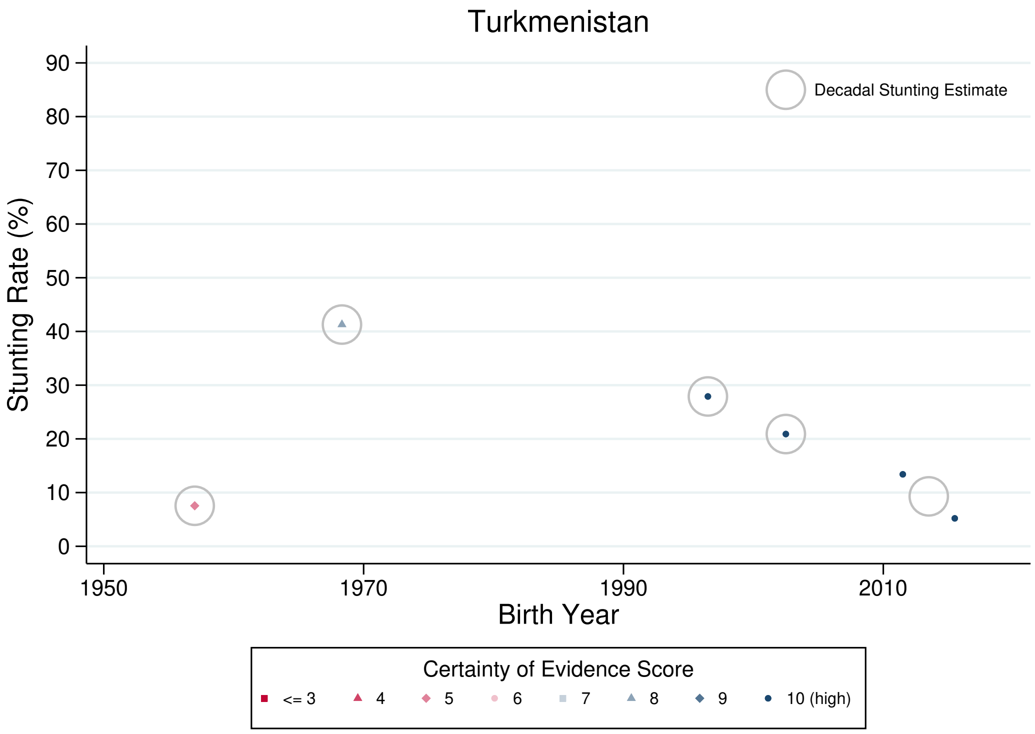

Figure O.112: Turkmenistan Study-Level Stunting Rates and Certainty of Evidence Scores

Sources: Worldwide Historical Stunting Dataset.

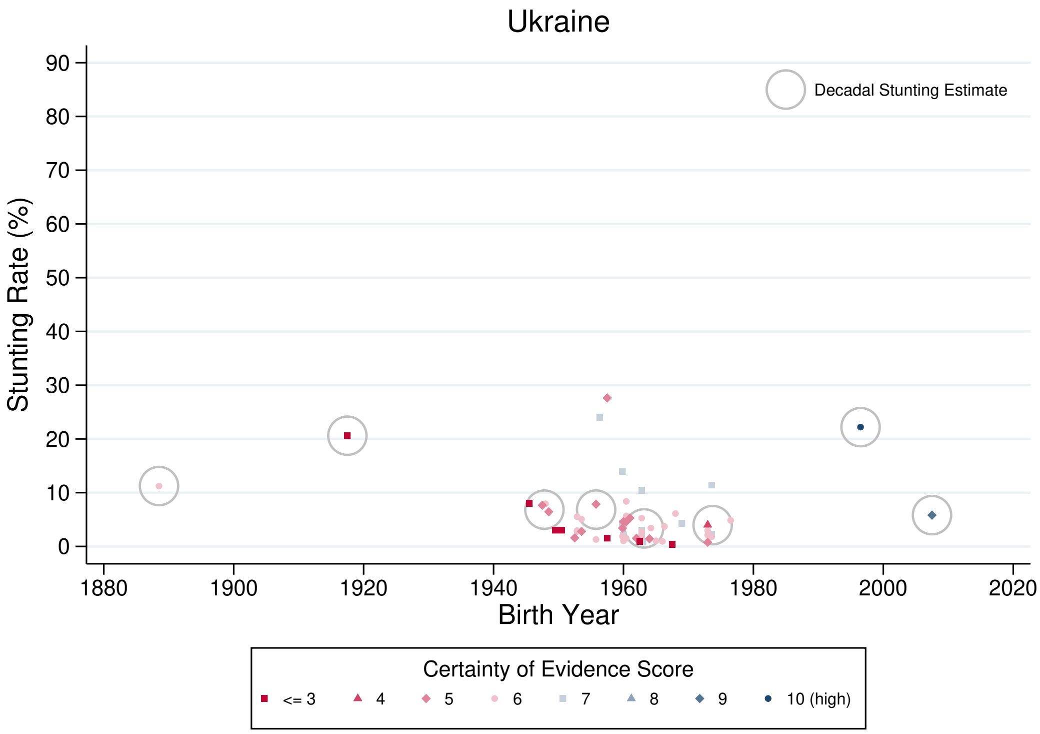

Figure O.113: Ukraine Study-Level Stunting Rates and Certainty of Evidence Scores

Sources: Worldwide Historical Stunting Dataset.

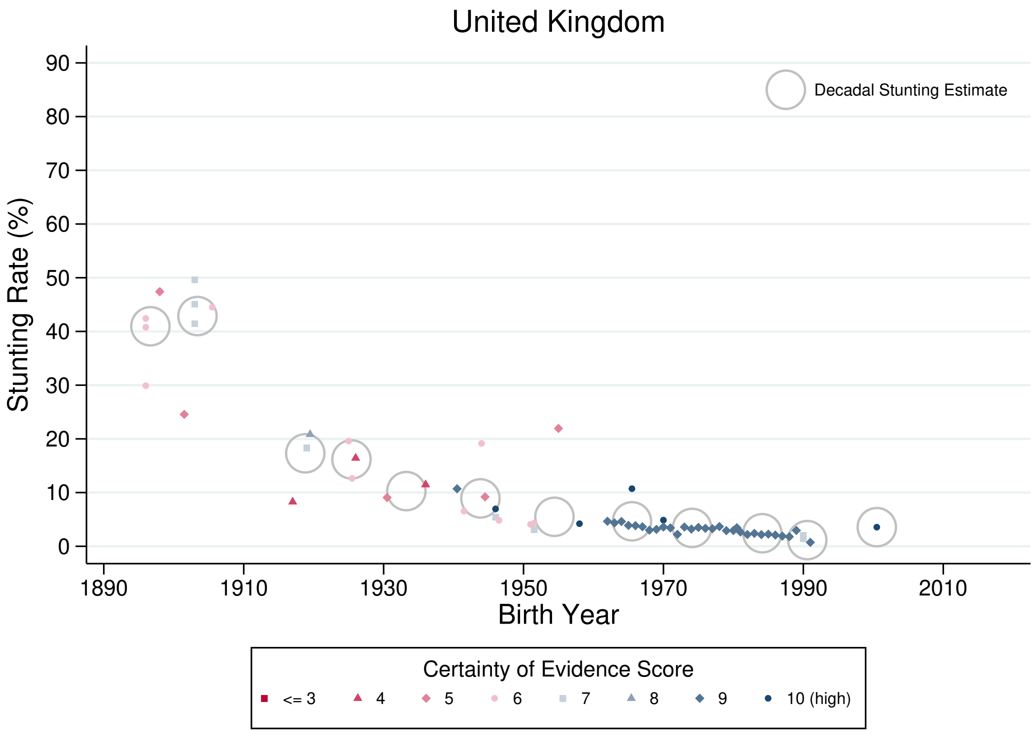

Figure O.114: United Kingdom Study-Level Stunting Rates and Certainty of Evidence Scores

Sources: Worldwide Historical Stunting Dataset.

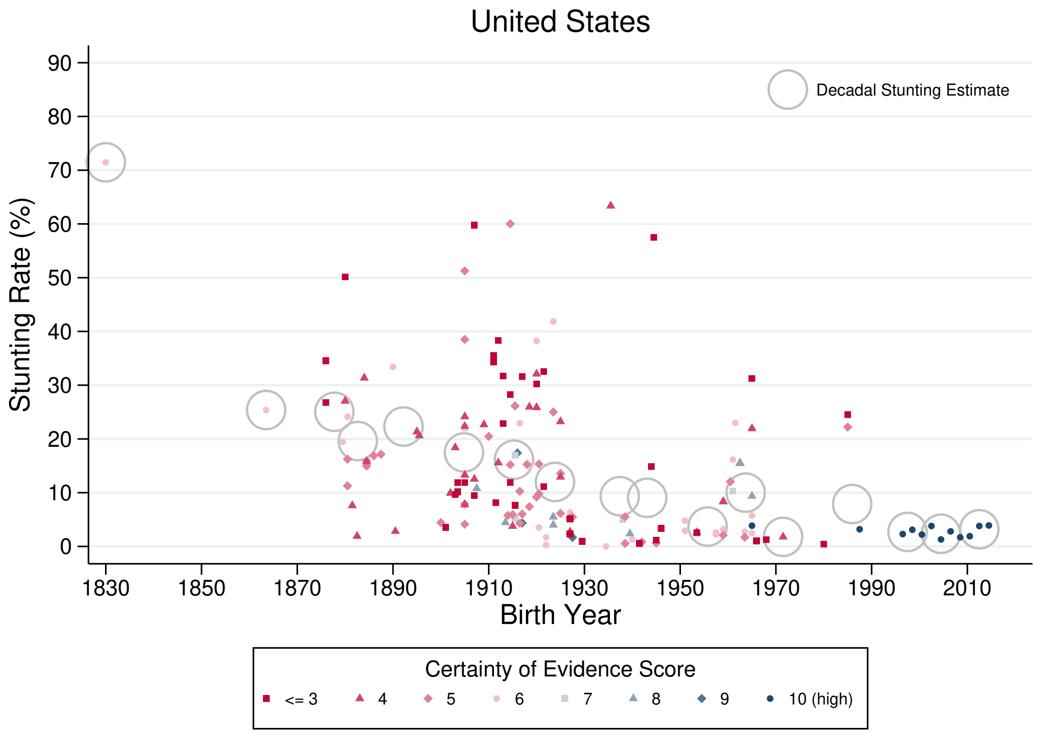

Figure O.115: United States Study-Level Stunting Rates and Certainty of Evidence Scores

Sources: Worldwide Historical Stunting Dataset.

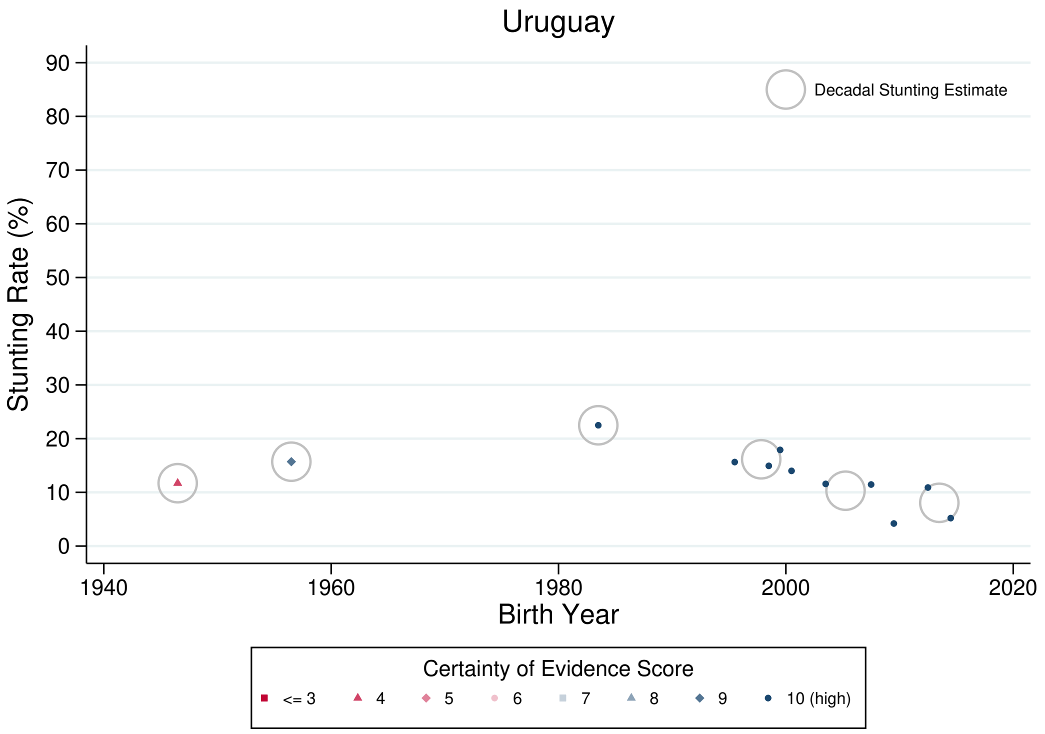

Figure O.116: Uruguay Study-Level Stunting Rates and Certainty of Evidence Scores

Sources: Worldwide Historical Stunting Dataset.

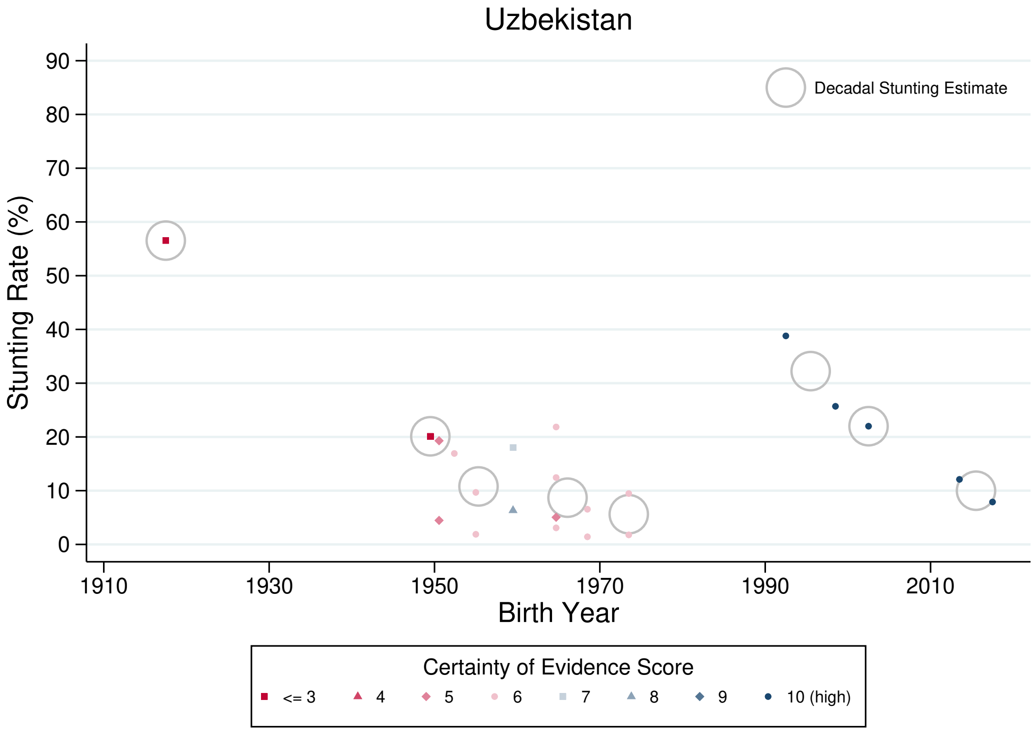

Figure O.117: Uzbekistan Study-Level Stunting Rates and Certainty of Evidence Scores

Sources: Worldwide Historical Stunting Dataset.

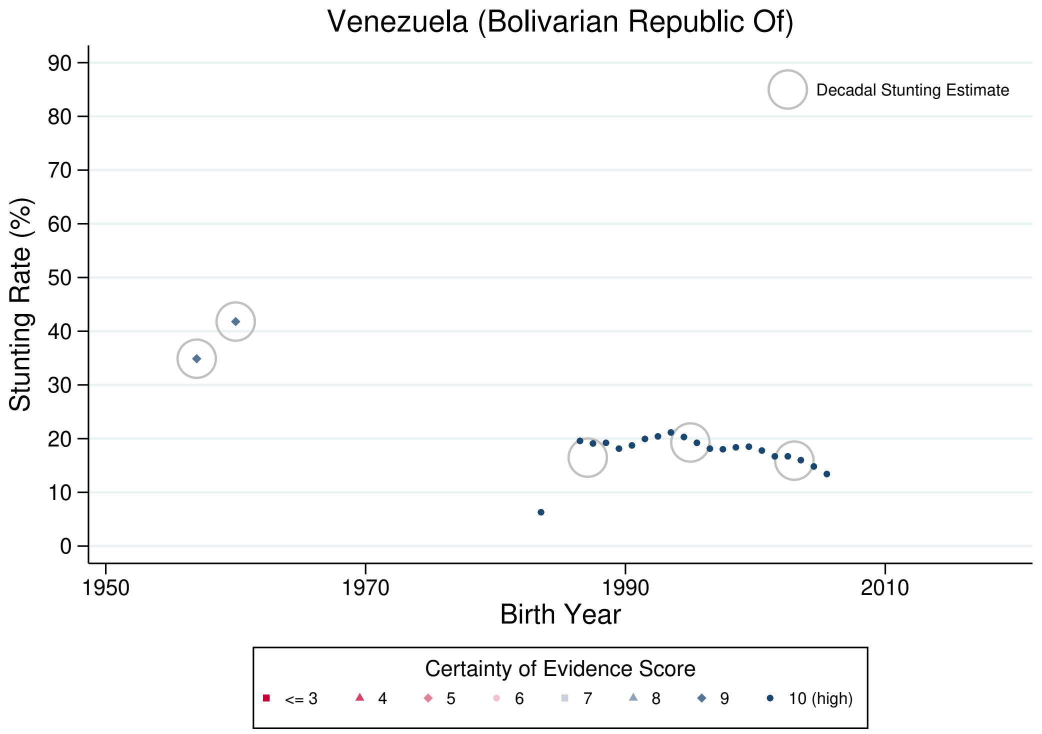

Figure O.118: Venezuela Study-Level Stunting Rates and Certainty of Evidence Scores

Sources: Worldwide Historical Stunting Dataset.

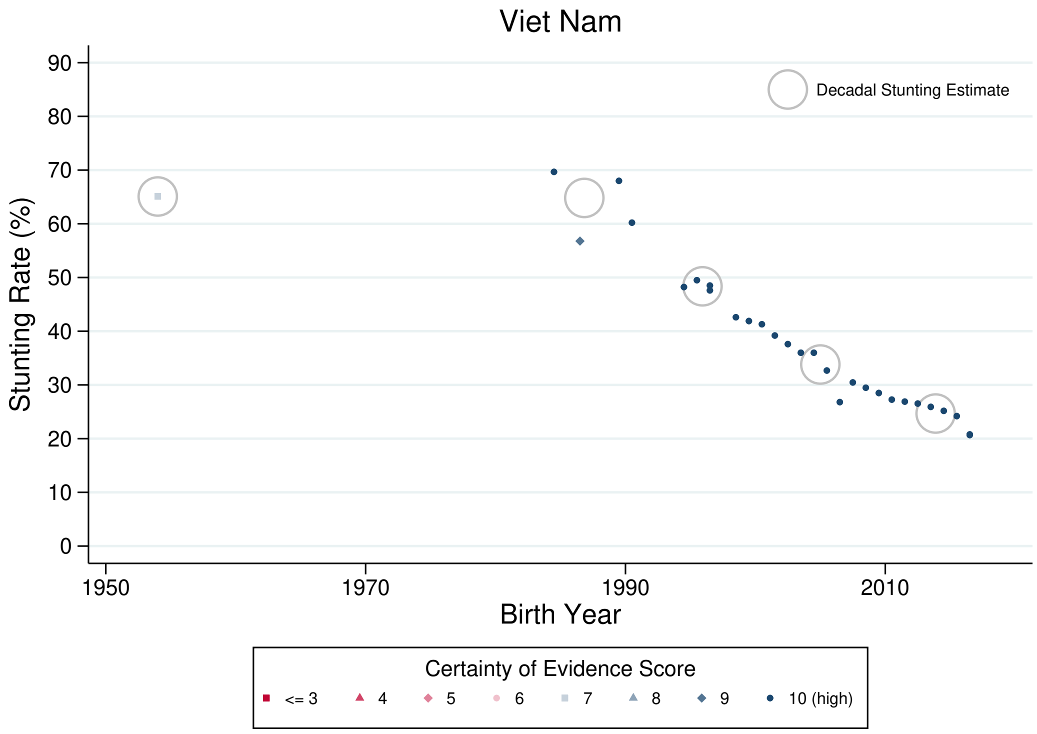

Figure O.119: Vietnam Study-Level Stunting Rates and Certainty of Evidence Scores

Sources: Worldwide Historical Stunting Dataset.

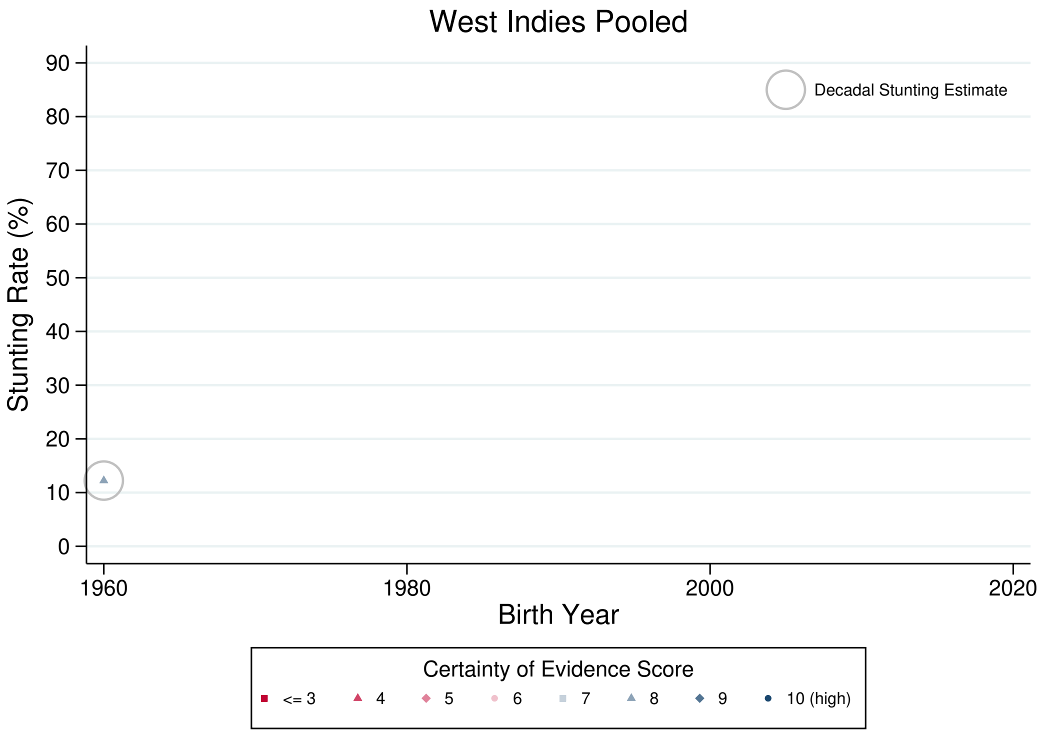

Figure O.120: West Indies Pooled Study-Level Stunting Rates and Certainty of Evidence Scores

Sources: Worldwide Historical Stunting Dataset.

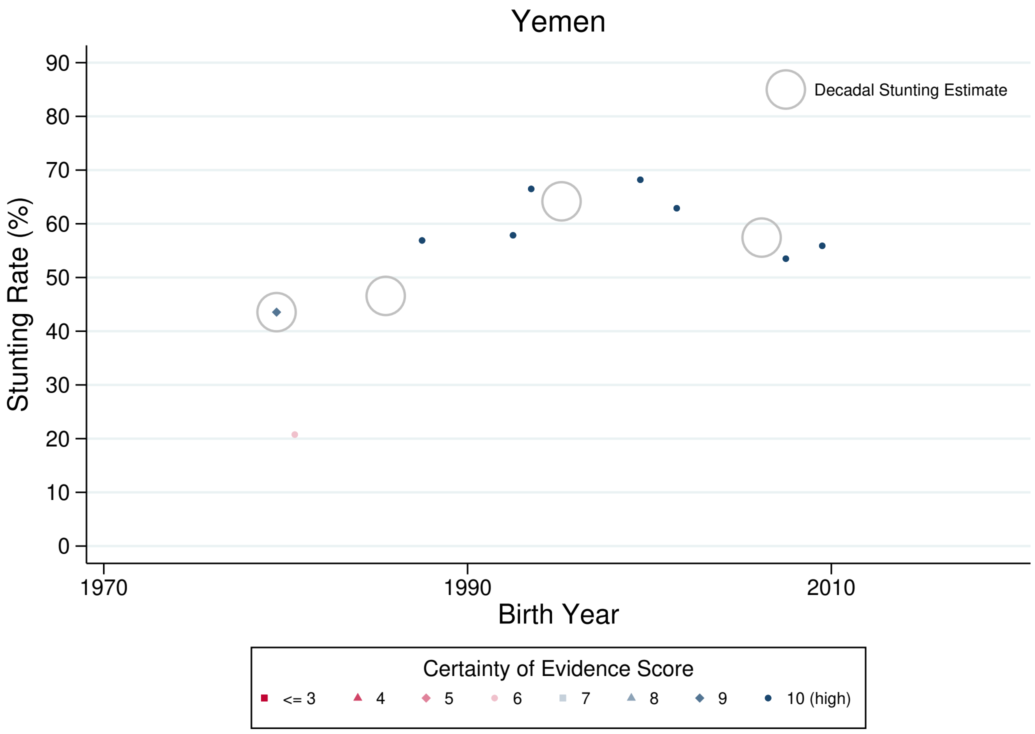

Figure O.121: Yemen Study-Level Stunting Rates and Certainty of Evidence Scores

Sources: Worldwide Historical Stunting Dataset.

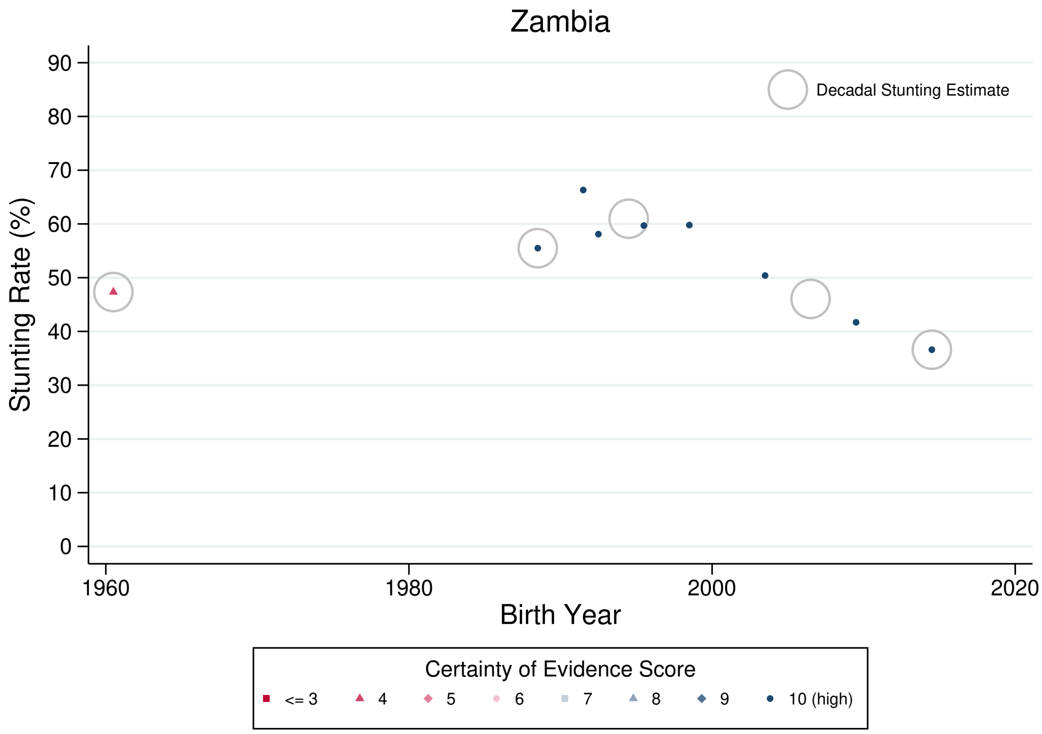

Figure O.122: Zambia Study-Level Stunting Rates and Certainty of Evidence Scores

Sources: Worldwide Historical Stunting Dataset.

P References

Aiyar, A. and Cummins, J. R. (2021). An age profile perspective on two puzzles in global child health: The Indian Enigma & economic growth. *Journal of Development Economics*, 148:102569.

Alderman, H. and Headey, D. (2018). The timing of growth faltering has important implications for observational analyses of the underlying determinants of nutrition outcomes. *PLoS ONE*, 13(4):e0195904 – 16.

Alderman, H., Hoddinott, J., and Kinsey, B. (2006). Long term consequences of early childhood malnutrition. *Oxford Economic Papers*, 58(3):450 – 474.

Alderman, H., Lokshin, M., and Radyakin, S. (2011). Tall claims: Mortality selection and the height of children in India. *Economics and Human Biology*, 9(4):393–406.

Almquist, A. (1994). The society and its environment. In Meditz, S. W. and Merrill, T., editors, *Zaire: A country study*, pages 61 – 134. US Government Printing Office, Washington, DC.

Bai, K. I., Raghavaprasad, K., Srinath, V., Kumar, R., and Reddy, C. O. (1979). Nutritional and anthropometric profile of primary school children in rural Andhra Pradesh. *Indian Pediatrics*, 12(16):1085–1090.

Baldwin, B. T. (1921). *The Physical Growth of Children from Birth to Maturity.* University of Iowa Press, Iowa City.

Benjamin-Chung, J., Mertens, A., Colford, J. M., Hubbard, A. E., Laan, M. J. v. d., Coyle, J., Sofrygin, O., Cai, W., Nguyen, A., Pokpongkiat, N. N., Djajadi, S., Seth, A., Jilek, W., Jung, E., Chung, E. O., Rosete, S., Hejazi, N., Malenica, I., Li, H., Hafen, R., Subramoney, V., Häggström, J., Norman, T., Brown, K. H., Christian, P., Arnold, B. F., Consortium, T. K. C. G., Abbeddou, S., Adair, L. S., Ahmed, T., Ali, A., Ali, H., Ashorn, P., Bahl, R., Barreto, M. L., Begín, F., Bessong, P. O., Bhan, M. K., Bhandari, N., Bhargava, S. K., Bhutta, Z. A., Black, R. E., Bodhidatta, L., Carba, D.,

Casanova, I. G., Checkley, W., Crabtree, J. E., Dewey, K. G., Duggan, C. P., Fall, C. H. D., Faruque, A. S. G., Fawzi, W. W., Filho, J. Q. d. S., Gilman, R. H., Guerrant, R. L., Haque, R., Hess, S. Y., Houpt, E. R., Humphrey, J. H., Iqbal, N. T., Jimenez, E. Y., John, J., John, S. M., Kang, G., Kosek, M., Kramer, M. S., Labrique, A., Lee, N. R., Lima, A. A. M., Mahfuz, M., Mahopo, T. C., Maleta, K., Manandhar, D. S., Manji, K. P., Martorell, R., Mazumder, S., Mduma, E., Mohan, V. R., Moore, S. E., Mostafa, I., Ntozini, R., Nyathi, M. E., Olortegui, M. P., Petri, W. A., Premkumar, P. S., Prentice, A. M., Sachdev, H. S., Sadiq, K., Sarkar, R., Saville, N. M., Shaikh, S., Shrestha, B. P., Shrestha, S. K., Soares, A. M., Sonko, B., Stein, A. D., Svensen, E., Syed, S., Umrani, F., Ward, H. D., West, K. P., Wu, L. S. F., Yang, S., and Yori, P. P. (2023). Early-childhood linear growth faltering in low- and middle-income countries. *Nature*, 621(7979):550–557.

Bhandari, B., Jain, A. M., Karna, P., Mathur, A., and Sharma, V. K. (1972). Nutritional anthropometry of rural school children of Udaipur District. *Indian Journal of Pediatrics*, 288(39):1–11.

Bowditch, H. (1877). *The growth of children.*

Bozzoli, C., Deaton, A., and Quintana-Domeque, C. (2009). Adult height and childhood disease. *Demography*, 46(4):647–669.

Brainerd, E. (2010). Reassessing the Standard of Living in the Soviet Union: An Analysis Using Archival and Anthropometric Data. *The Journal of Economic History*, 70(1):83 – 117.

Burk, F. (1898). Growth of Children in Height and Weight. *The American Journal of Psychology*, 9(3):253–326.

Cameron, N. (1979). The growth of London schoolchildren 1904-1966: an analysis of secular trend and intra-county variation. *Annals of Human Biology*, 6(6):505–525.

Central Bureau of Statistics (1979). Report of the Child Nutrition Survey 1978/79. Technical report, Central Bureau of Statistics, Nairobi.

Central Bureau of Statistics - CBS/Kenya, Ministry of Health - MOH/Kenya, and ORC Macro (2003). Kenya Demographic and Health Survey 2003 [Dataset]. Calverton, Maryland, USA: CBS, MOH, and ORC Macro [Producers]. ICF [Distributor], 2023.

Coovadia, H. M., Adhikari, M., and Mthethwa, D. (1978). Physical Growth of Negro Children in the Durban Area. *Tropical and Geographical Medicine*, 30(3):373–381.

de Onis, M., Garza, C., Victora, C. G., Onyango, A. W., Frongillo, E. A., and Martines, J. (2004). The WHO Multicentre Growth Reference Study: planning, study design, and methodology. *Food and Nutrition Bulletin*, 25(1 Suppl):S15 – S26.

de Onis, M., Onyango, A., Borghi, E., Siyam, A., Blössner, M., Lutter, C., and Group, W. M. G. R. S. (2012). Worldwide implementation of the WHO Child Growth Standards. *Public Health Nutrition*, 15(9):1603–1610.

de Onis, M., Onyango, A. W., Borghi, E., Siyam, A., Nishida, C., and Siekmann, J. (2007). Development of a WHO growth reference for school-aged children and adolescents. *Bulletin of the World Health Organization*, 85(9):660 – 667.

Deaton, A. (2007). Height, health, and development. *Proceedings of the National Academy of Sciences*, 104(33):13232.

Eveleth, P. B. and Tanner, J. M. (1976). *Worldwide variation in human growth.* Cambridge University Press, Cambridge.

Eveleth, P. B. and Tanner, J. M. (1991). *Worldwide variation in human growth, 2nd edition.* Cambridge University Press, Cambridge.

Finucane, M. M., Paciorek, C. J., Stevens, G. A., and Ezzati, M. (2015). Semiparametric Bayesian Density Estimation With Disparate Data Sources: A Meta-Analysis of Global Childhood Undernutrition. *Journal of the American Statistical Association*, 110(511):889–901.

Gao, P. and Schneider, E. B. (2021). The growth pattern of British children, 1850–1975. *The Economic History Review*, 74(2):341–371.

Golden, M. H. (1994). Is complete catch-up possible for stunted malnourished children? *European Journal of Clinical Nutrition*, 48 Suppl 1:S58 – 70– discussion S71.

Habakkuk, E. (1926). A Statistical Study of the Physique of Elementary School Children with special reference to their Mentality. *The Journal of Hygiene*, 25(3):295.

Healy, M. J. R. (1962). The effect of age-grouping on the distribution of a measurement affected by growth. *American Journal of Physical Anthropology*, 20(1):49–50.

Hermanussen, M., Bogin, B., and Scheffler, C. (2018). Stunting, starvation and refeeding: a review of forgotten 19th and early 20th century literature. *Acta Paediatrica*, 107(7):1166–1176.

Hermanussen, M., Stec, K., Aßmann, C., Meigen, C., and van Buuren, S. (2016). Synthetic growth reference charts. *American Journal of Human Biology*, 28(1):98–111.

Higgins, J. P. T., Li, T., and Deeks, J. J. (2019). Chapter 6: Choosing Effect Measures and Computing Estimates of Effect. In Higgins, J., Thomas, J., Chandler, J., Cumpston, M., Li, T., Page, M., and Welch, V., editors, *Cochrane Handbook for Systematic Reviews of Interventions*, pages 143–176. John Wiley & Sons, Chichester (UK).

Higman, B. (1979). Growth in Afro-Caribbean slave populations. *American Journal of Physical Anthropology*, 50(3):373–385.

Hoorweg, J., Niemeijer, R., and van Steenbergen, W. (1983). Nutrition Survey in Murang'a District, Kenya. Technical report, African Studies Centre, Leiden.

Indian Council of Medical Research (1972). Growth and Physical Development of Indian Infants and Children. Technical report.

Jansen, A. A. J. (1984). Weight, Height, Weight-For-Height and Quetelet's Index of Akamba Schoolchildren and Adults. *East African Medical Journal*, 61(4):272 – 282.

Jedwab, R., Selhausen, F. M. z., and Moradi, A. (2022). The economics of missionary expansion: evidence from Africa and implications for development. *Journal of Economic Growth*, 27(2):149–192.

Jelliffe, D. B. (1966). *The Assessment of Nutritional Status of the Community (with special reference to field surveys developing regions of the world).* World Helath Organization, Geneva.

Kark, S. and le Riche, H. (1944). The Nutrition and Health of South African Bantu School Children - Somatometrical and clinical study. Technical report, Department of Public Health, Pretoria.

Kasongo Project Team (1982). Weight, height and arm circumference in 0 to 5 year-old children from Kasongo (Zaire). *Ecology of Food and Nutrition*, 12(1):19–28.

Kenya National Bureau of Statistics, Ministry of Health/Kenya, National AIDS Control Council/Kenya, Kenya Medical Research Institute, National Council for Population and Development/Kenya, and ICF International (2014). Kenya Demographic and Health Survey 2014 [Dataset]. Rockville, MD, USA: Kenya National Bureau of Statistics, Ministry of Health/Kenya, National AIDS Control Council/Kenya, Kenya Medical Research Institute, National Council for Population and Development/Kenya, and ICF International. [Producers]. ICF [Distributor], 2023.

Kim, J., Liu, Y., Wang, W., Blossom, J. C., Dwivedi, L. K., James, K. S., Sarwal, R., Kim, R., and Subramanian, S. (2021). Estimating the Burden of Child Undernutrition for Smaller Electoral Units in India. *JAMA Network Open*, 4(10):e2129416.

Kotzé, J. P., Merwe, G. J. v. d., Mostert, W. P., Reynders, J. J., Barnard, S. O., and Snyman, N. (1982). Anthropometric Survey of Different Cultural Groups in South Africa. *Journal of Dietetics and Home Economics*, 10(2):77–81.

Lassance, M., Peeters, E., and J, H. (1957). Tables biométriques de la population scolaire du Centre extra-coutumier de Stanleyville. *Annales de la Société Belge de Médicine Tropicale*, 37(5):631 – 639.

Lemarchand, R. (1994). Historical setting. In Meditz, S. W. and Merrill, T., editors, *Zaire: A country study*, pages 4 – 60. US Government Printing Office, Washington, DC.

Leroy, J. L., Ruel, M., Habicht, J.-P., and Frongillo, E. A. (2015). Using height-for-age differences (HAD) instead of height-for-age z-scores (HAZ) for the meaningful measurement of population-level catch-up in linear growth in children less than 5 years of age. *BMC Pediatrics*, 15(1):1 – 11.

Leys, N. M. and Joyce, T. A. (1913). Note on a Series of Physical Measurements from East Africa. *The Journal of the Royal Anthropological Institute of Great Britain and Ireland*, 43:195 – 267.

Lundeen, E. A., Stein, A. D., Adair, L. S., Behrman, J. R., Bhargava, S. K., Dearden, K. A., Gigante, D., Norris, S. A., Richter, L. M., Fall, C. H., Martorell, R., Sachdev, H. S., Victora, C. G., and Investigators, o. b. o. t. C. (2014). Height-for-age z scores increase despite increasing height deficits among children in 5 developing countries. *American Journal of Clinical Nutrition*, 100(3):821 – 825.

Lurie, G. and Ford, F. (1935). Our Land: Is our population satisfactory? The results of Inspection of Children of School Ages. *South African Medical Journal*, 9(23):819–824.

Lurie, G. and Ford, F. (1988). Heights and weights of European and Coloured Schoolchildren in Cape Town. *South African Medical Journal*, 32(42):1017–1025.

MacKay, D. H. and Martin, W. J. (1952). Dentition and Physique of Bantu Children. *The Journal of Tropical Medicine and Hygiene*, 55:265 – 275.

Martorell, R., Schroeder, D. G., Rivera, J. A., and Kaplowitz, H. J. (1995). Patterns of linear growth in rural Guatemalan adolescents and children. *The Journal of nutrition*, 125(4 Suppl):1060S–1067S.

Martínez-Carrión, J.-M. and Moreno-Lázaro, J. (2007). Was there an urban height penalty in Spain, 1840–1913? *Economics & Human Biology*, 5(1):144–164.

Meredith, H. V. (1964). Change in The Stature and Body Weight of North American Boys During the Last 80 Years. *Advances in Child Development and Behavior*, 1:69–114.

Meredith, H. V. (1976). Findings from Asia, Australia, Europe, and North America on secular change in mean height of children, youths, and young adults. *American Journal of Physical Anthropology*, 44(2):315–325.

Moradi, A. (2009). Towards an objective account of nutrition and health in colonial Kenya: A study of stature in African army recruits and civilians, 1880-1980. *Journal of Economic History*, 69(3):719.

Moradi, A. (2010). Nutritional status and economic development in sub-Saharan Africa, 1950–1980. *Economics and Human Biology*, 8(1):16 – 29.

Mukerji, B. (1970). Anthropometric observations on urban primary school children. *Indian Journal of Medical Research*, 9(58):1257–1271.

Naik, P., Zopf, T., Kakar, D., Sing, M., and Sandhu, S. (1975). Primary school children in rural Punjab: Nutritional and anthropometric profile. *Indian Pediatrics*, 11(12):1083–1097.

National Council for Population and Development - NCPD/Kenya, Central Bureau of Statistics - CBS/Kenya, and Macro International (1993). Kenya Demographic and Health Survey 1993 [Dataset]. Calverton, Maryland, USA: NCPD, CBS, and Macro International [Producers]. ICF [Distributor], 2023.

NCD Risk Factor Collaboration (2016). A century of trends in adult human height. *eLife*, pages 1–29.

Nichols, L. (1936). A Nutritional Survey of the Poorer Classes in Ceylon. *Ceylon Journal of Science*, 4(1):1–70.

Orr, J. B. and Gilks, J. R. (1931). Studies of Nutrition: The Physique and Health of Two African Tribes. Technical Report Series, No. 155, Medical Research Council, London.

Papadimitriou, A., Chiotis, D., Tsiftis, G., Hatzisimeon, M., Maniati, M., Krikos, X., Tzonou, A., and Dacou-Voutetakis, C. (2002). Secular growth changes in the Hellenic population in the twentieth century. *HORMONES*, 1(4):245–250.

Parent, M. (1959). Contribution à l'étude de l'état de nutrition des enfants au Katanga. *Mémoires de l'Académie Royale des Sciences Coloniales*, 8(3):1 – 46.

Porter, W. T. (1894). The Growth of St. Louis Children. *Transactions of the Academy of Science of St. Louis*, 6(12):263–380.

Prentice, A. M., Ward, K. A., Goldberg, G. R., Jarjou, L. M., Moore, S. E., Fulford, A. J., and Prentice, A. (2013). Critical windows for nutritional interventions against stunting. *American Journal of Clinical Nutrition*, 97(5):911 – 918.

Ramon-Muñoz, R. and Ramon-Muñoz, J.-M. (2024). The urban–rural height gap: evidence from late nineteenth-century Catalonia. *Cliometrica*, 18(1):103–149.

Roberts, E. and Warren, J. R. (2017). Family structure and childhood anthropometry in Saint Paul, Minnesota in 1918. *The History of the Family*, 22(2-3):258–290.

Robertson, A. S. (1952). Height and weight tables of pre-school African native children. *Transactions of The Royal Society of Tropical Medicine and Hygiene*, 46(5):560–563.

Rosenbaum, S. (1988). 100 Years of Heights and Weights. *Journal of the Royal Statistical Society: Series A (Statistics in Society)*, 151(2):276–309.

Roth, D. E., Krishna, A., Leung, M., Shi, J., Bassani, D. G., and Barros, A. J. D. (2017). Early childhood linear growth faltering in low-income and middle-income countries as a whole-population condition: analysis of 179 Demographic and Health Surveys from 64 countries (1993-2015). *The Lancet Global Health*, 5(12):e1249 – e1257.

Schneider, E. B. (2016). Health, Gender and the Household: Children's Growth in the Marcella Street Home, Boston, MA, and the Ashford School, London, UK. *Research in Economic History*, 32:277–361.

Schneider, E. B. (2017). Children's growth in an adaptive framework: explaining the growth patterns of American slaves and other historical populations. *The Economic History Review*, 70(1):3 – 29.

Schneider, E. B. (2020). Sample-Selection Biases and the Historical Growth Pattern of Children. *Social Science History*.

Schneider, E. B. (2024). The determinants of child stunting and shifts in the growth pattern of children: A long-run, global review. *Journal of Economic Surveys*.

Schneider, E. B. and Ogasawara, K. (2018). Disease and child growth in industrialising Japan: Critical windows and the growth pattern, 1917-39. *Explorations in Economic History*, 69:64–80.

Schneider, E. B., Ogasawara, K., and Cole, T. J. (2021). Health Shocks, Recovery, and the First Thousand Days: The Effect of the Second World War on Height Growth in Japanese Children. *Population and Development Review*, 47(4):1075–1105.

Schünemann, H. J., Higgins, J. P. T., Vist, G. E., Glasziou, P., Akl, E. A., Skoetz, N., and Guyatt, G. H. (2019). Chapter 14: Completing 'Summary of Findings' Tables and Grading the Certainty of Evidence. In Higgins, J., Thomas, J., Chandler, J., Cumpston, M., Li, T., Page, M., and Welch, V., editors, *Cochrane Handbook for Systematic Reviews of Interventions*, pages 375–402. John Wiley & Sons, Chichester (UK).

Shamssain, M. H. (1991). Growth of Normal Urban Black Southern African Children Aged 6–19 Years. *Journal of Tropical Pediatrics*, 37(1):4–12.

Singh, S., Sidhu, L., and Malhotra, P. (1987). Growth performance of Punjabi children aged 6–12 years. *Annals of Human Biology*, 14(2):169–179.

Smit, P. J., Potgieter, J. F., and Fellingham, S. A. (1967). Body measurement of school children of four racial groups in Pretoria. *South African medical journal*, 41(35):868–90.

Smith, H., Meditz, S. W., and Merrill, T. (1994). The economy. In Meditz, S. W. and Merrill, T., editors, *Zaire: A country study*, pages 135 – 200. US Government Printing Office, Washington, DC.

Steckel, R. (1986). A peculiar population: The nutrition, health, and mortality of American slaves from childhood to maturity. *Journal of Economic History*, 46(3):721–741.

Steckel, R. H. (1987). Growth depression and recovery: the remarkable case of American slaves. *Annals of Human Biology*, 14(2):111–132.

Steckel, R. H. (1996). Percentiles of Modern Height Standards for Use in Historical Research. *Historical Methods*, 29(4):157–166.

Stevens, G. A., Finucane, M. M., Paciorek, C. J., Flaxman, S. R., White, R. A., Donner, A. J., Ezzati, M., and Growth), o. b. o. N. I. M. S. G. C. (2012). Trends in mild, moderate, and severe stunting and underweight, and progress towards MDG 1 in 141 developing countries: a systematic analysis of population representative data. *Lancet*, 380(9840):824–834.

Szreter, S. and Mooney, G. (1998). Urbanization, Mortality, and the Standard of Living Debate: New Estimates of the Expectation of Life at Birth in Nineteenth-century British Cities. *The Economic History Review*, 51(1):84 – 112.

Tanner, J. M. (1981). *A history of the study of human growth.* Cambridge University Press, Cambridge.

Twiesselman, F. (1957). *De la croissance des écoliers noirs de Léopoldville, entre la 6e et la 17e année d'âge.* Academie royale de sciences coloniales. Classe des science naturelles, Bruxelles.

Unicef, WHO, and World Bank Group (2021). Technical Notes from the Background Document for Country Consultations on the 2021 edition of the UNICEF-WHO-World Bank Joint Malnutrition Estimates. Technical report, Geneva.

Unicef, WHO, and World Bank Group (2023). Levels and trends in child malnutrition: Key findings of the 2023 edition. Technical report, Geneva.

UNICEF/WHO/World Bank (2023). Joint Child Malnutrition Estimates Expanded Database: Stunting (Survey Estimates) [Dataset]. https://data.unicef.org/wp-content/uploads/2019/04/UNICEF_WHO_WB_Global_Expanded_Databases_Stunting_May_2023.xlsx. Accessed July 2023.

van Braekel, G. (1959). Etude sur la robusticité et la croissance des écoliers noirs d'Elisabehtville. *Annales de la Société Belge de Médicine Tropicale*, 39(1):201 – 222.

Van Hees, J.-N., Van Brussel, M., and Eeckels, R. (1966). Contribution à l'étude de l'état de santé des écoliers de Kinshasa. Influence du mileu socio-économique. *Annales des Sociétés belges de Médecine tropicale de Parasitologie et de Mycologie*, 46(6):709 – 726.

van Loon, H., Saverys, V., Vuylsteke, J., Vlietinck, R. F., and Eeckels, R. (1986). Nutritional anthropometry in children from 0 to 6 years of age in different geographical areas. *Annals of Tropical Paediatrics*, 6(1):79–92.

Victora, C. G., de Onis, M., Hallal, P. C., Blossner, M., and Shrimpton, R. (2010). Worldwide Timing of Growth Faltering: Revisiting Implications for Interventions. *Pediatrics*, 125(3):e473–e480.

Walker, A. R. and Walker, B. F. (1977). Studies on increases in growth rate of South African Black schoolchildren and their significance to health. *South African medical journal = Suid-Afrikaanse tydskrif vir geneeskunde*, 51(20):707–12.

Wells, J. C. K. (2017). Worldwide variability in growth and its association with health: Incorporating body composition, developmental plasticity, and intergenerational effects. *American Journal of Human Biology*, 29(2):e22954 – 16.

WHO (2006). *WHO Child Growth Standards: Length/height-for-age, weight-for-age, weight-for-length, weight-for-height and body mass index-for-age: Methods and Development.*

Yang, H. and de Onis, M. (2008). Algorithms for converting estimates of child malnutrition based on the NCHS reference into estimates based on the WHO Child Growth Standards. *BMC Pediatrics*, 8(1):19.
